# Supplementary material for: Ex Vivo Vascular Imaging and Perfusion Studies of Normal Kidney and Tumor Vasculature
Source: Cancers (Basel). 2024 May 20;16(10):1939. doi: 10.3390/cancers16101939 (PMC11119251; doi:10.3390/cancers16101939)
Supplement: Supplementary file 1 [file cancers-16-01939-s001.zip › cancers-2993788-supplementary.pdf]

H1

Discarded due to technical reasons.

RCC case H2

Sex: Male
Age at surgery: 51 years
Survival from surgery: 4 years
Cause of death: Renal cancer
Initial stage: 6.5cm pT1b N0 M0
Tumour volume: 137 cm^3
Tumour type: clear cell renal cell carcinoma, ISUP grade: 2
Specimen weight: 382 g
Perfusion pressure: 34 mmHg. Perfusate flow: 23 mL/min.

Specimen PRU: 1.47
Cortical tissue PRU: 0.63 n=1
Tumour tissue PRU span: 0.93-9.55 n=5

No contrast infusion No autoradiogram

Fresh section: Bar 100 mm

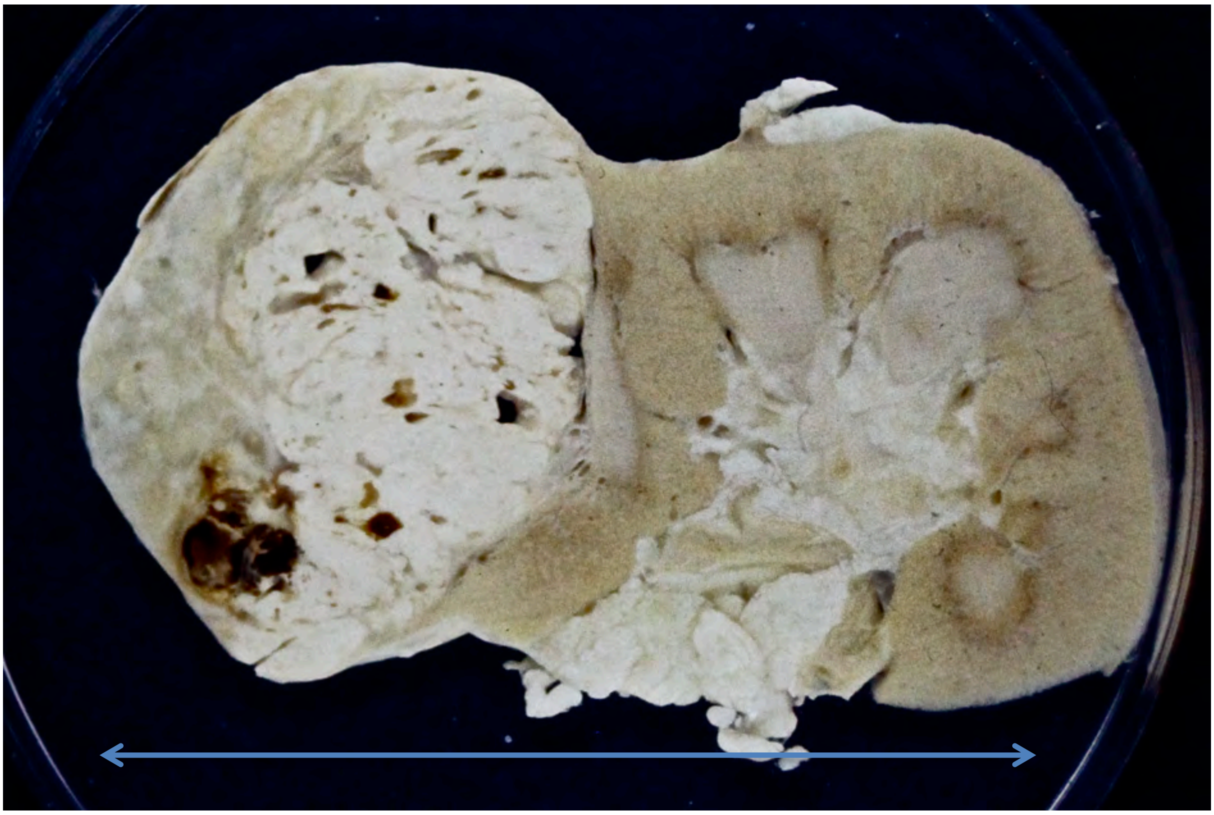

Dark-field CD31

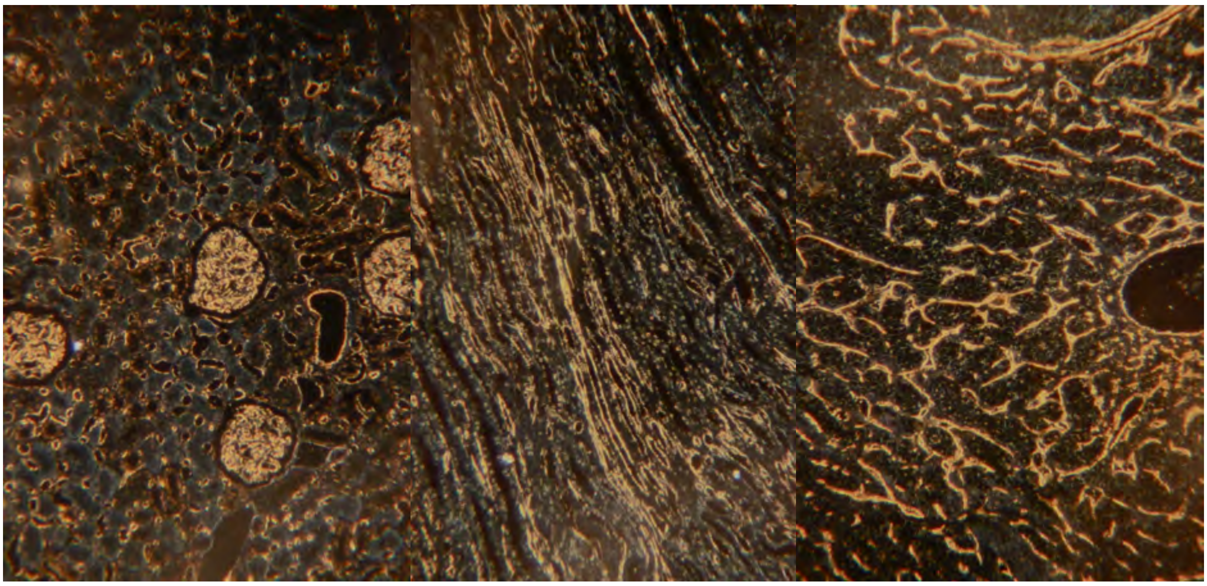

Cortex 2:1 PRU 0.63   Medulla 2:2 PRU 9.55   Tumour 2:7 PRU 0.93

Tumour H/E 20X magnification

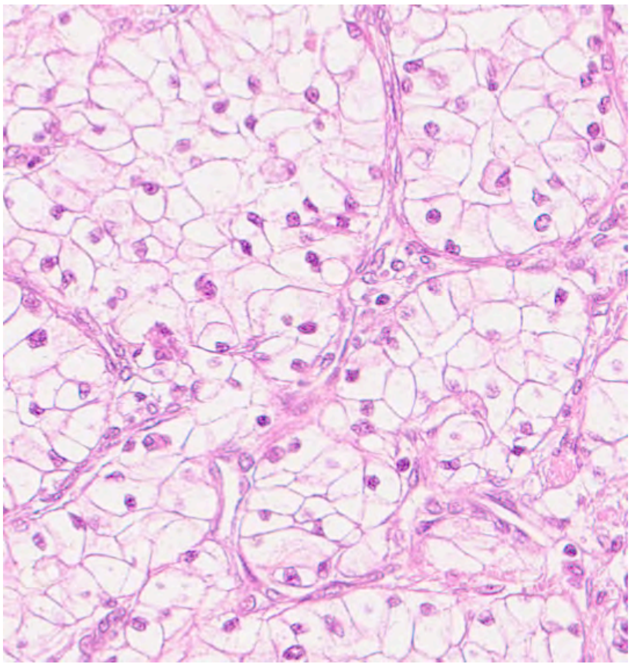

2:7 20x PRU 0.93

RCC case H3

Sex: Male
Age at surgery: 66 years
Survival from surgery: 1 year
Cause of death: Renal cancer
Initial stage: Tx N0 M0
Tumour type: Clear +intermed polymorph CCRCC ISUP grade 1
Tumour volume: 151 cm^3

Specimen weight: 341g
Perfusion pressure: 27 mmHg Perfusate flow: 80 mL/min
Specimen PRU: 0.34
Cortical tissue PRU: 0.05 n=1
Tumour tissue PRU span: 0.12-29.1 n=4

No contrast infusion No autoradiogram
Fresh section: Bar 100 mm

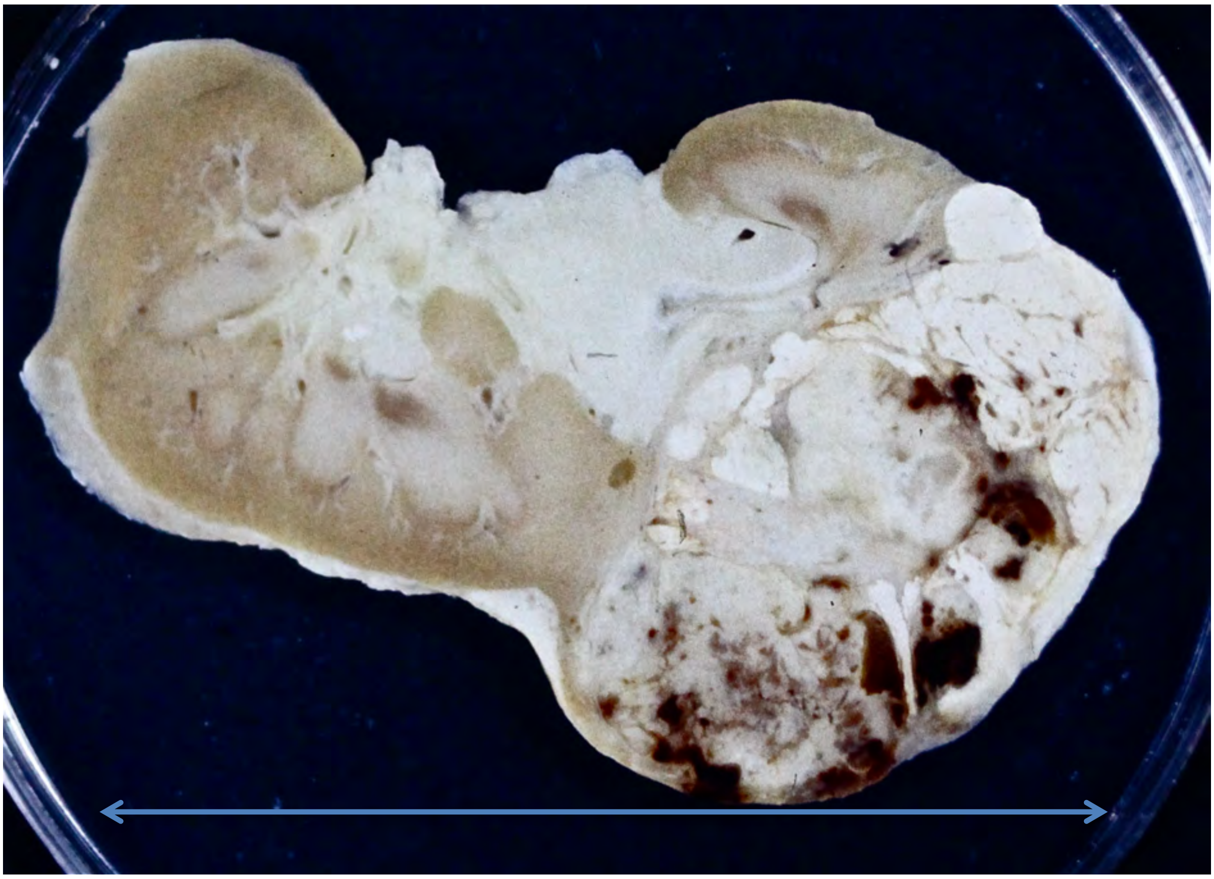

Dark-field CD31

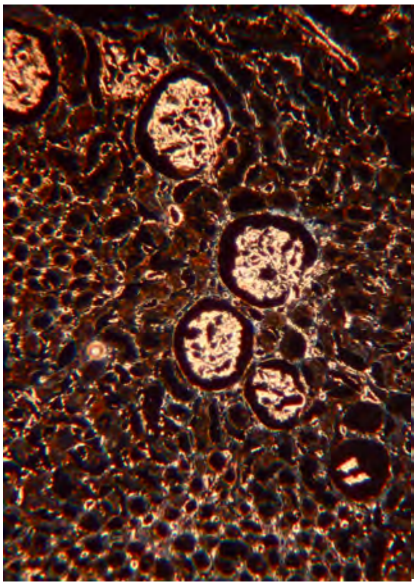

Cortex 3:1 PRU 0.05

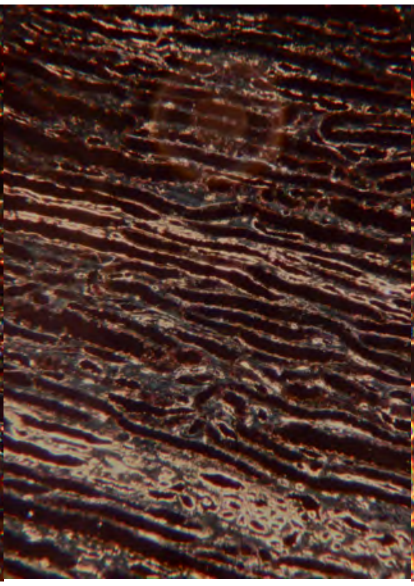

Medulla 3:2 PRU 0.53

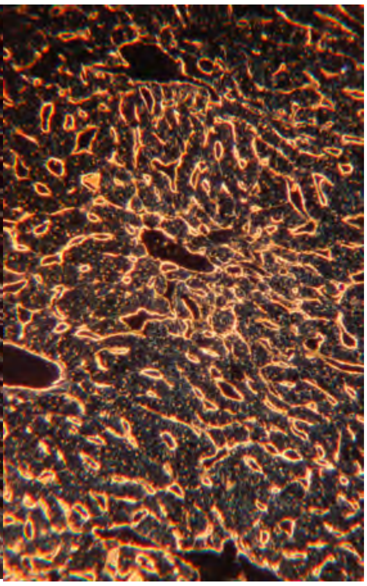

Tumour 3:5 PRU 0.12

Tumour HE 20x

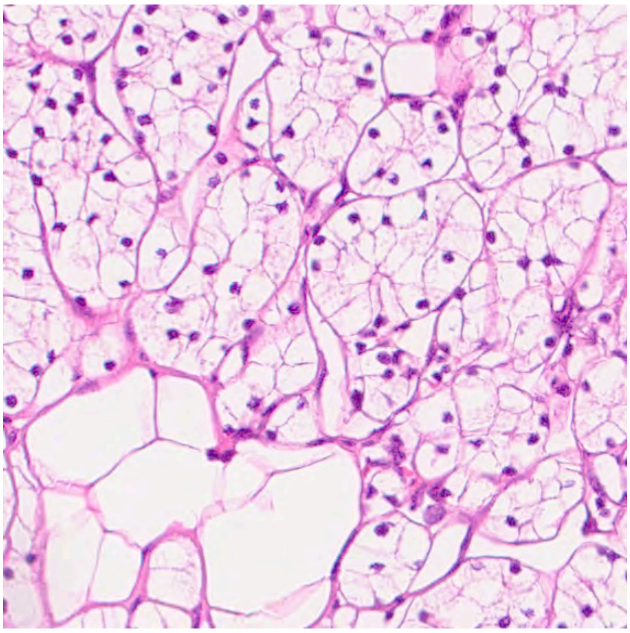

3:5 20x PRU 0.12

H4
Sex: Female
Age at surgery: 73 years
Survival from surgery: 0 year
Cause of death: Renal cancer
Initial stage: Tx N1 M1
Tumour type: nulear polymorph, mitoses Eosinophilic with necrosis ISUP grade 4
Tumour volume: 191 cm^3

Specimen weight: 513 g
Perfusion pressure: 51 mmHg Perfusate flow: 43 mL/min
Specimen PRU: 1.19
Cortical tissue PRU: 0.19 n=1
Tumour tissue PRU span: 4.64-510 n=4
No contrast infusion No autoradiogram
Fresh section

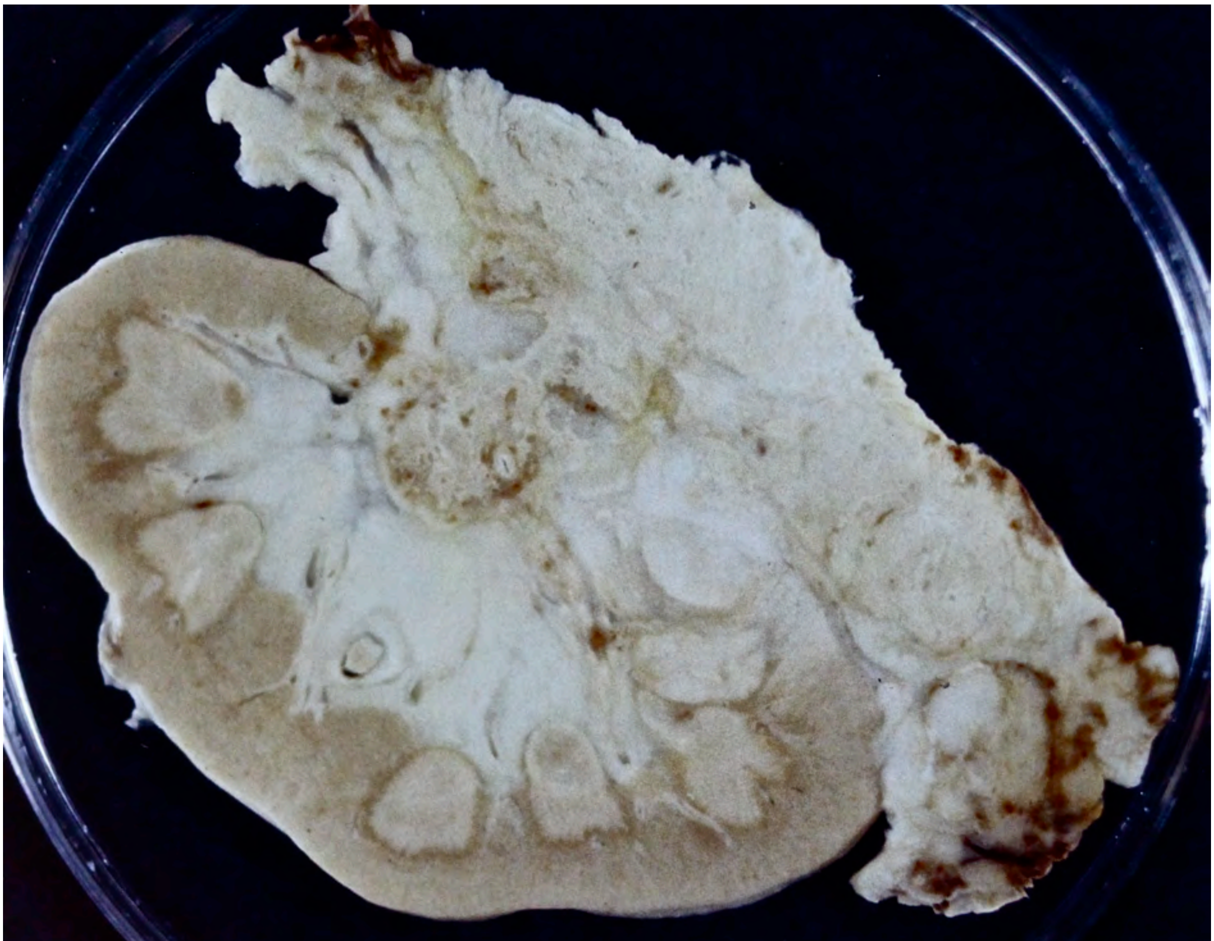

Dark.field CD31

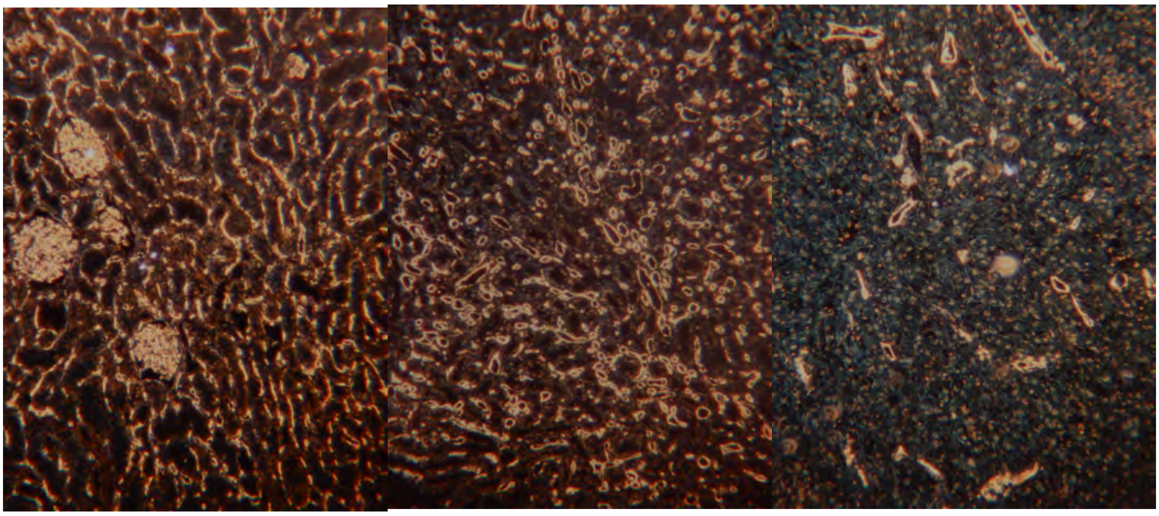

Cortex 4:1 PRU 0.19 Medulla 4:2 PRU 1.46 Tumour 4:6 PRU 4.64

Tumour HE 20x

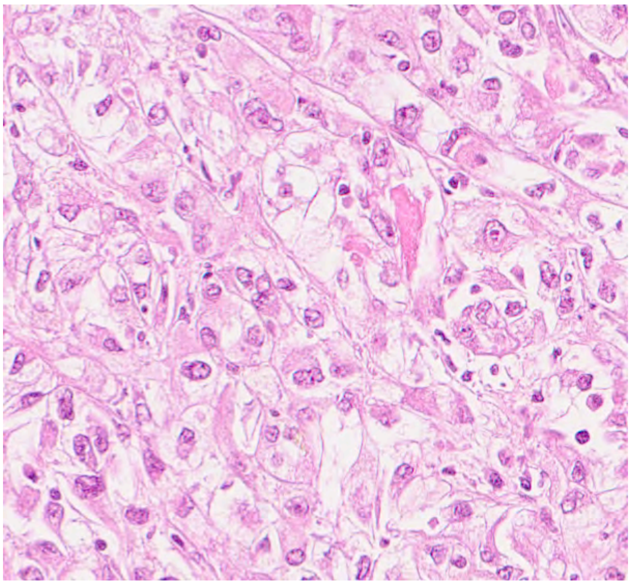

4:6 20x PRU 4.64

H5
Sex: Male
Age at surgery: 79 years
Survival from surgery: 0 year
Cause of death: Renal cancer
Initial stage: T 7x5 cm pT2 N1 M1
Tumour typeplasmic polymorph CCRCC eosinophilic ISUP grade3
Tumour volume: 123 cm^3

Specimen weight: 268 g
Perfusion pressure: 32 mmHg Perfusate flow: 39 mL/min
Specimen PRU: 0.82
Cortical tissue PRU: 0.21 n=1
Tumour tissue PRU span: 0.26-1.58 n=4
No contrast infusion No autoradiogram
Fresh section Bar: 100 mm

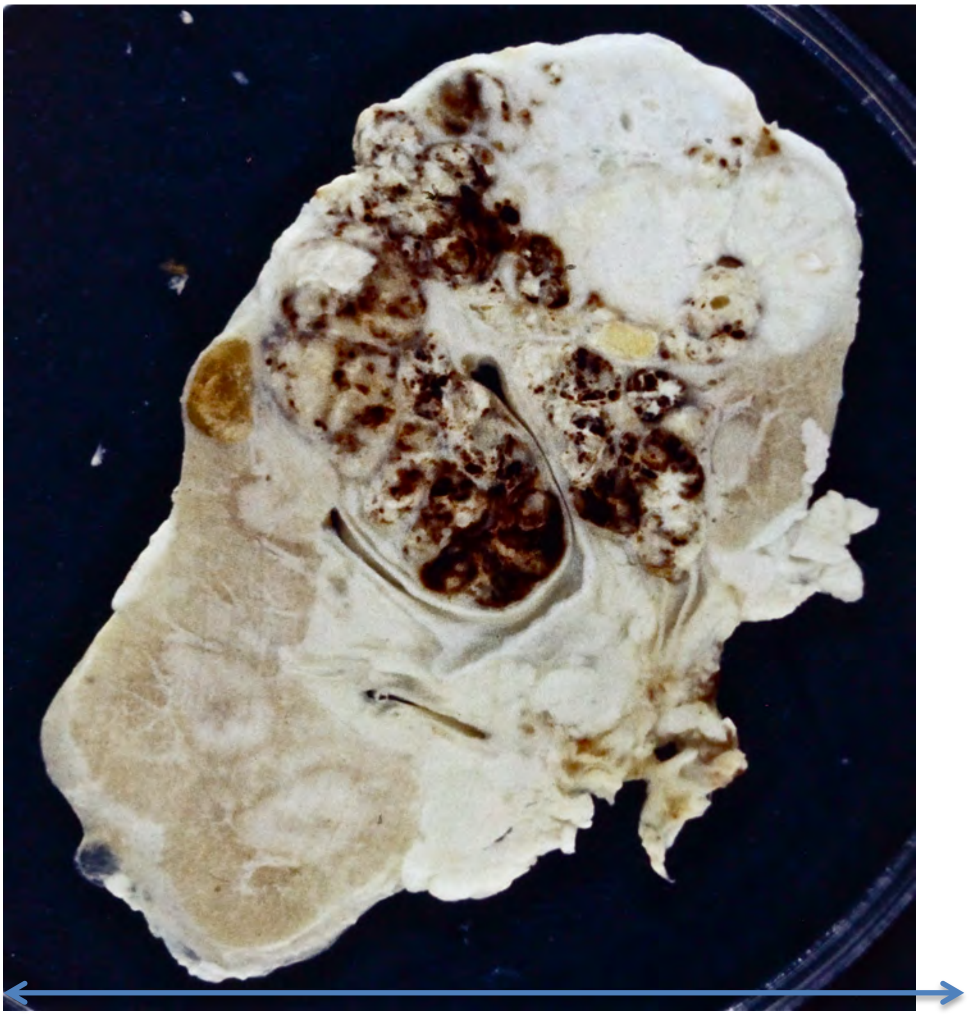

Dark-field CD31

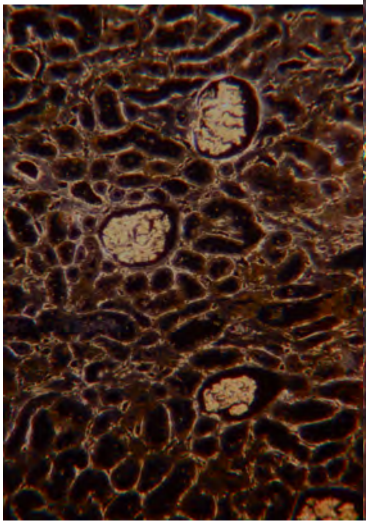

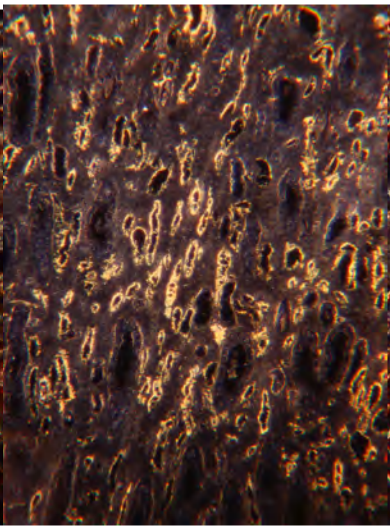

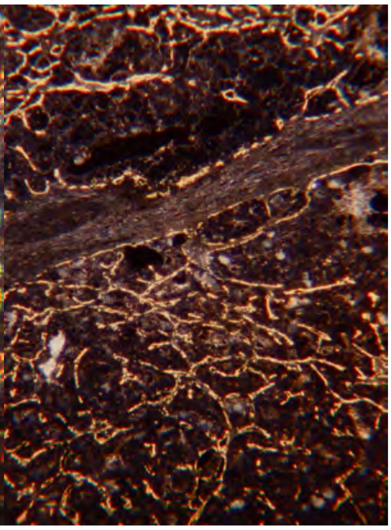

Cortex 5:6 PRU 0.21 Medulla 5:7 PRU 0.53 Tumour 5:5 PRU 0.26

Tumour HE 20x

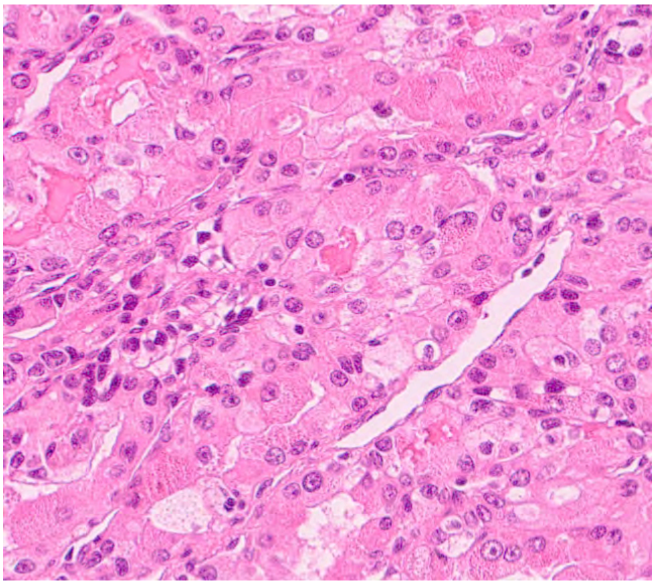

5:5 20x PRU 0.26

H6

Sex: Female
Age at surgery: 52 years
Survival from surgery: 5 years
Cause of death: Renal cancer
Initial stage: 10 cm T7.5 cm pT2 N0 M0
Tumour type: clear+ intermediate CCRCC ISUP grade 2
Tumour volume: 192 cm^3

Specimen weight: 539 g
Perfusion pressure: 28 mmHg Perfusate flow: 63 mL/min
Specimen PRU: 0.44
Cortical tissue PRU: 0.11 n=1
Tumour tissue PRU span: 0.29-26.7 n=3

No contrast infusion No angiogram

Fresh section: Bar 100 mm

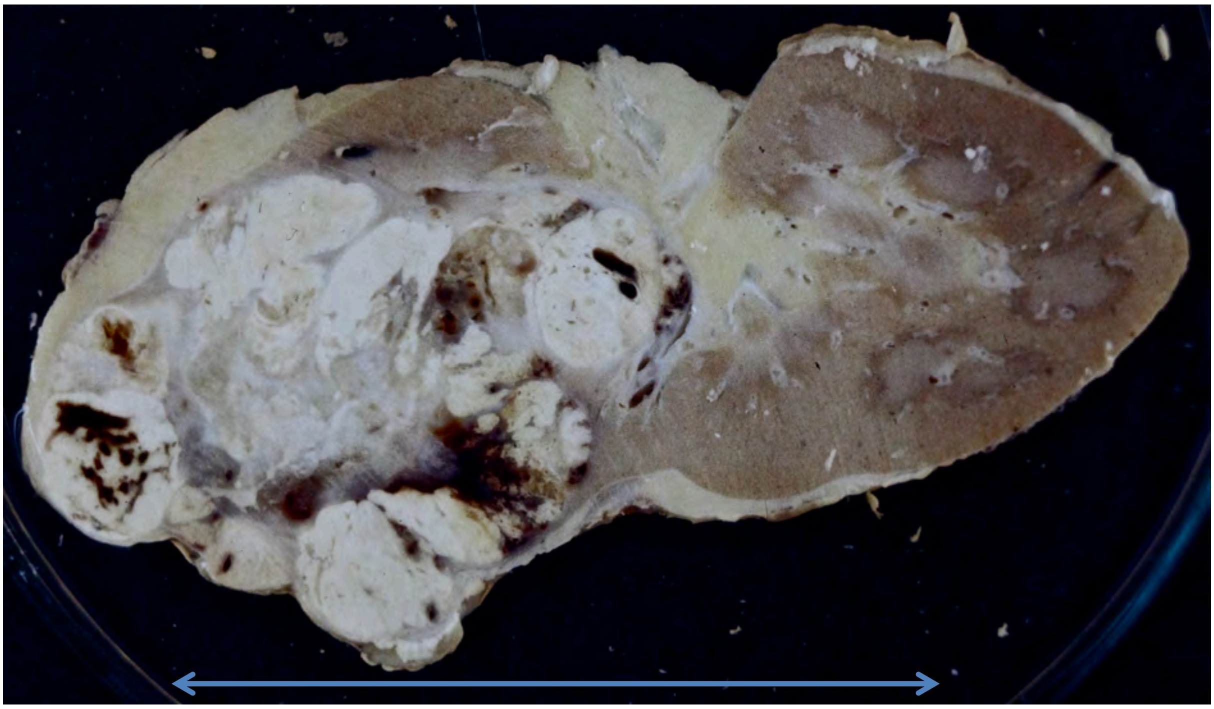

Dark-field CD31

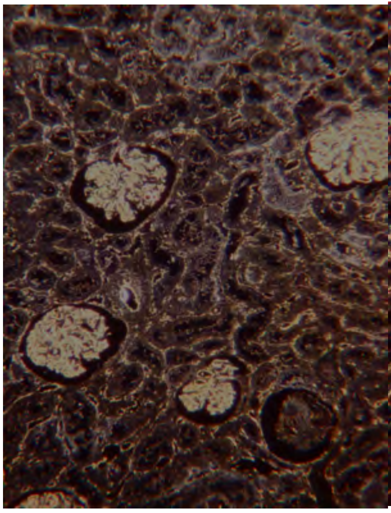
Cortex 6:4PRU 0.11

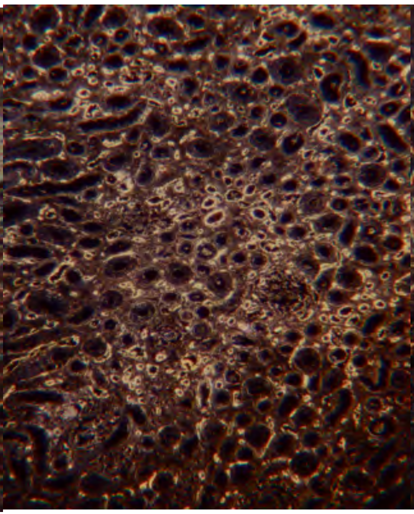
Medulla 6:5 PRU 0.57

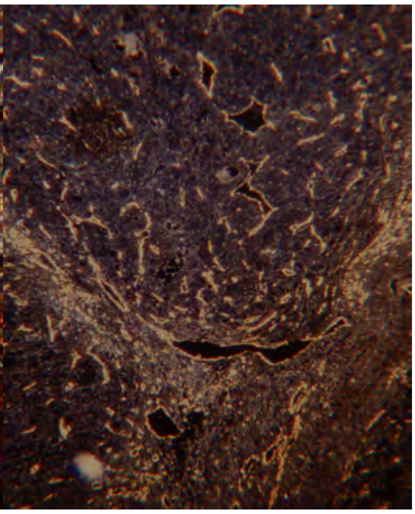
Tumour 6:1 PRU 0.49

Tumour HE 20x

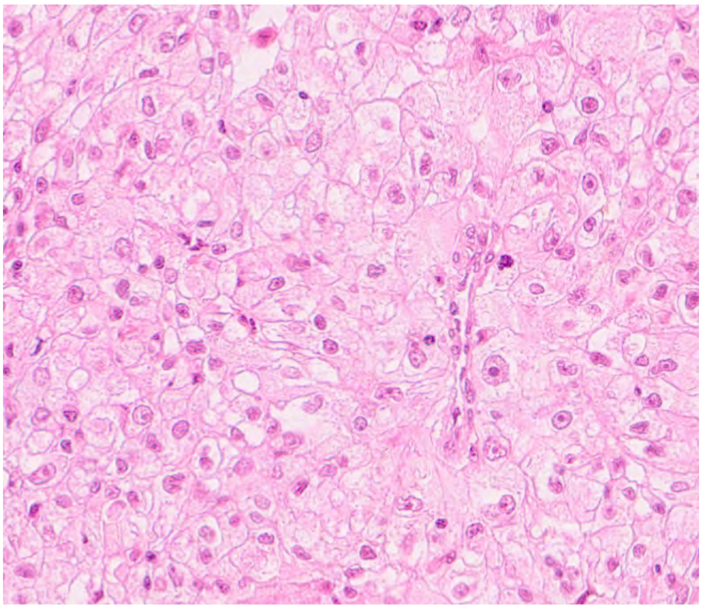
6:1 20x PRU 0.49

H7

Sex: Male
Age at surgery: 78 years
Survival from surgery: 0 year
Cause of death: Renal cancer
Initial stage: T5 cm pT1b N1M1
Tumour type: CCRCC ISUP grade 1
Tumour volume: 91 cm^3

Specimen weight: 290 g
Perfusion pressure: 27 mmHg Perfusate flow: 84 mL/min
Specimen PRU: 0.32
Cortical tissue PRU: 0.12 n=1
Tumour tissue PRU span: 0.16-10.0 n=3

Fresh section: Bar 100 mm

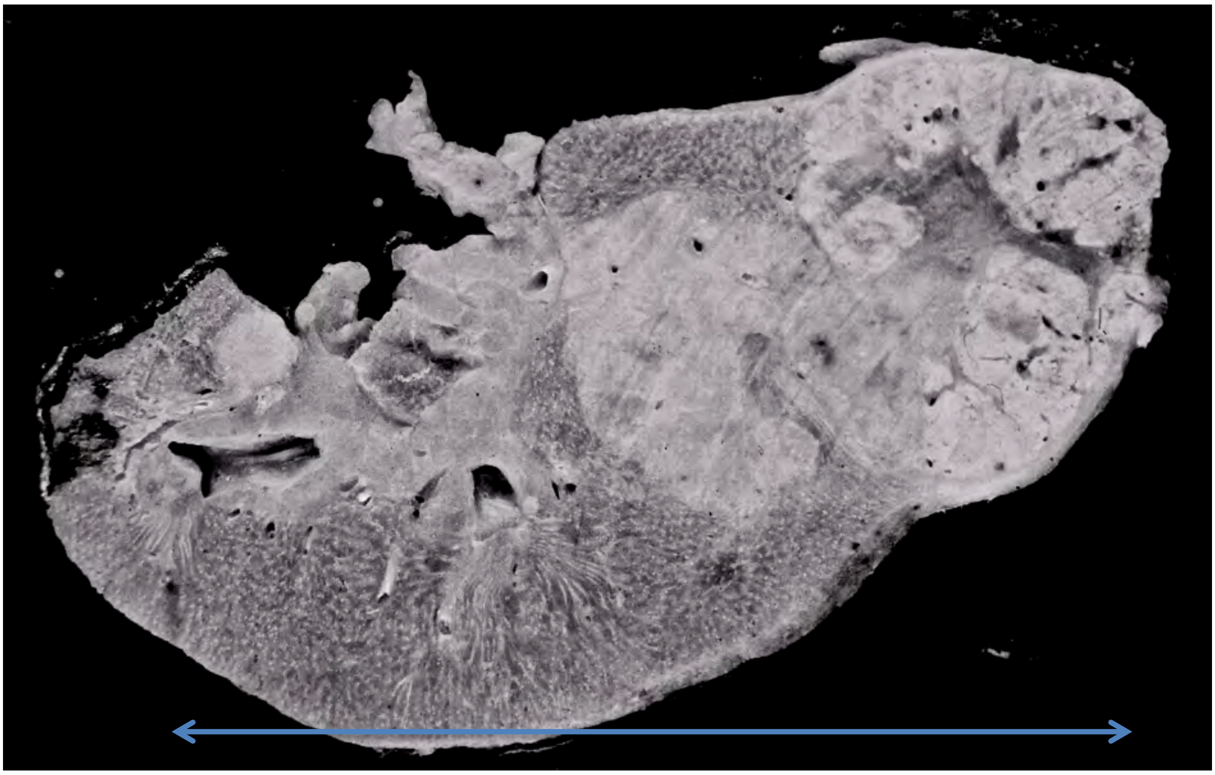

Angiography

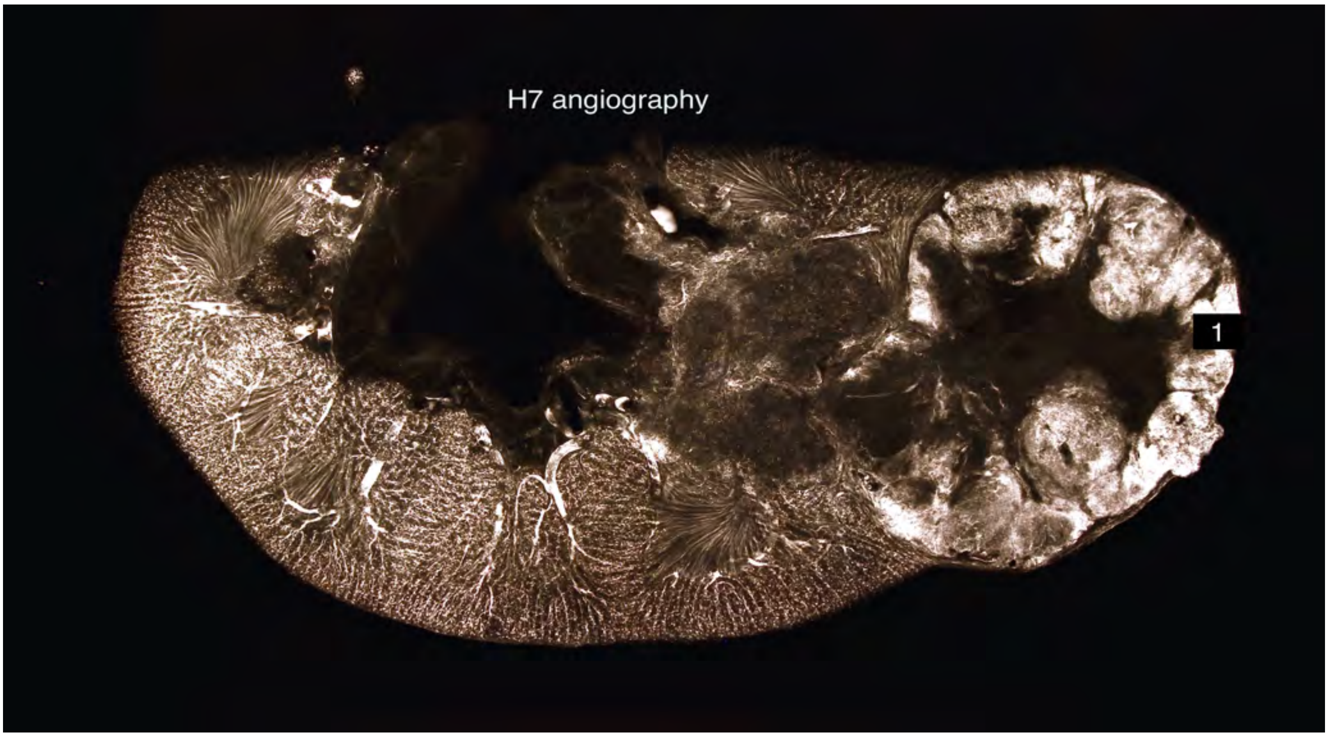

Autoradiography

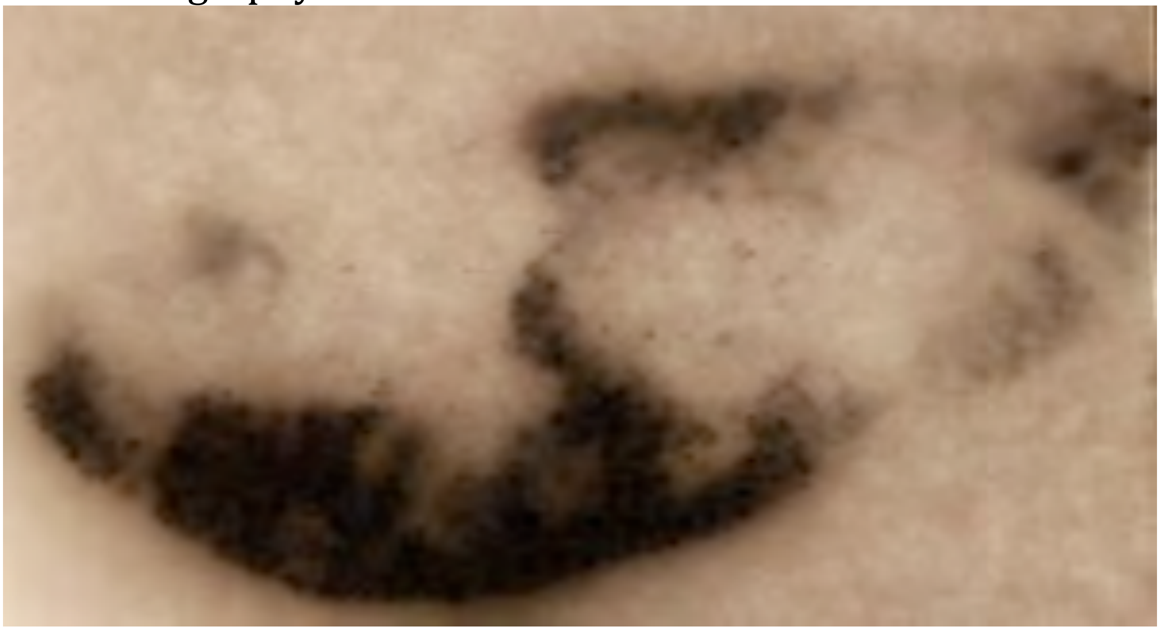

Dark-field macrophotography

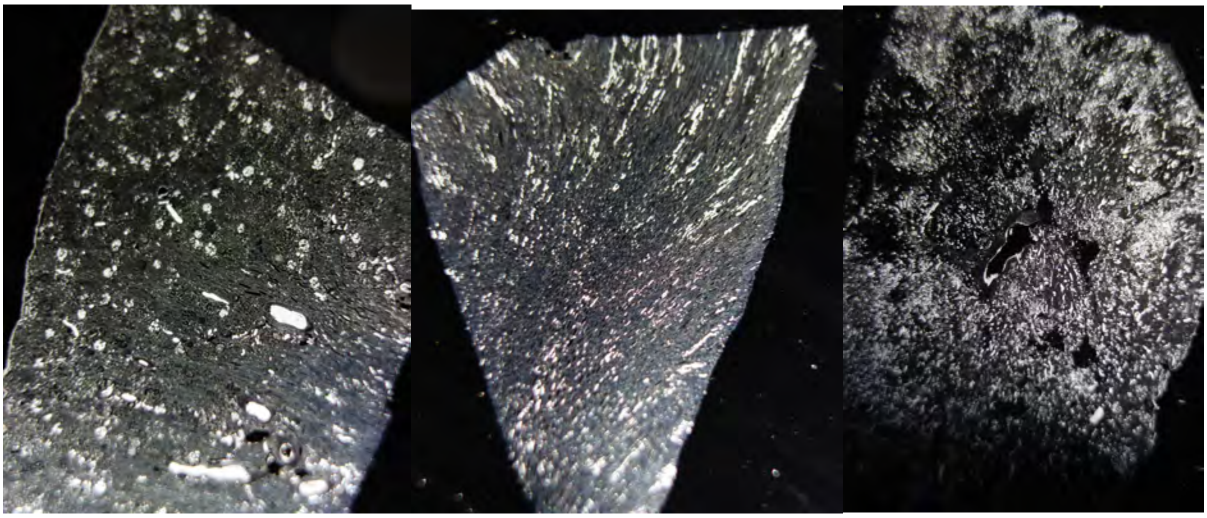

Cortex 7:5 PRU 0.12 Medulla 7:6 PRU 1.04 Tumour 7:1 PRU 0.69

Darkfield CD31

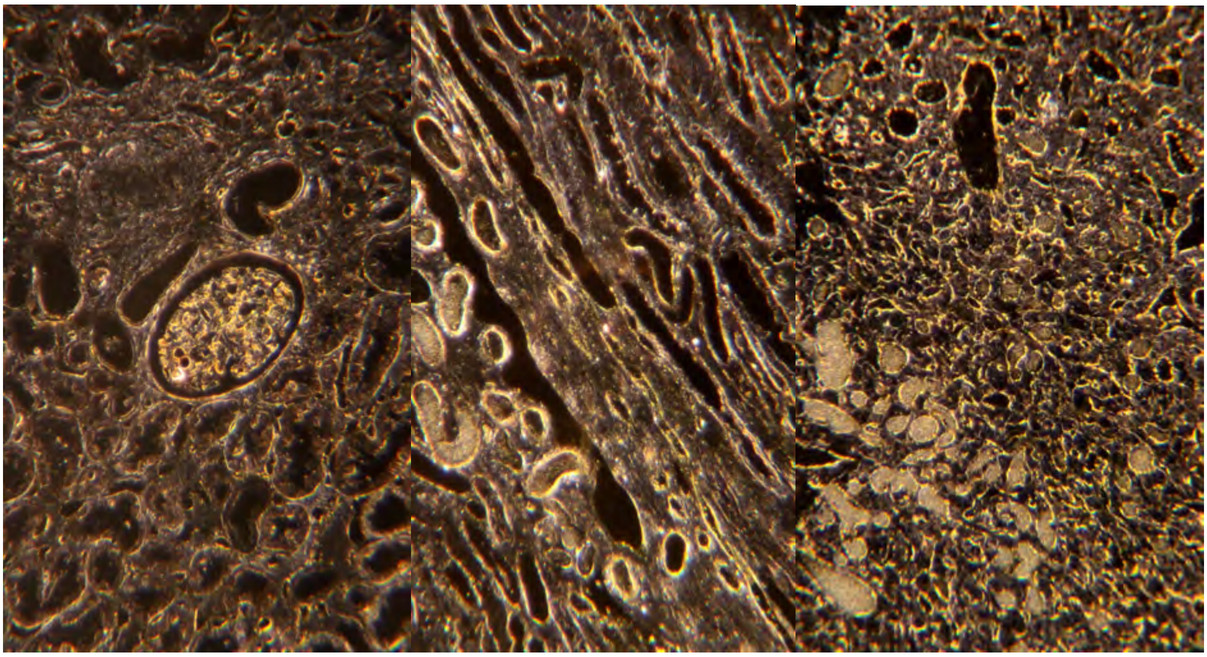

Cortex 7:5 PRU 0.12 Medulla 7:6 PRU 1.04 Tumour 7:1 PRU 0.69

Brightfield

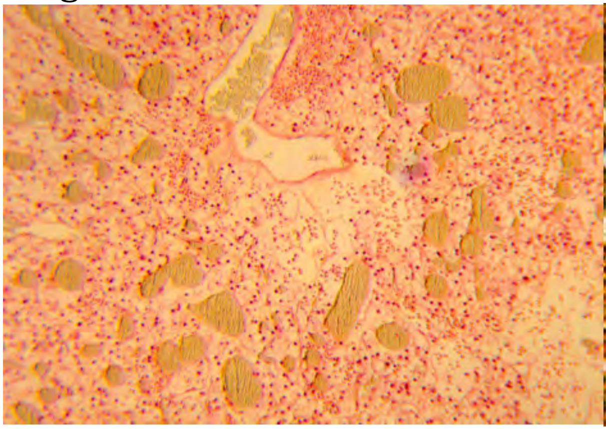

Darkfield

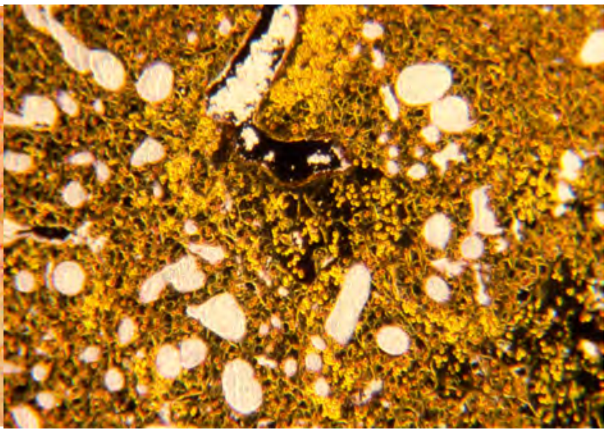

7:1 PRU 0.69

HE 20x

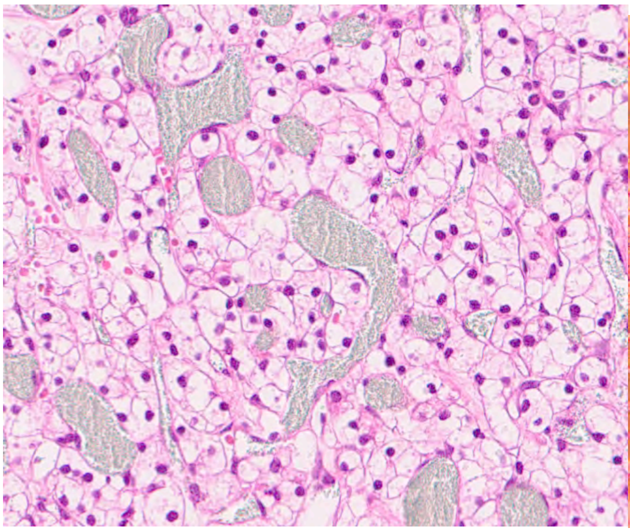

7:1 PRU 0.69

HE 16x

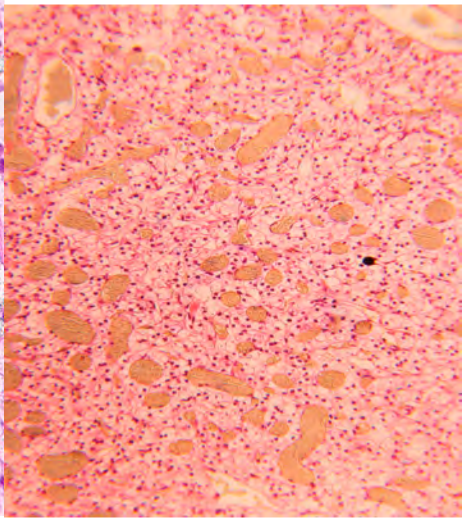

Observe the 15 um sphere

H8

Sex: Male
Age at surgery: 76 years
Survival from surgery: 12 years
Cause of death: other cause
Initial stage: 6 cm T6.5 cm NxM0
Tumour type: clear cell nuclear polymorph   Tumour grade: 1-2 ?
Tumour volume: 210 cm^3

Specimen weight: 289 g
Perfusion pressure: 43 mmHg   Perfusate flow: 83 mL/min
Specimen PRU: 0.52
Cortical tissue PRU: 0.18 n=1
Tumour tissue PRU span: 3.34-25   n=4

Fresh section: Bar 100 mm

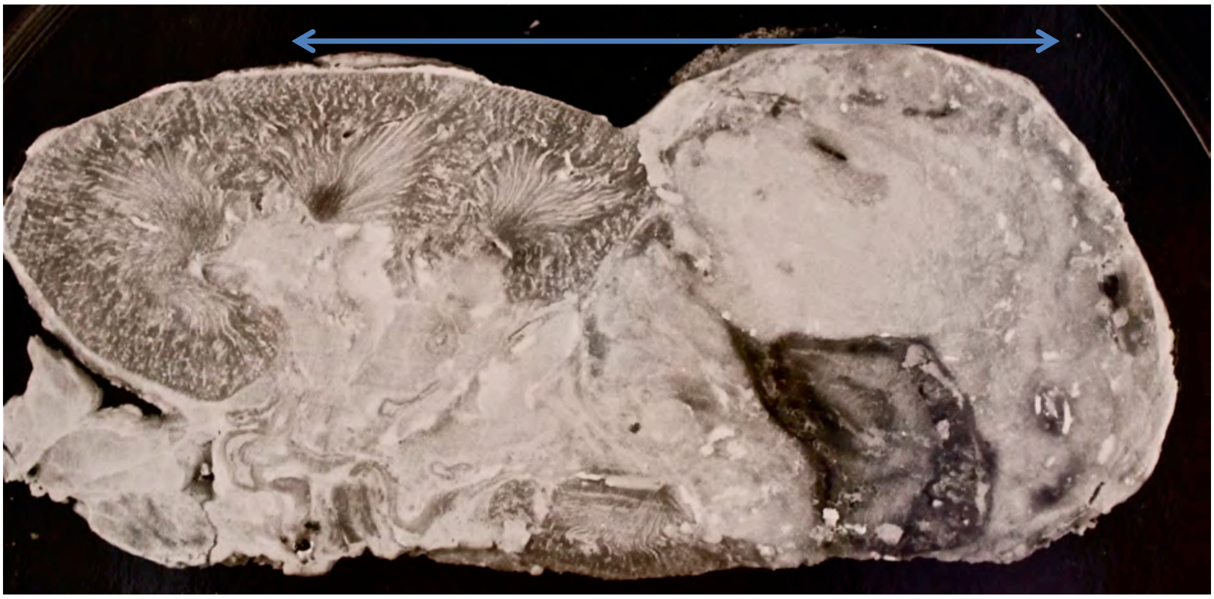

Angiography

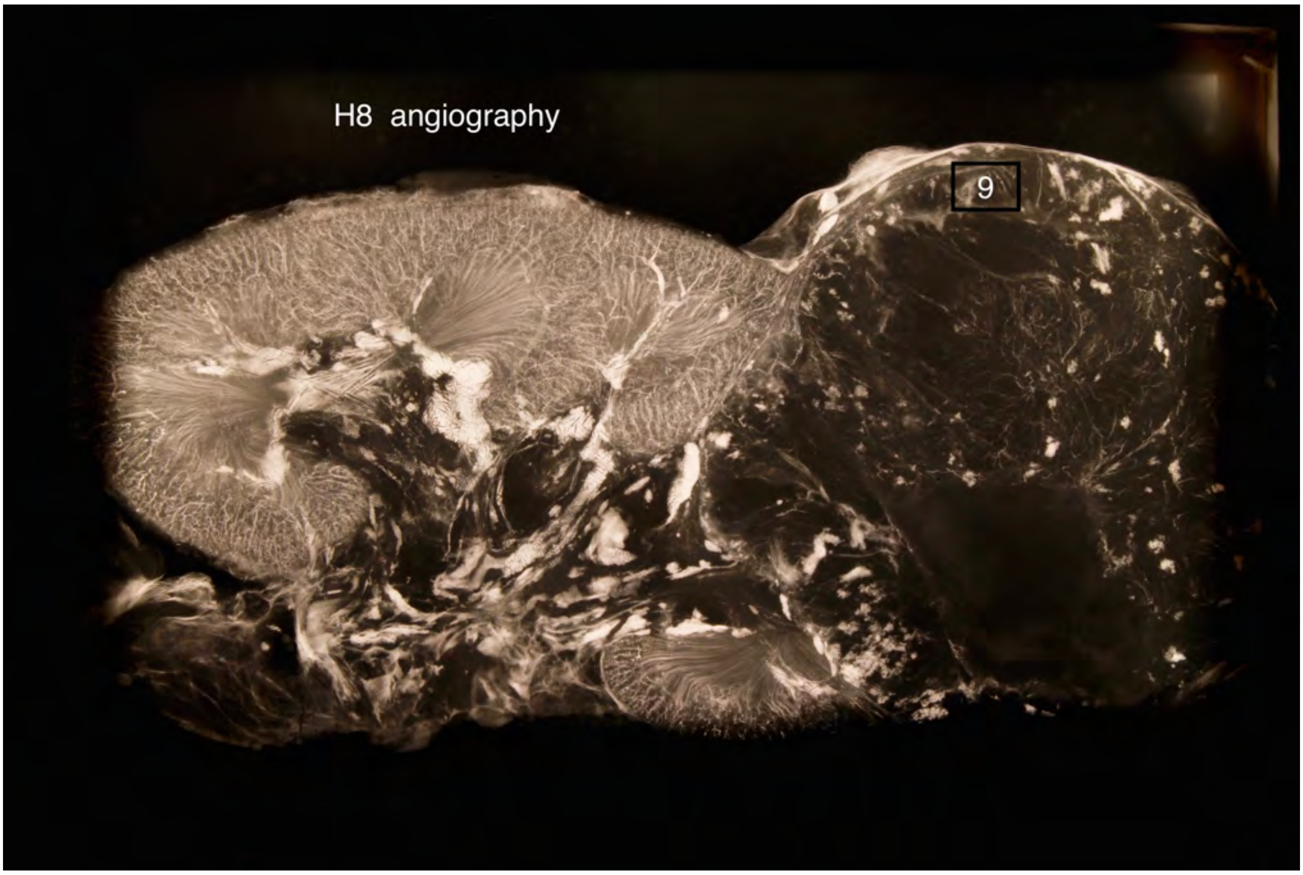

Autoradiography

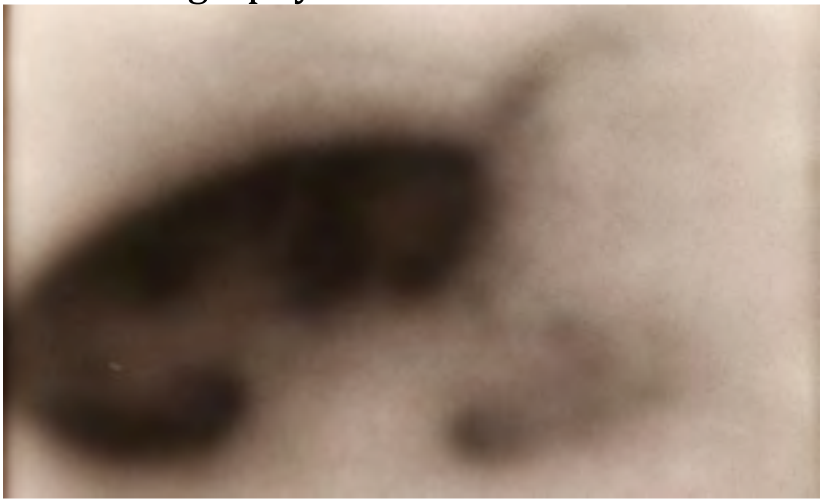

Dark-field macrophotography

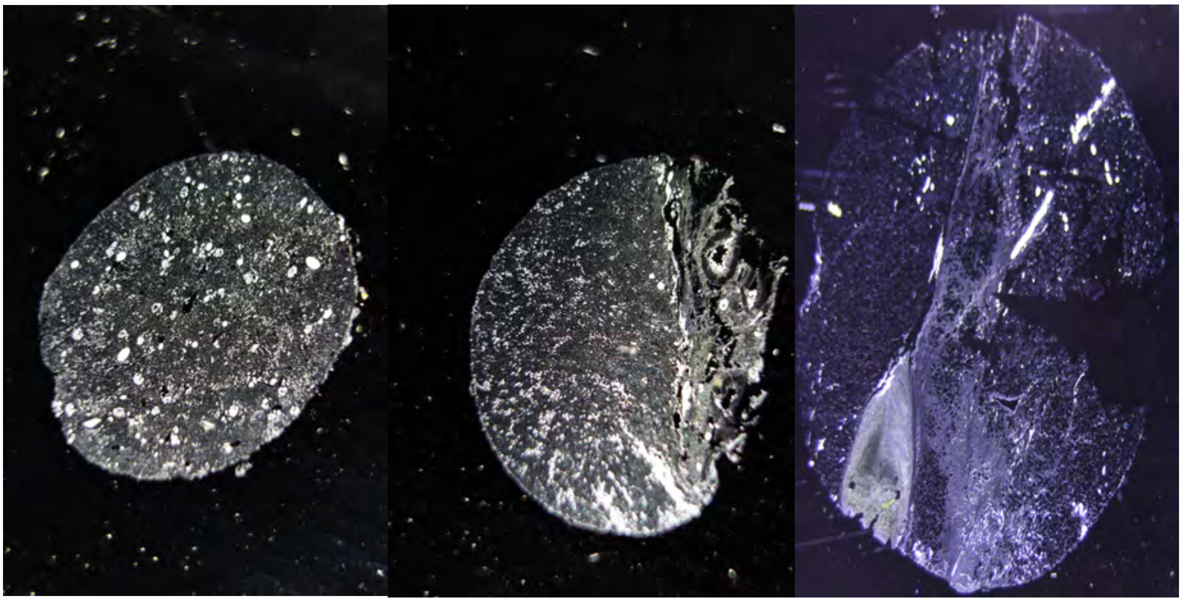

Cortex 8:1 PRU 0.18   Medulla 8:5 PRU 1.66 Tumour 8:9 PRU 3.34

Darkfield CD31

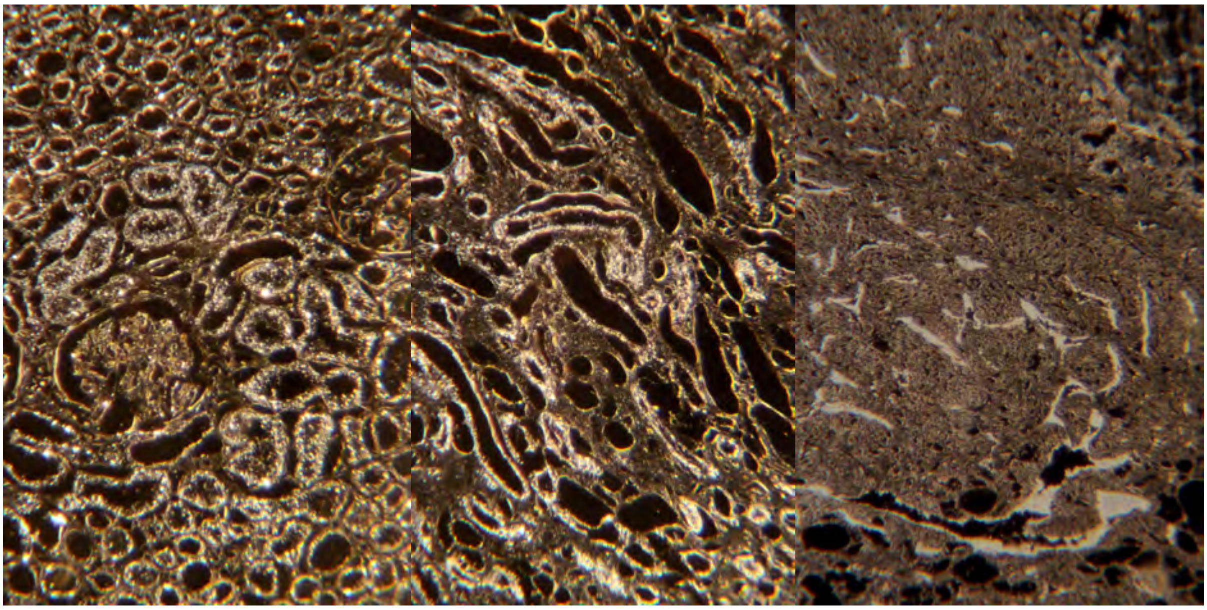

Cortex 8:1 PRU 0.18 Medulla 8:5 PRU 1.66 Tumour 8:9 PRU 3.34

H9

Sex: Male
Age at surgery: 65 years
Survival from surgery: 18 years
Cause of death: other cause
Initial stage: T9x6.5cm pT2 Nx M0
Tumour type: CCRCC ISUP grade 1
Tumour volume: 298 cm^3

Specimen weight: 169 g
Perfusion pressure: 28 mmHg Perfusate flow:103 mL/min
Specimen PRU: 0.27
Cortical tissue PRU: 0.17 n=1
Tumour tissue PRU span: 0.39-10.8 n=5

No contrast infusion No angiogram No autoradiogram
Fresh section: Bar 100 mm

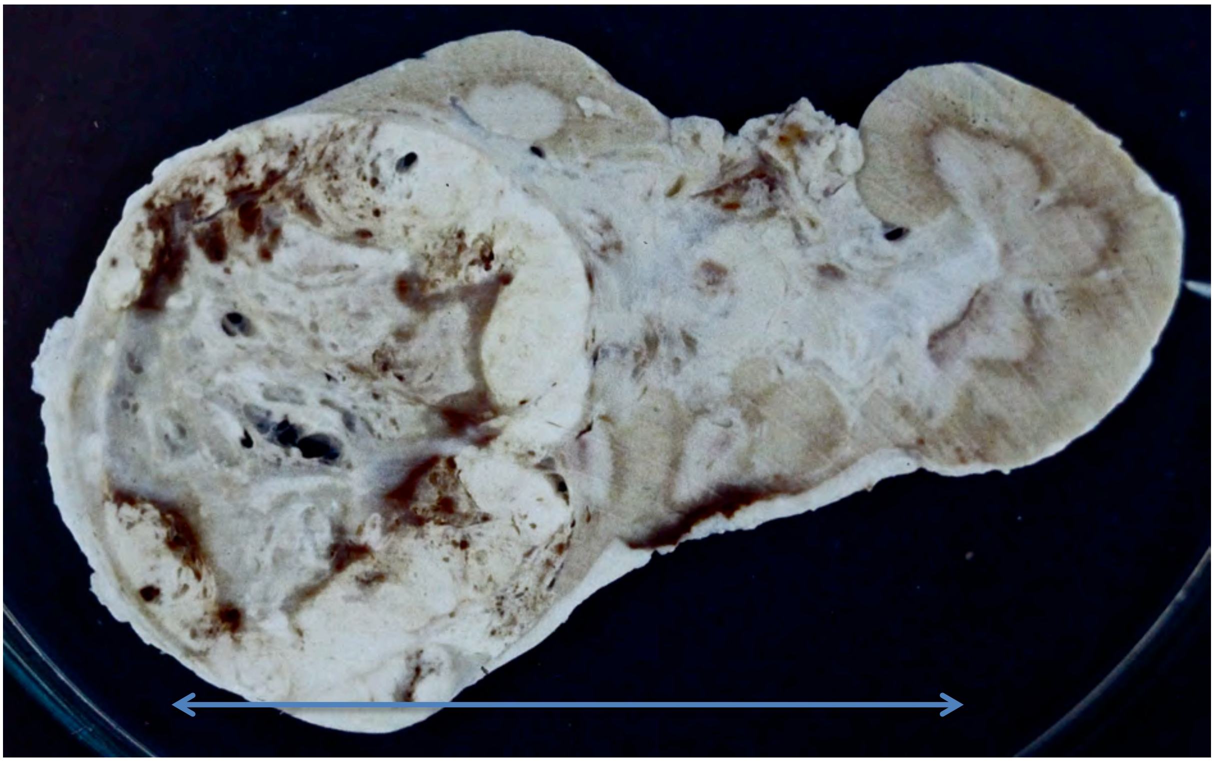

Darkfield CD31

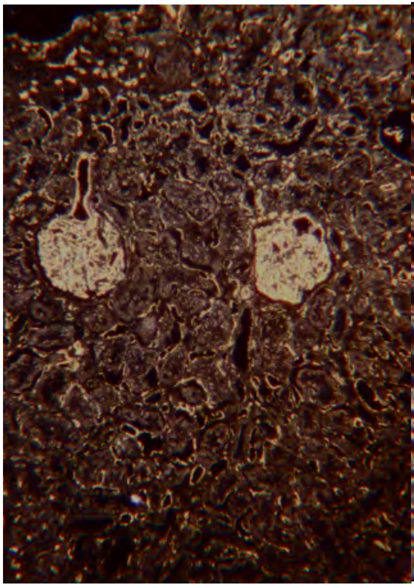 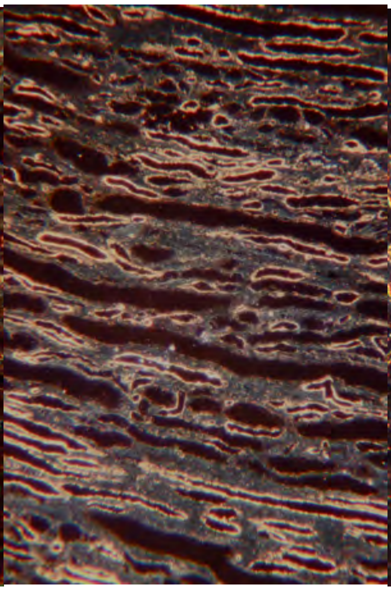 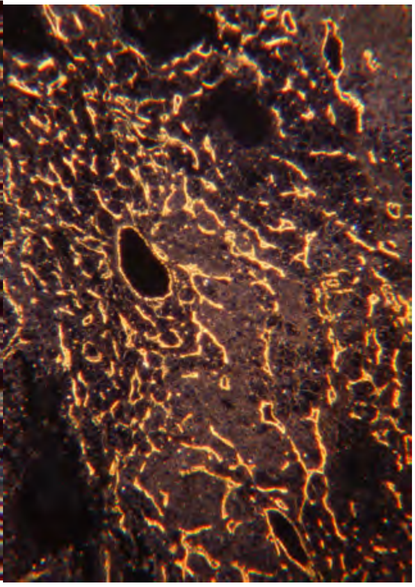

Cortex 9:1 PRU 0.17   Medulla 9:2 PRU 3.64 Tumour 9:5 PRU 0.39

Tumour HE 20x

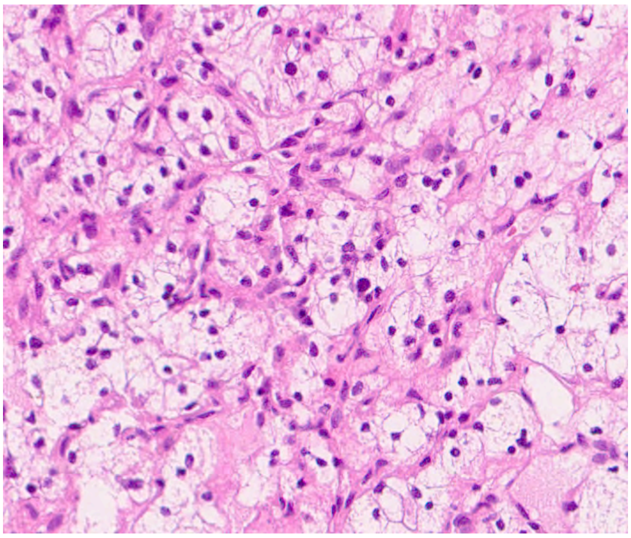

9.5 20x PRU 0.39

H10

Sex: Male
Age at surgery: 65 years
Survival from surgery: 2 years
Cause of death: Renal cancer
Initial stage: T16x10 cm pT2 Nx
Tumour type: intermediate-plasmic CCRCC ISUP grade 1
Tumour volume: 480 cm^3

Specimen weight: 1271 g
Perfusion pressure: 8 mmHg Perfusate flow: 14 mL/min
Specimen PRU: 0.57
Cortical tissue PRU: NE n=3
Tumour tissue PRU span: NE n=9
Reference withdrawal failed
Fresh section: Bar 100 mm

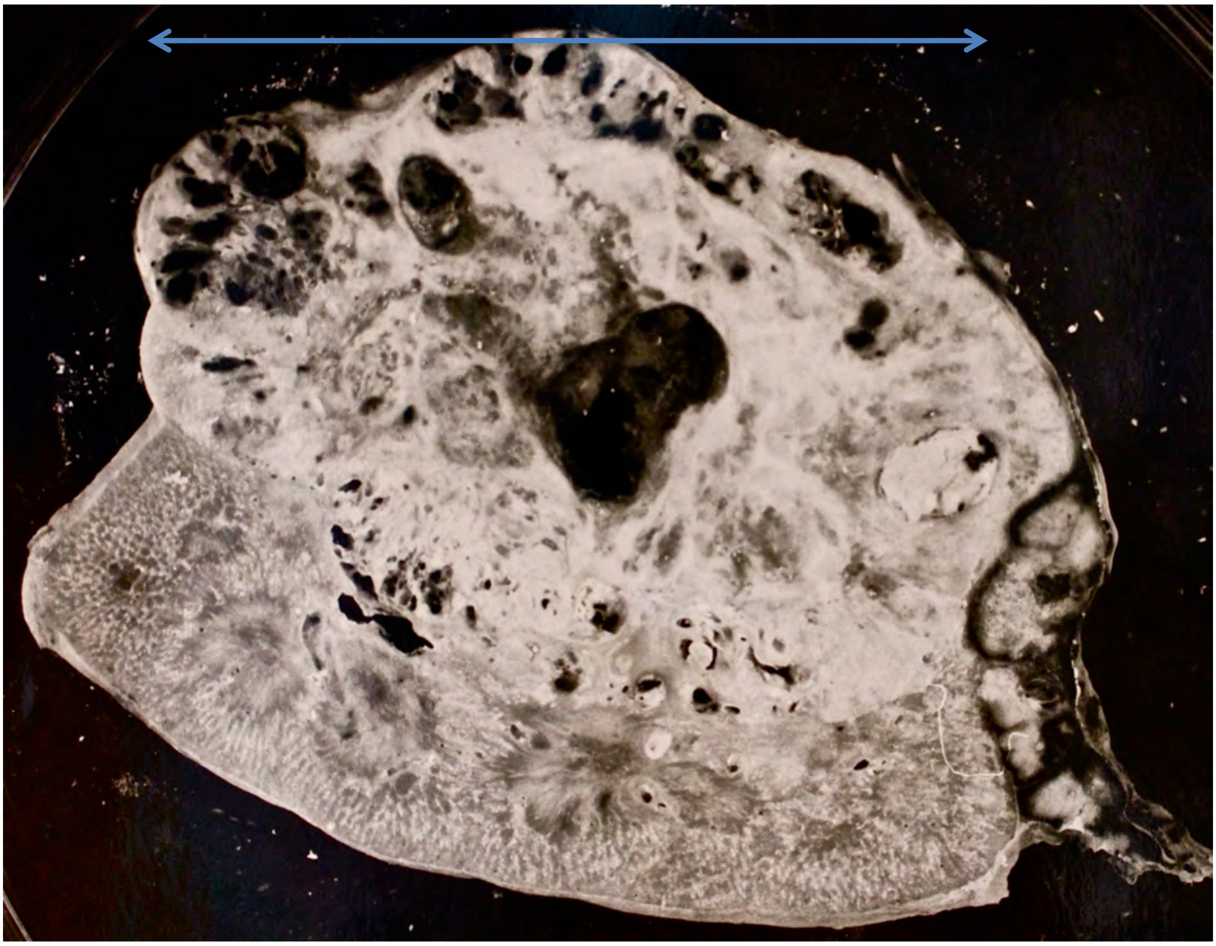

Angiography

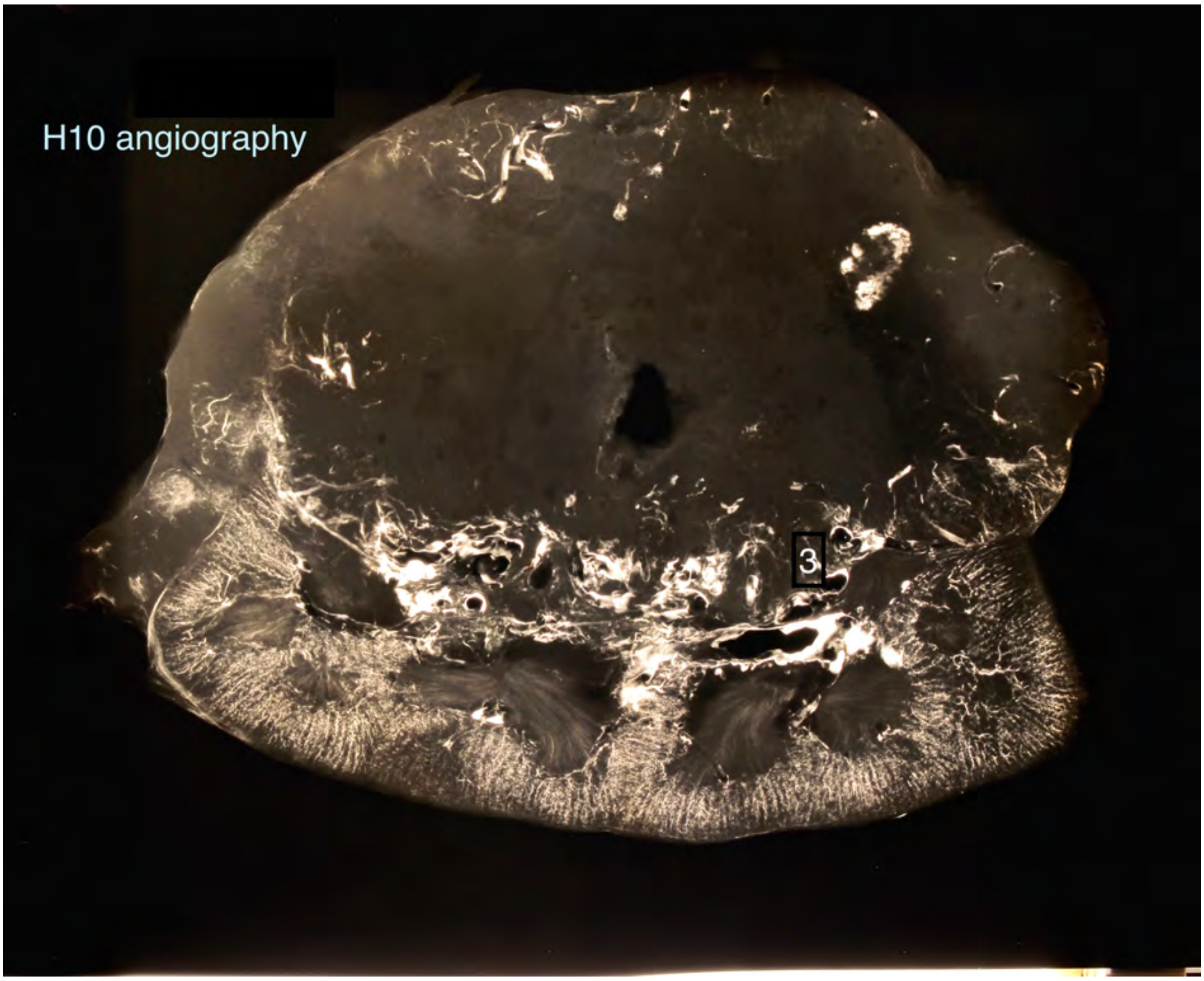

Autoradiography

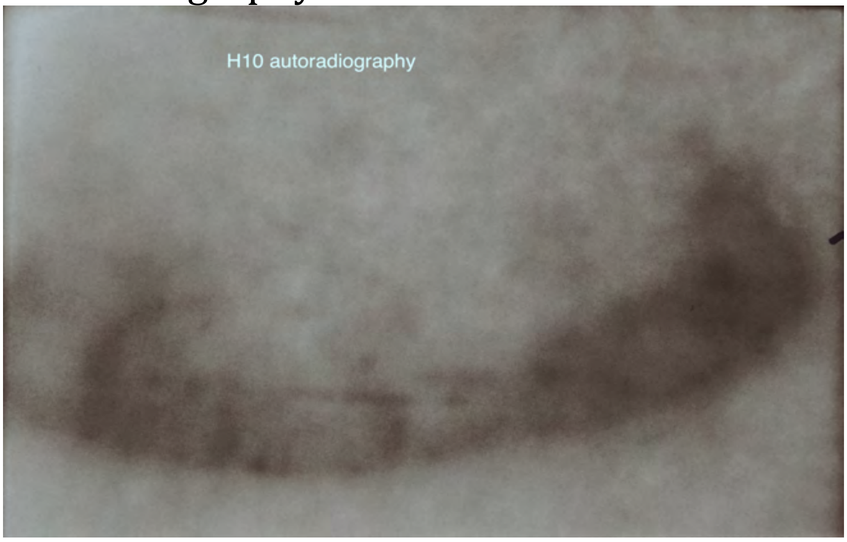

Darkfield CD31

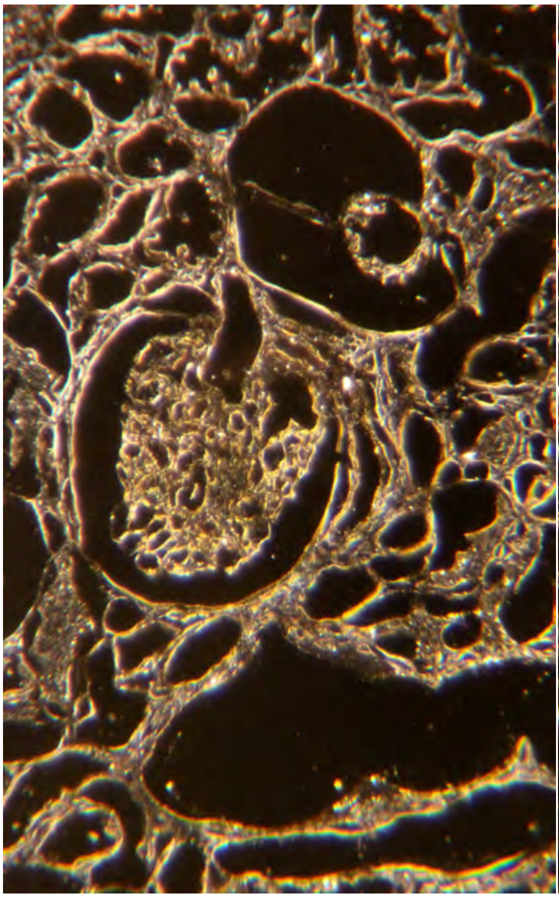

Cortex 10:12 PRU_{rel} 1

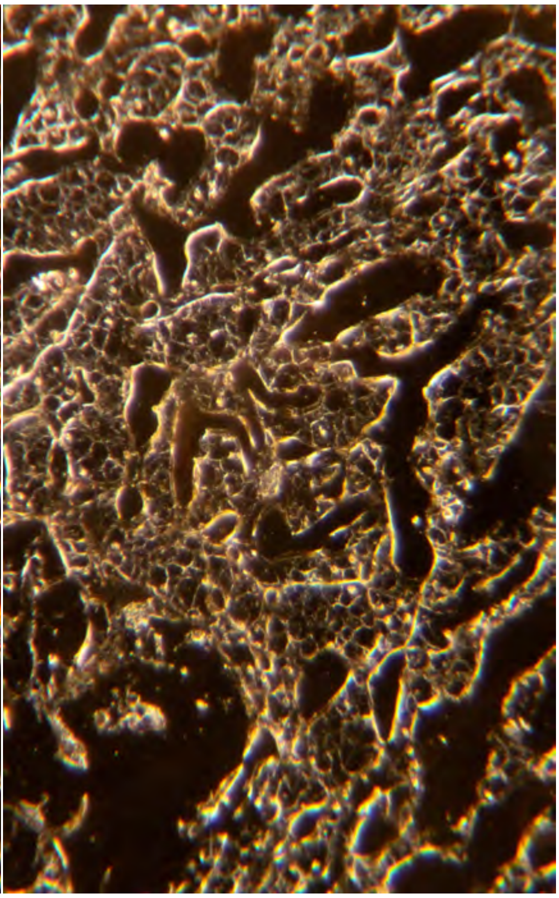

Tumour 10:3 PRU_{rel} 1.7

Brightfield

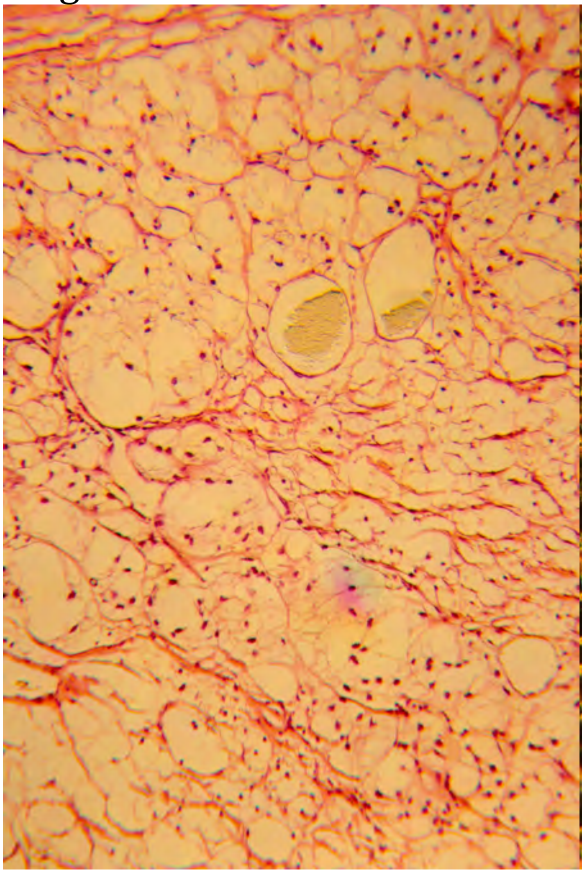

Darkfield

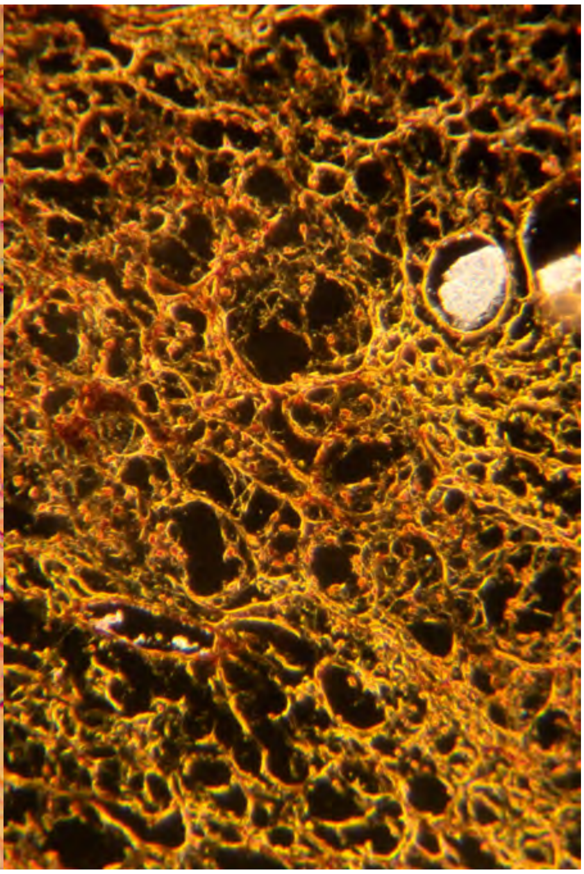

10:3 PRU_{rel} 1.7

Tumour HE 20x

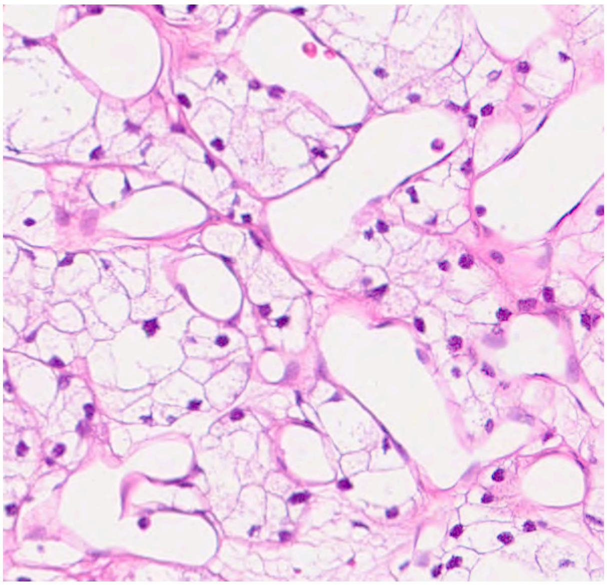

10:3 PRU_{rel}1.7

H12

Sex: Male
Age at surgery: 70 years
Survival from surgery: 3 years
Cause of death: Renal cancer
Initial stage: T5cm pT1a N1M1
Tumour type: Eosinophilic CCRCC ISUP grade 3
Tumour volume. 108 cm^3

Specimen weight: 320 g
Perfusion pressure: 15 mmHg Perfusate flow: 49 mL/min
Specimen PRU: 0.31
Cortical tissue PRU: 0.19 +/-….. n=6
Tumour tissue PRU span: NE n=30
Too few spheres in tumour cf autoradiogram
Fresh section: Bar 100 mm

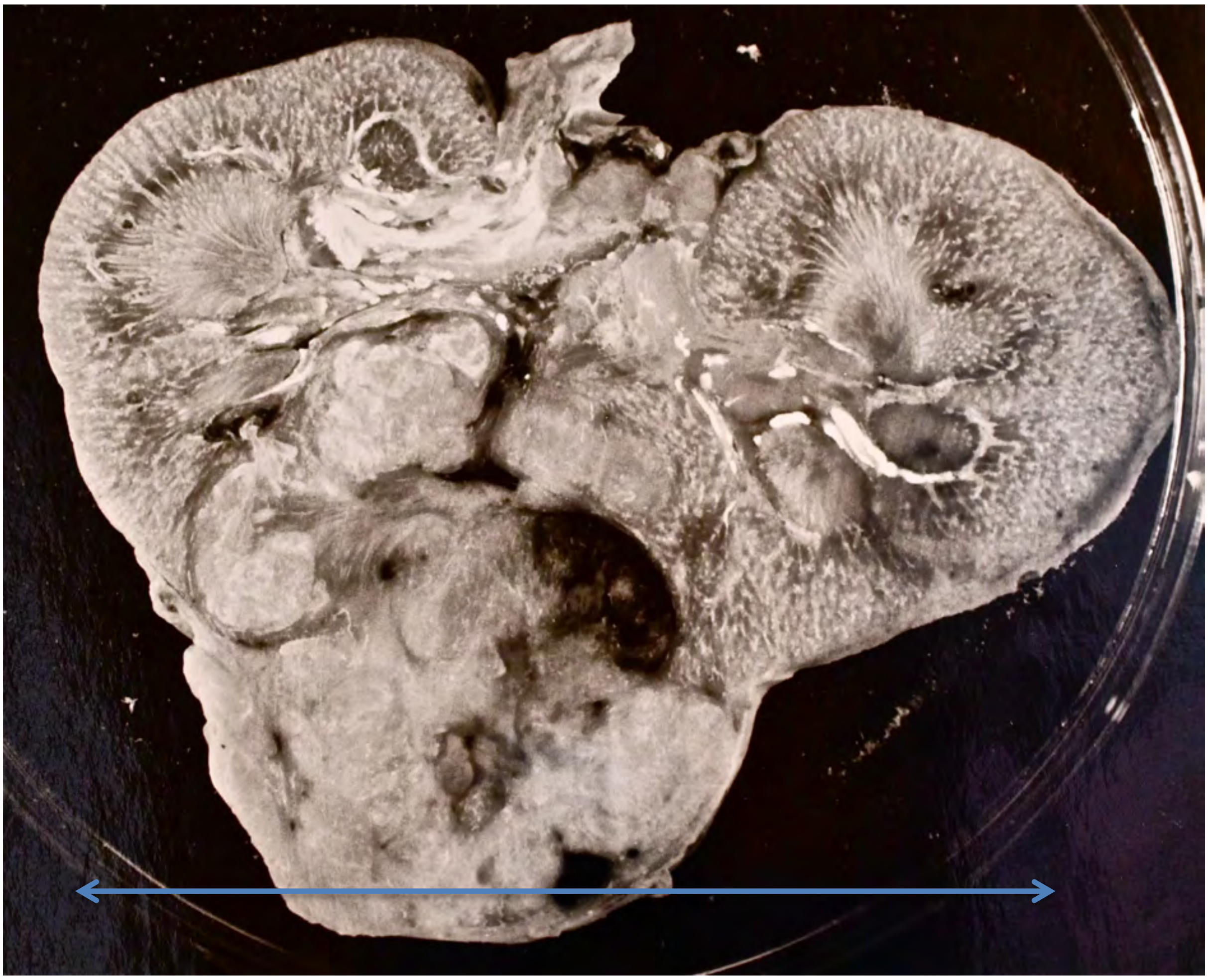

Angiography

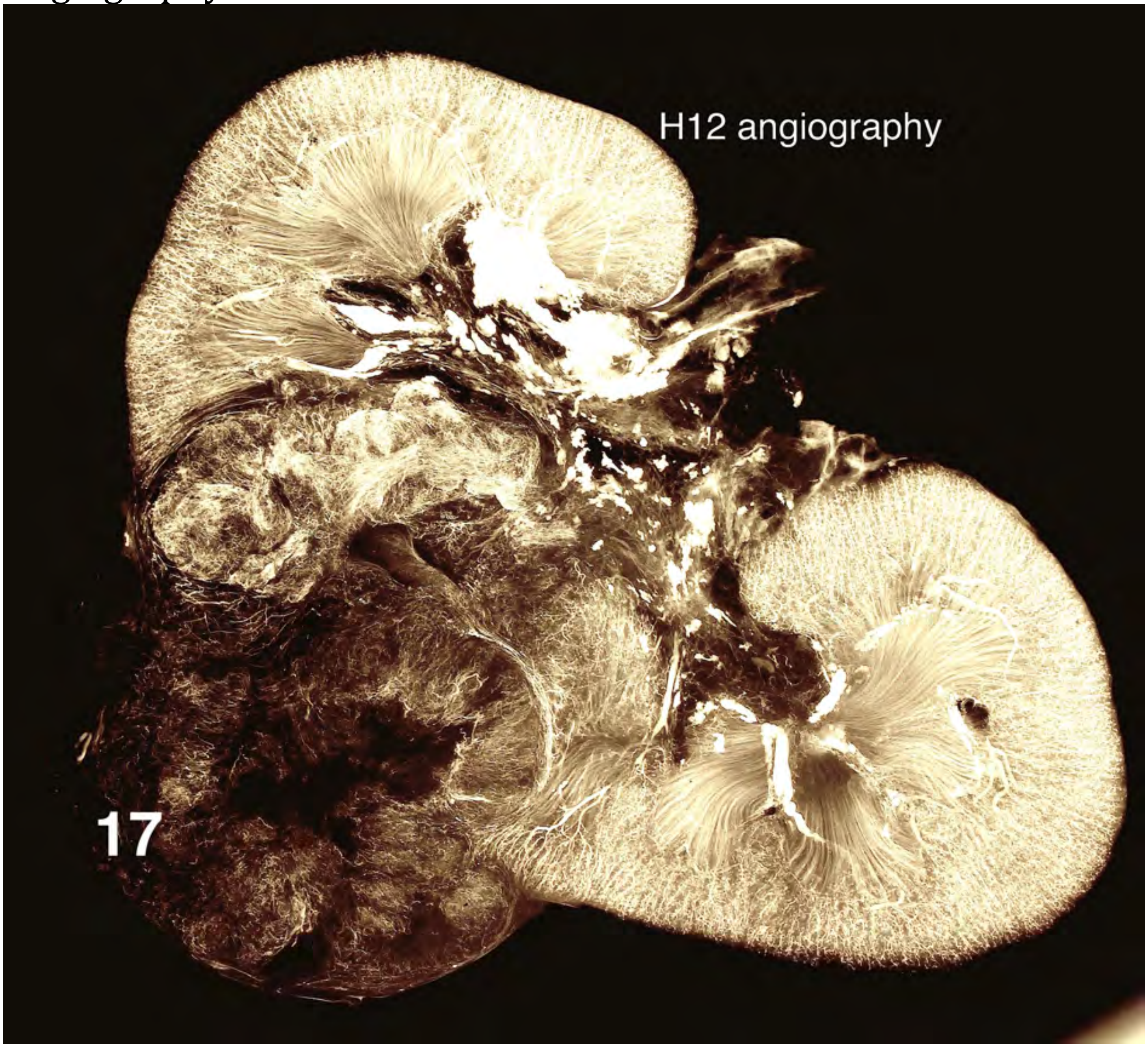

Autoradiography

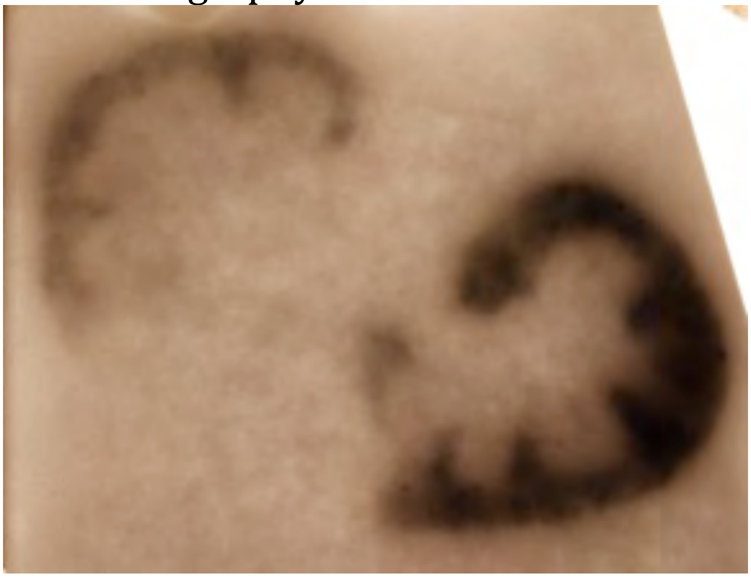

Darkfield macrophotography

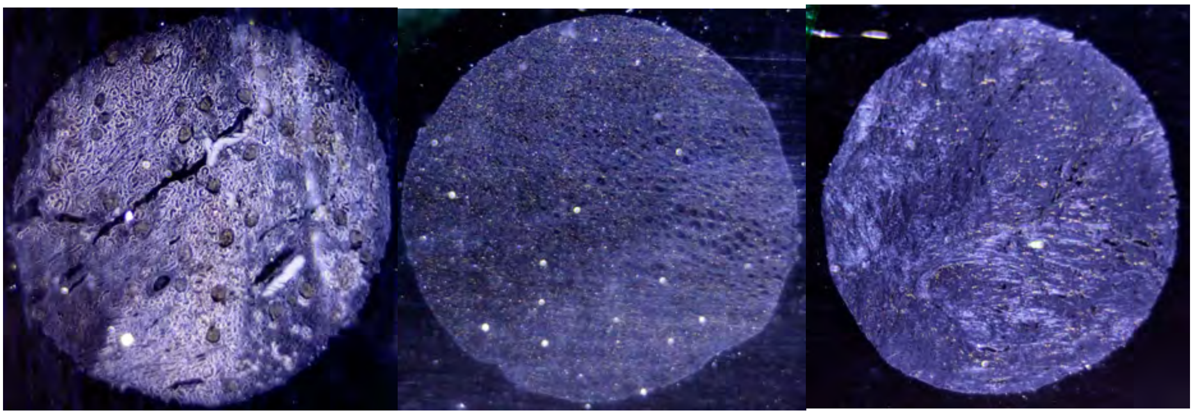

12:5 cortex PRU 0.19   12:23 medulla P NE   12:17 tumour P NE

Darkfield CD31

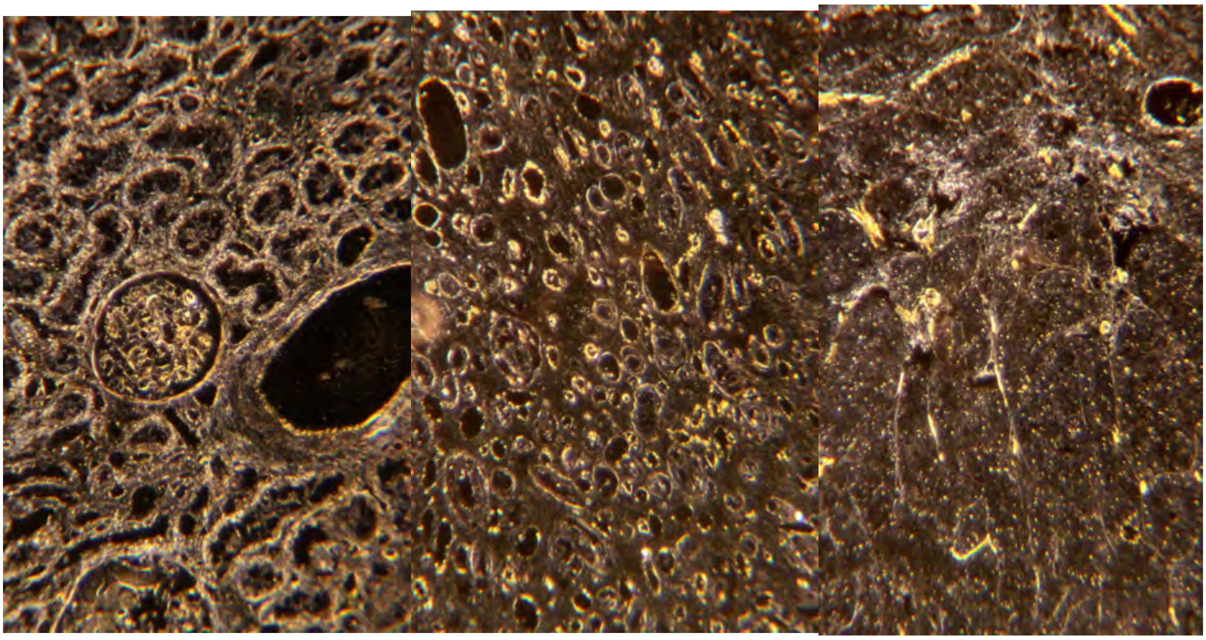

Cortex 12:5 PRU 0.19   Medulla 12:23 P NE   Tumour 12:17 P NE

HE 16x

Brightfield

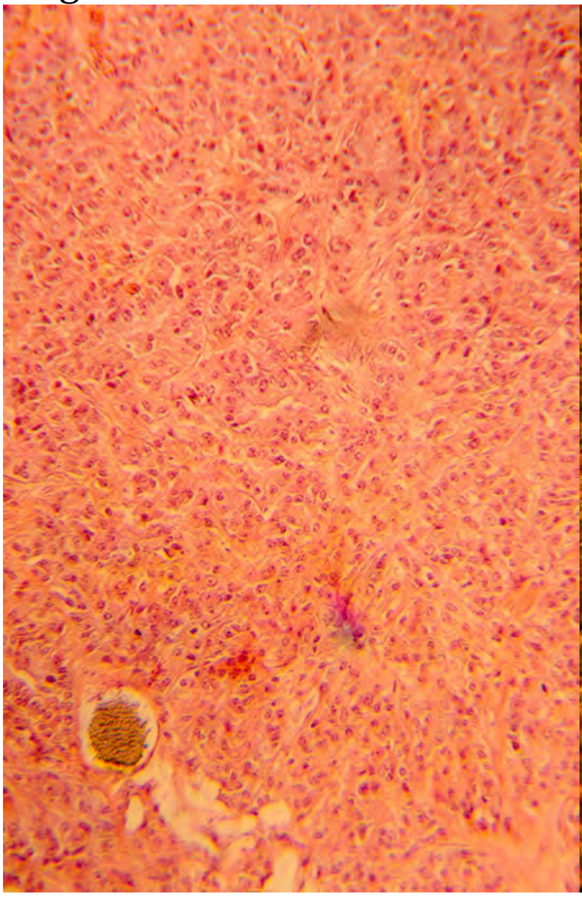

Darkfield

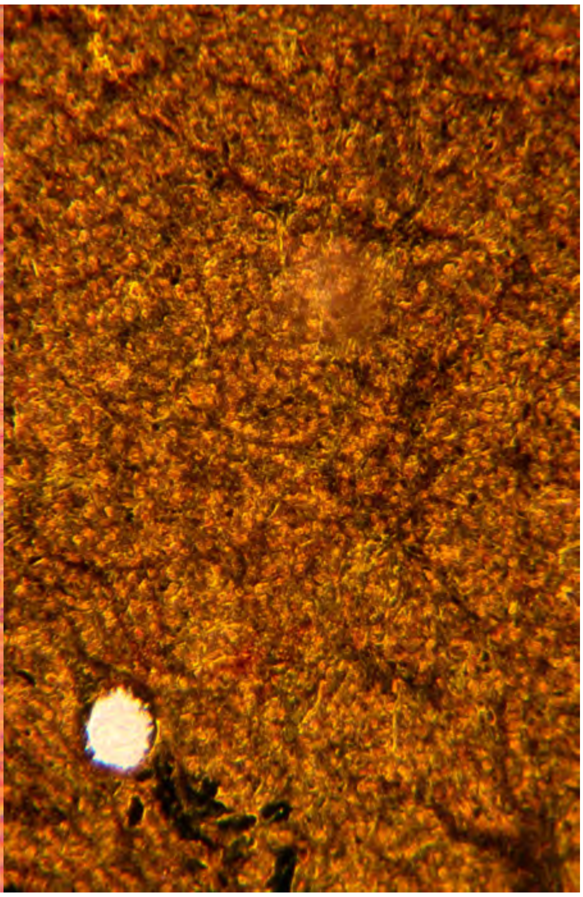

12:17 PRU NE

Tumour HE 20x

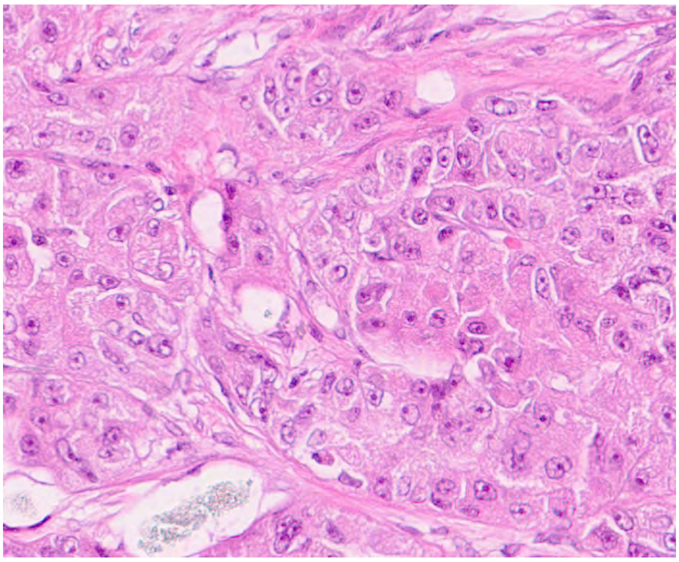

12:17 PRU NE

H13

Sex: Male
Age at surgery: 58 years
Survival from surgery: 7 years
Cause of death: Renal cancer
Initial stage: T9x7 cm pT2 N1
Tumour type: Eosinophilic CCRCC ISUP grade 3
Tumour volume: 94 cm^3

Specimen weight: 540
Perfusion pressure: 30 mmHg Perfusate flow: 42 mL/min
Specimen PRU: 0.71
Cortical tissue PRU: 0.17 n=5
Tumour tissue PRU span: NE n=23

Fresh section: Bar 100 mm

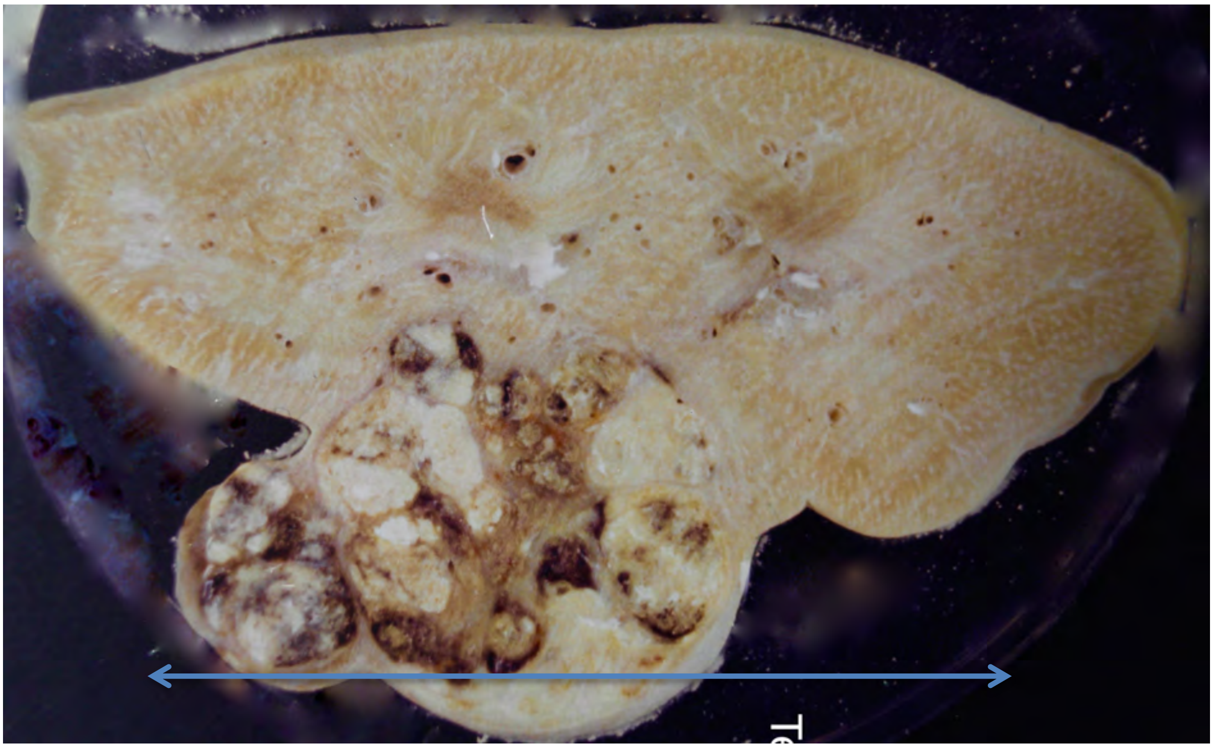

Angiography

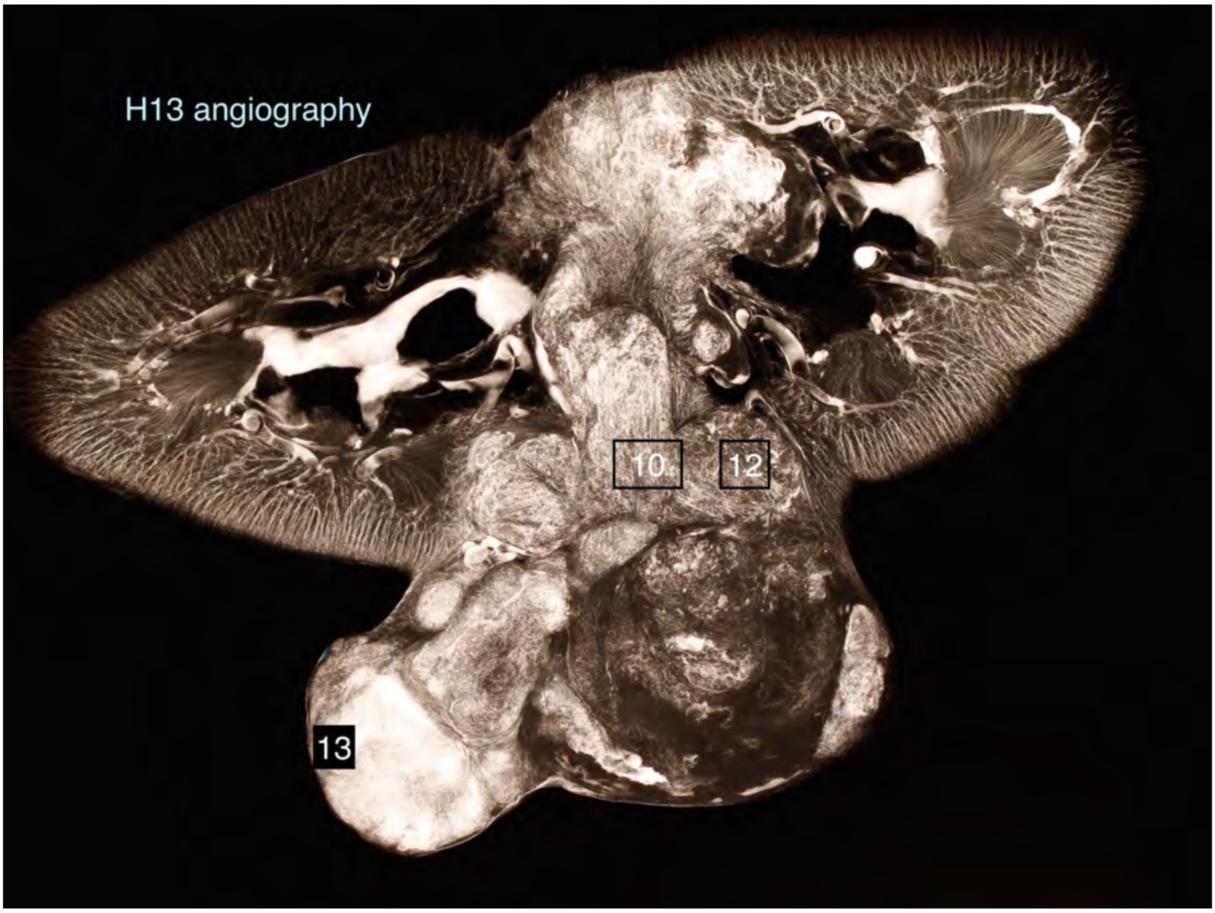

Autoradiography

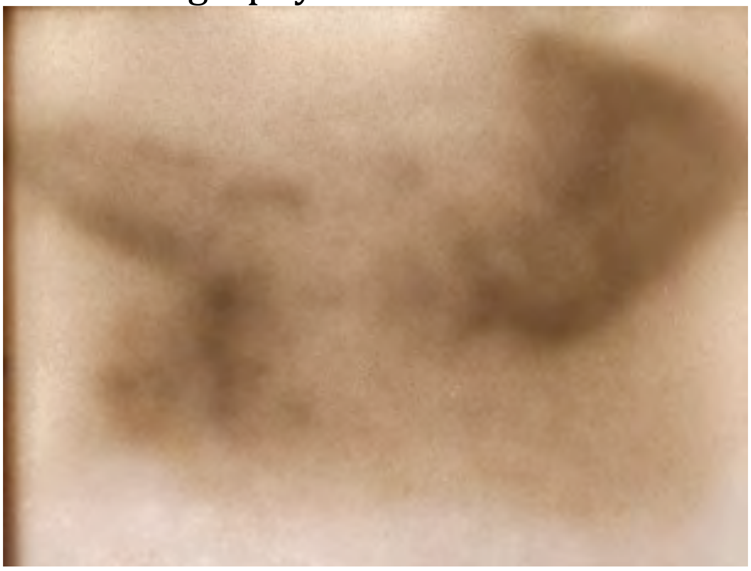

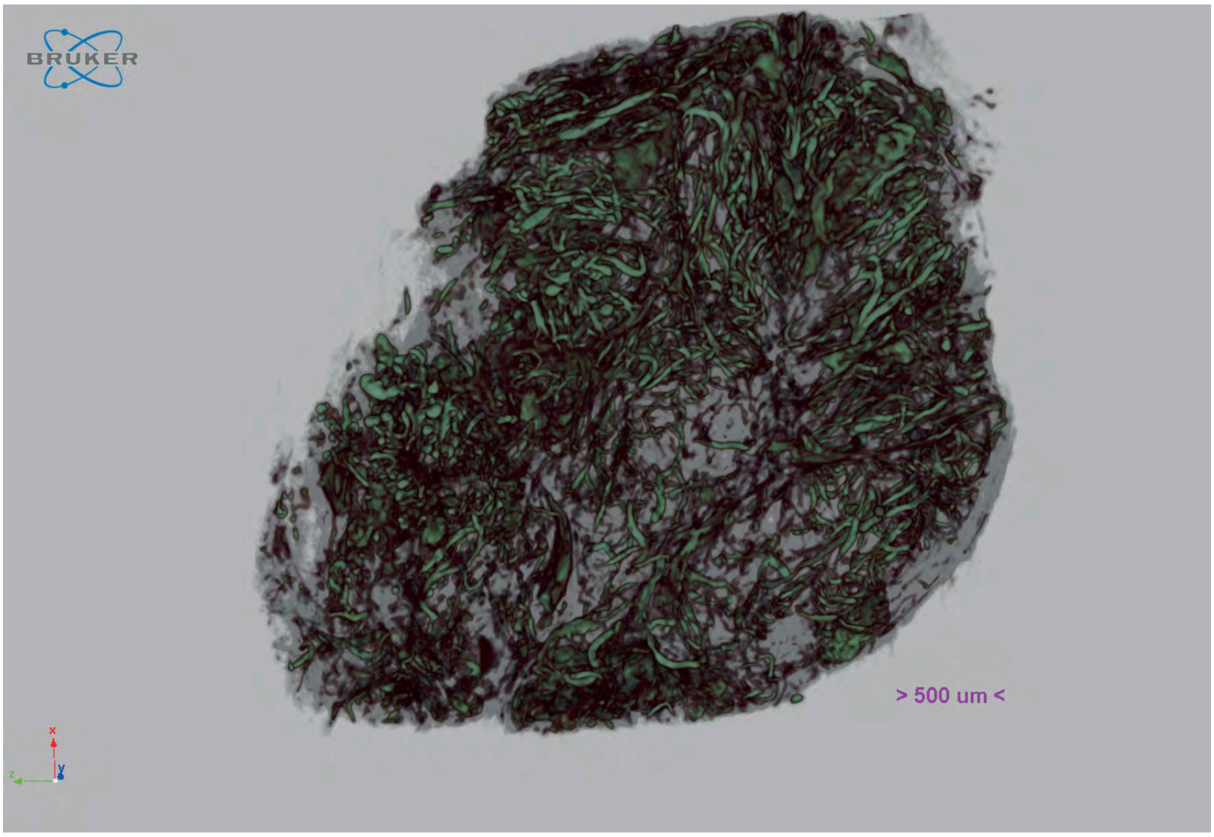

MicroCT Tumour H13:12

Darkfield macrophotography

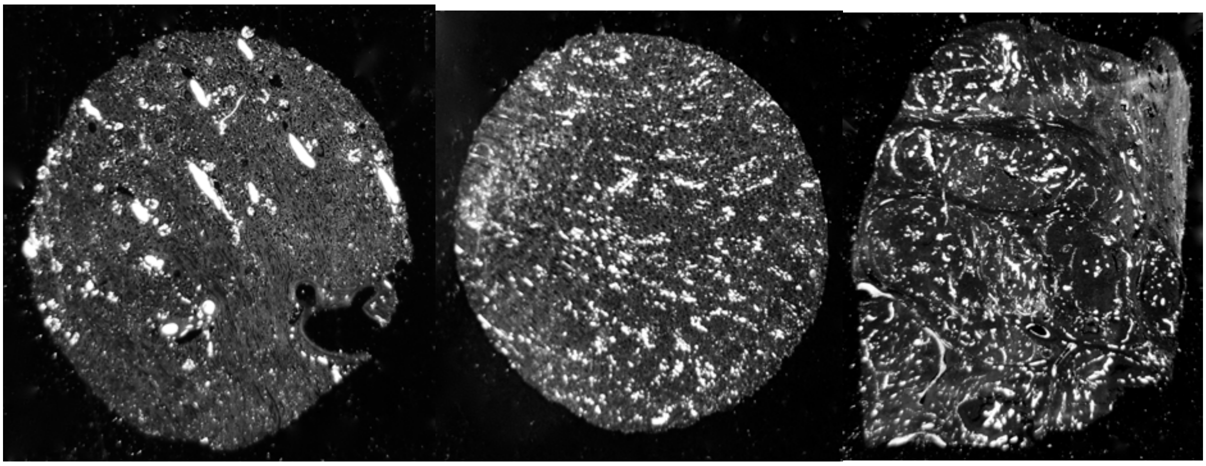

Cortex 13:32 PRU 0.32 Medulla 13:2 PRU 2.36 Tumour 13:12 PRU 0.76

Darkfield CD31

Contrast

CD31

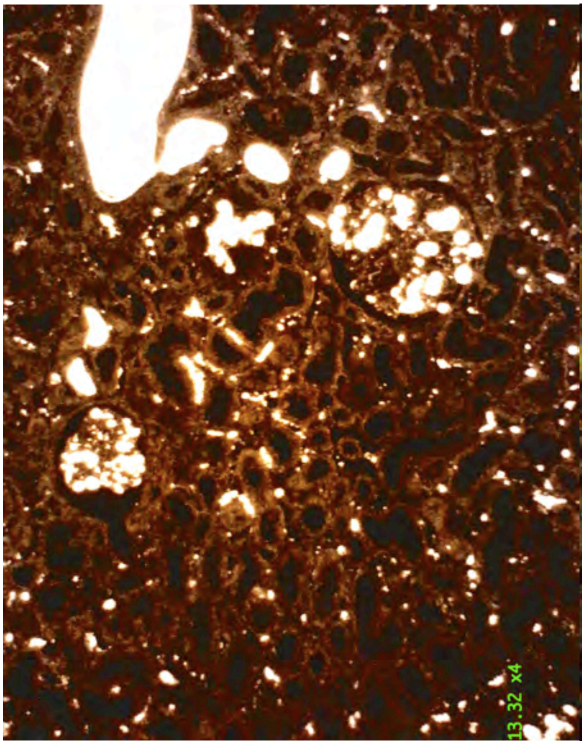

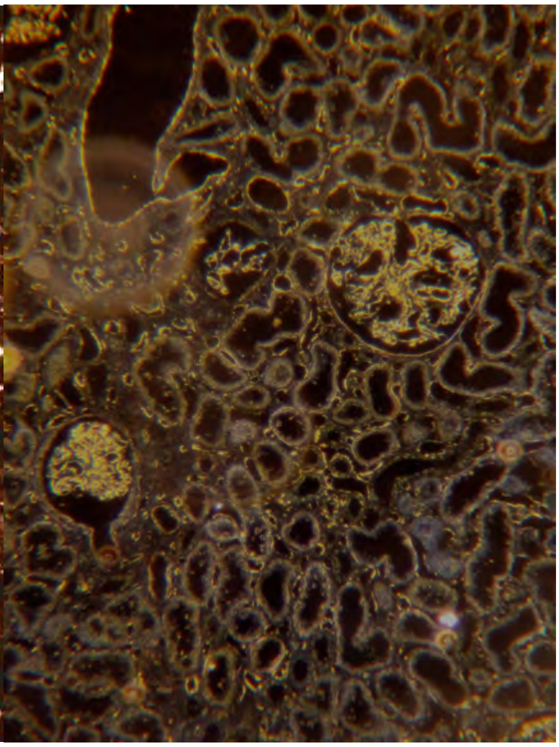

Cortex 13:32 PRU 0.32

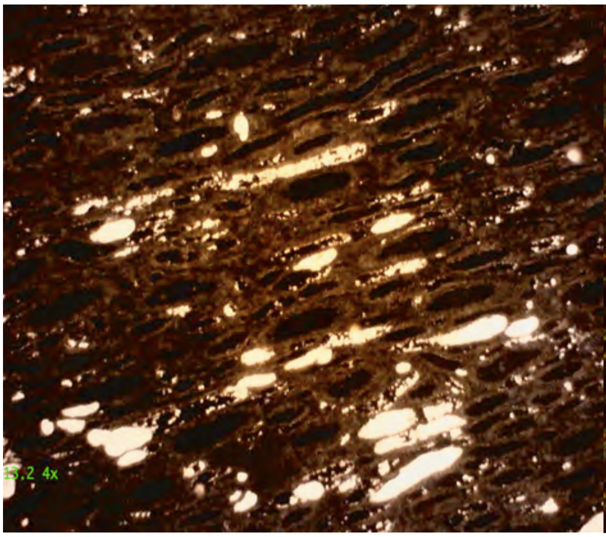

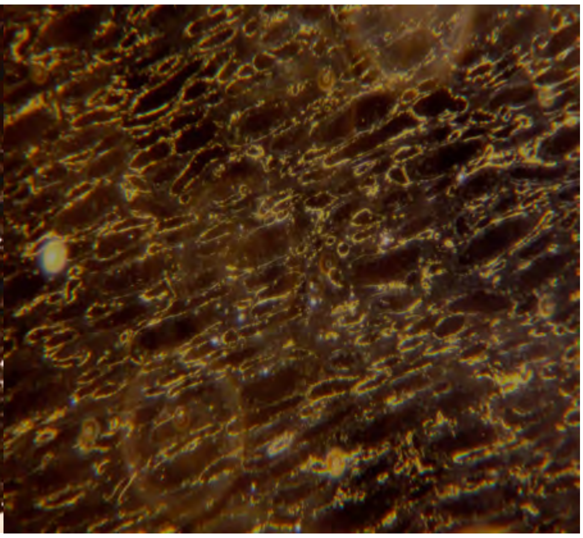

Medulla 13:2 PRU 2.36

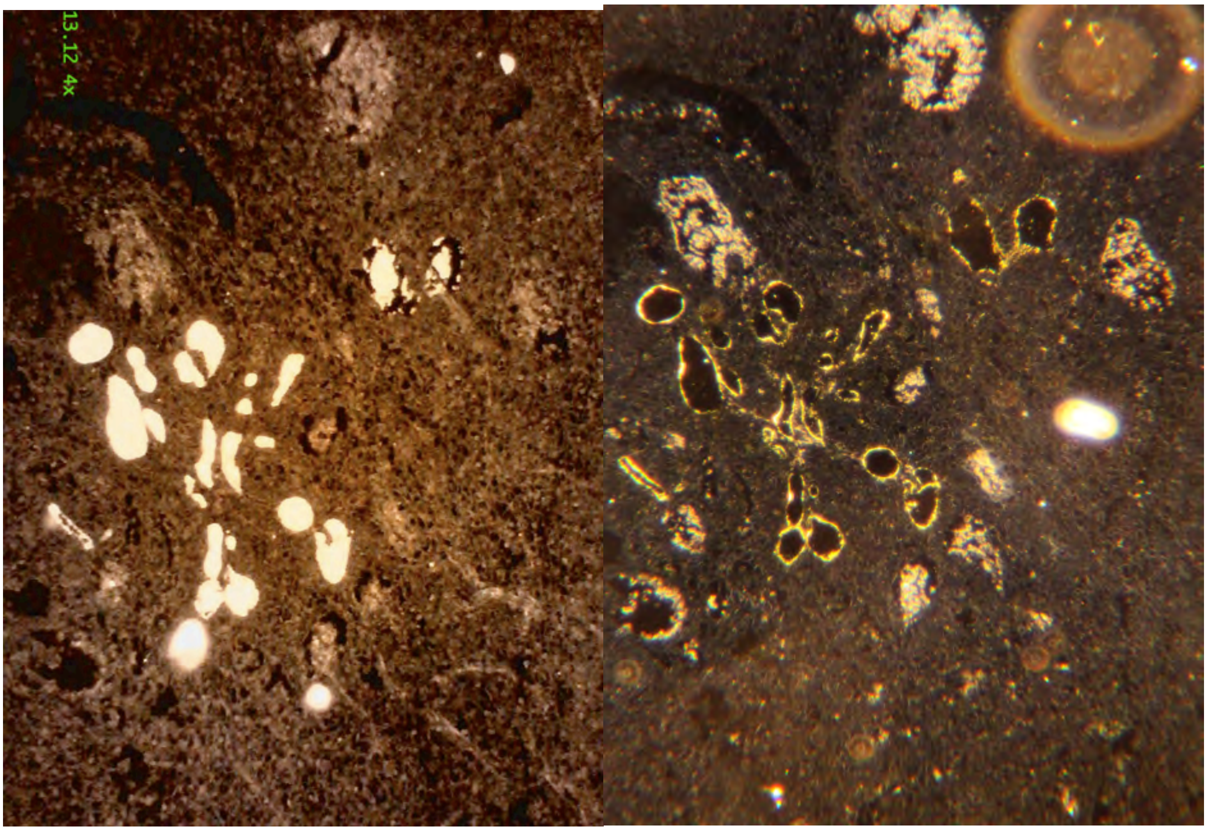

Tumour 13:12 PRU 0.76

HE-stained tumour sample

Bright field Darkfield

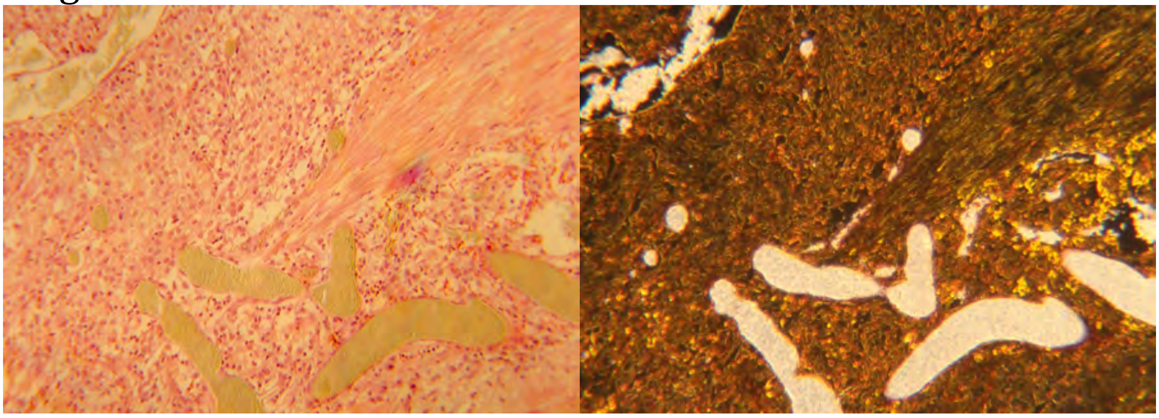

13:12 PRU 0.76

HE 20x

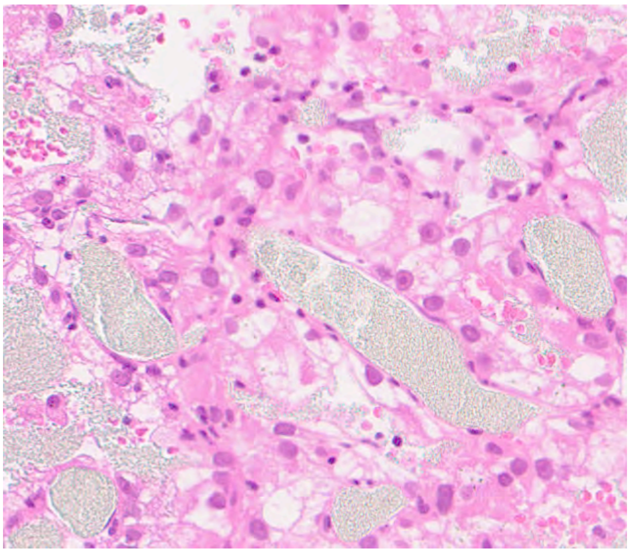

13:12 20x PRU 0.76

H14

Sex: Female
Age at surgery: 80 years
Survival from surgery: 0 year
Cause of death: Renal cancer
Initial stage: TxN1
Tumour type: spolcellig Oncocytoma ISUP grade NE
Tumour volume: 480 cm^3

Specimen weight: 619 g
Perfusion pressure: 46 mmHg Perfusate flow: 29 mL/min
Specimen PRU: 1.59
Cortical tissue PRU: 0.18+/-0.08 n=8
Tumour tissue PRU span: 1.09-170 n=22

Fresh section: Bar 100 mm

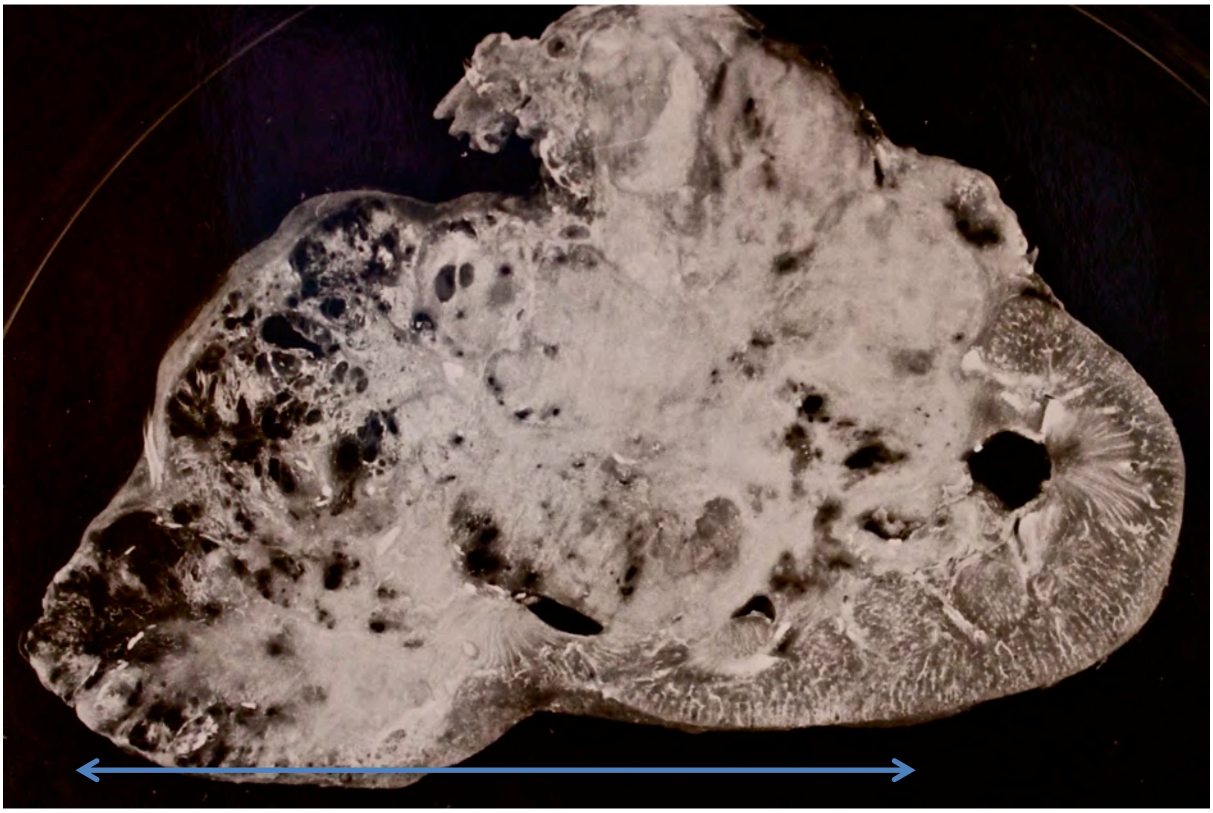

Angiography

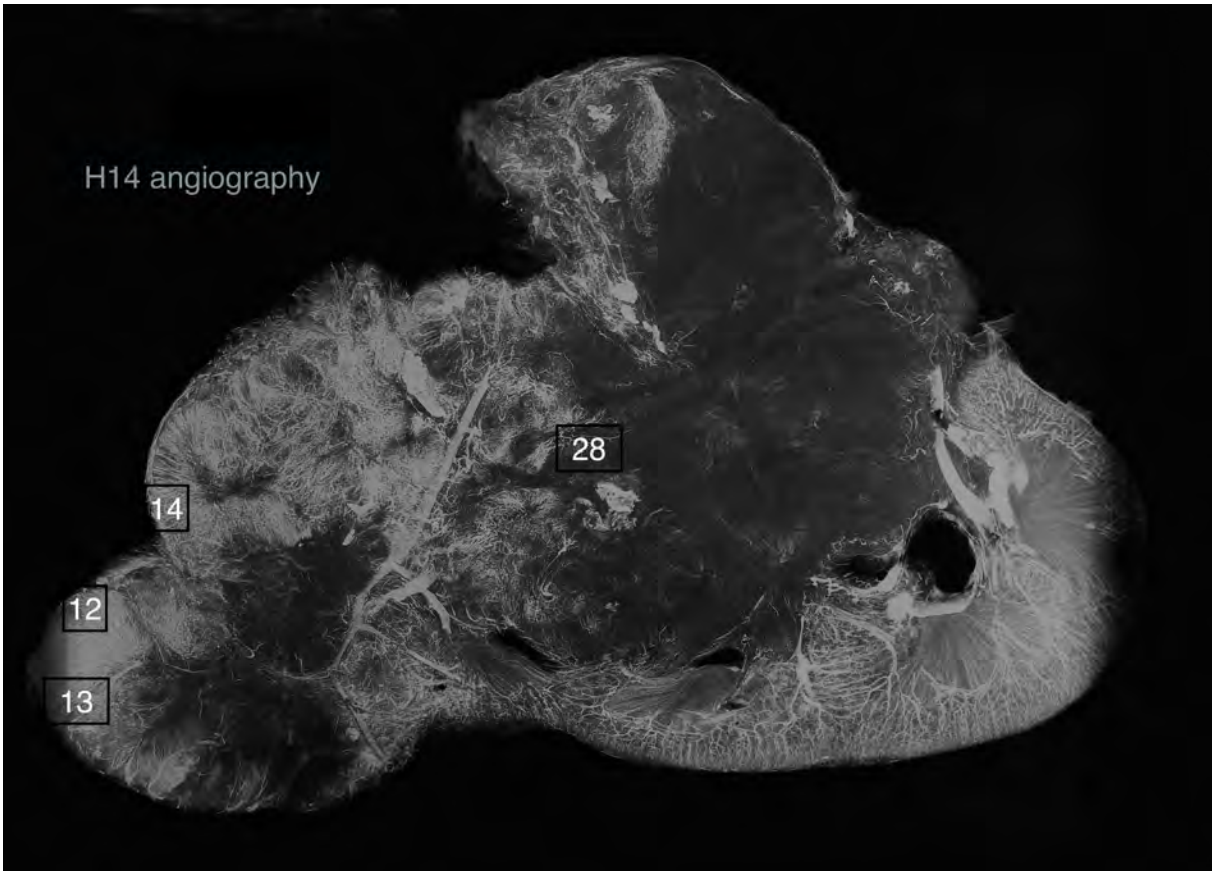

Heterogenous vascularity and sphere trapping

Autoradiography

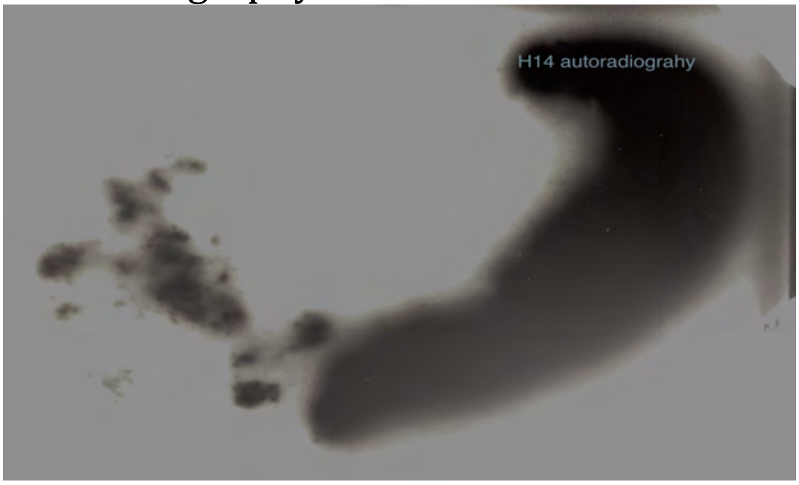

Micro-CT

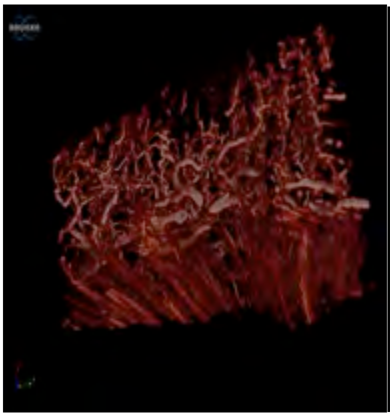

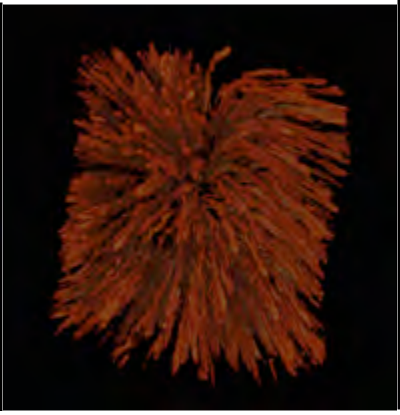

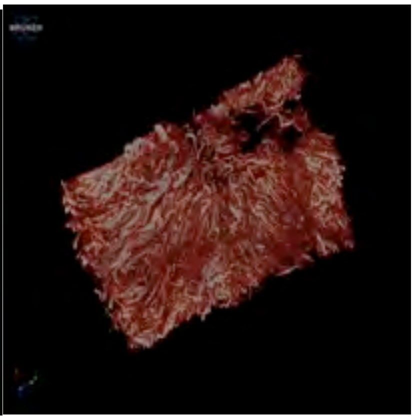

Cortico-medullary   Medulla towards papilla   Tumour

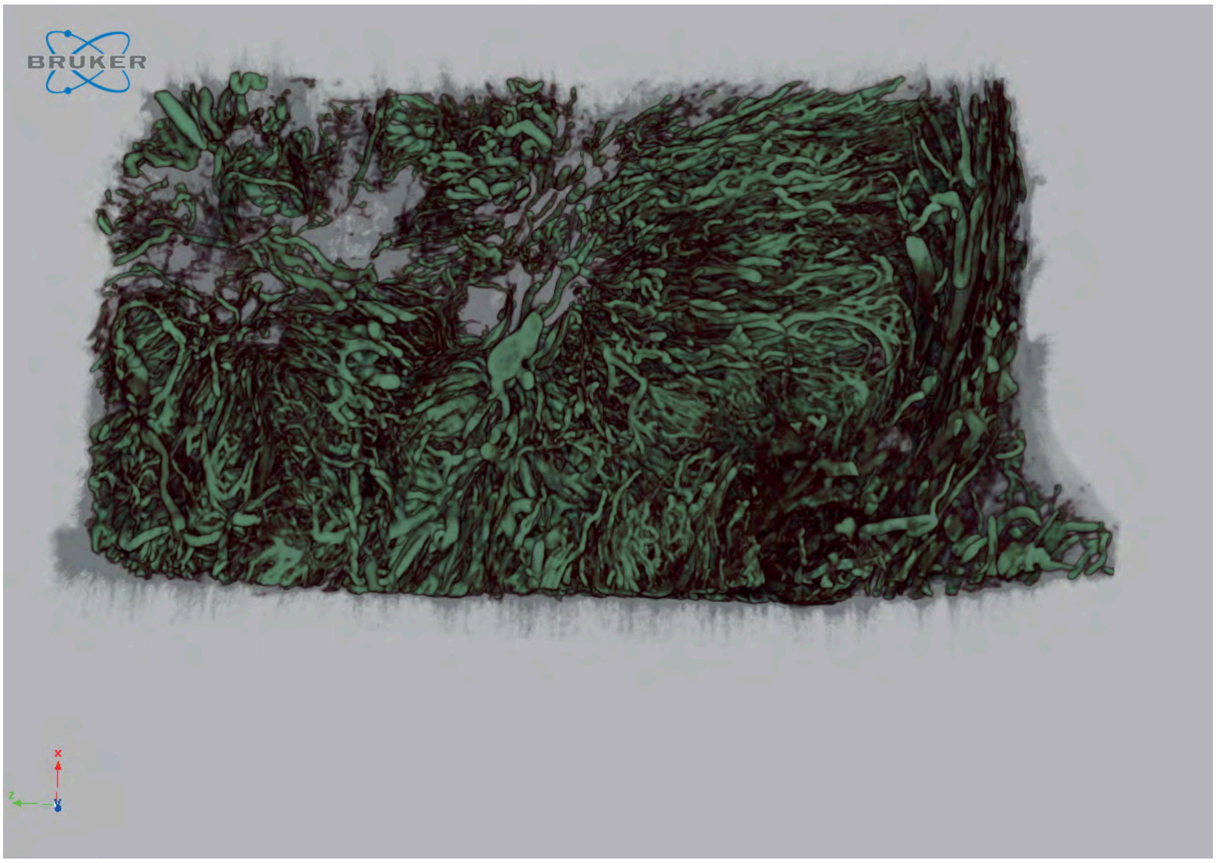

MicroCT Tumour H14:14

Dark-field macrophotography

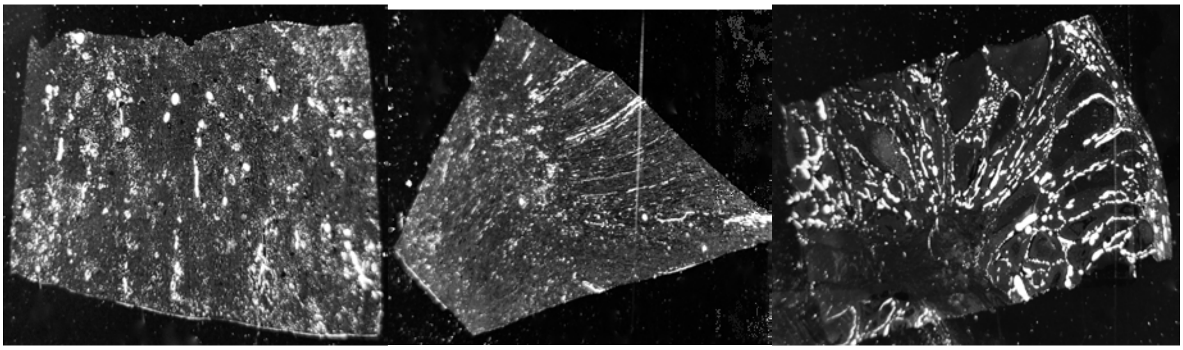

Cortex 14:1PRU 0.15 Medulla 14:10 P 0.54 Tumour 14:14 P 2.49

Darkfield
Contrast CD31

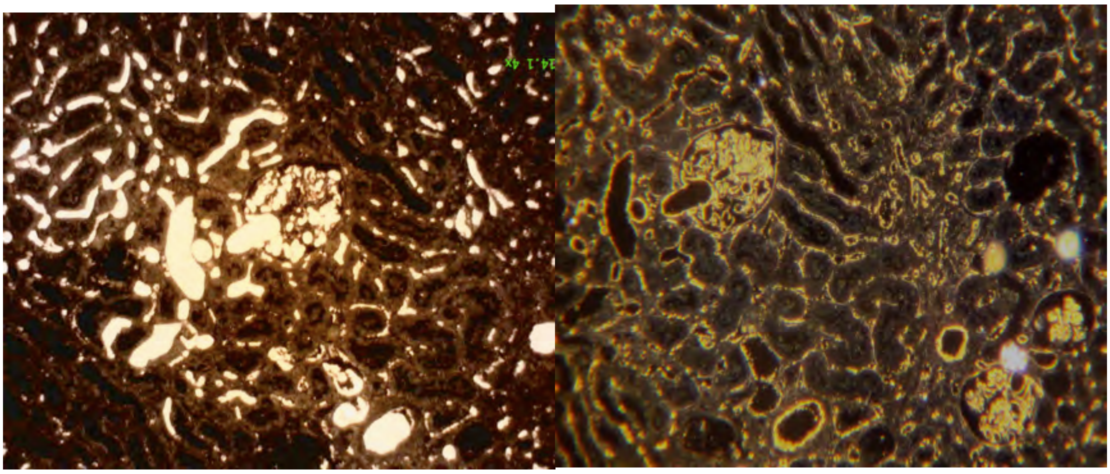

Cortex 14:1 PRU 0.15

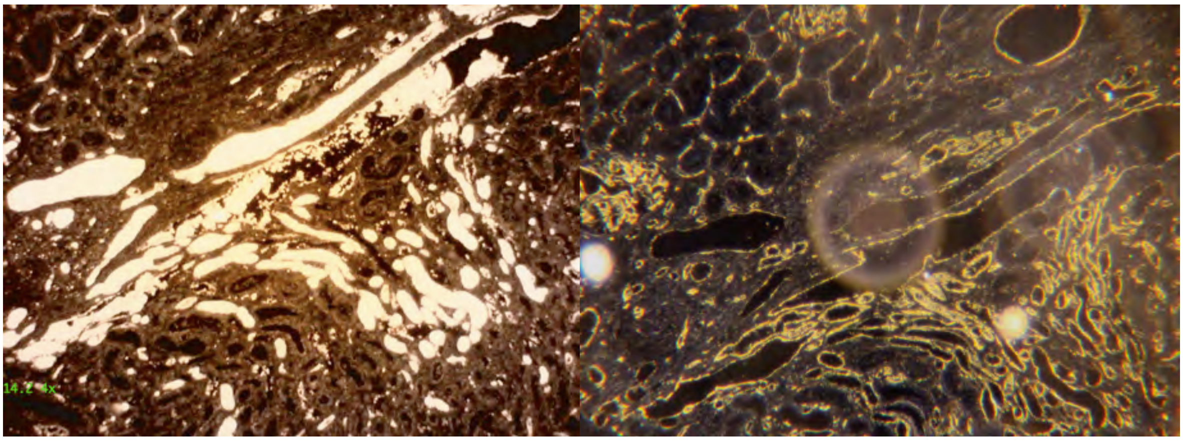

Medulla 14:10 PRU 0.54

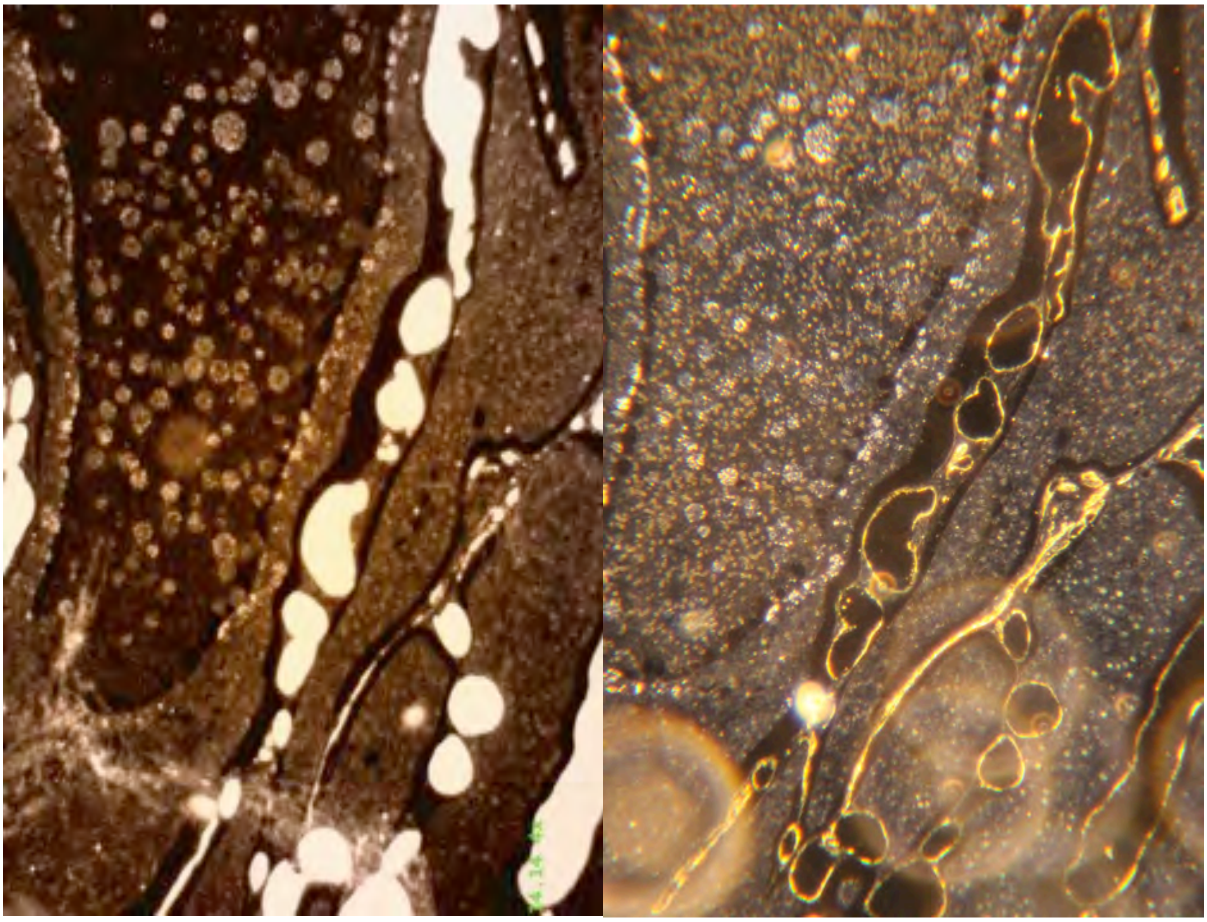

Tumour 14:14 PRU 2.49

Tumour sample HE-stained

Bright field

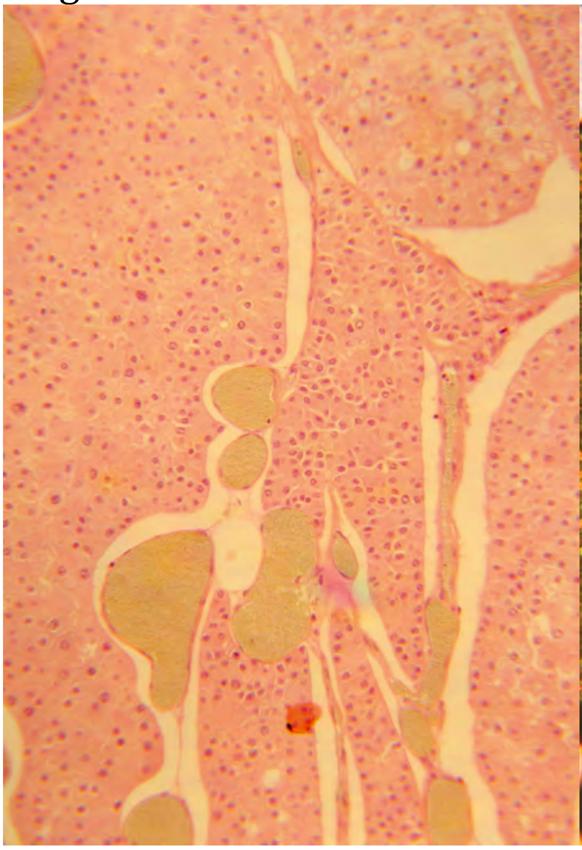

Darkfield

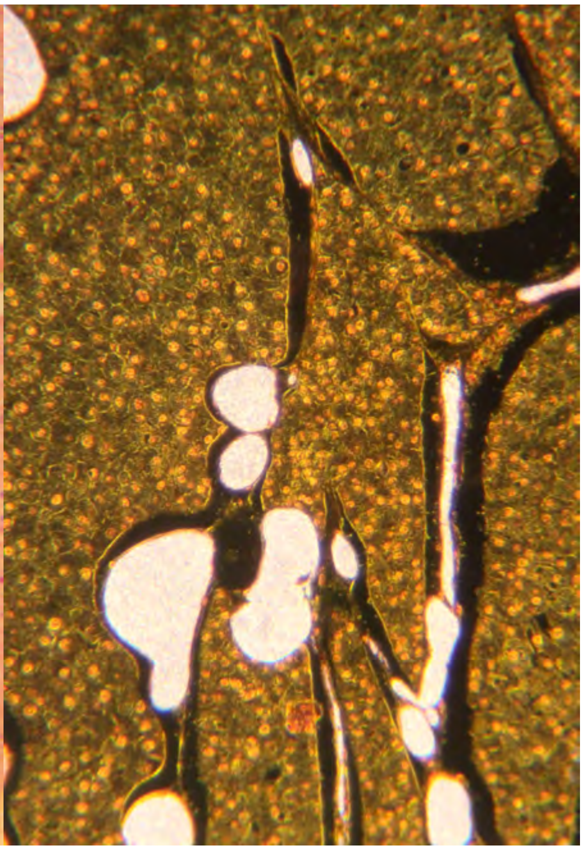

14:14 PRU 2.49

HE 20x

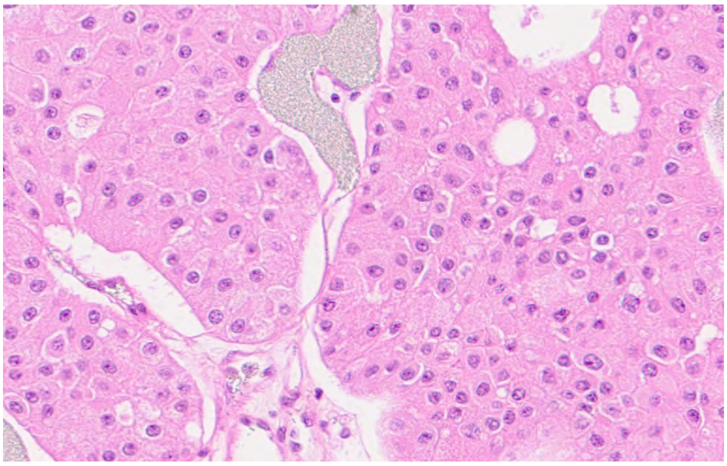

14:14

H15

Sex: Male
Age at surgery: 72 years
Survival from surgery: 0 year
Cause of death: Renal cancer? Mors subita cardiac event
Initial stage: 6 cm pT1b Nx M1?
Tumour type: clear-intermed-plasmic CCRCC   ISUP grade 2
Tumour volume: 210 cm^3

Specimen weight: 417 g
Perfusion pressure: 45 mmHg   Perfusate flow: 45 mL/min
Specimen PRU: 1.00
Cortical tissue PRU: 0.20 +/-0.04   n=6
Tumour tissue PRU span: 0.75-450   n=22

Fresh section: Bar 100 mm

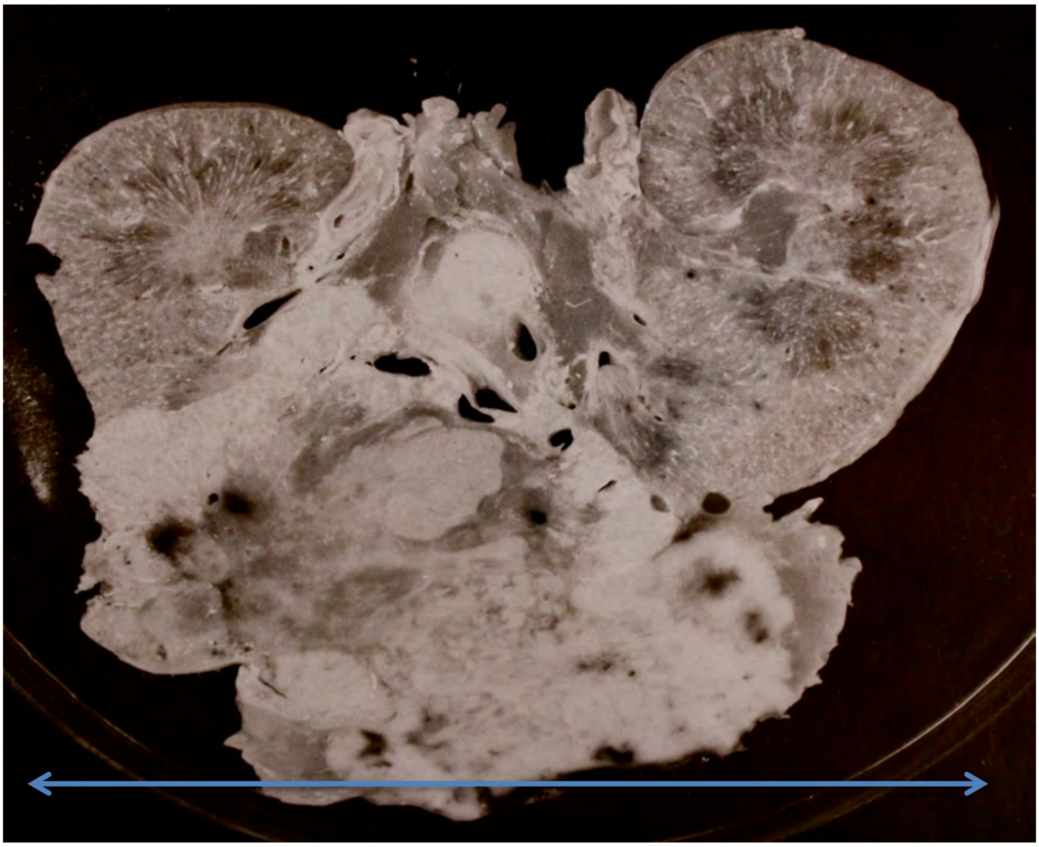

Angiography

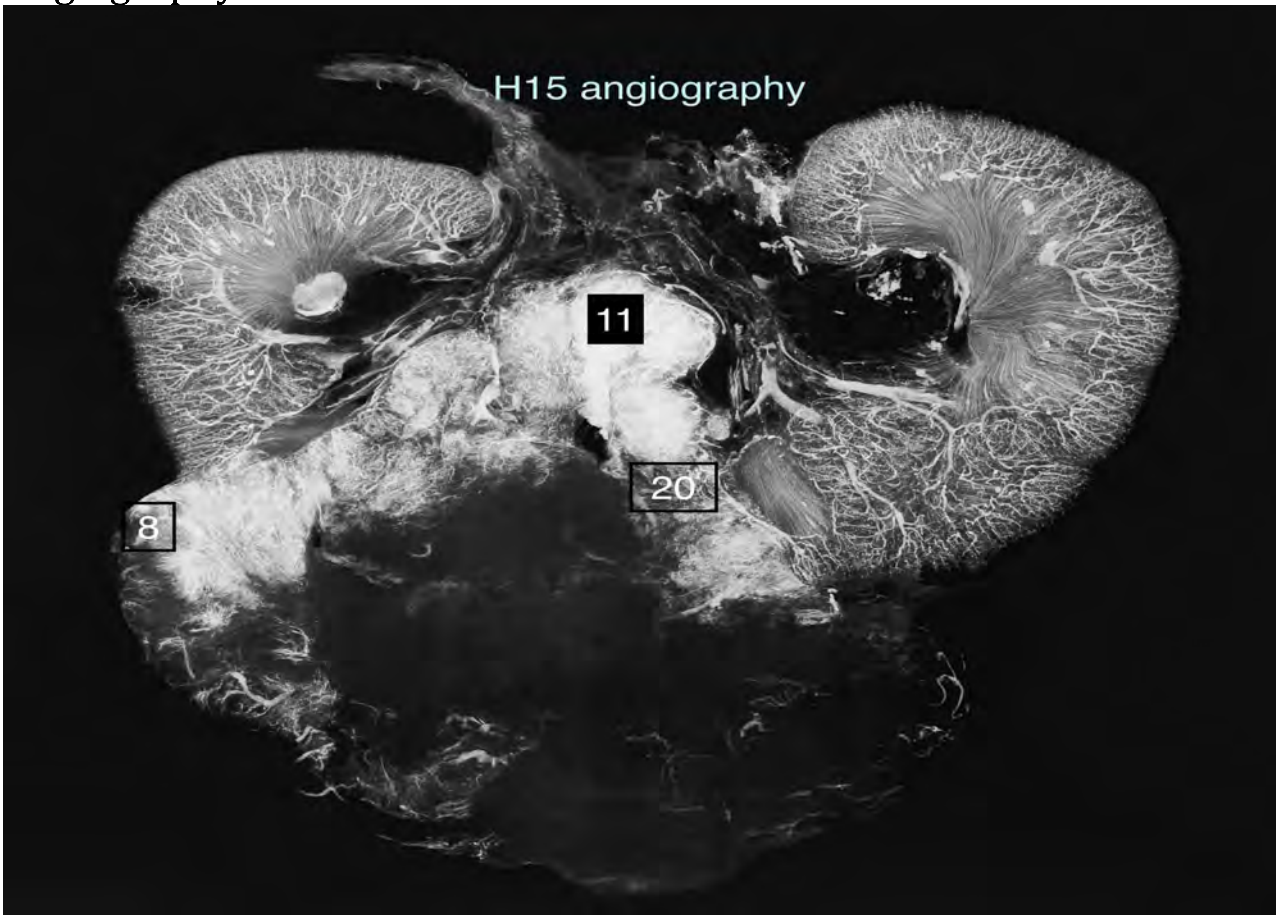

Heterogenous vascularity and 15 um sphere trapping

Autoradiography

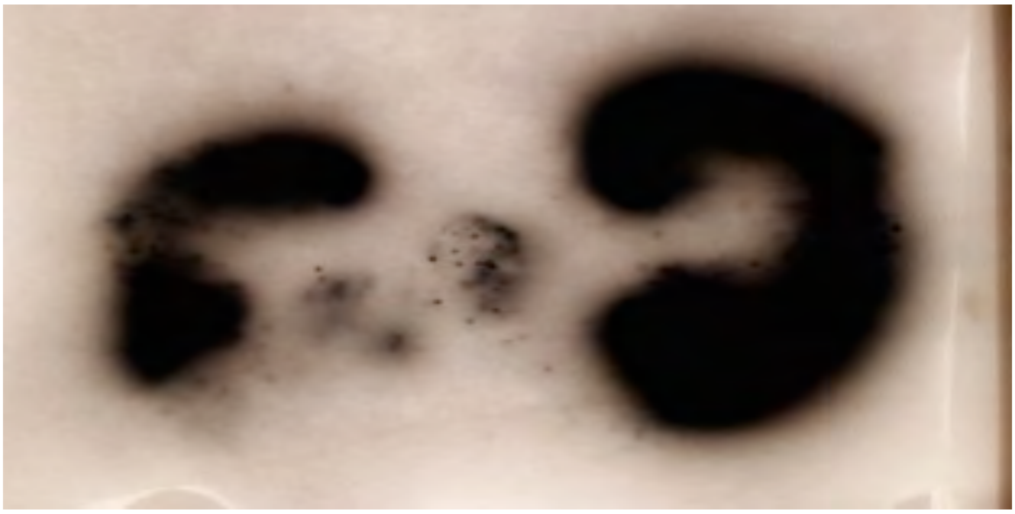

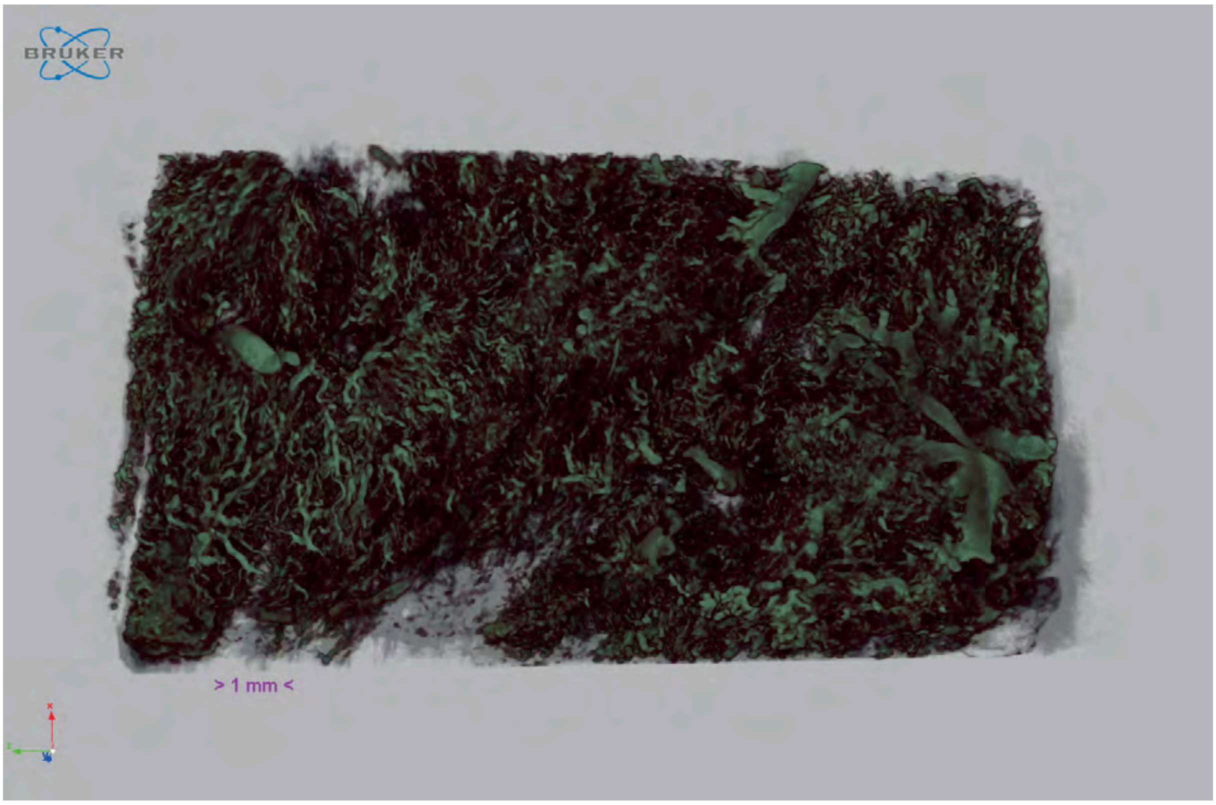

MicroCT Tumour H15:11

Dark-field macrophotography

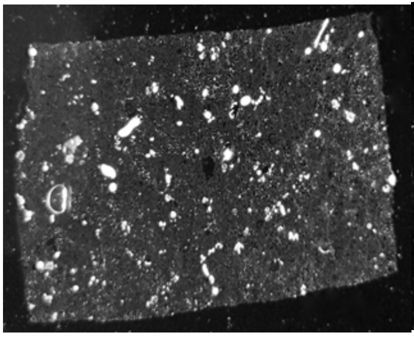
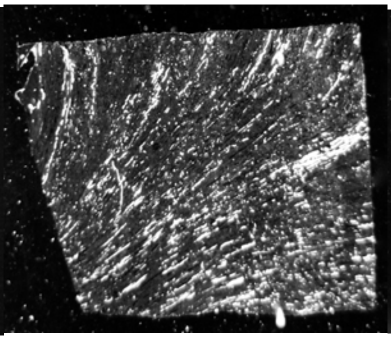
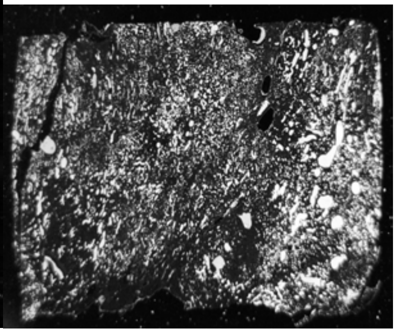

Cortex 15:3 P 0.16 Medulla 15:7 P 0.54 Tumour 15:11 P 0.75

Darkfield

Contrast CD31

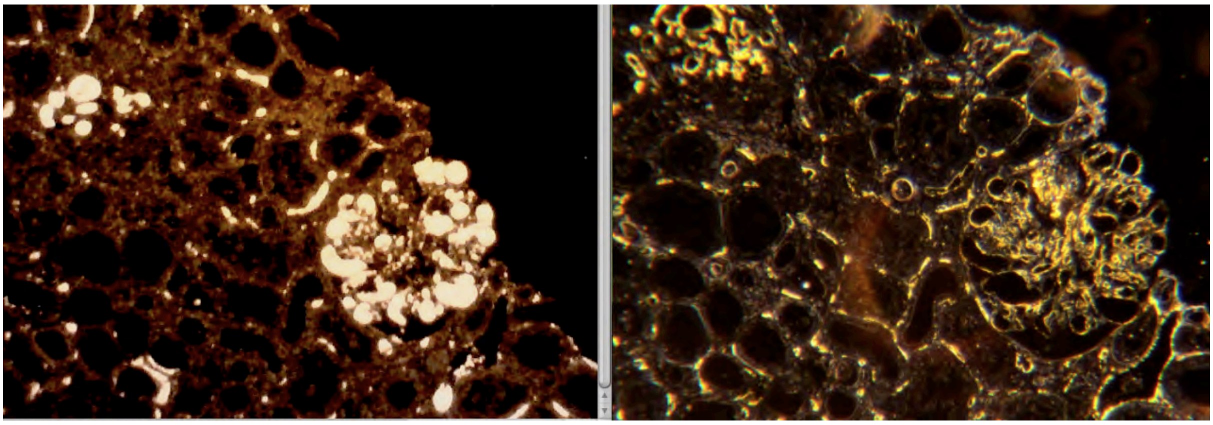

Cortex 15:3 PRU 0.16

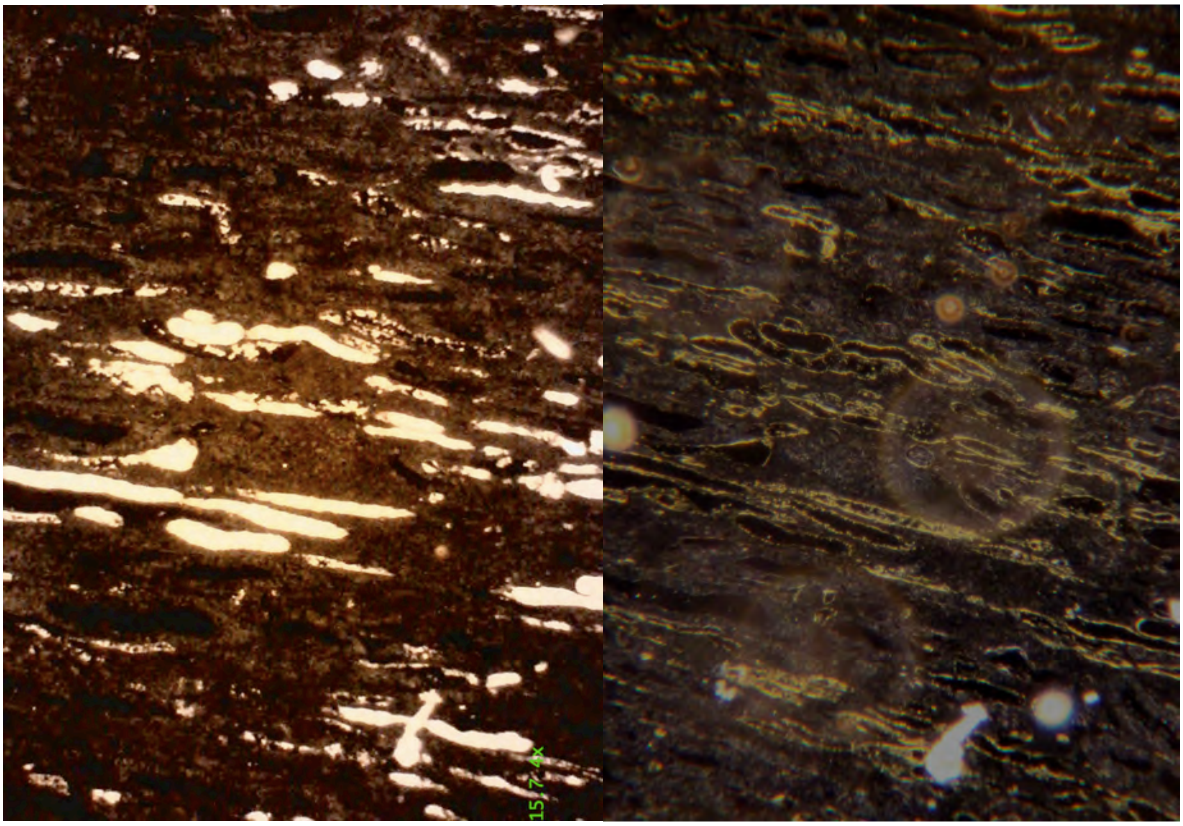

Medulla 15:7 PRU 0.54

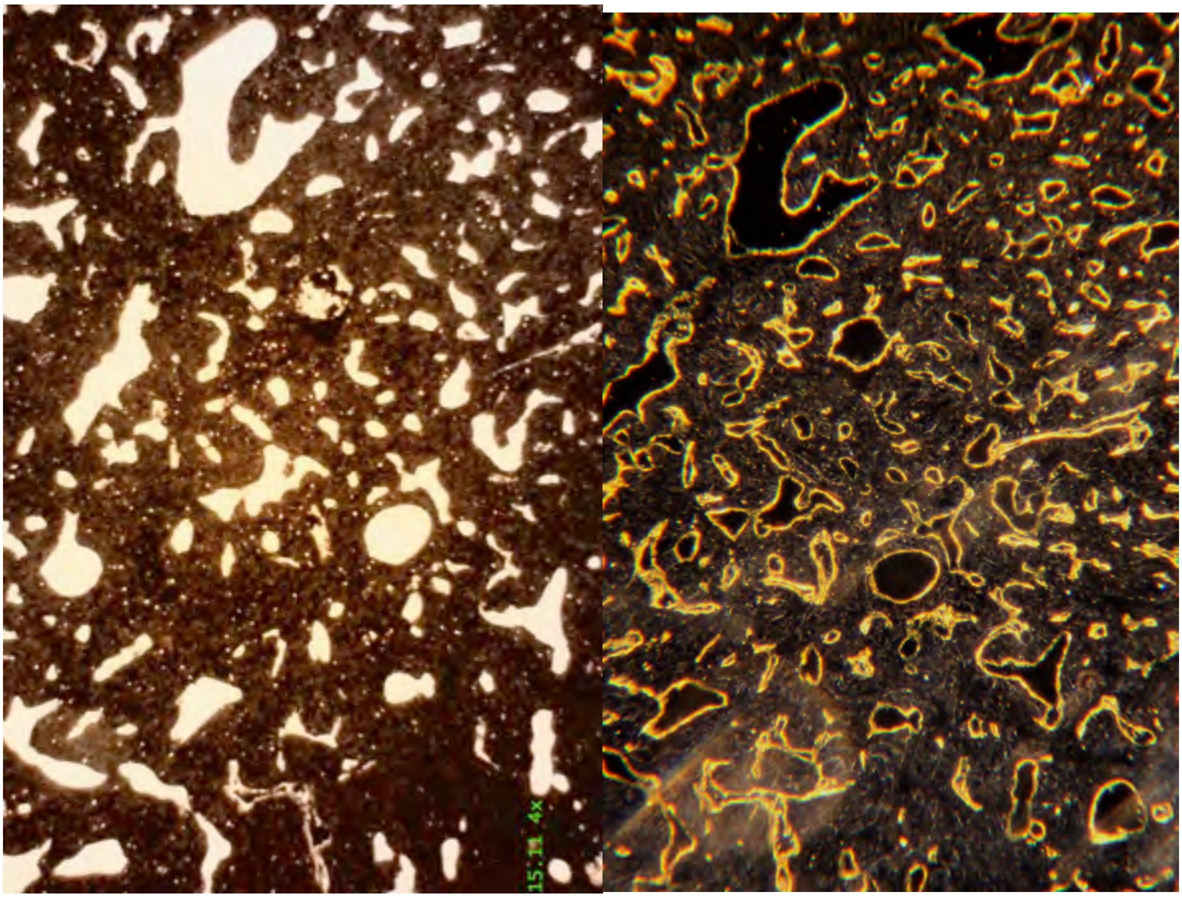

Tumour 15:11 PRU 0.75

HE 20x

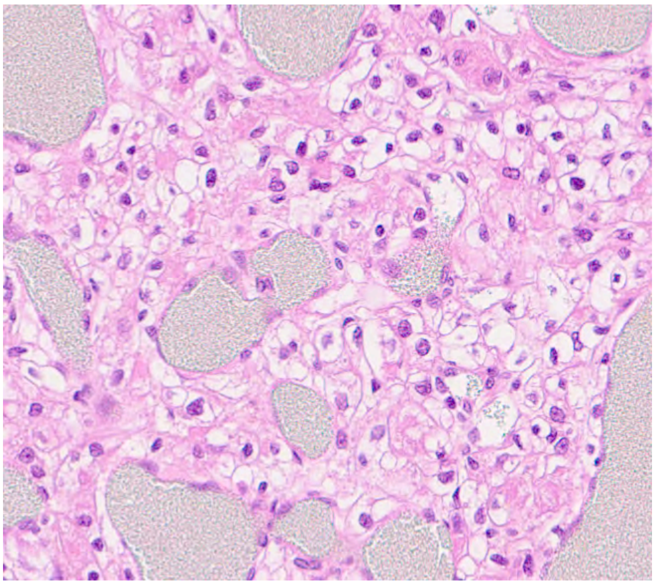

15.11 20x PRU 0.75

Tumour samples HE stained

Bright field Darkfield

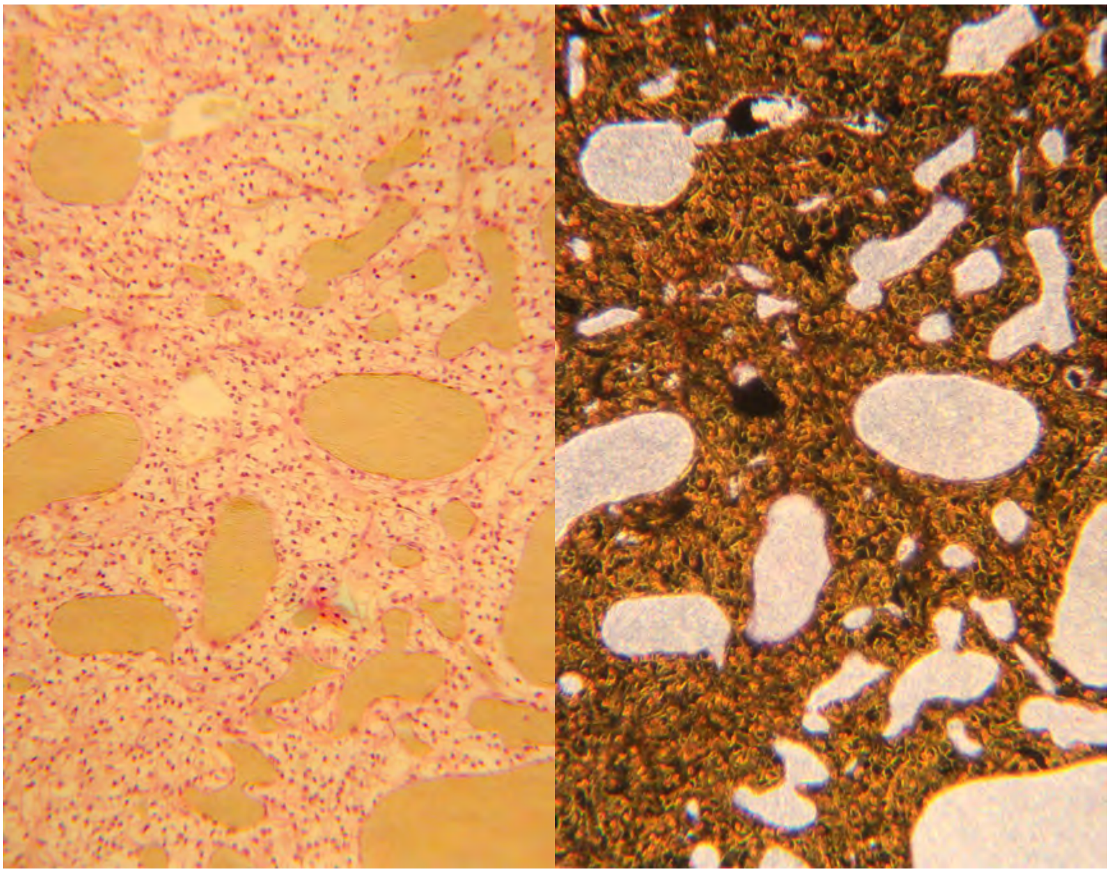

15-11 PRU 0.75

H16

Sex: Male
Age at surgery: 65 years
Survival from surgery: 2 years
Cause of death: Renal cancer
Initial stage: TxNxM1
Tumour type: CCRCC polymorph ISUP grade: 1-(2) ?
Tumour volume: 168 cm^3

Specimen weight: 496 g
Perfusion pressure: 40 mmHg Perfusate flow: 60 mL/min
Specimen PRU: 0.67
Cortical tissue PRU: 0.12+/-0.01 n=5
Tumour tissue PRU span: 0.51-10.5 n=12

Fresh section: Bar 100 mm

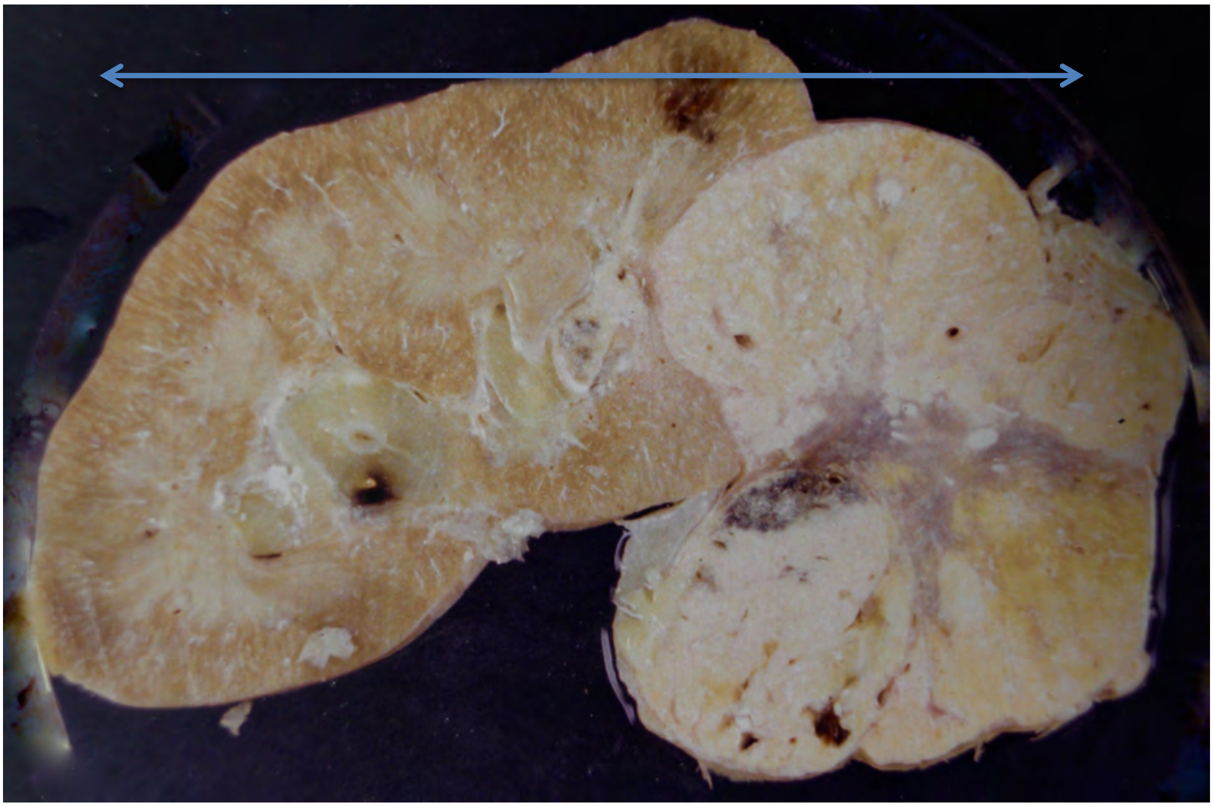

Angiography

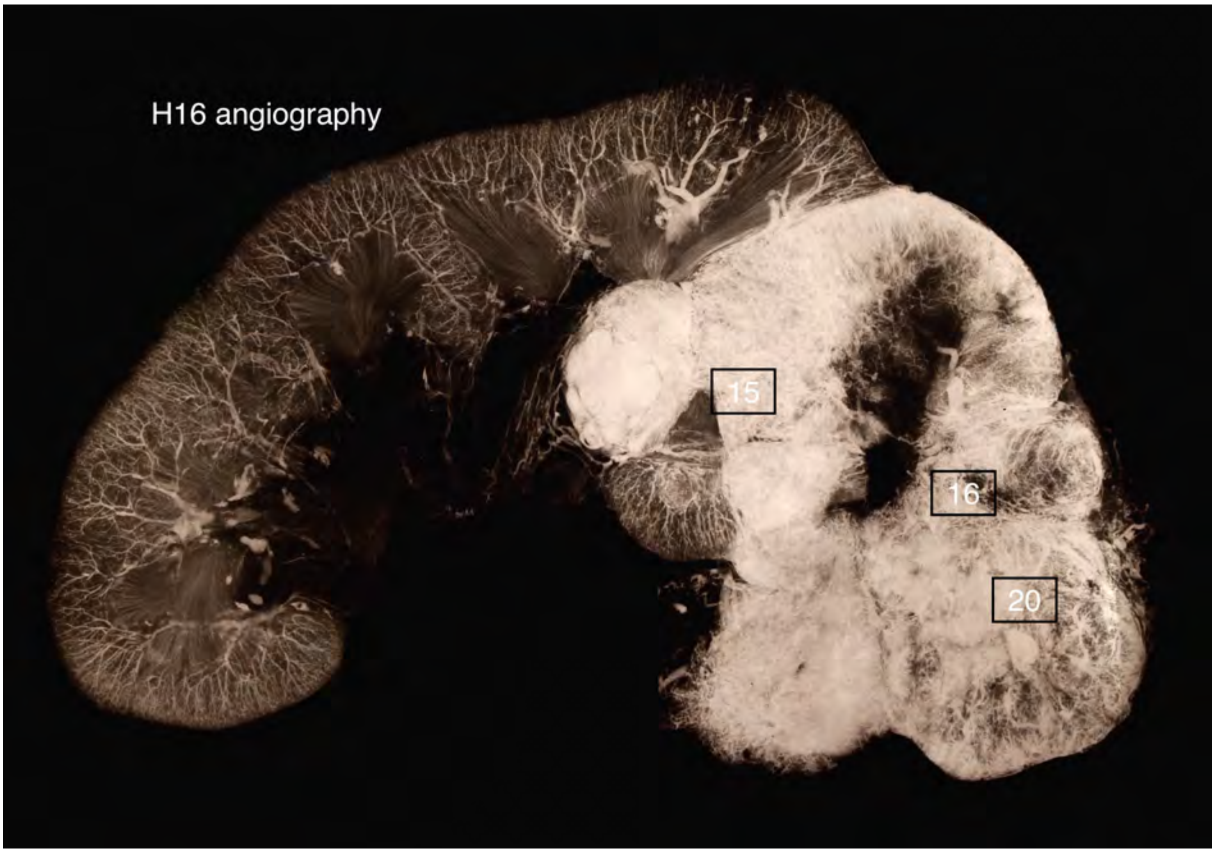

Rich vascularity with central necrosis, heterogenous 15 um sphere trapping

Autoradiography

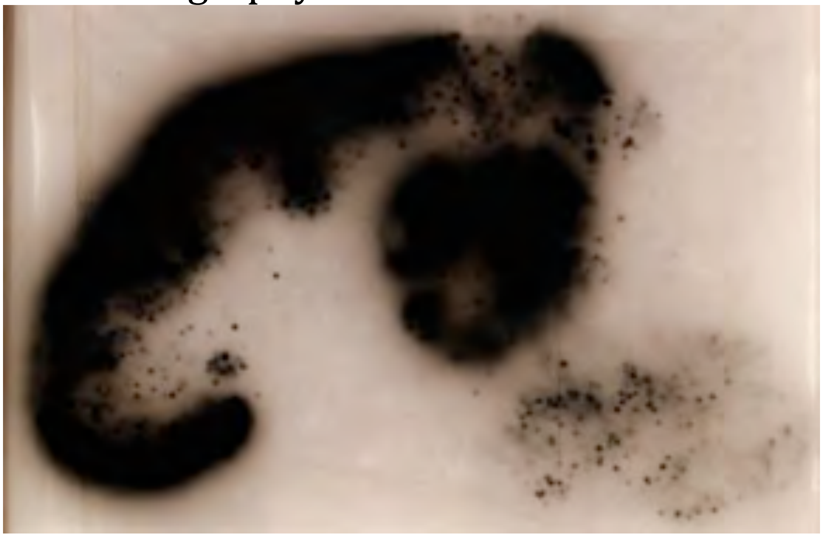

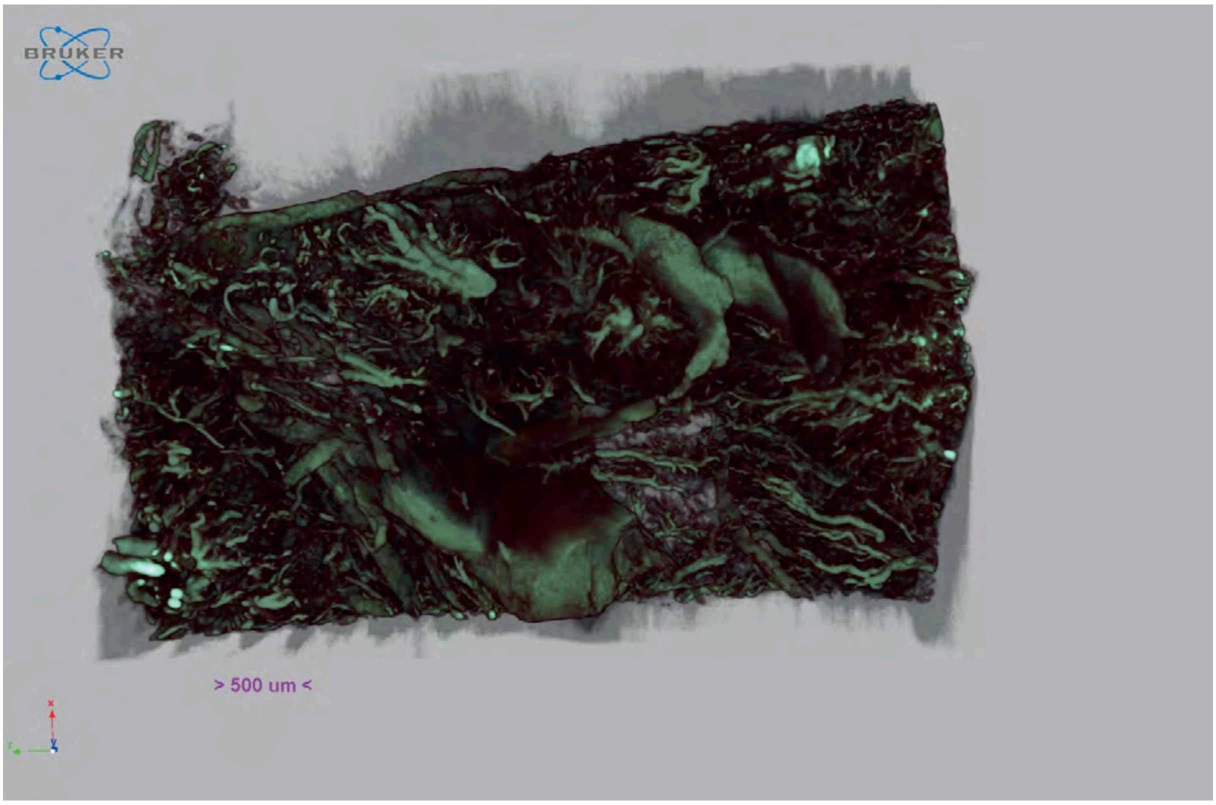

MicroCT Tumour H16:16

Dark-field macrophotography

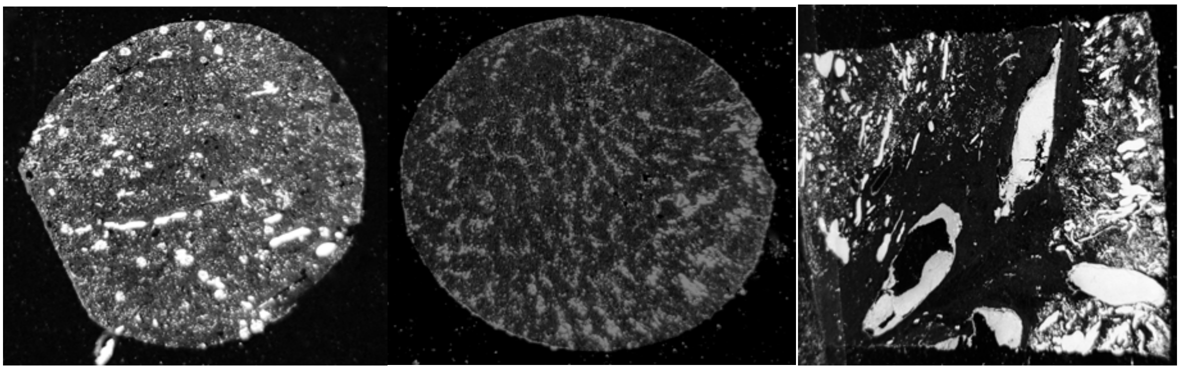

Cortex 16:1 P 0.13 Medulla 16:10 P 0.29 Tumour 16:16 P 0.74

Dark-field

Contrast CD31

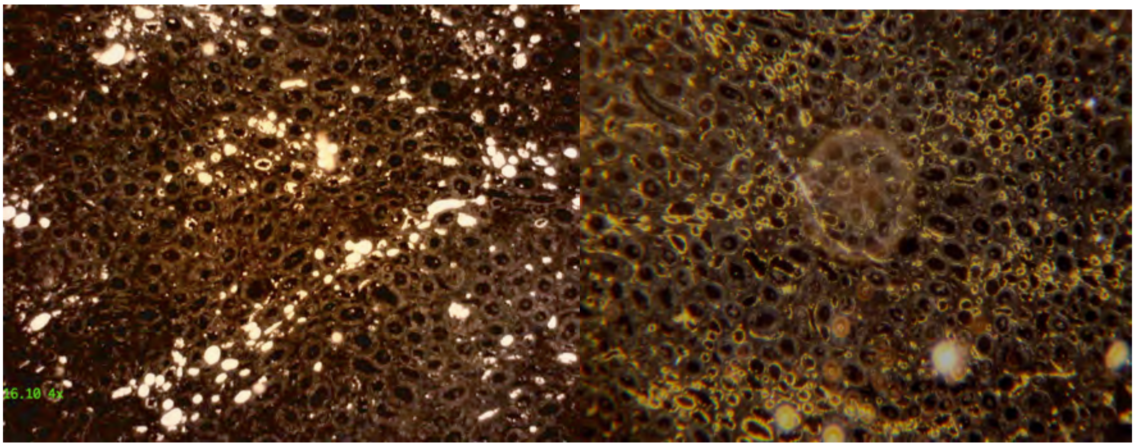

Cortex 16:1 PRU 0.13

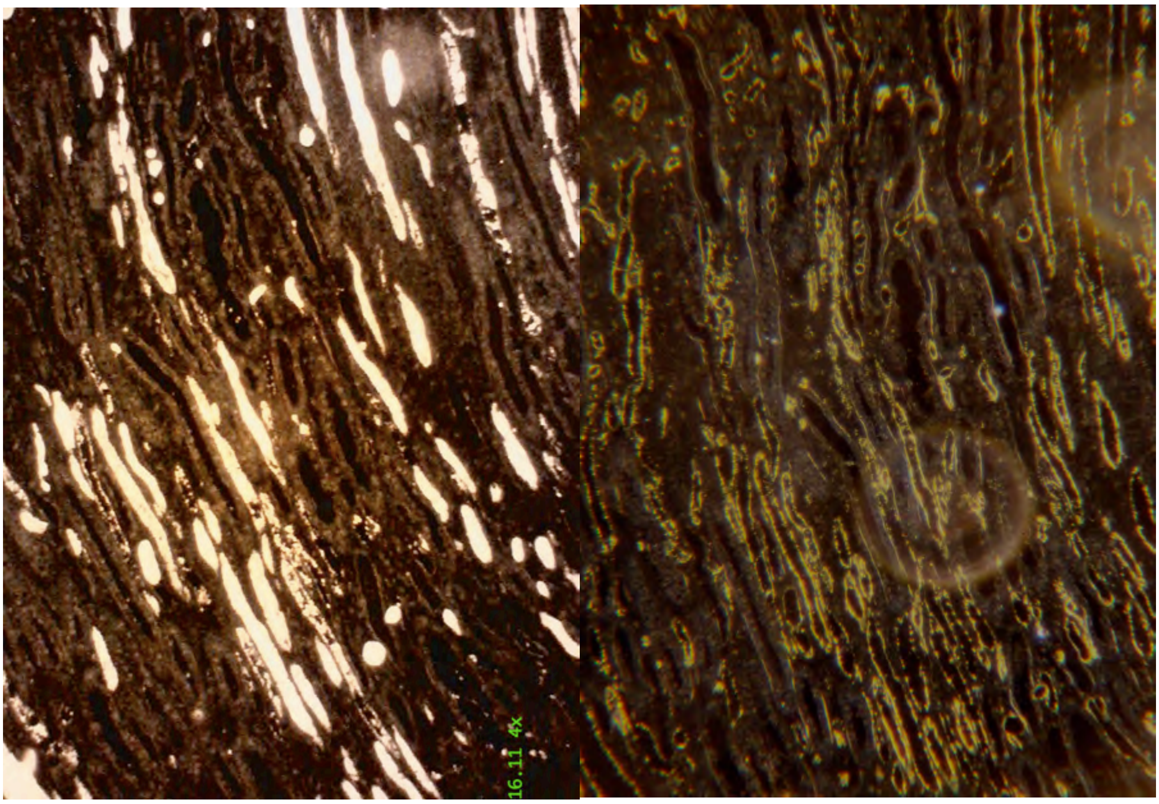

Medulla 16:10 PRU 0.29

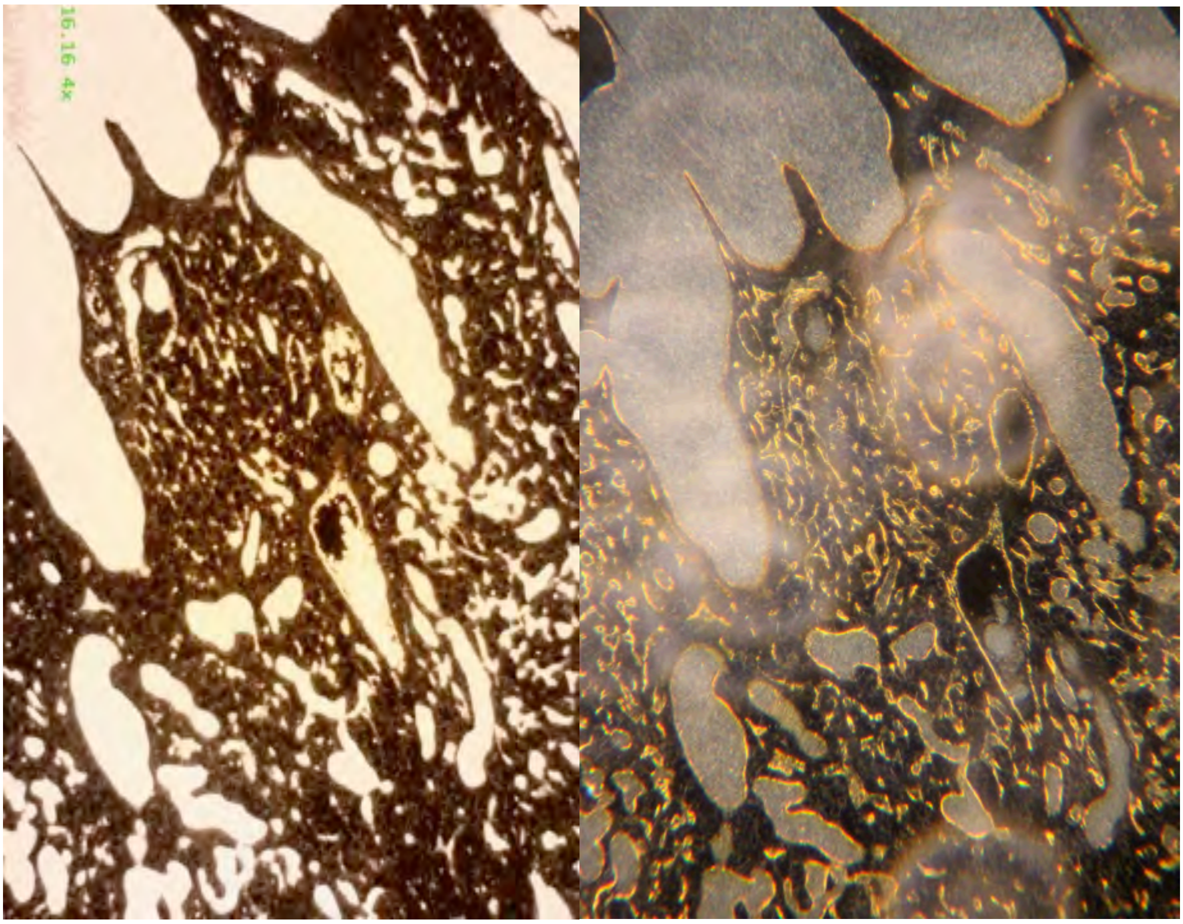

Tumour 16:16 PRU 0.74

Tumour sample HE-stained

Bright field

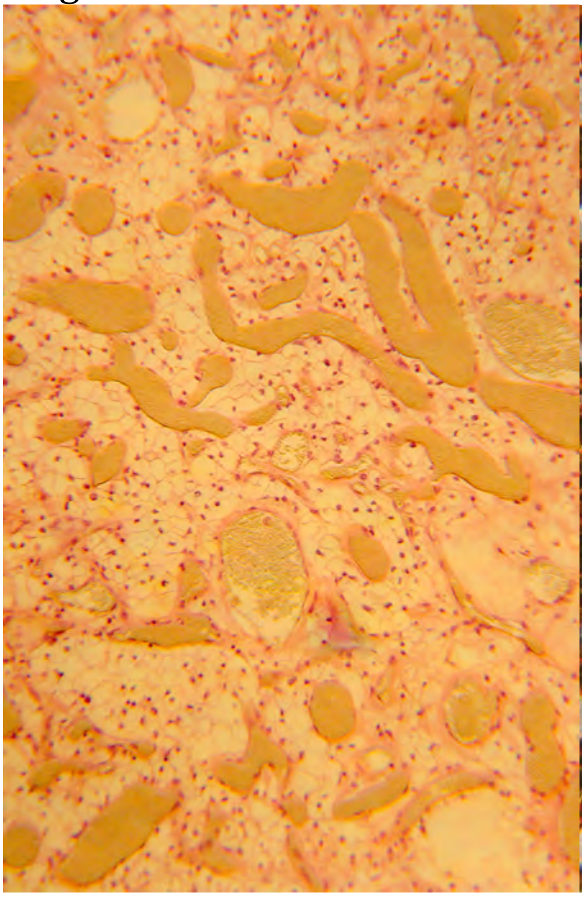

Darkfield

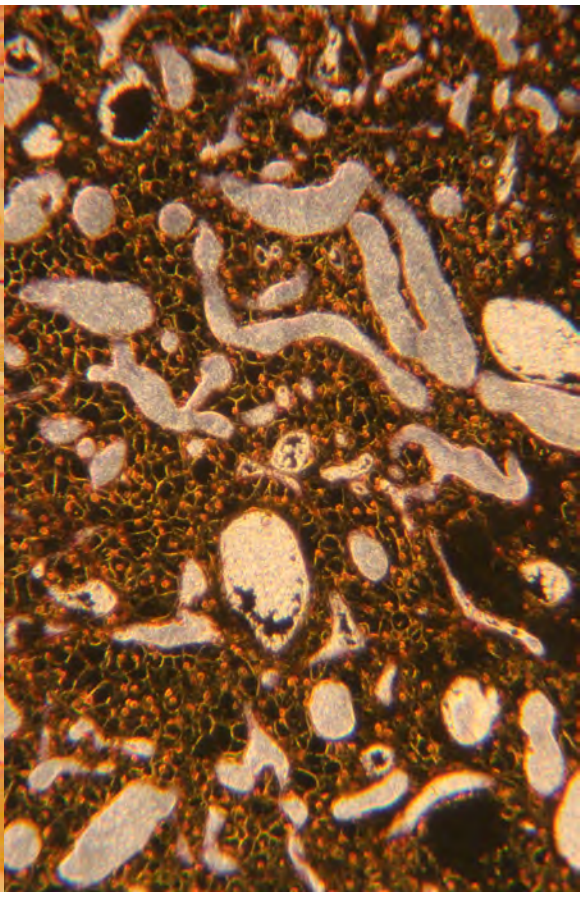

16:20 PRU 1.88

HE 20x

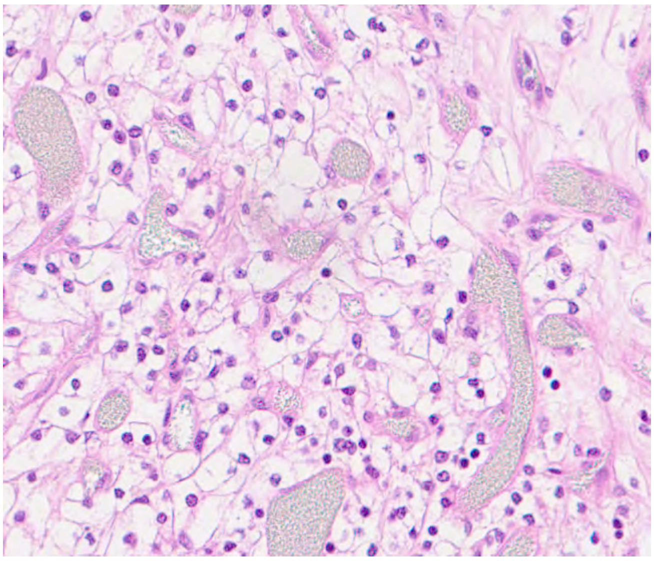

16:20 PRU 1.88

H17

Sex: Male
Age at surgery: 72 years
Survival from surgery: 4 years
Cause of death: other cause
Initial stage: 8 cm pT2 Nx M0
Tumour type: CCRCC ISUP grade 1
Tumour volume: 502 cm^3

Specimen weight: NE
Perfusion pressure: 56 mmHg Perfusate flow: 90 mL/min
Specimen PRU: 0.62
Cortical tissue PRU: NE n=13
Tumour tissue PRU span: NE n=35
Reference withdrawal failed

Angiography : Bar 100 mm

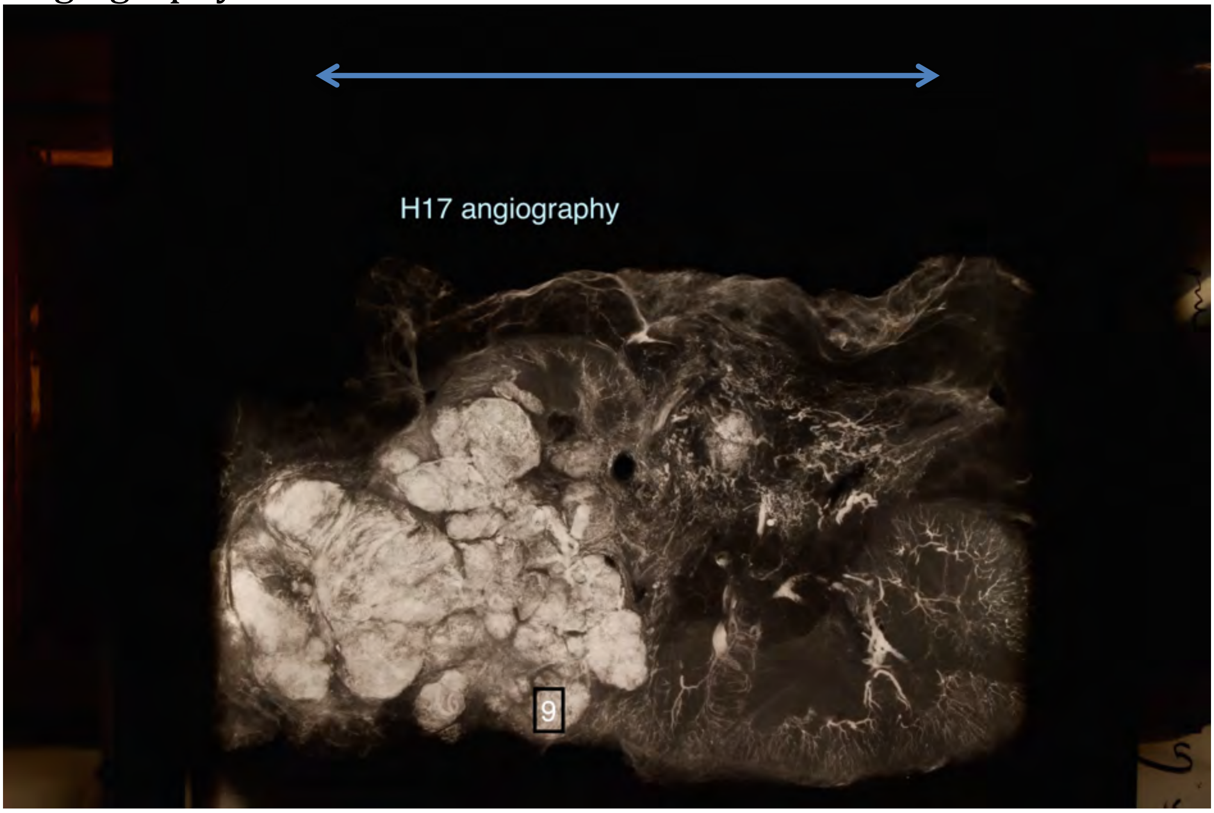

Lobular growth with intense vascularity with heterogenously high 15 um sphere trapping

Autoradiography

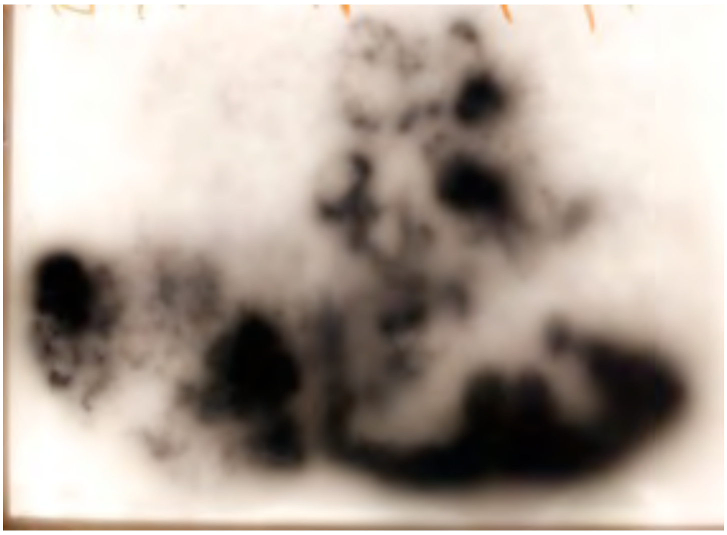

Darkfield macrophotography

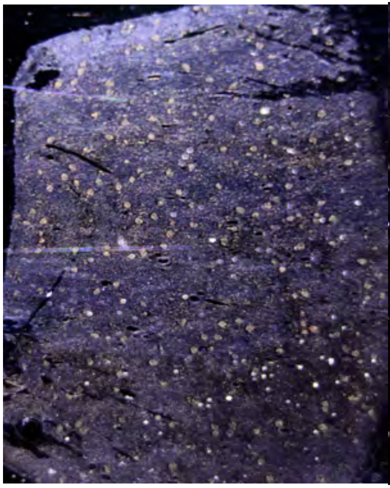

17:24 cortex PRU NE

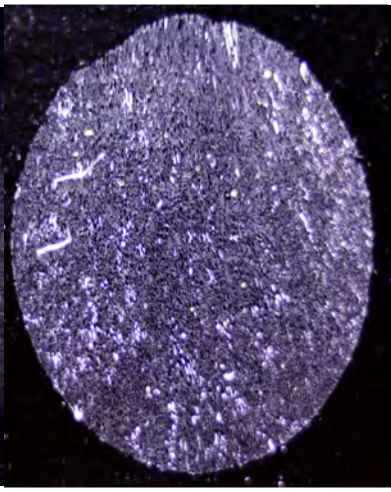

17:8 medulla P NE

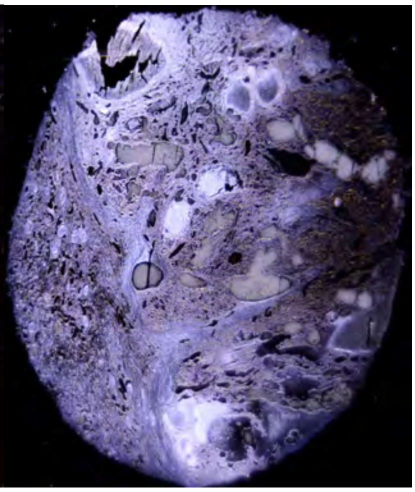

17:9 tumour P NE

Darkfield CD31

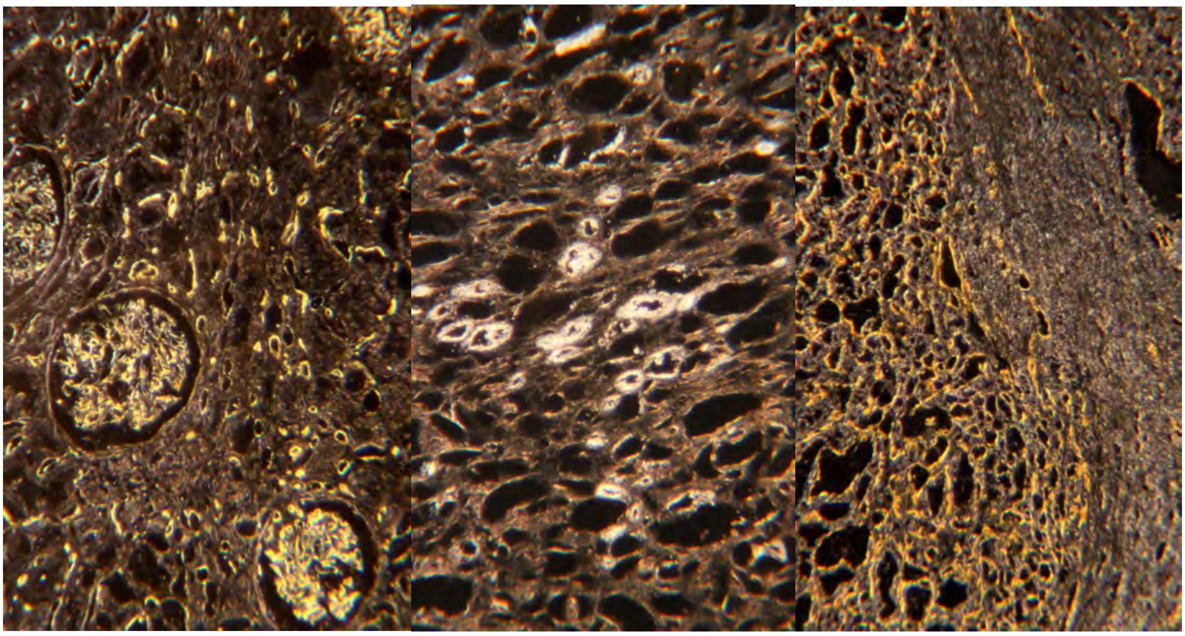

Cortex 17:24 PRU$_{rel}$ 1 Medulla 17:9 P$_{rel}$ Tumour 17:9 P$_{rel}$ 0.43

Tumour HE 16x

Brightfield

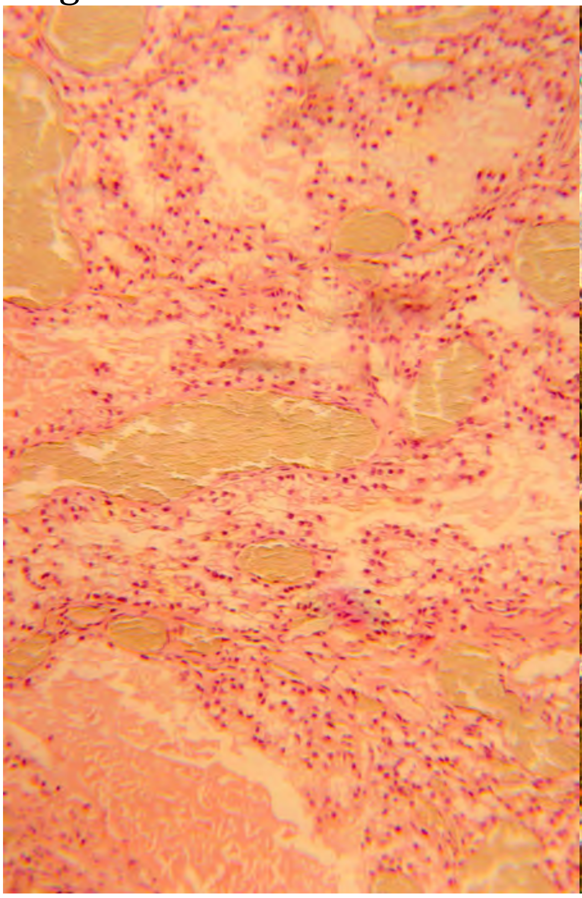

Darkfield

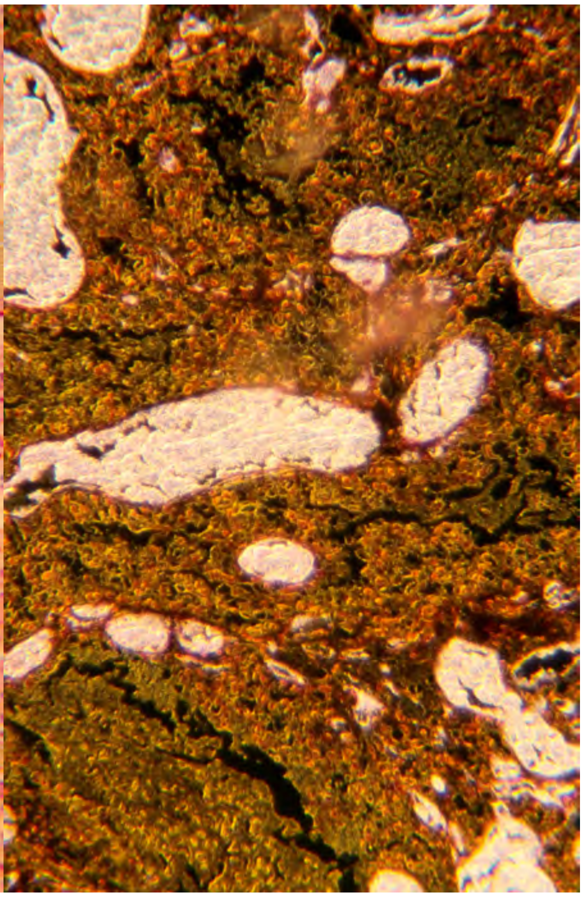

17:9 PRU$_{rel}$ 0.43

HE 20x

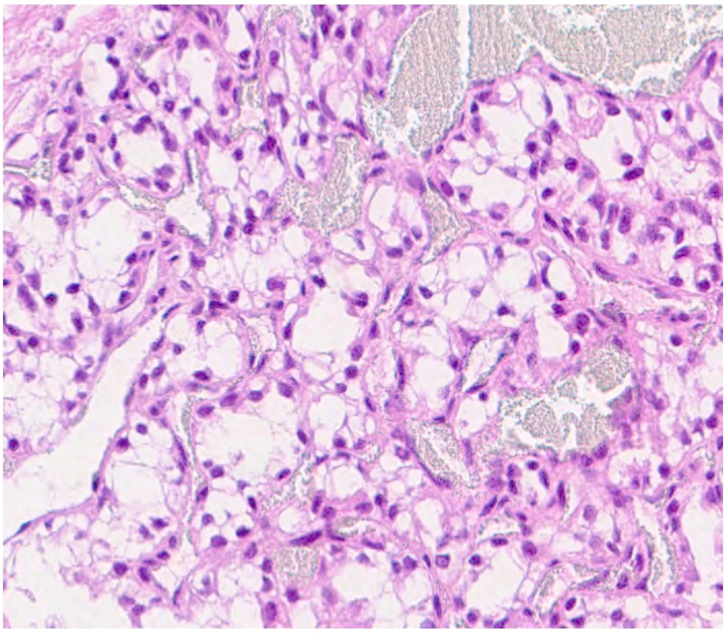

17:9 20x PRU_{rel} 0.43

H18

Sex: Female
Age at surgery: 62 years
Survival from surgery: 20 years
Cause of death: other cause
Initial stage:10x8 cm pT2 Nx M0
Tumour type: CCRCCgranulated ISUP grade 2
Tumour volume: 252 cm^3

Specimen weight: 606 g
Perfusion pressure: 30 mmHg   Perfusate flow: 43 mL/min
Specimen PRU: 0.70
Cortical tissue PRU: 0.25 +/-0.04 n=5
Tumour tissue PRU span: 0.39-37   n=18

Fresh section: Bar 100 mm

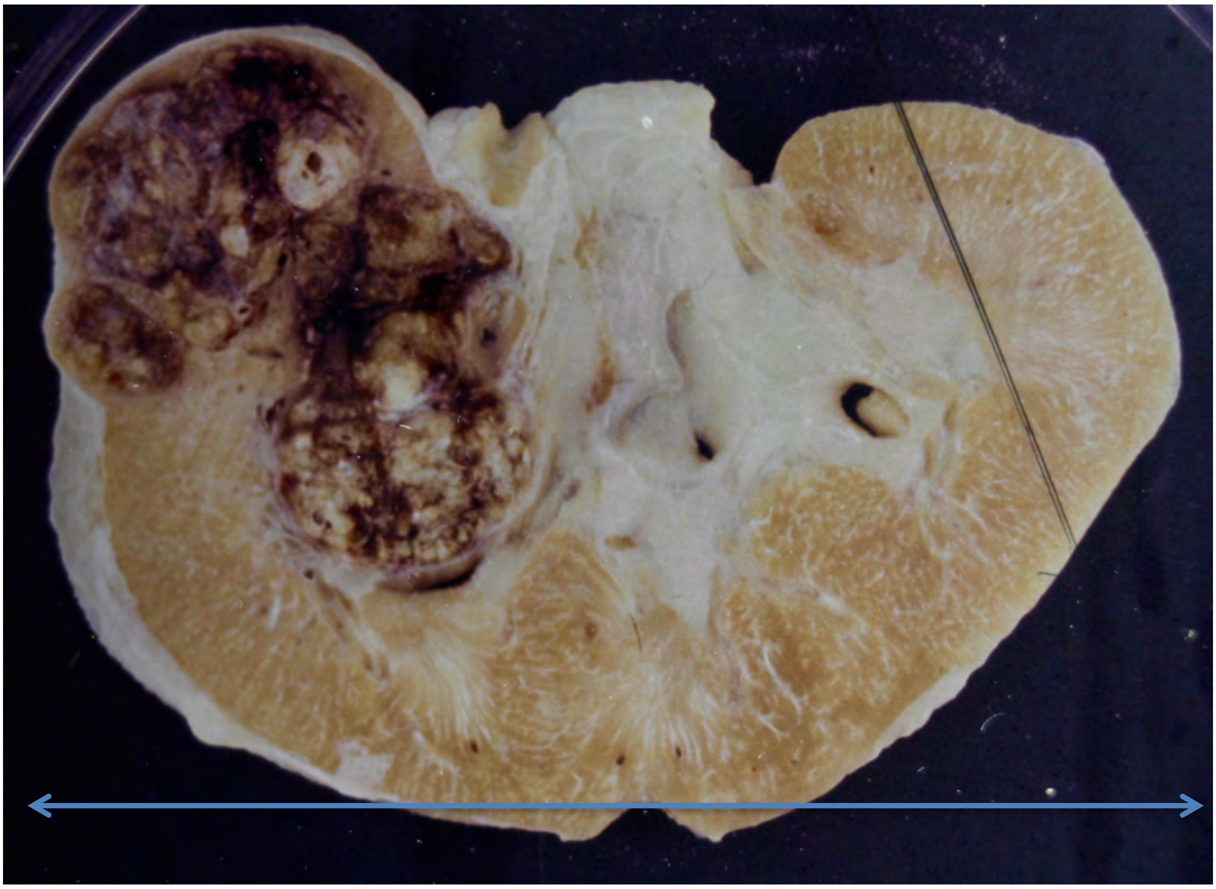

Angiography

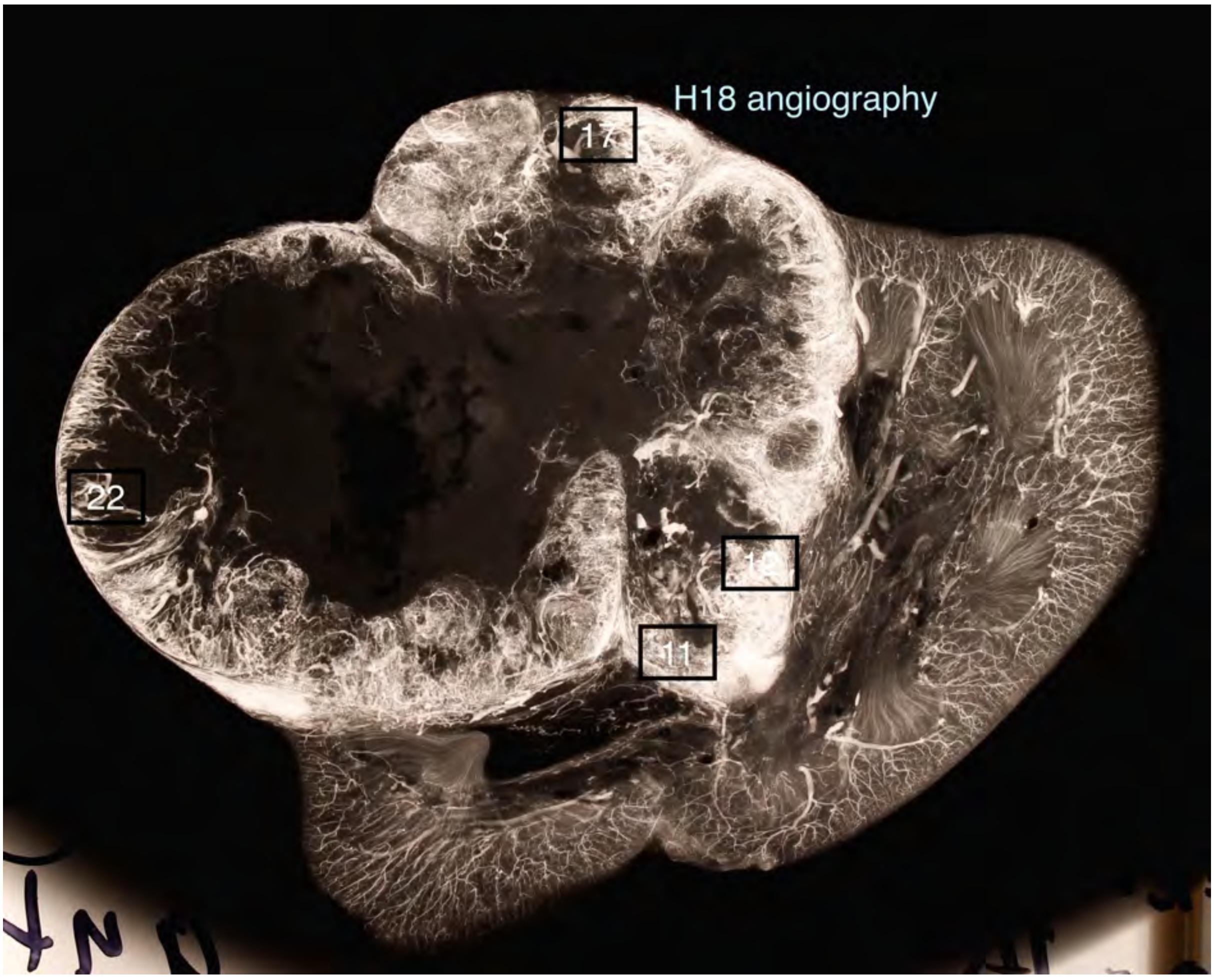

Peripheral rich vascularity with low 15 um sphere trapping. Extensive central necrosis.

Autoradiography

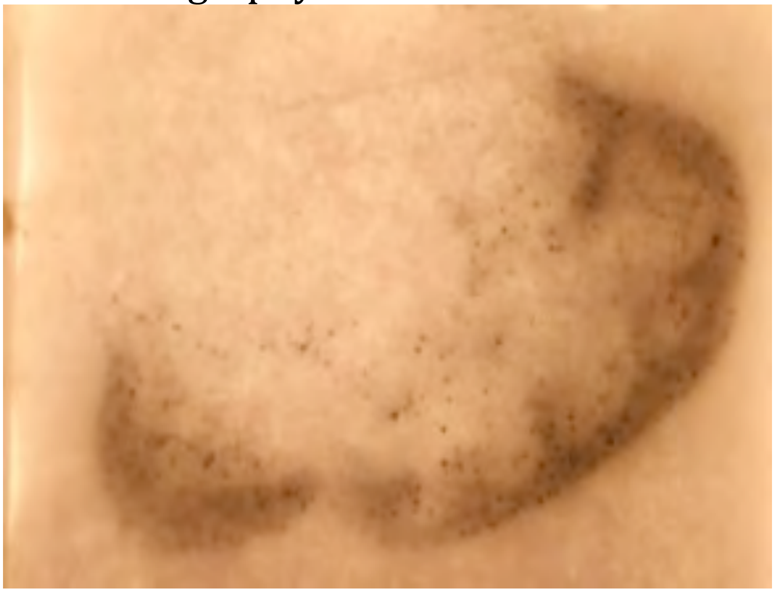

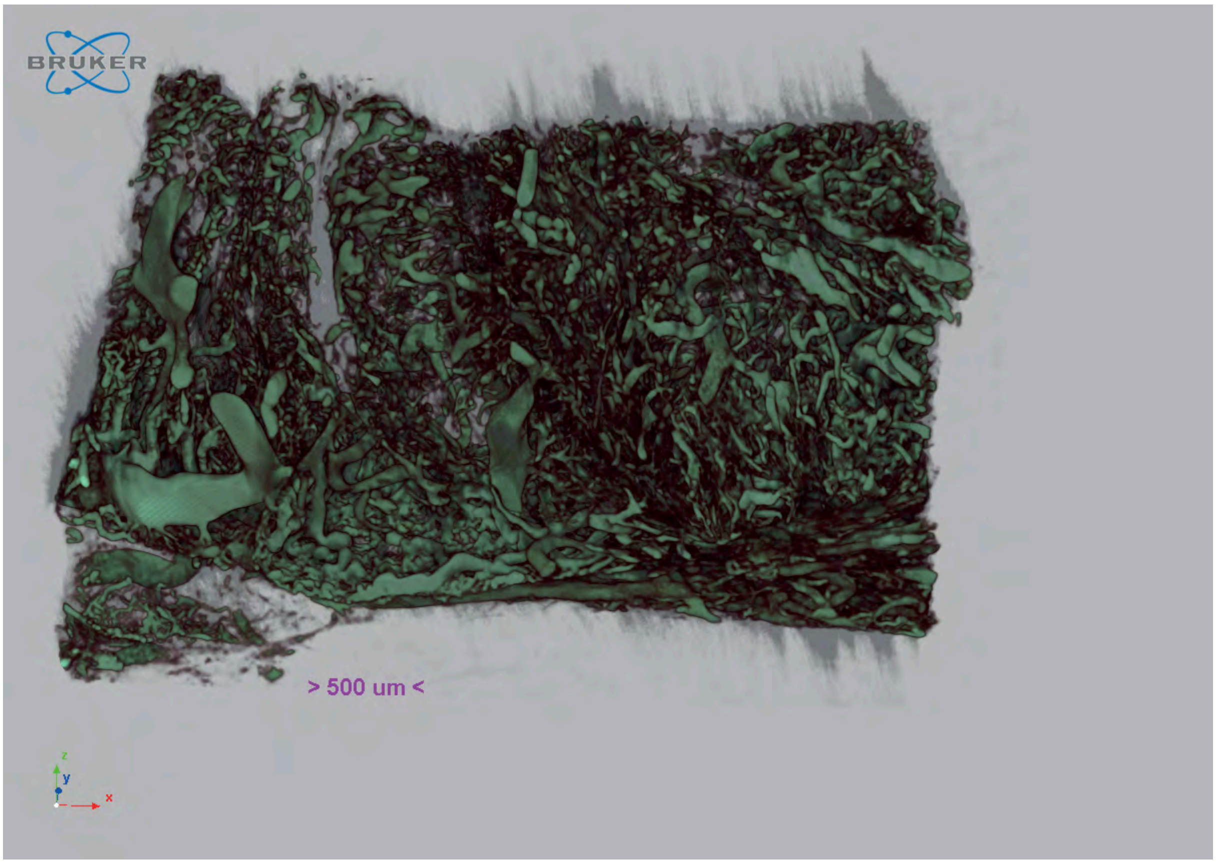

MicroCT Tumour H18:22

Dark-field macrophotography

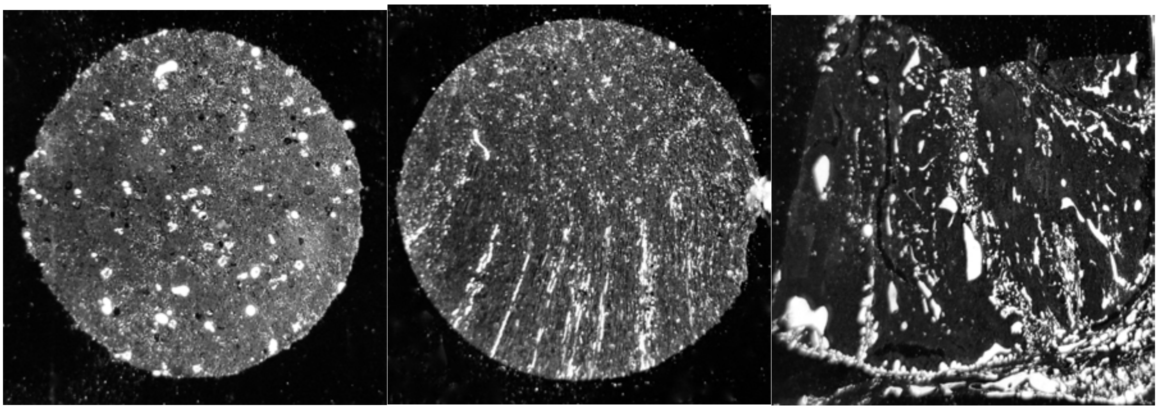

Cortex 18:3 P 0.18 Medulla 18:9 P 0.93 Tumour 18:22 P 5.1

Dark-field

Contrast CD31

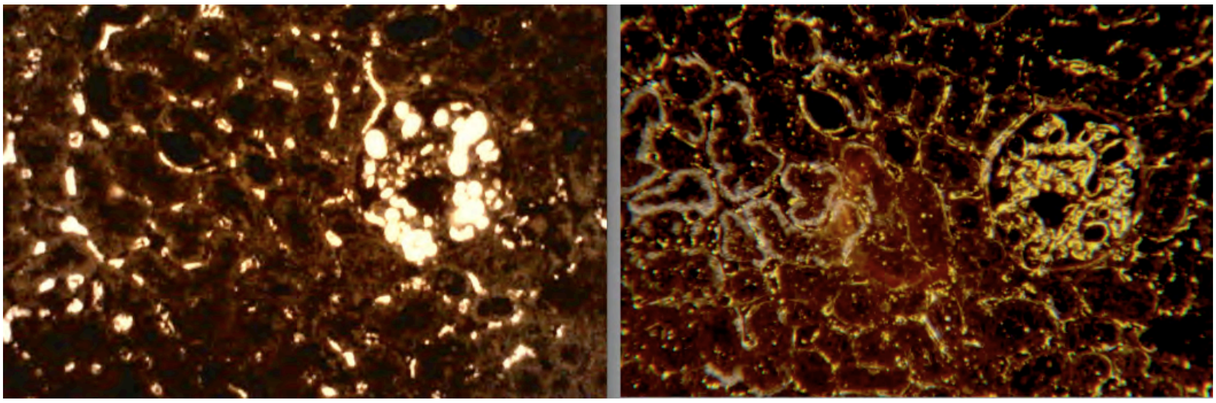

Cortex 18:3 PRU 0.18

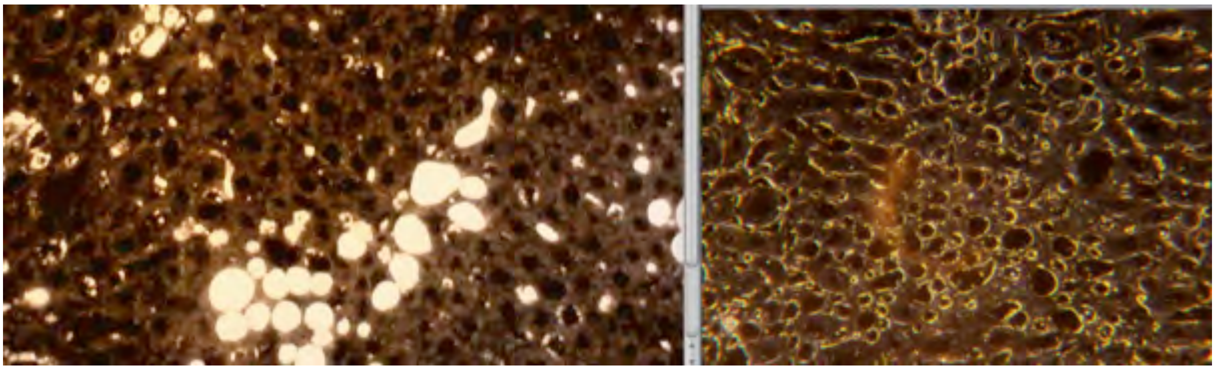

Medulla 18:4 PRU 0.27

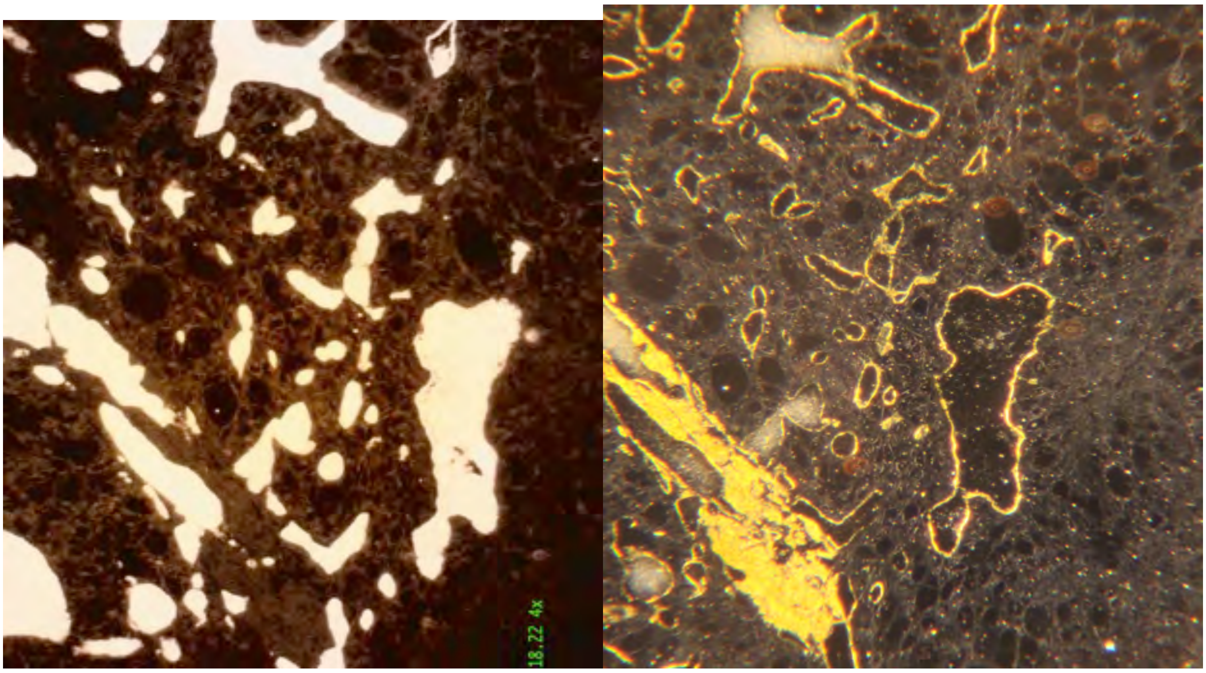

Tumour 18:22 PRU 5.1

Tumour sample HE stained

Bright field

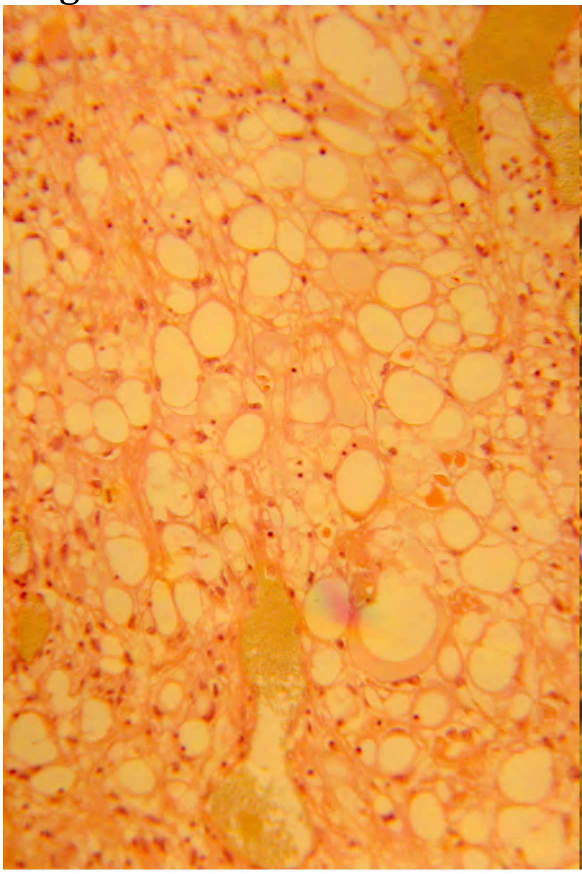

Darkfield

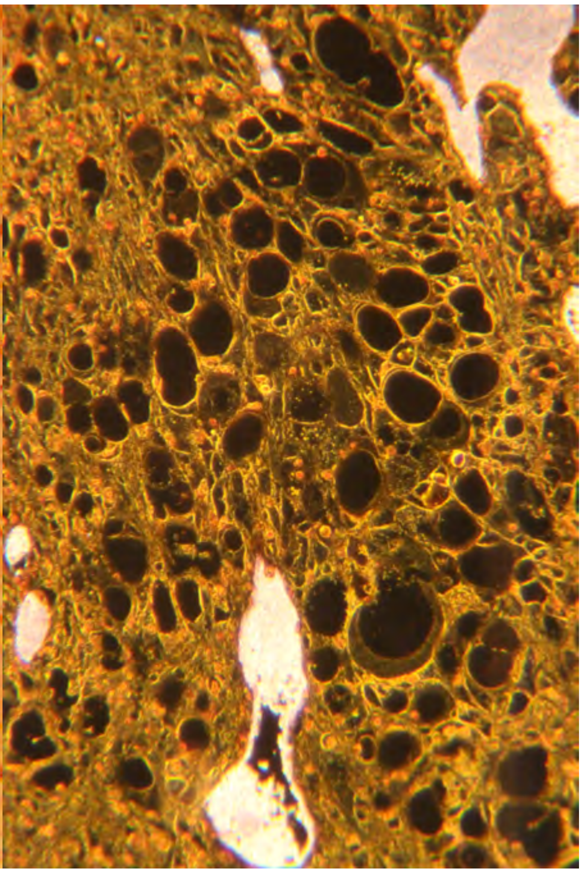

18:22 PRU 5.1

HE 20x

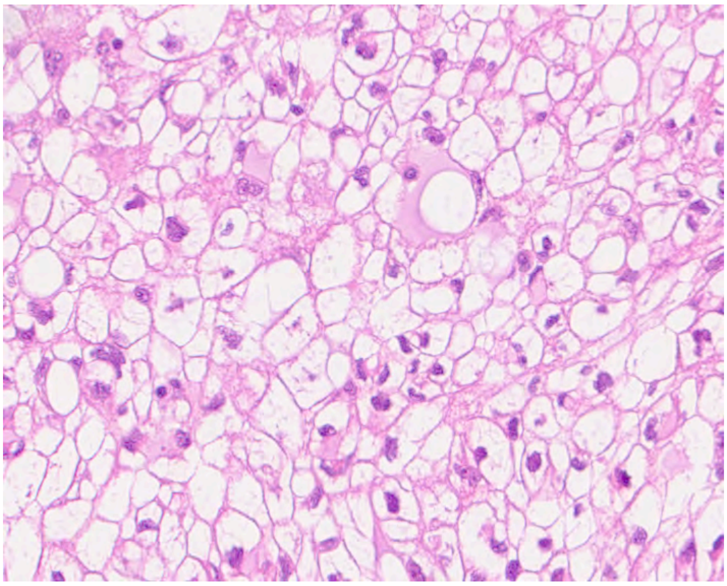

18:22 PRU 5.1

H19

Sex: Male
Age at surgery: 71 years
Survival from surgery: 16 years
Cause of death: other cause
Initial stage: T2.5 cm pT1a Nx M0
Tumour type: Eosinophilic CCRCC ISUP grade 3
Tumour volume: 11 cm^{3},

Specimen weight: 569 g
Perfusion pressure: 52 mmHg Perfusate flow: 33 mL/min
Specimen PRU: 1.58
Cortical tissue PRU: 0.16+/-0.04 n=10
Tumour tissue PRU span: 0.24-0.94 n=10

Angiography: Bar 100 mm

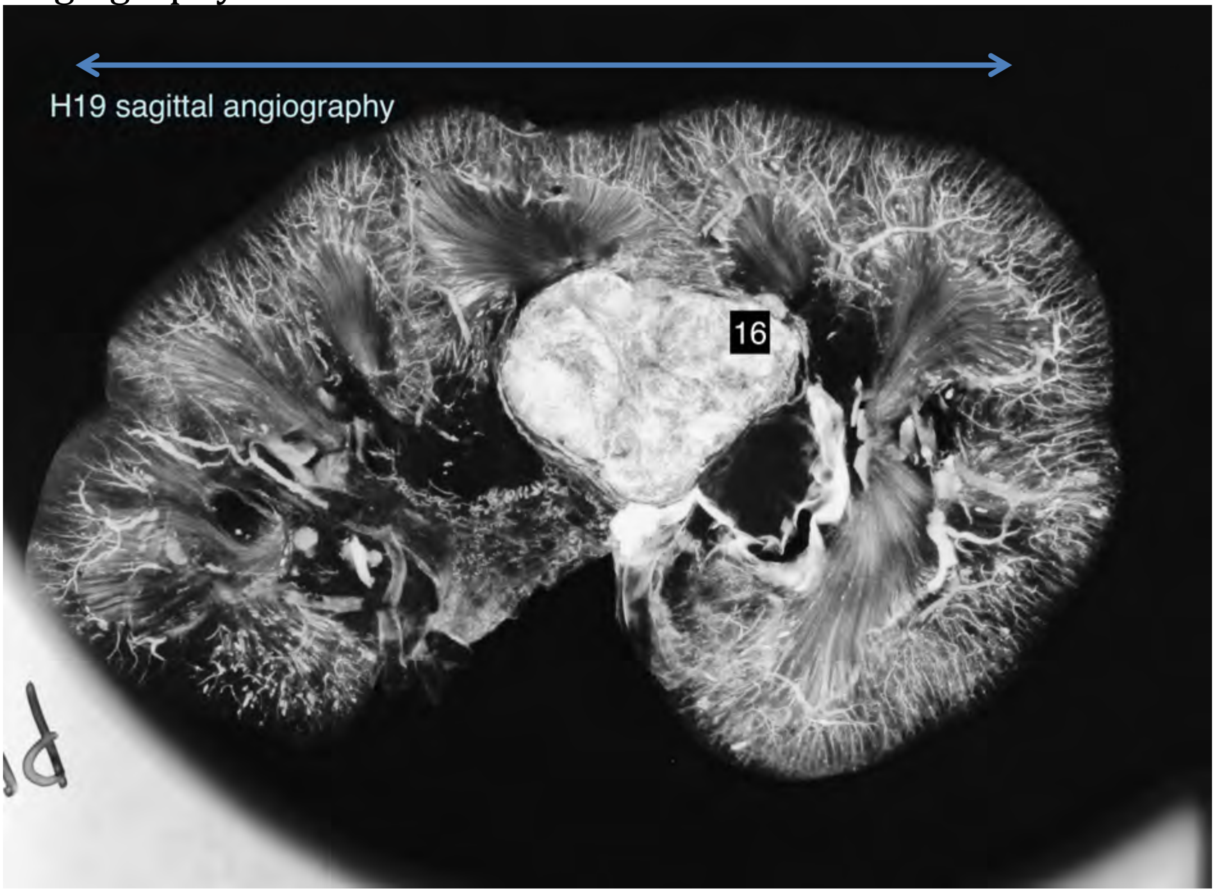

Intense vascularity with high 15 um sphere trapping

Autoradiography

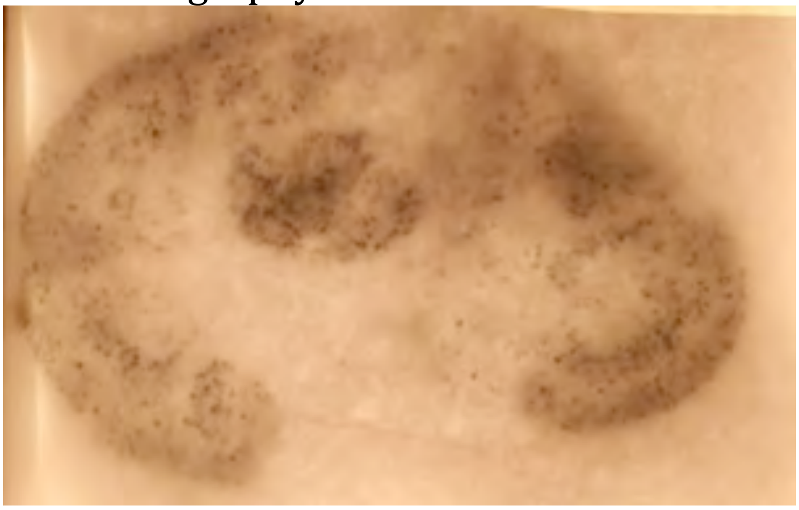

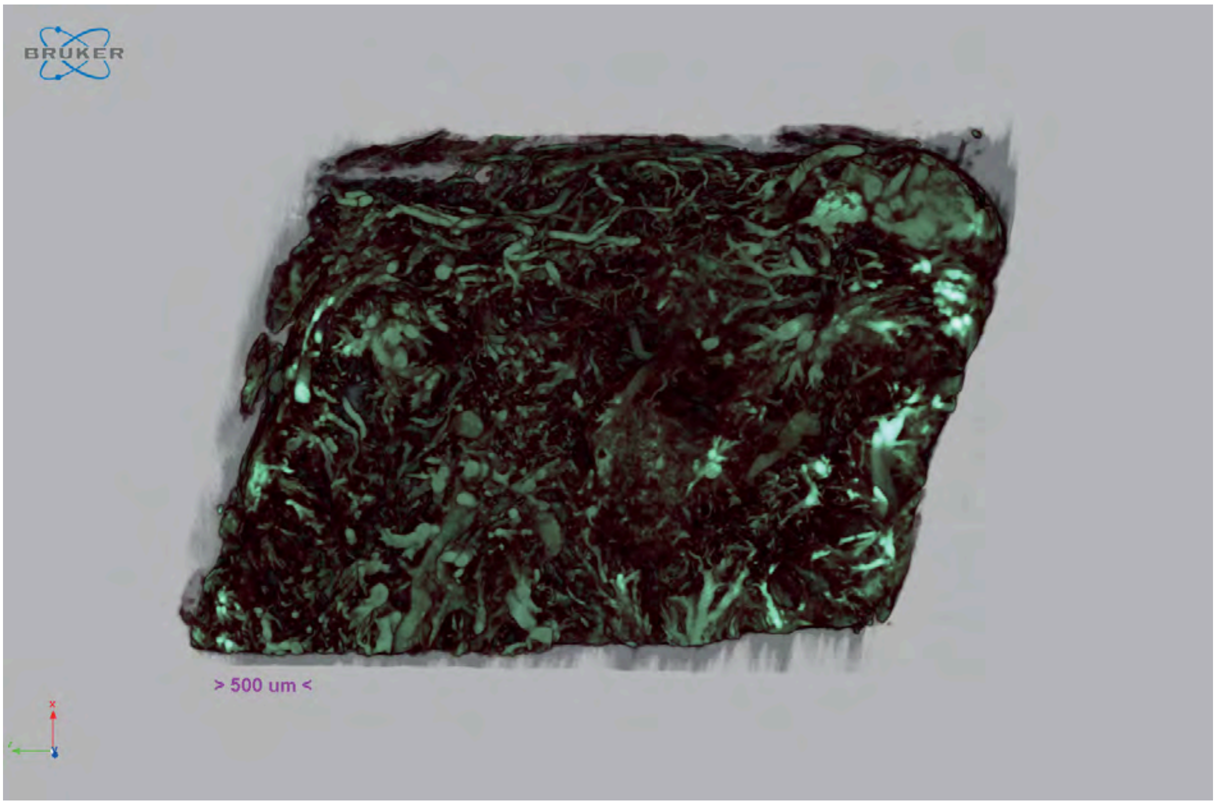

MicroCT Tumour H19:16

Dark-field macrophotography

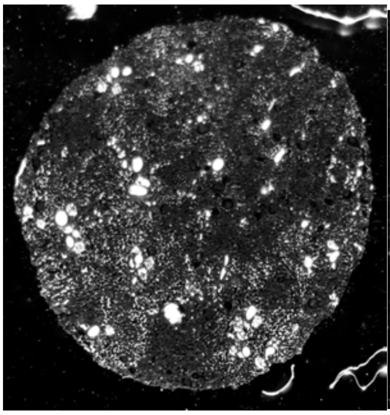

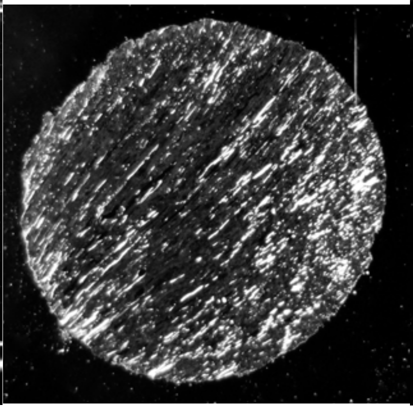

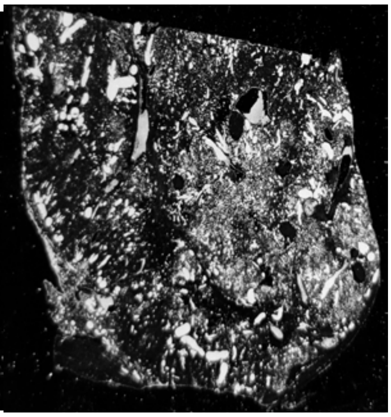

Cortex 19:5 P 0.21 Medulla 19:11 P 0.95 Tumour 19:16 P 0.45

Darkfield

Contrast CD31

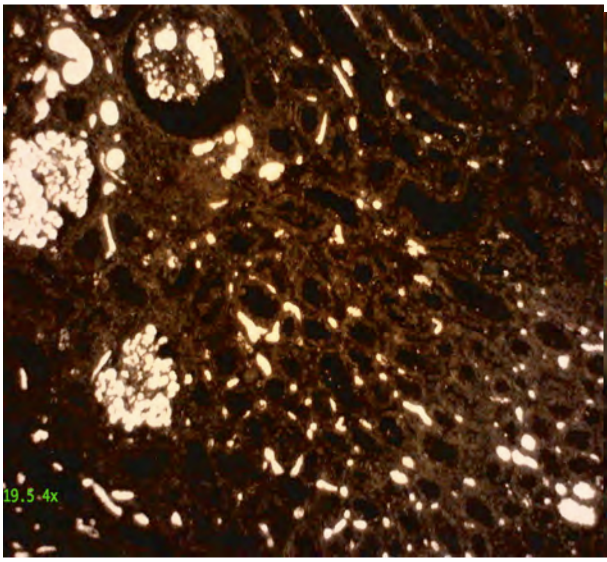

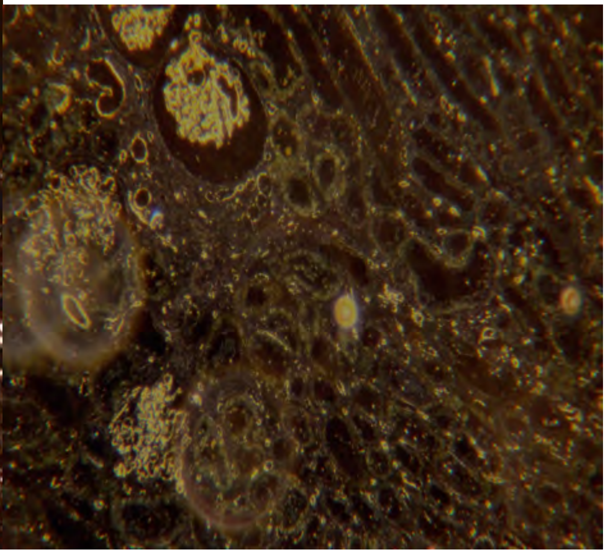

Cortex 19:5 PRU 0.21

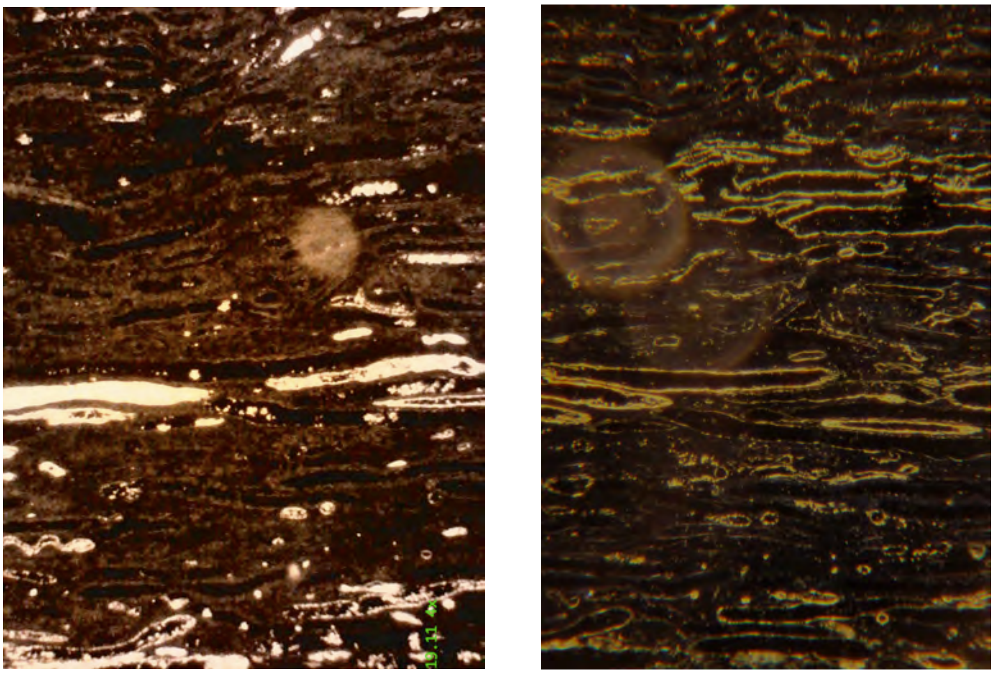

Medulla 19:11 PRU 0.95

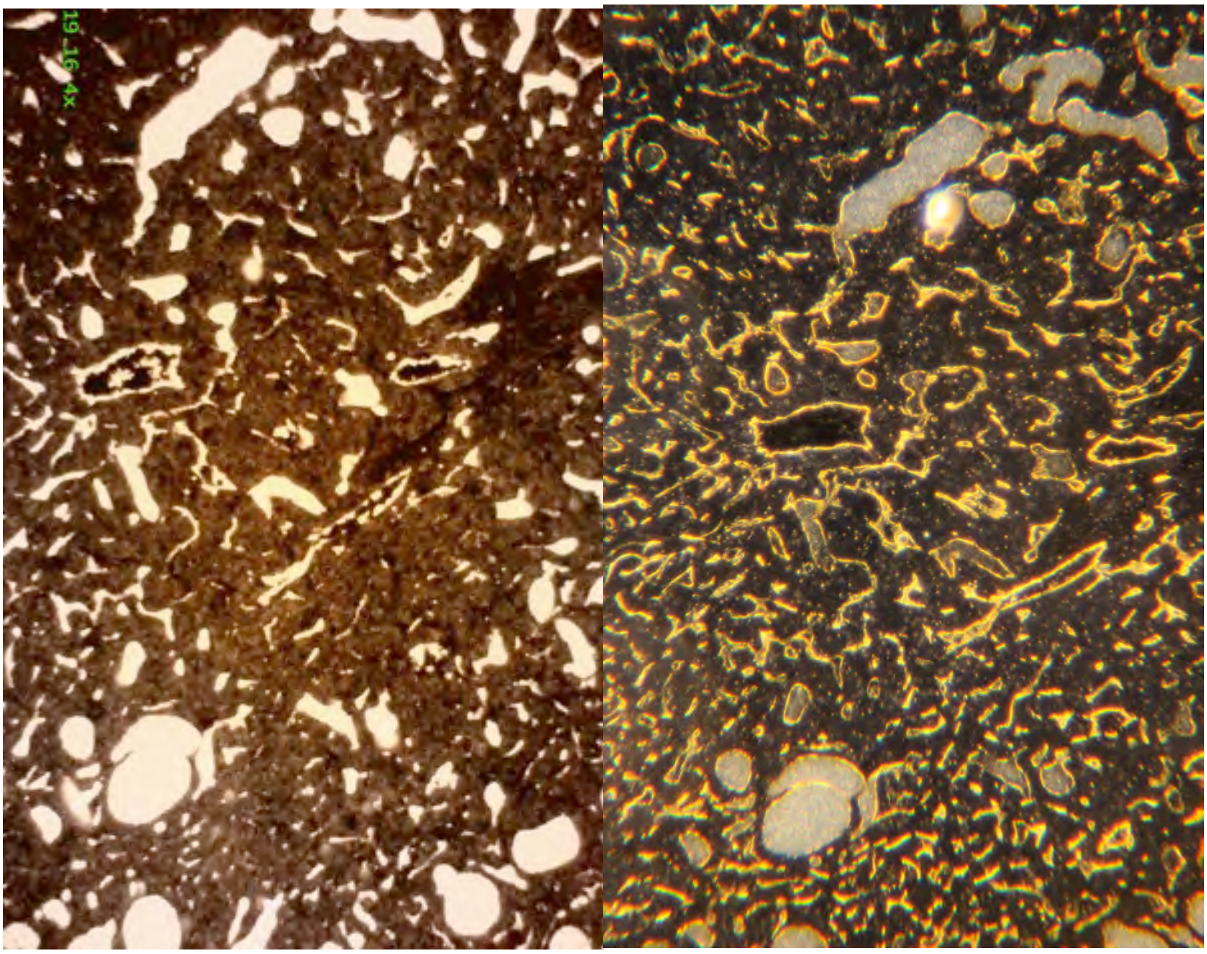

Tumour 19:16 PRU 0.45

Tumour sample HE stained

Bright field

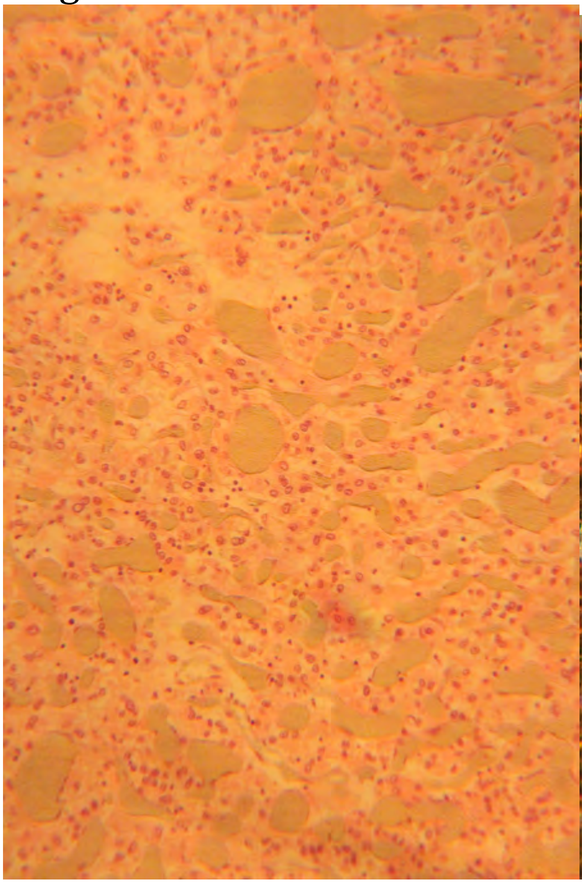

Darkfield

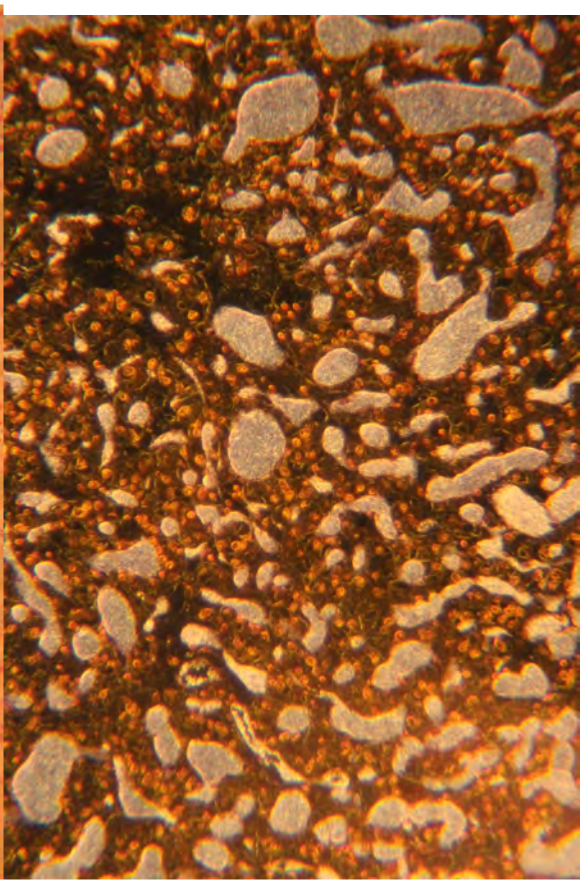

19:16 PRU 0.45

HE 20x

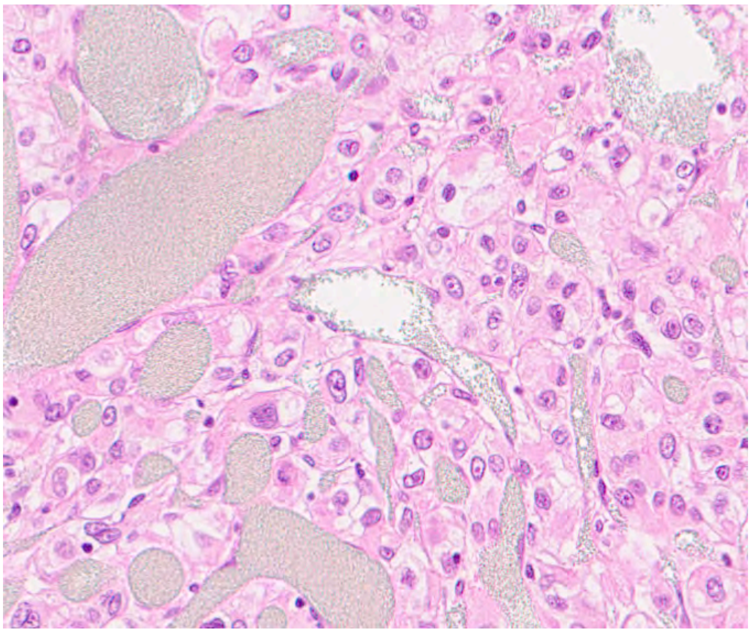

19:16 20x PRU 0.45

H20

Sex: Male
Age at surgery: 51 years
Survival from surgery: 28 years
Cause of death: other cause
Initial stage: T8x5 cm pT2 Nx M0
Tumour type: CCRCC eosinophilic ISUP grade 2
Tumour volume: 130 cm^3

Specimen weight: 737 g
Perfusion pressure: 38 mmHg Perfusate flow: 24 mL/min
Specimen PRU: 1.58
Cortical tissue PRU: 0.36+/-0.09 n=9
Tumour tissue PRU span: 0.52-10 n=23

Fresh section: Bar 100 mm

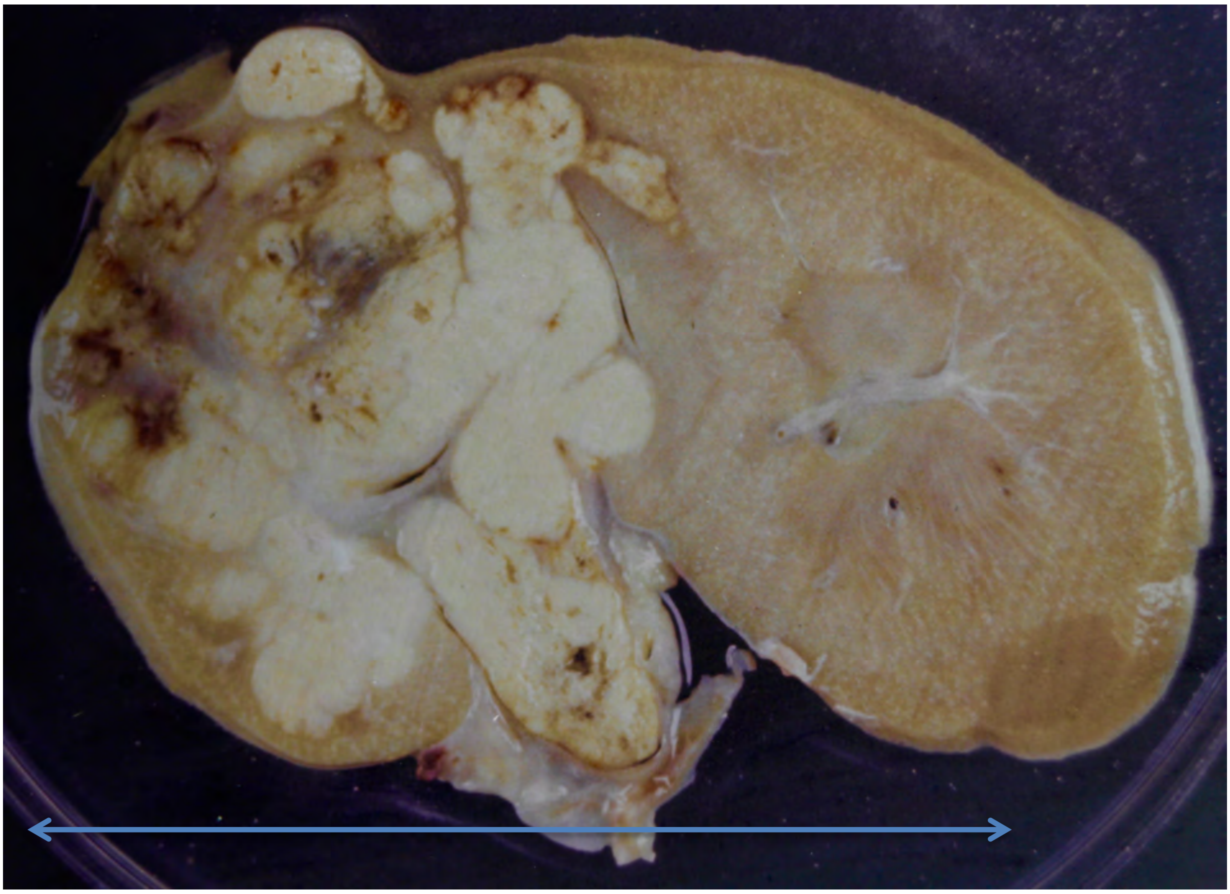

Angiography

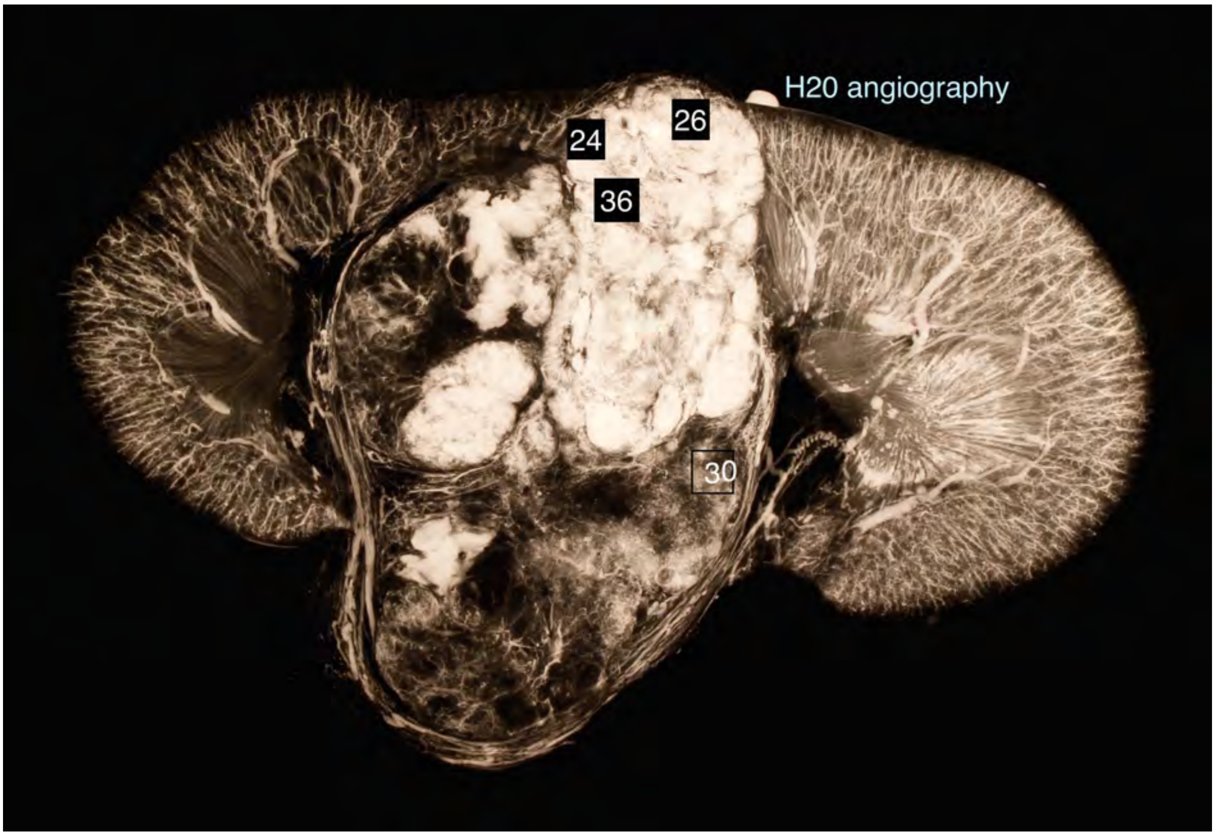

Lobular growth with heterogenous vascularity and 15 um sphere trapping

Autoradiography

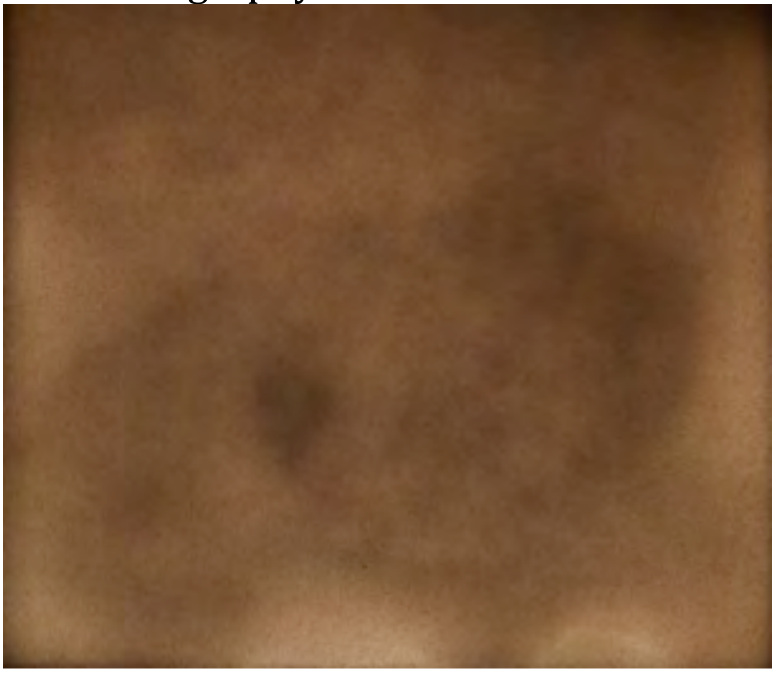

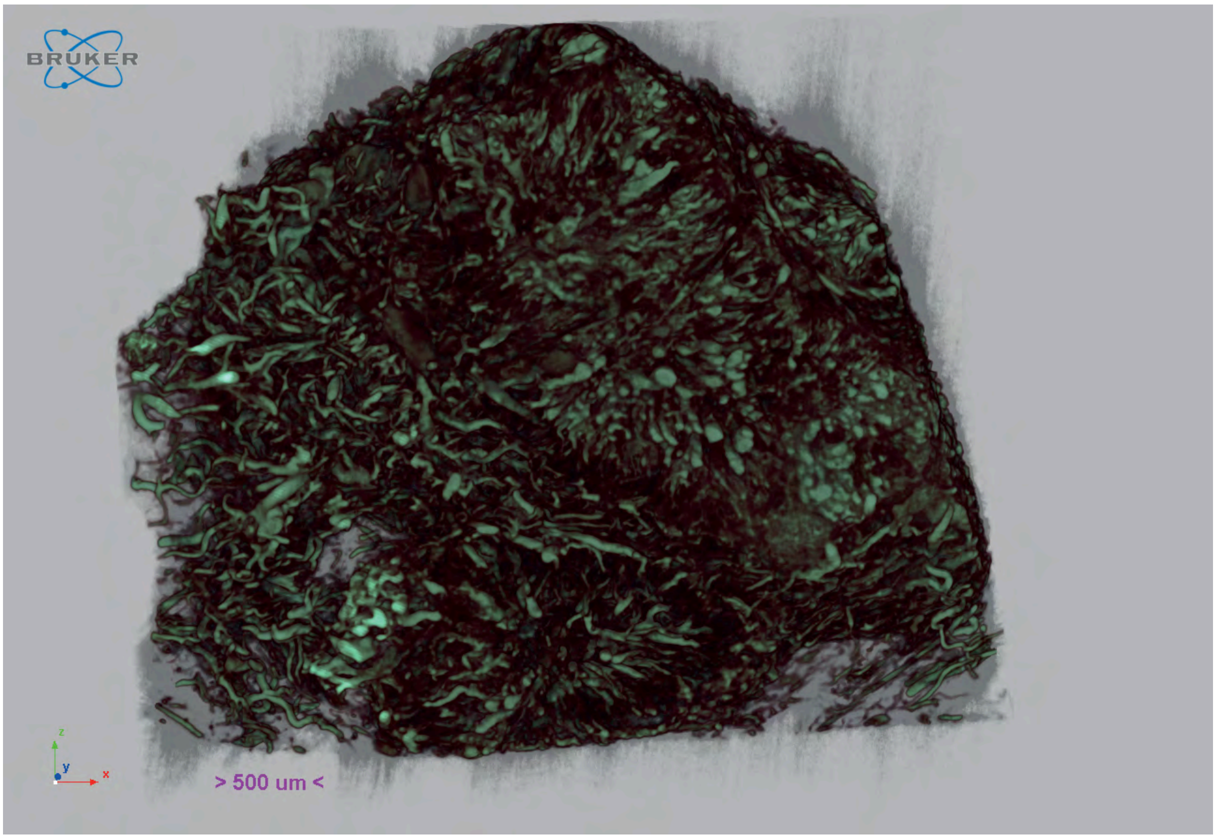

MicroCT Tumour H20:30

Dark-field macrophotography

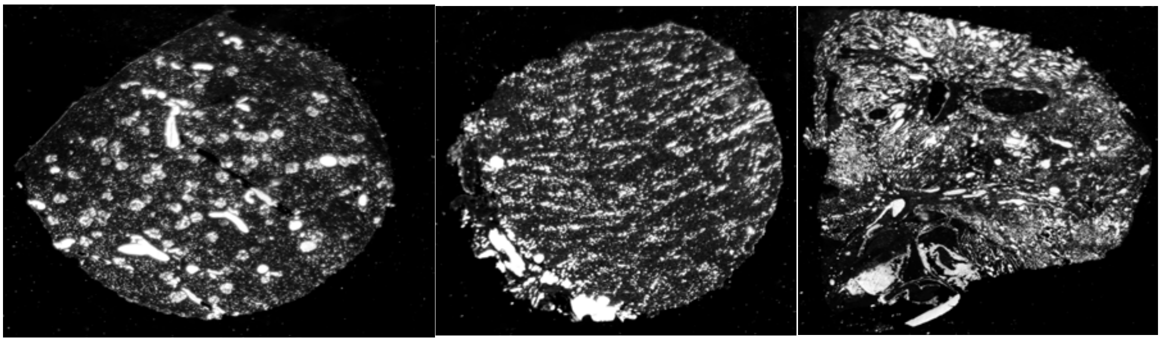

Cortex 20:1 P 0.31 Medulla 20:13 P 5.67 Tumour 20:30 P 0.9

Darkfield
Contrast   CD31

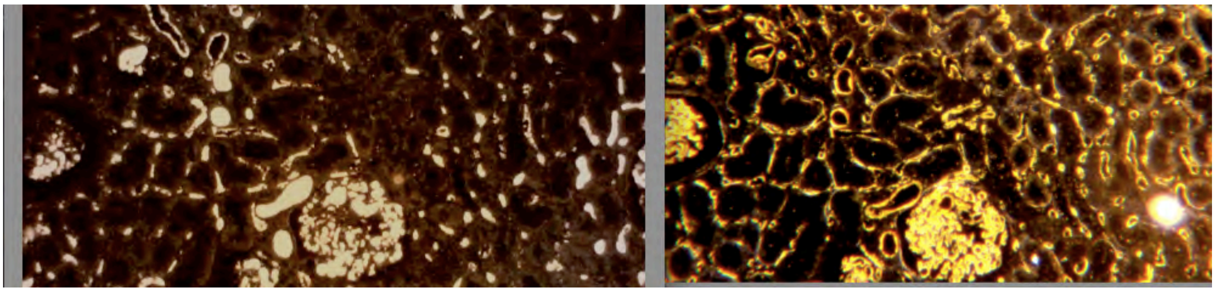

cortex 20:1 PRU 0.31

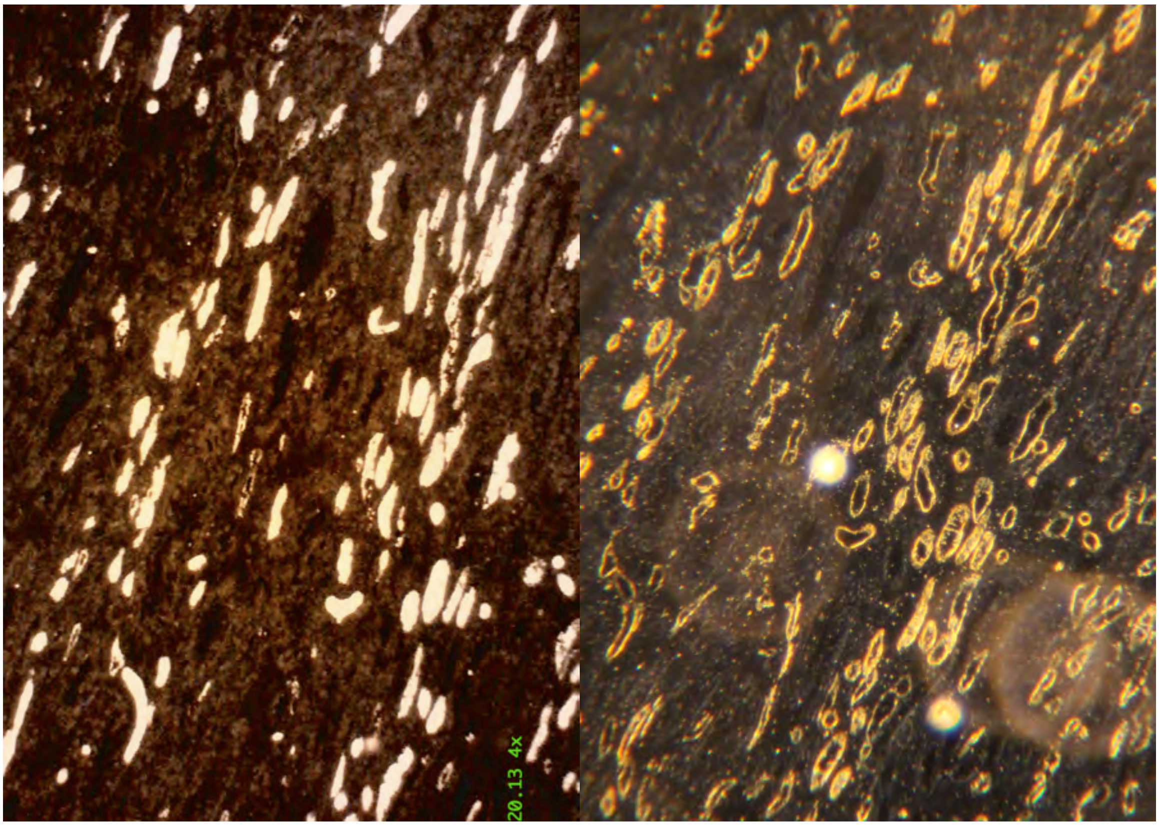

Medulla 20:13 PRU 5.67

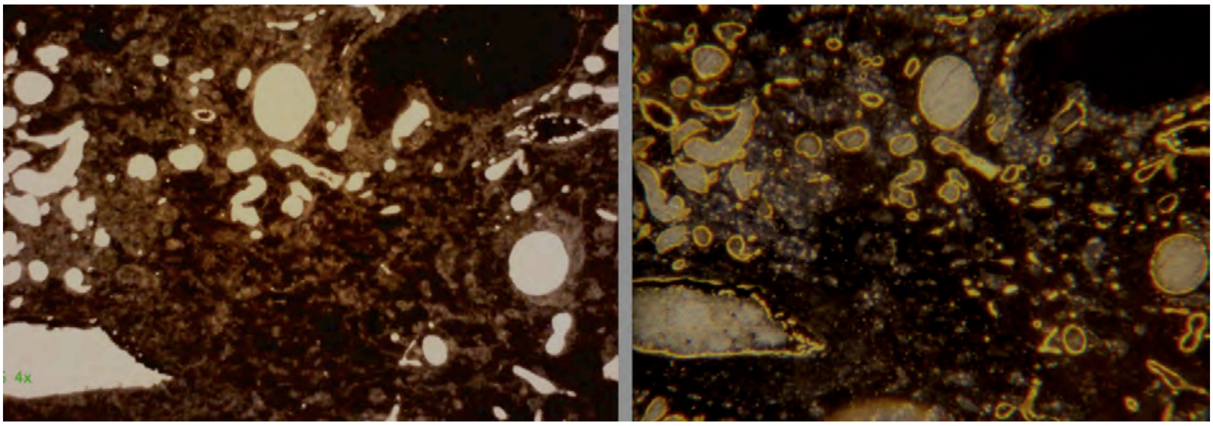

Tumour 20:36 PRU 1.27

Tumour sample HE stained

Bright field Darkfield

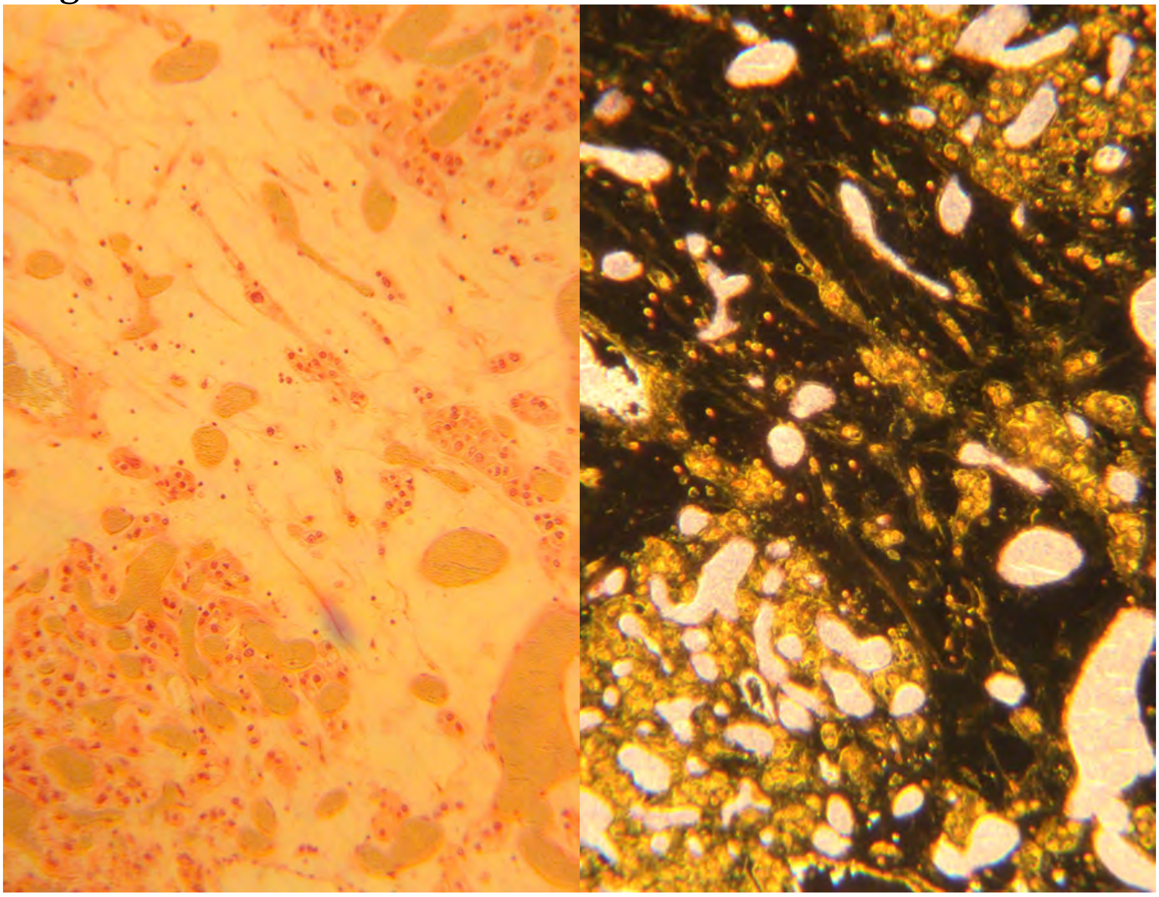

20:36 PRU 1.27

HE 20x

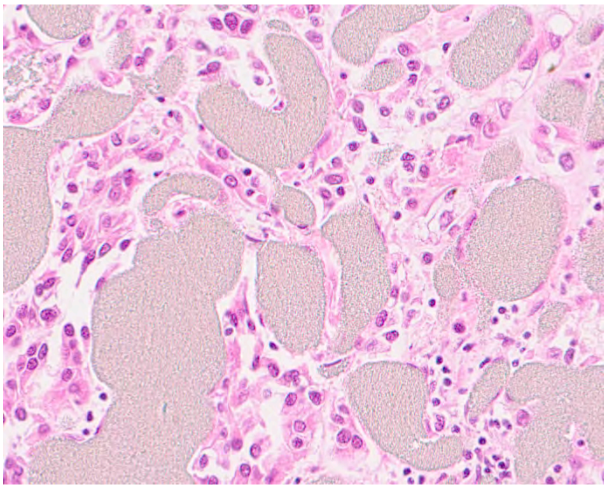

20:36 PRU 1.27

H21

Sex: Male
Age at surgery: 55 years
Survival from surgery: 0 year
Cause of death: Renal cancer
Initial stage: 8x8 cm pT2 N1
Tumour type: undifferentiated- embryonal sarcomatoid/rhabdoid
ISUP grade 4
Tumour volume: 83 cm^3

Specimen weight: 623 g
Perfusion pressure: 26 mmHg   Perfusate flow: 63 mL/min
Specimen PRU: 0.41
Cortical tissue PRU: NE. n=14
Tumour tissue PRU span: NE n=15
Reference withdrawal failed

Angiography

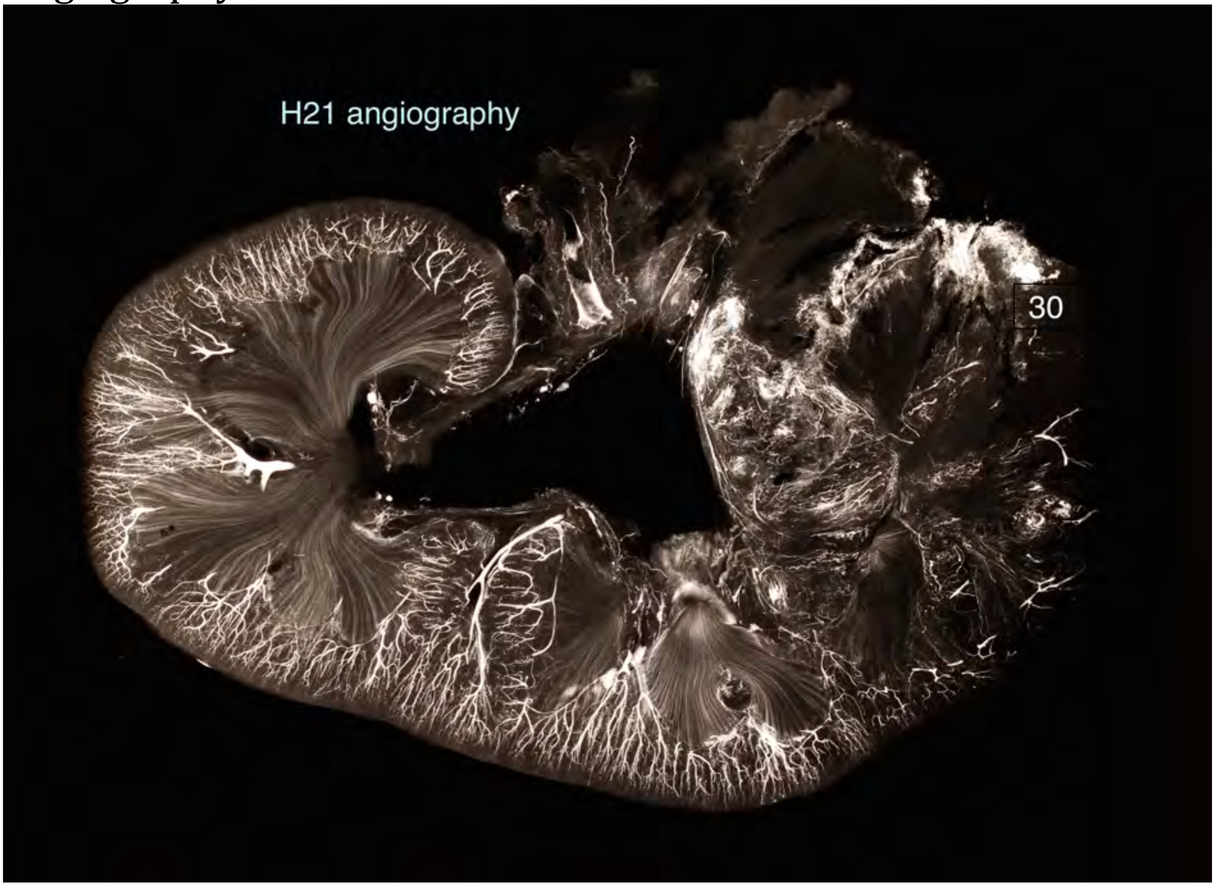

Scarersly vascularized with low 15 um sphere trapping

Autoradiography

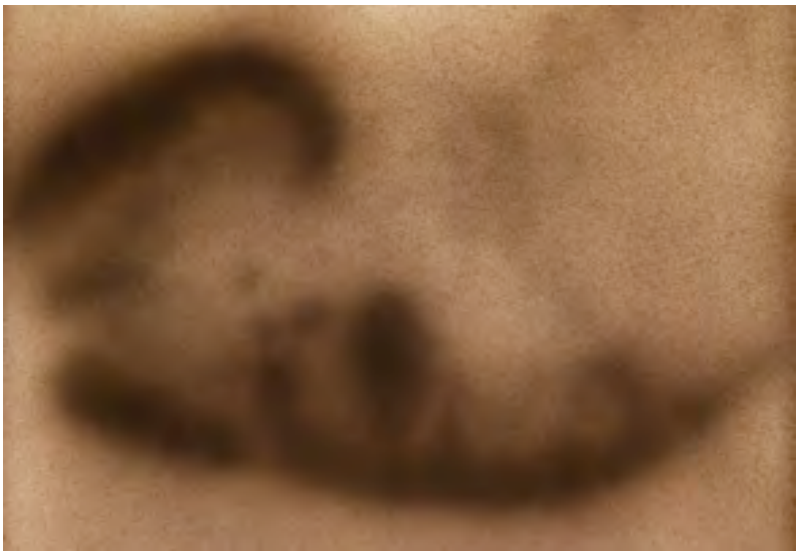

Darkfield macrophotography

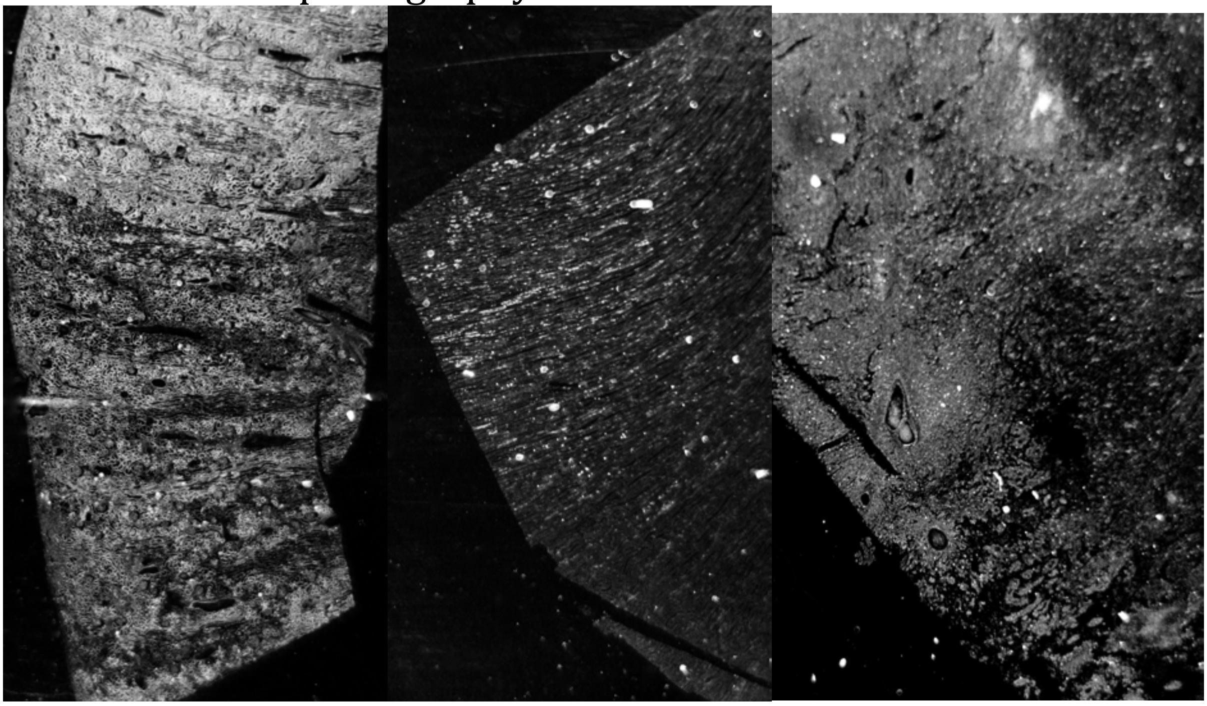

21:6 cortex 21:15 medulla 21:30 tumour

Darkfield CD31

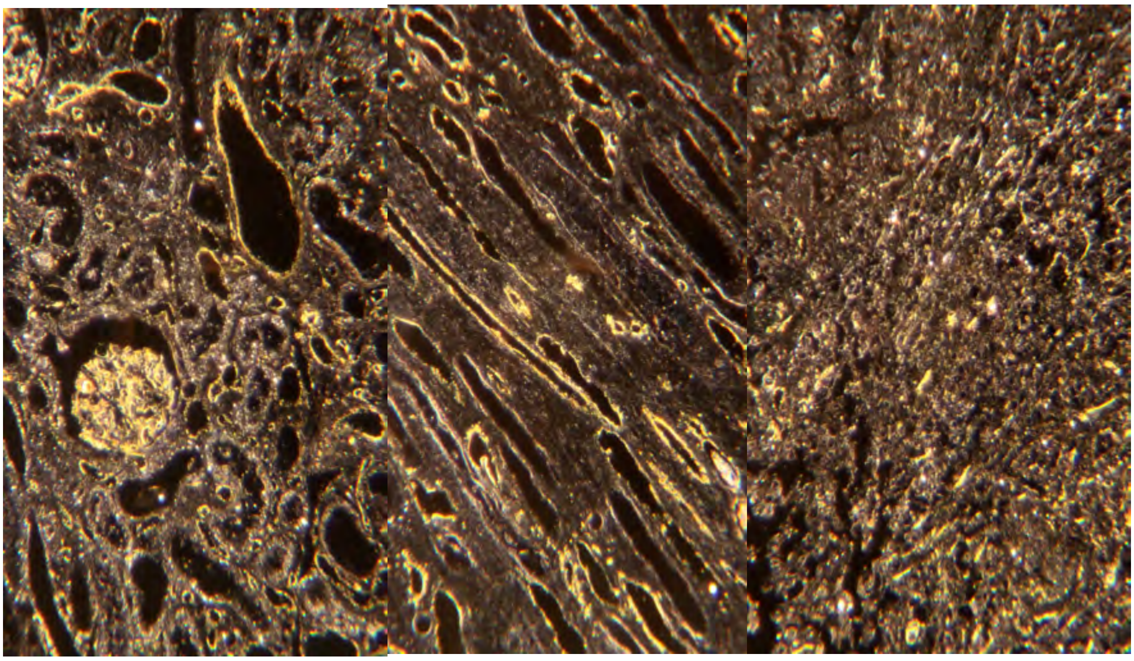

Cortex 21:6 PRUr 1 Medulla 21:15 Tumour 21:30 PRUr3.61

Tumour HE 16x

Brightfield Darkfield

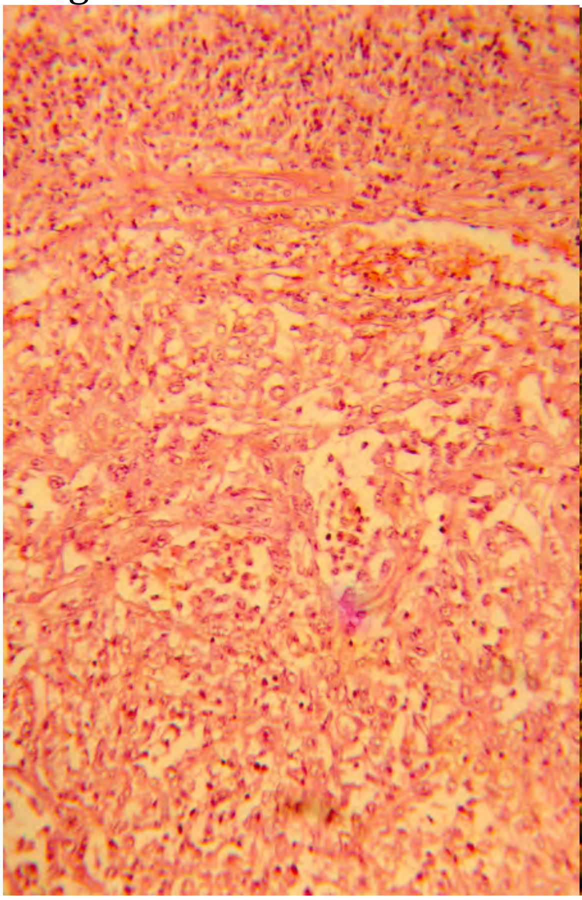
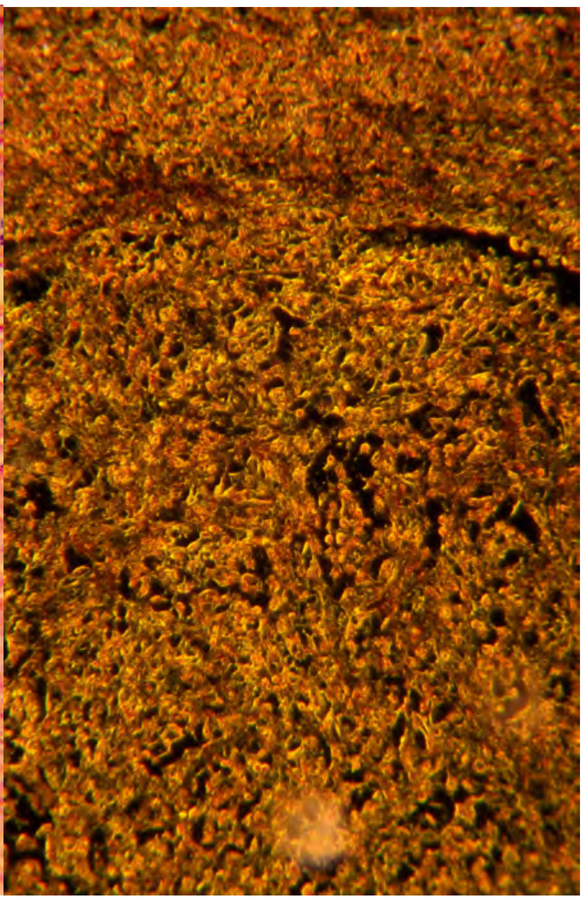

21:30 PRUrel 3.61

HE 20x

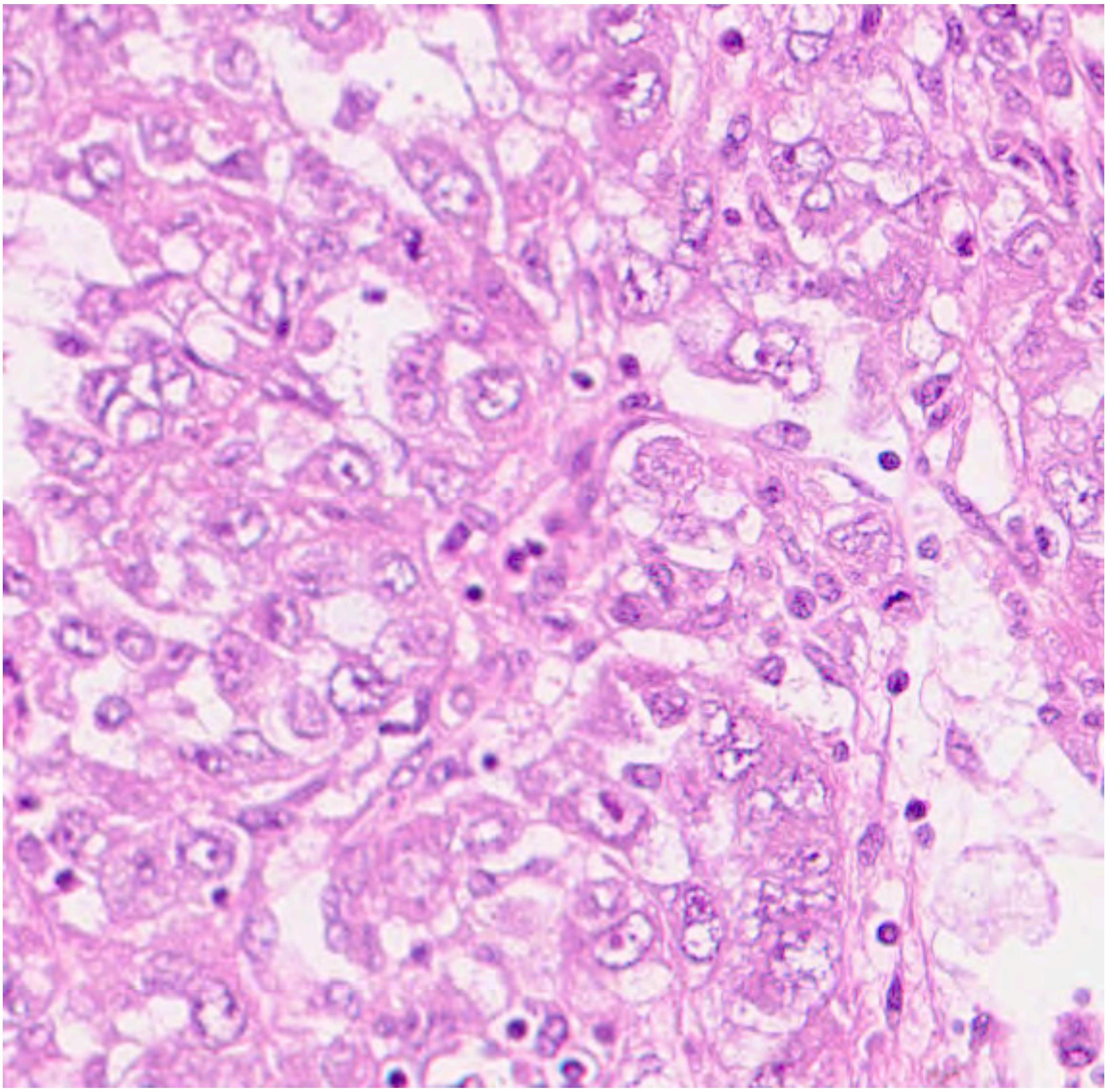

21:30 20x PRU 80

H22

Sex: Male
Age at surgery: 59 years
Survival from surgery: 10 years
Cause of death: other cause
Initial stage: 10x7 cm T6x4 cm pT2 NX M0
Tumour type: CCRCC ISUP grade 2
Tumour volume: 121 cm^3

Specimen weight: 865 g
Perfusion pressure: 43 mmHg Perfusate flow: 50 mL/min
Specimen PRU: 0.86
Cortical tissue PRU: 0.06 +/-0.02 n=7
Medullary tisue PRU: NE
Tumour tissue PRU span: 0.08-0.83 n=15
Angiography

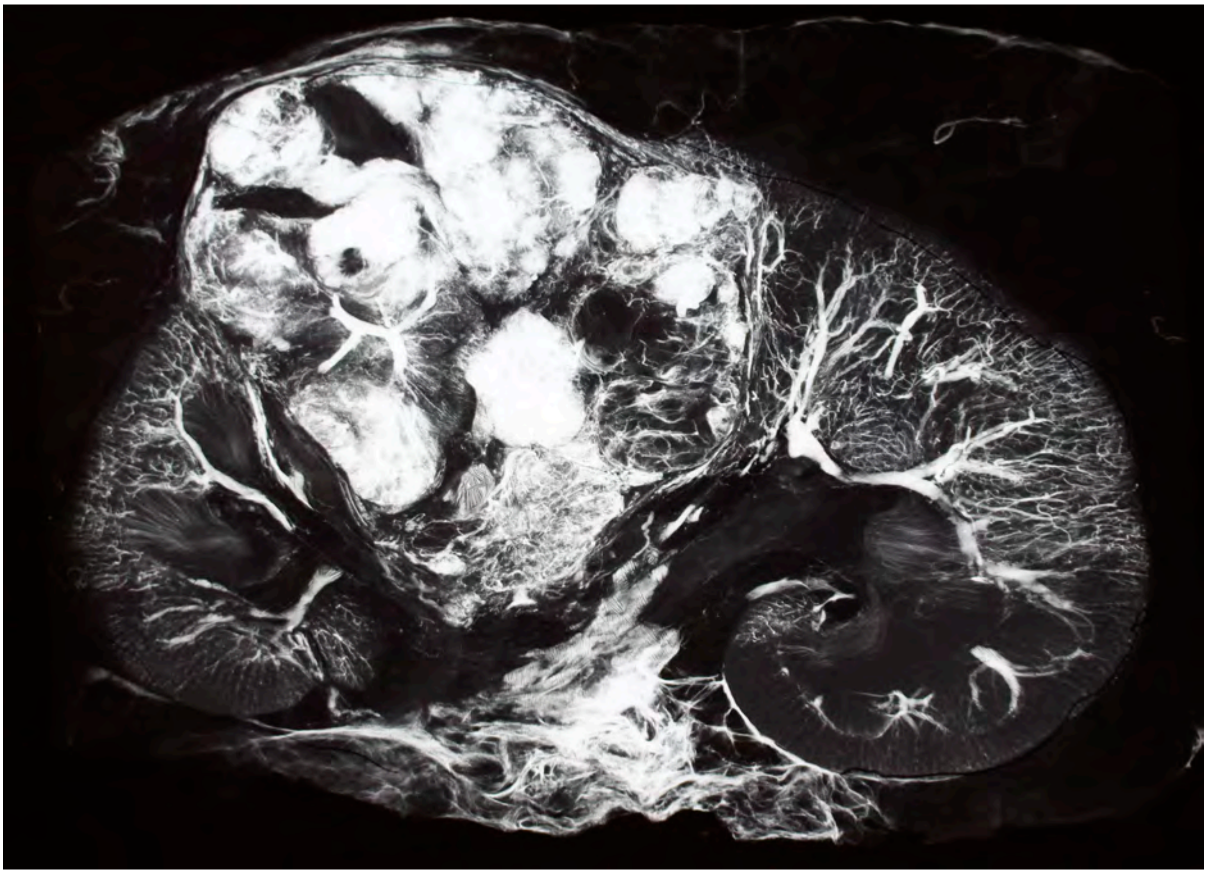

Lobular growth with intense vascularity with high 15 um sphere trapping

Autoradiography

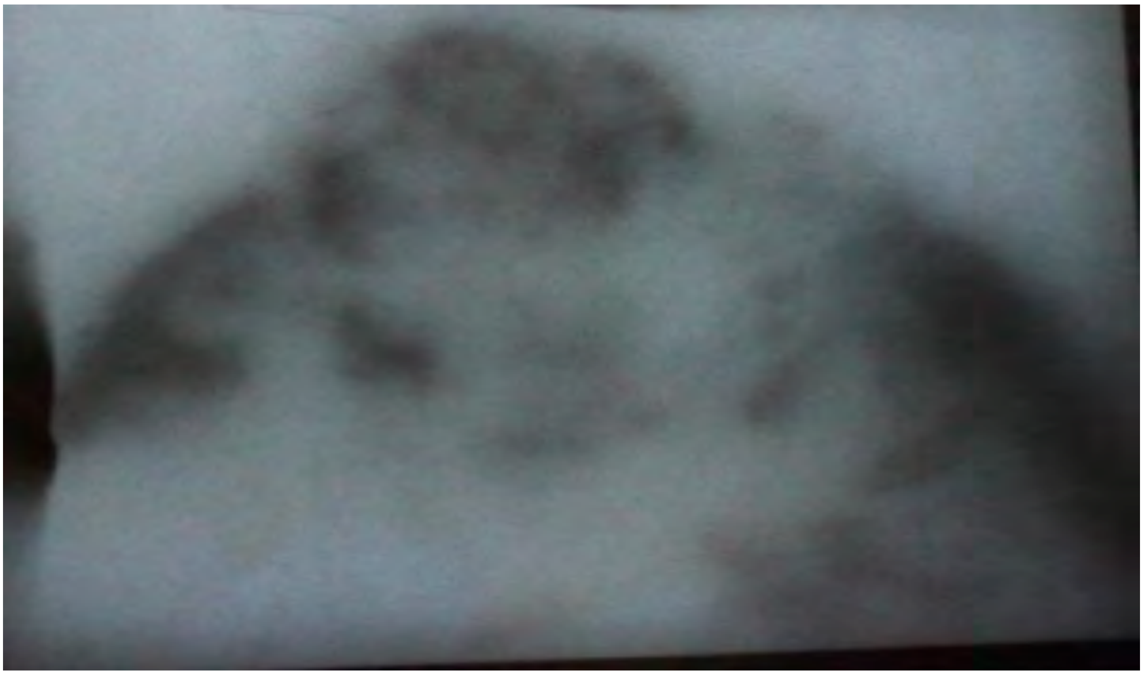

Darkfield macrophotography

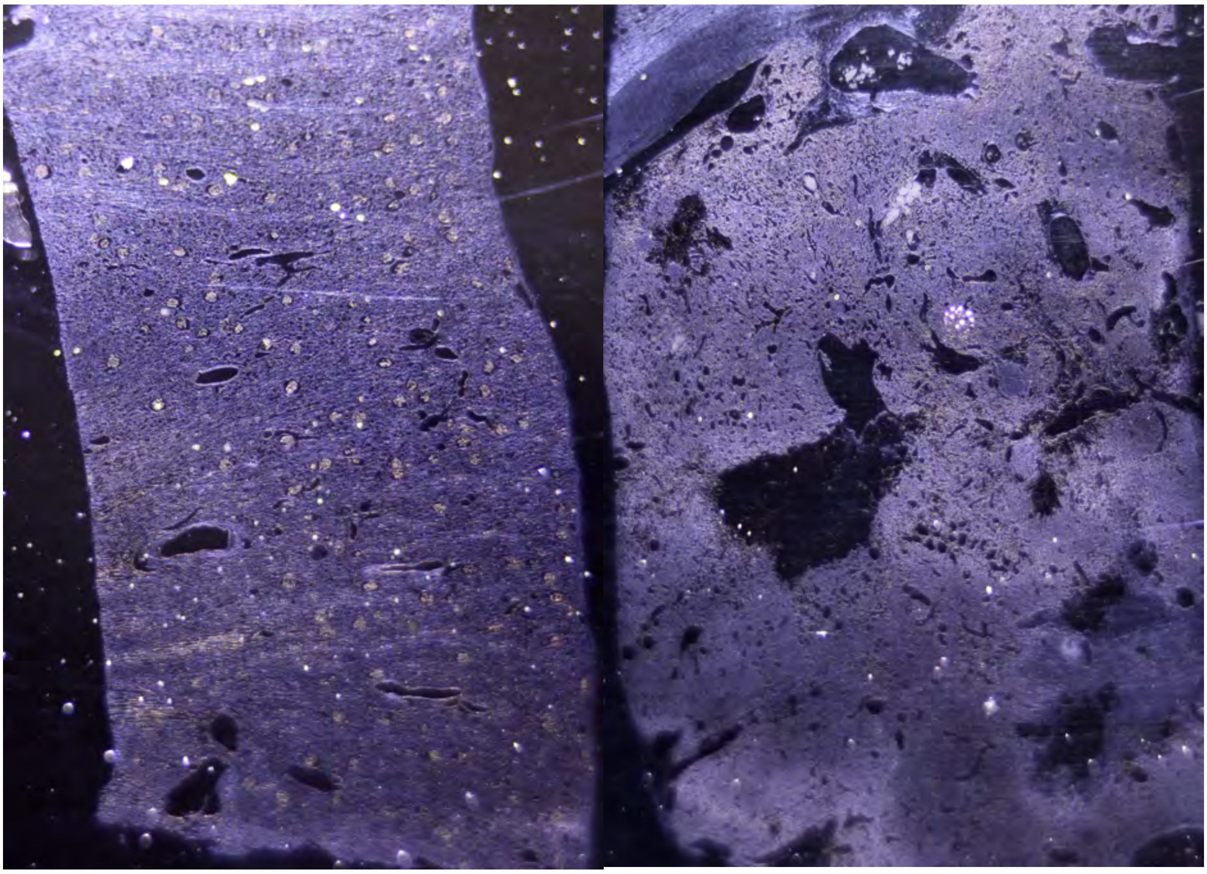

H22:5 cortex PRU 0.05 H22:14 tumour PRU 0.1

Darkfield CD31

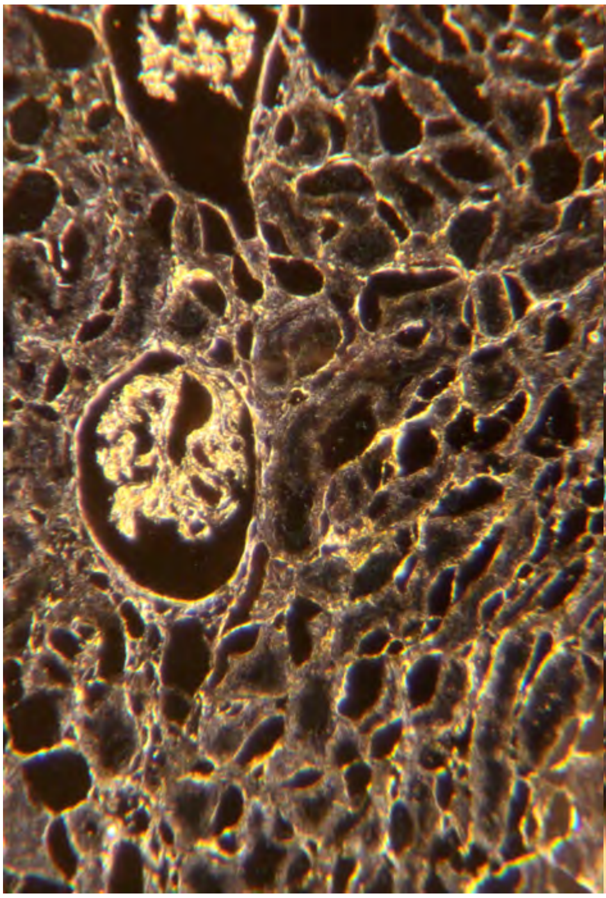

Cortex 22:5 PRU 0.05

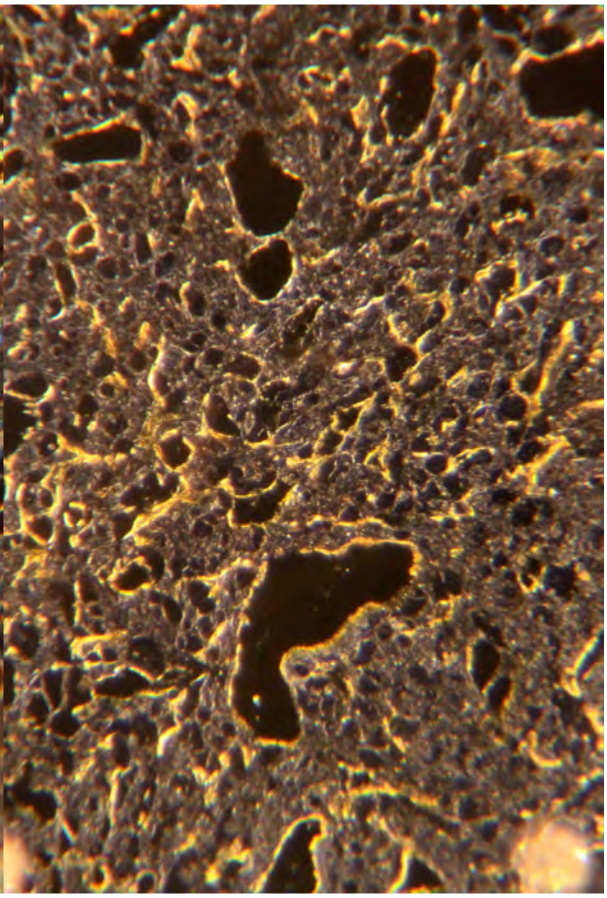

Tumour 22:14 PRU 0.10

Tumour HE stained 16x

Contrast

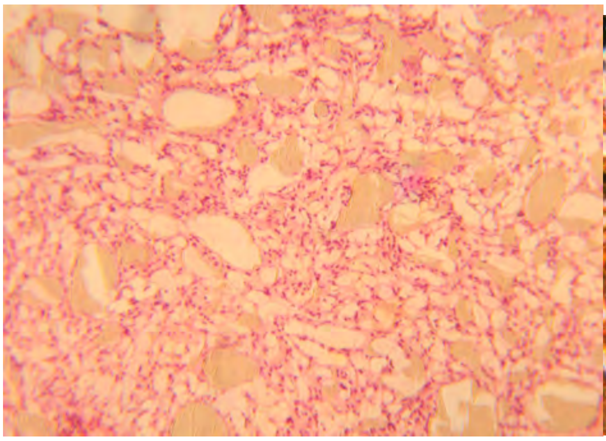

H22:14 PRU 0.10

Darkfield

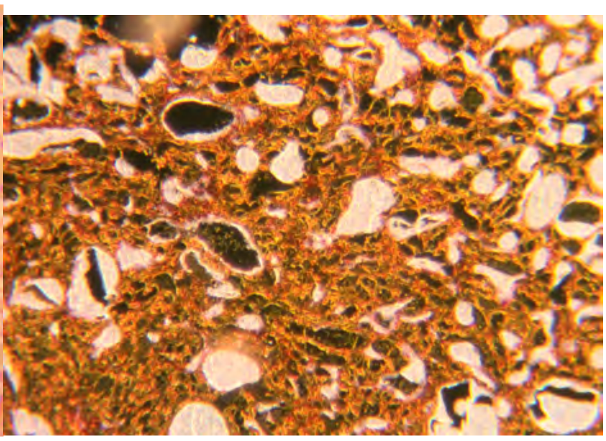

HE 20x

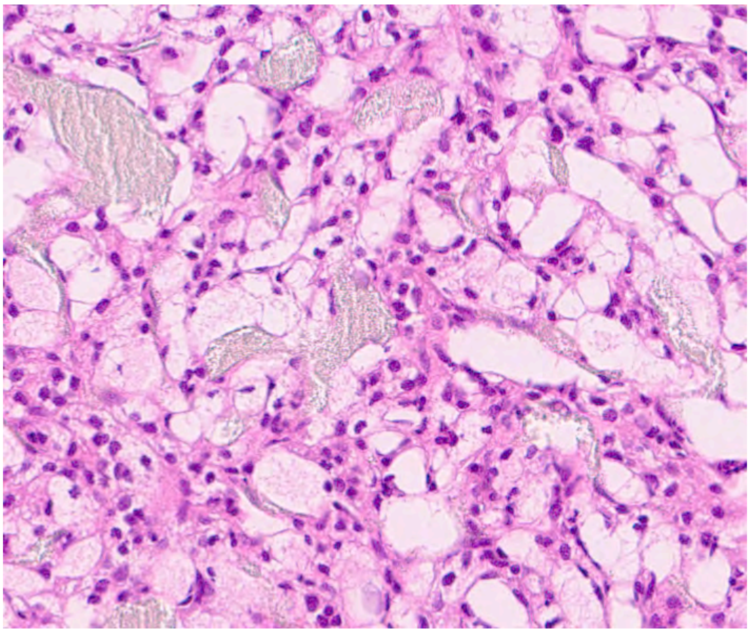

22:14 PRU 0.10

H23

Sex: Female
Age at surgery: 71years
Survival from surgery: 9 years
Cause of death: other cause
Initial stage: 7x7 cm T5 cm pT1b Nx M0
Tumour type: CCRCC & eosinophilic Tumour grade 3 (eosinophilic)
Tumour volume: 54 cm^3

Specimen weight: NE
Perfusion pressure: 38 mmHg   Perfusate flow: 47 mL/min
Specimen PRU: 0.81
Cortical tissue PRU: 0.09 +/- 0.02 n=6
Tumour tissue PRU span: 0.8-6.7   n=14

Angiography

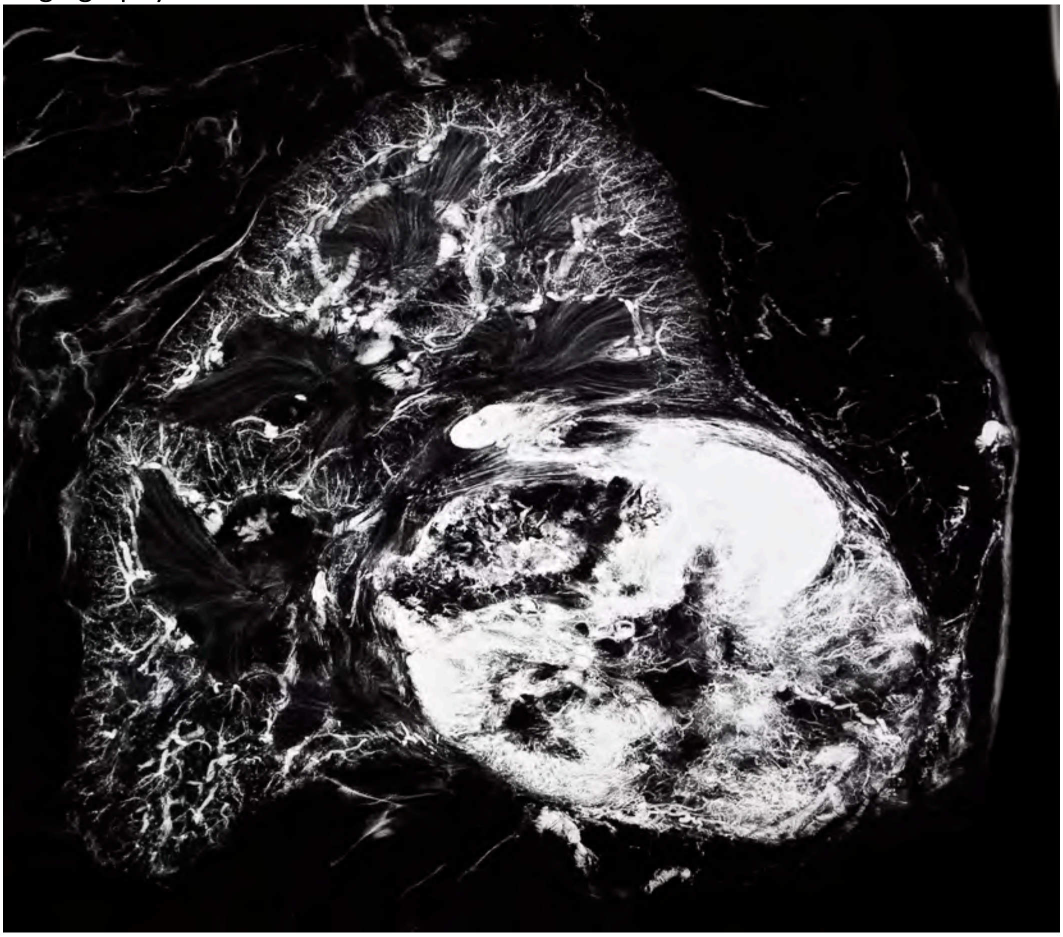

Heterogenous vascularity with poor 15 um sphere trapping

Autoradiography

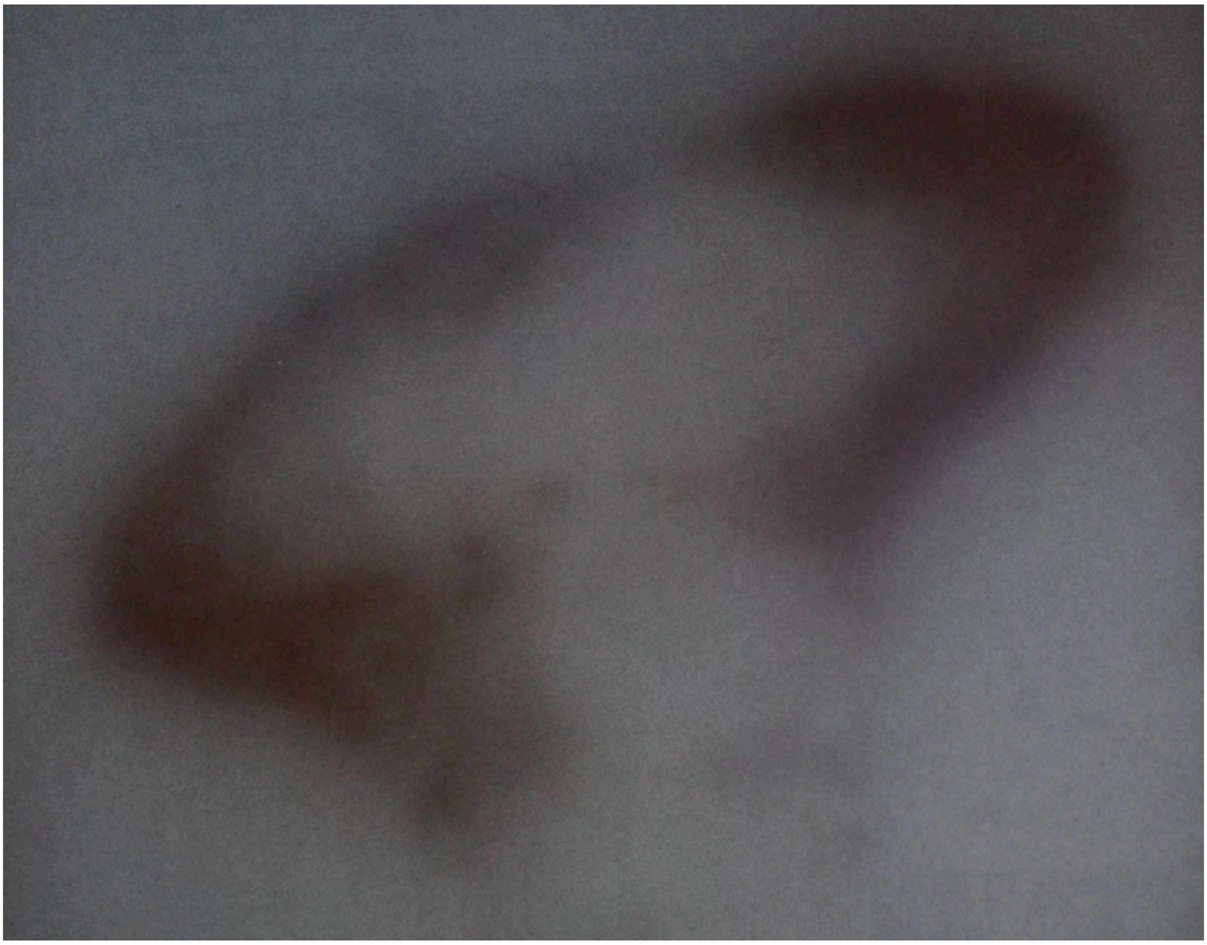

Dark-field macrophotography

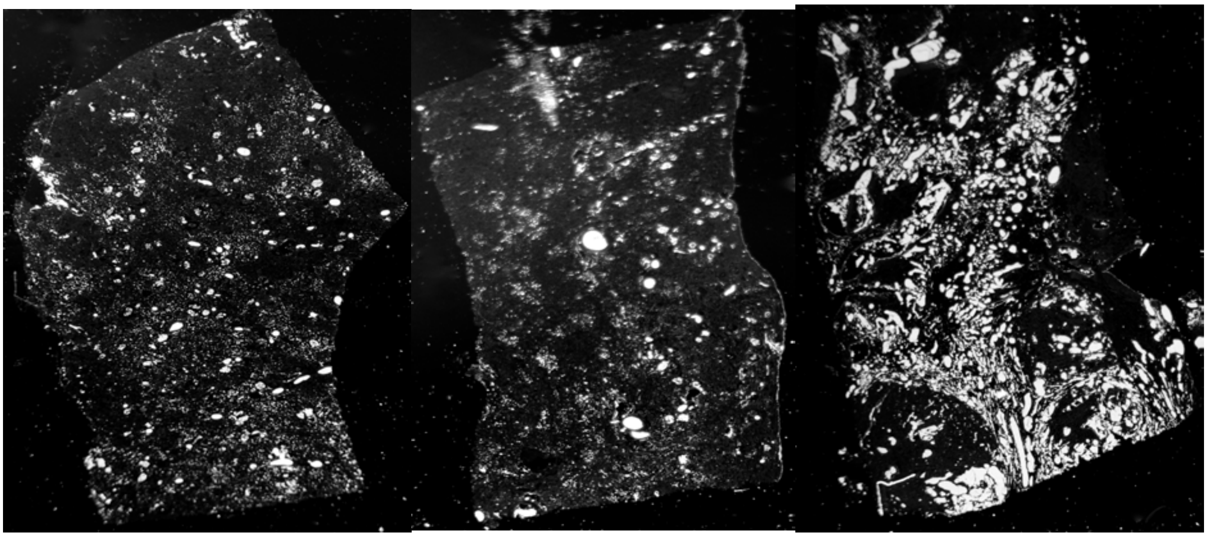

Cortex 23:3 PRU 0.07   Medulla 23:9 P 1.1   Tumour 23:20 P 2.9

Darkfield

Contrast CD31

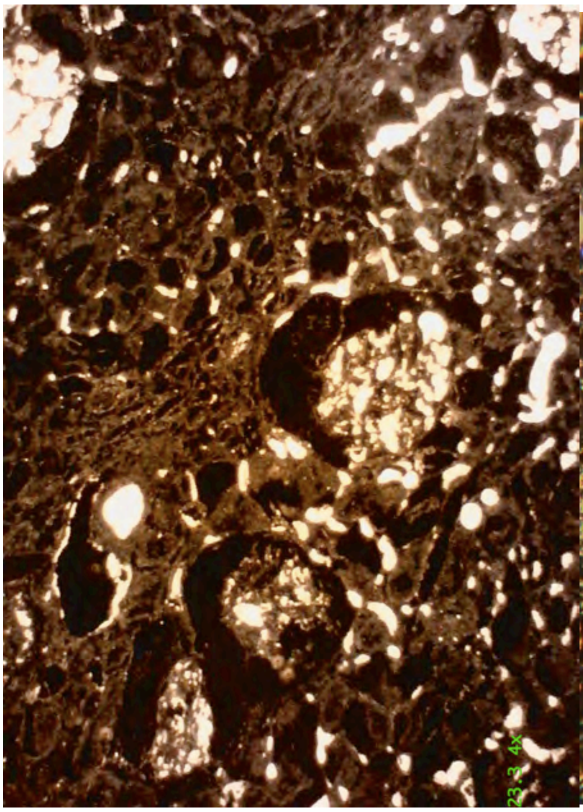

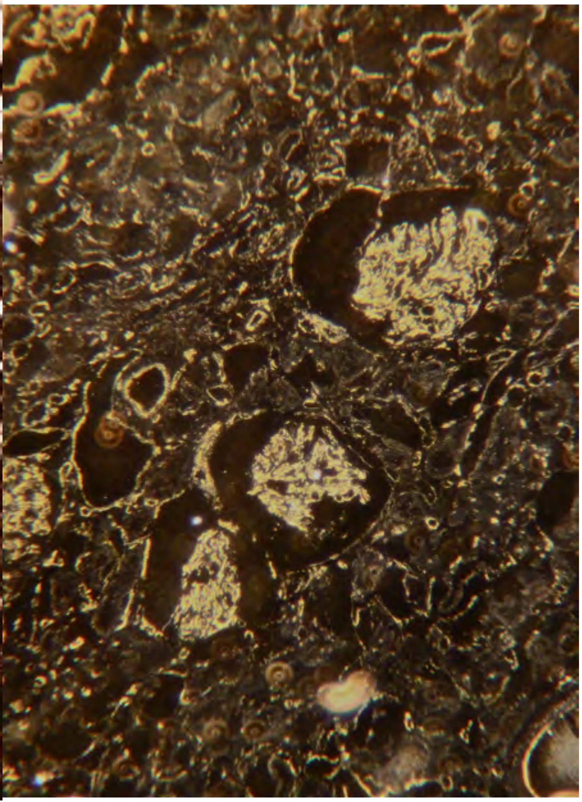

Cortex 23:3 PRU0.07

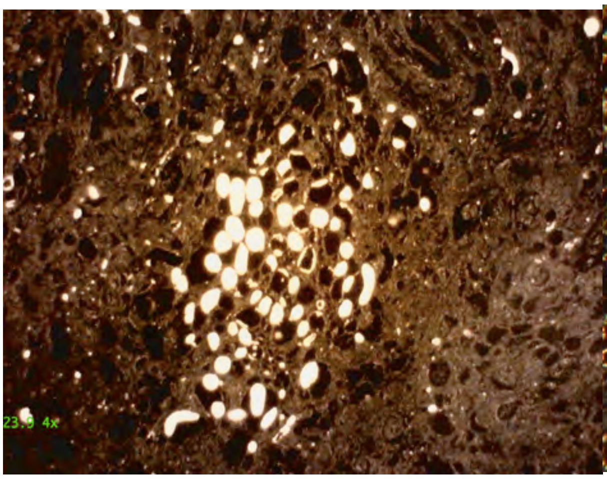

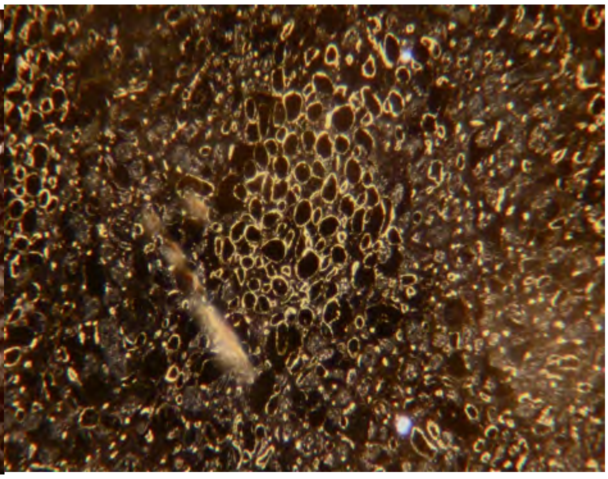

Medulla 23:9 PRU 1.1

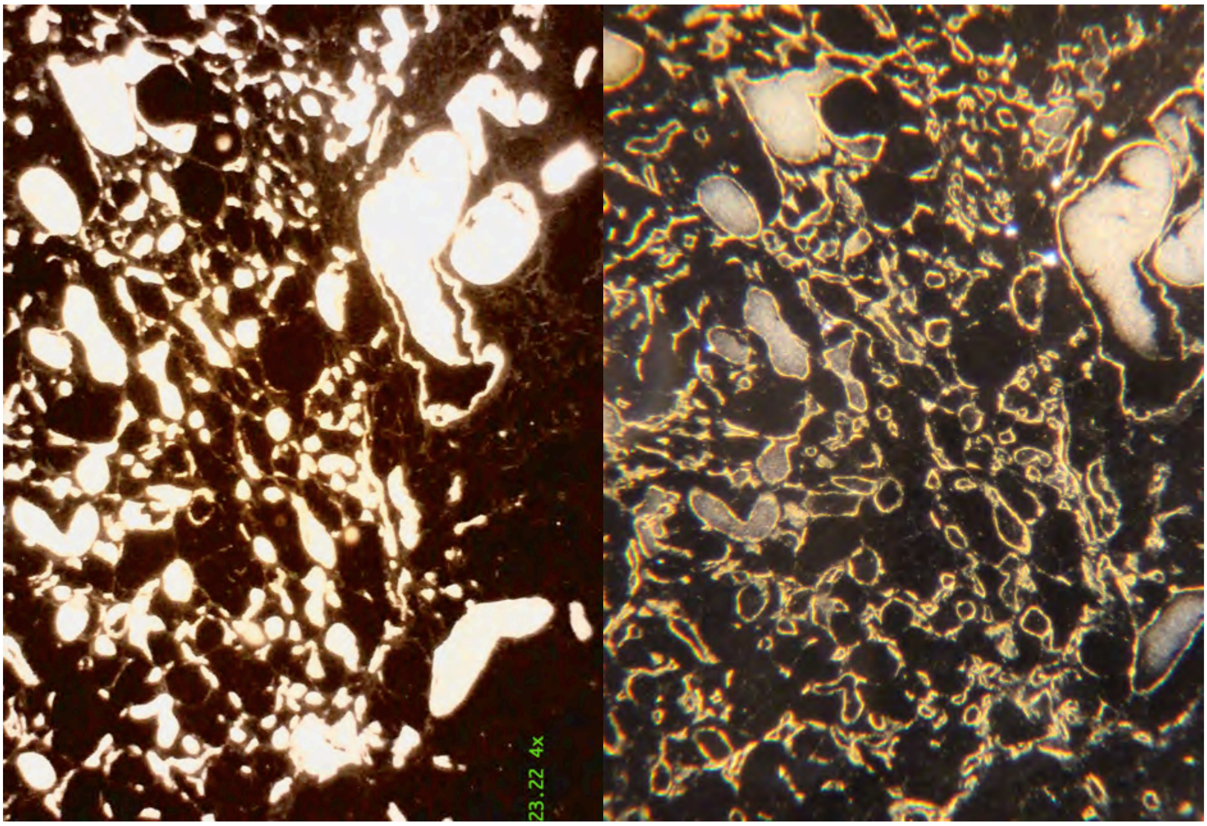

Tumour 23:22 PRU 1.8

Tumour sample HE stained

Bright field Darkfield

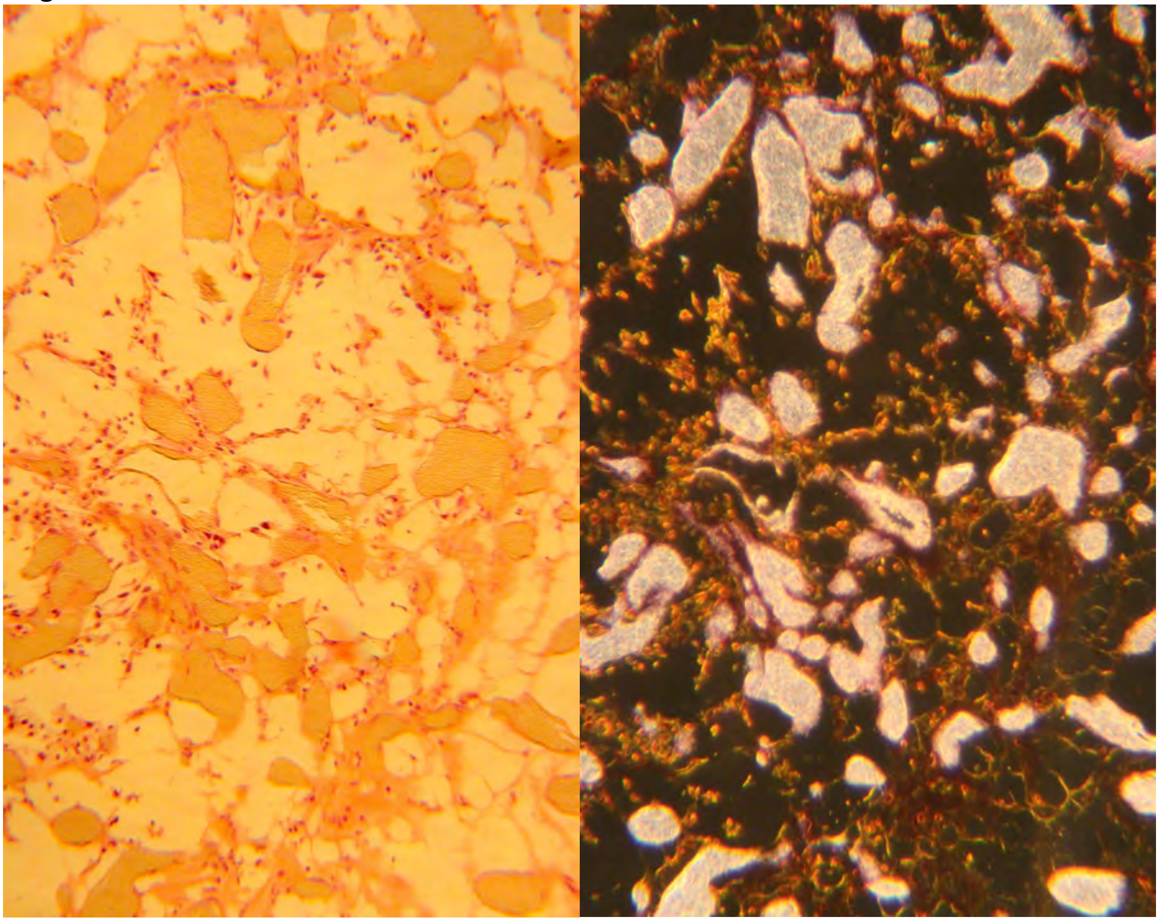

23:22 PRU 1.8

HE 20x

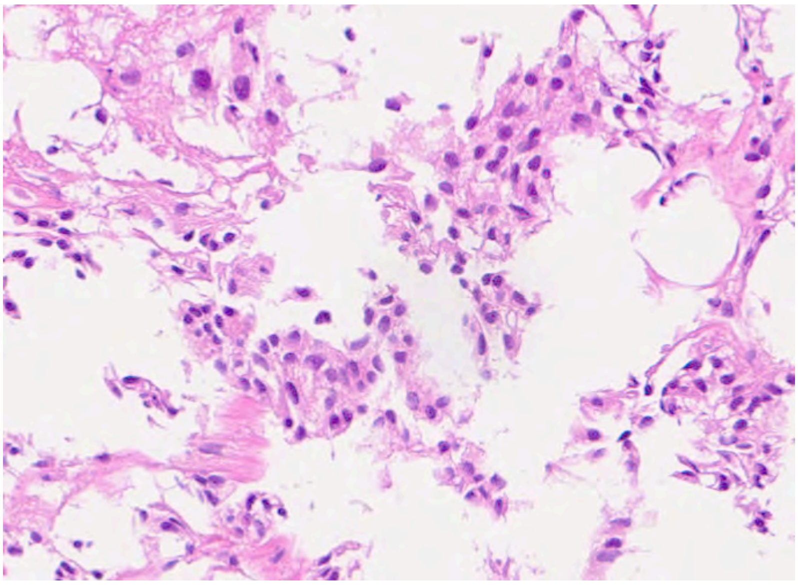

23:22 20x PRU1.8

H24

Sex: Male
Age at surgery: 67 years
Survival from surgery: 10 years
Cause of death: other cause
Initial stage: 8x8 cm T8x4 cm pT2 NX M0
Tumour type: CCRCC. ISUP grade 2
Tumour volume: 72 cm^3

Specimen weight: NE
Perfusion pressure: 24 mmHg Perfusate flow: NE
Specimen PRU: NE
Cortical tissue PRU: 0.13 +/-0.03 n=14
Tumour tissue PRU span:0.71-1.48 n=11

Angiography

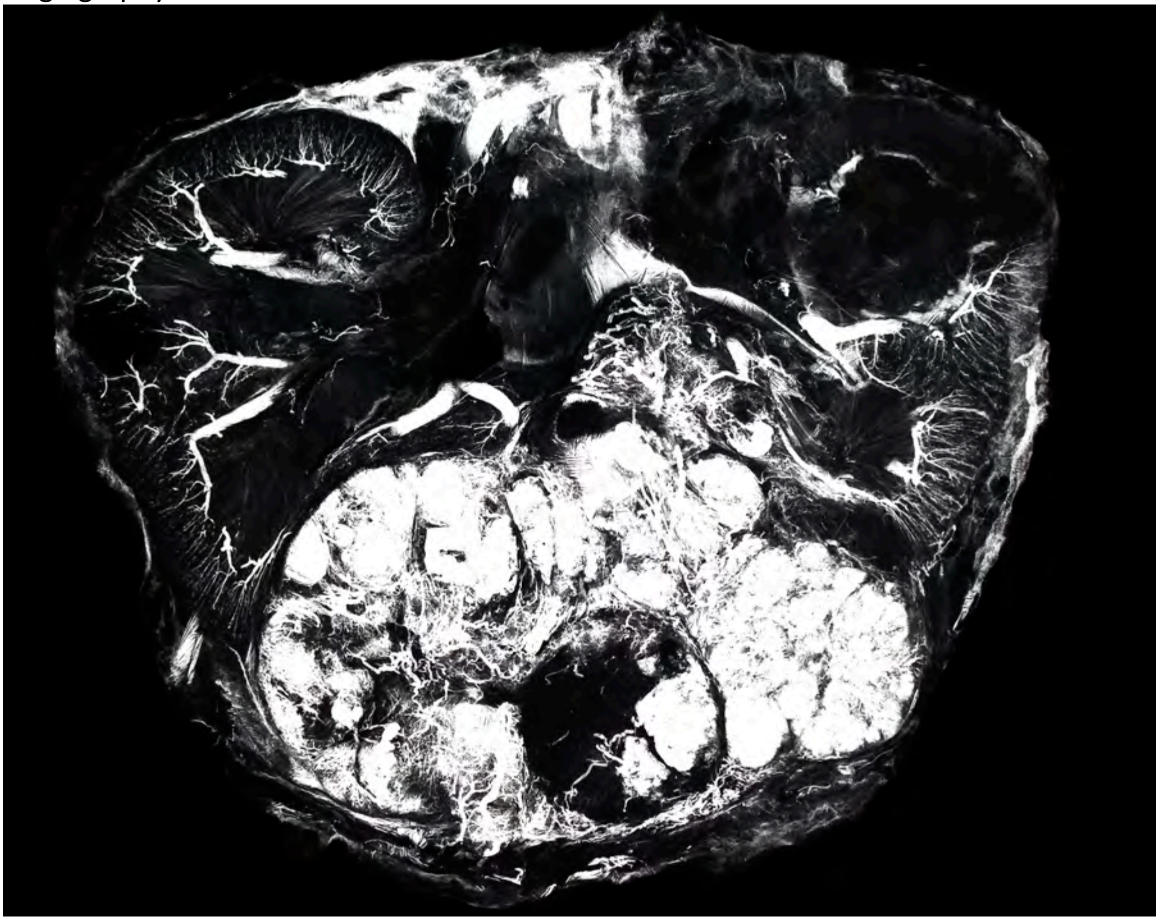

Lobular growth with rich vascularity and heterogenous 15 um sphere trapping

Autoradiography

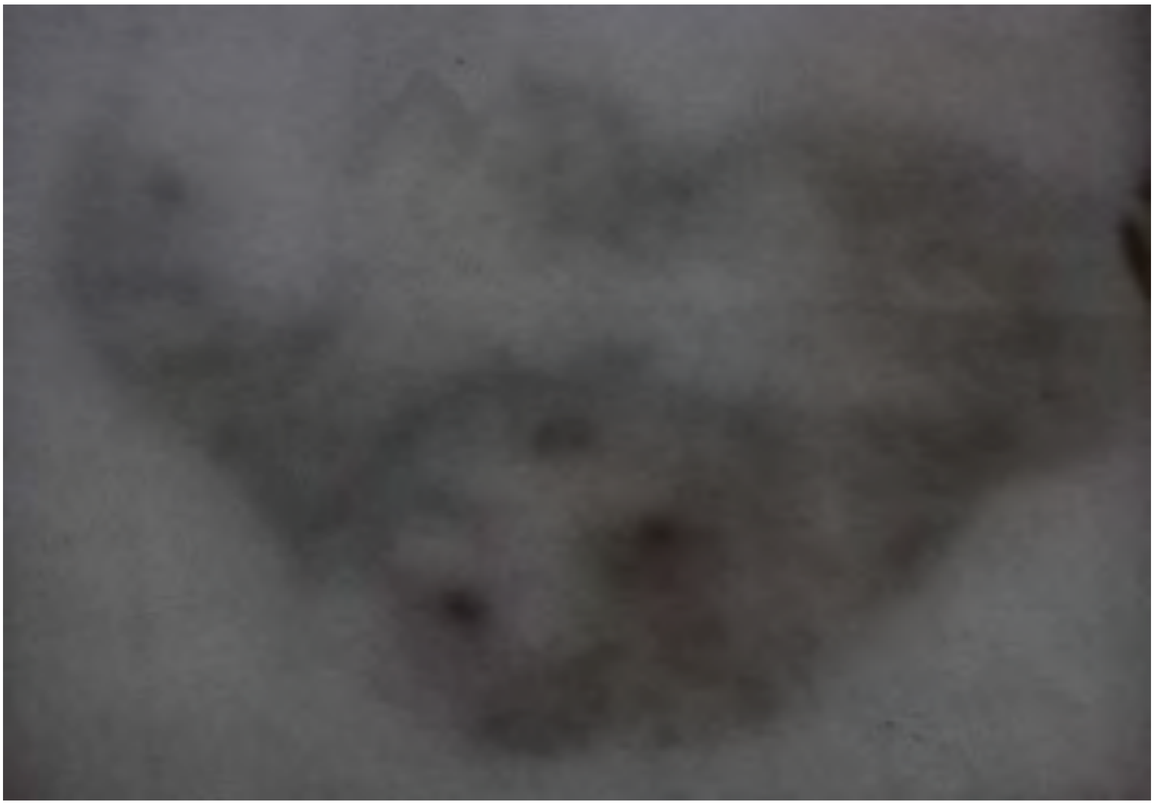

Dark-field macrophotography

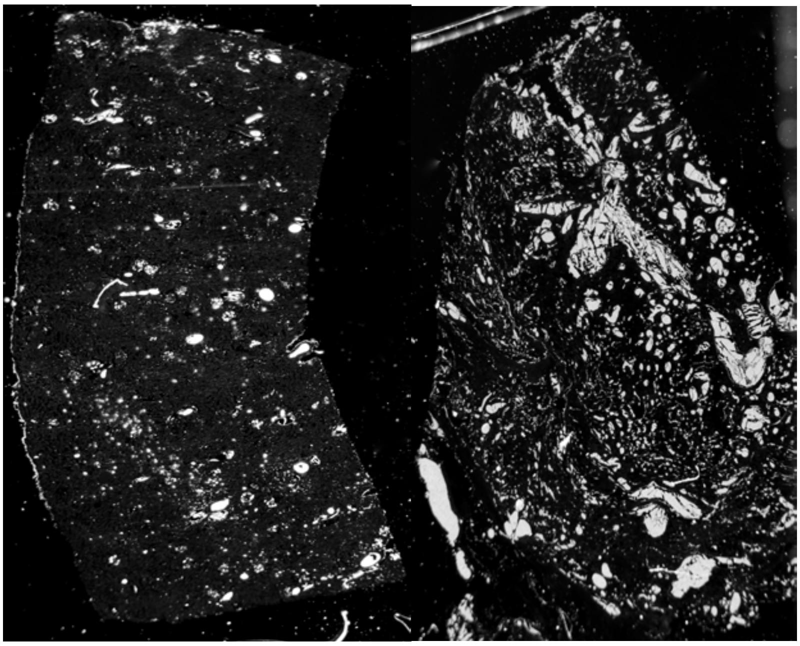

Cortex 24:3 PRU 0.11 Tumour 24:27 PRU 1.48

Darkfield
Contrast CD31

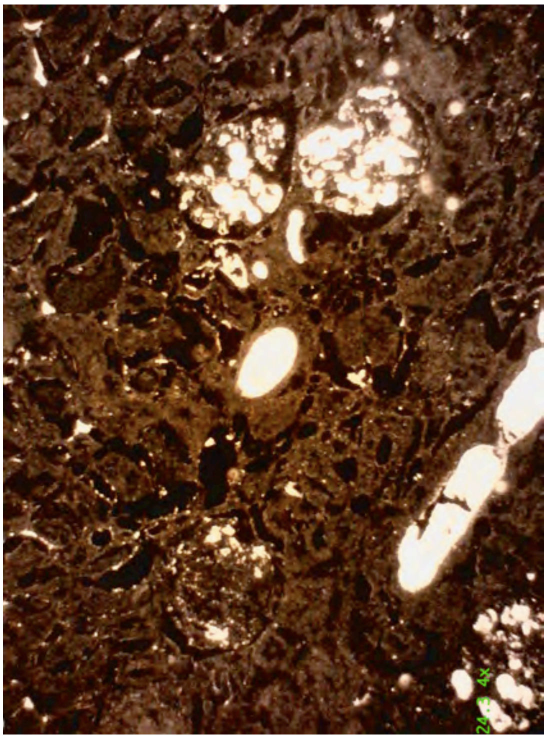

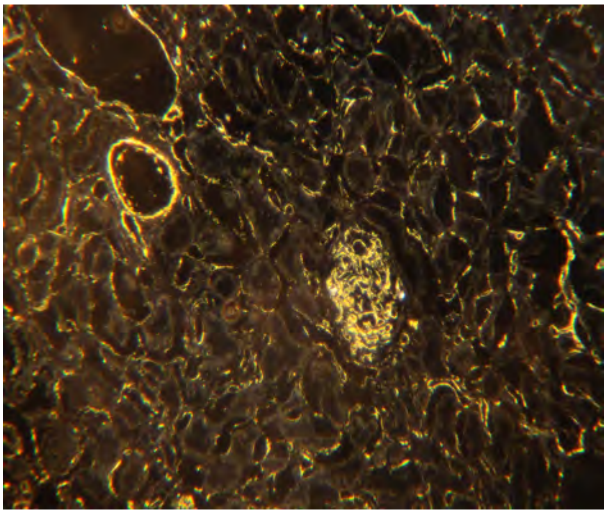

cortex 24:3 PRU 32-3

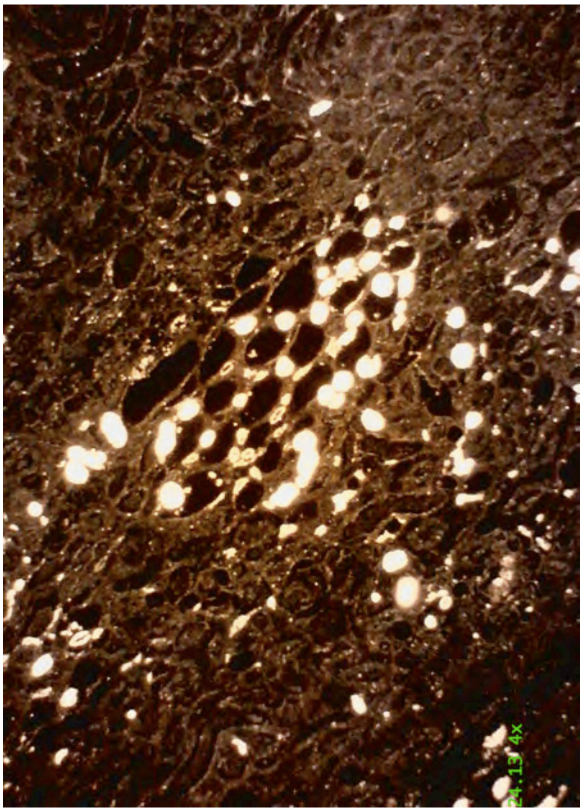

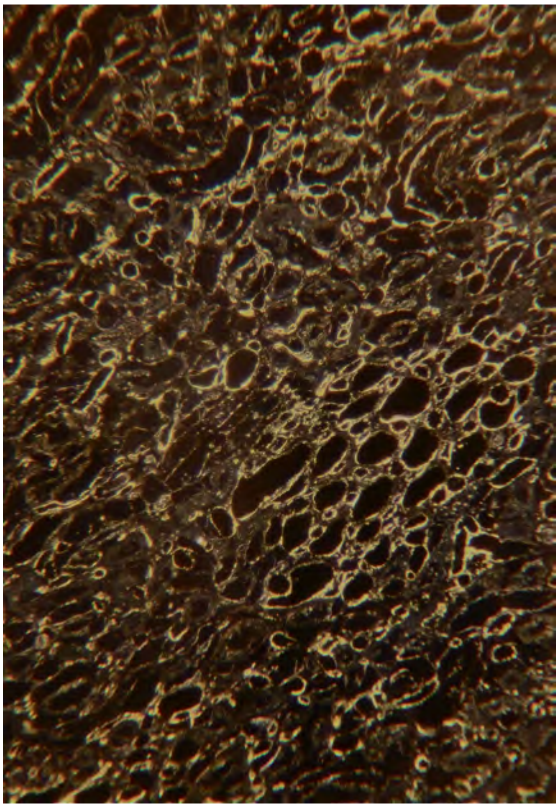

Medulla 24:13 PRU 0.14

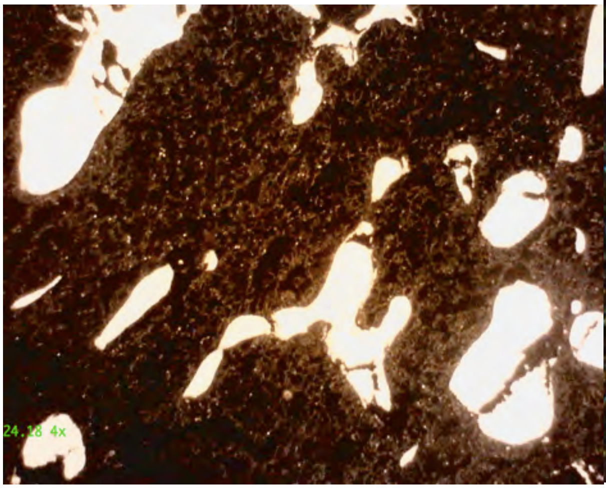

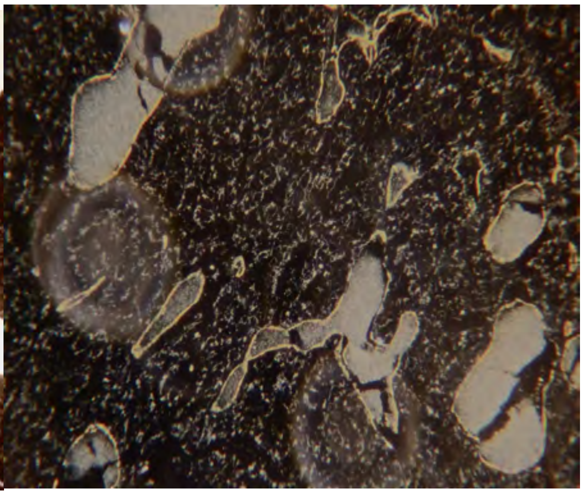

Tumour 24:18 PRU 1.18

Tumour sample HE stained 16x

Bright field Darkfield

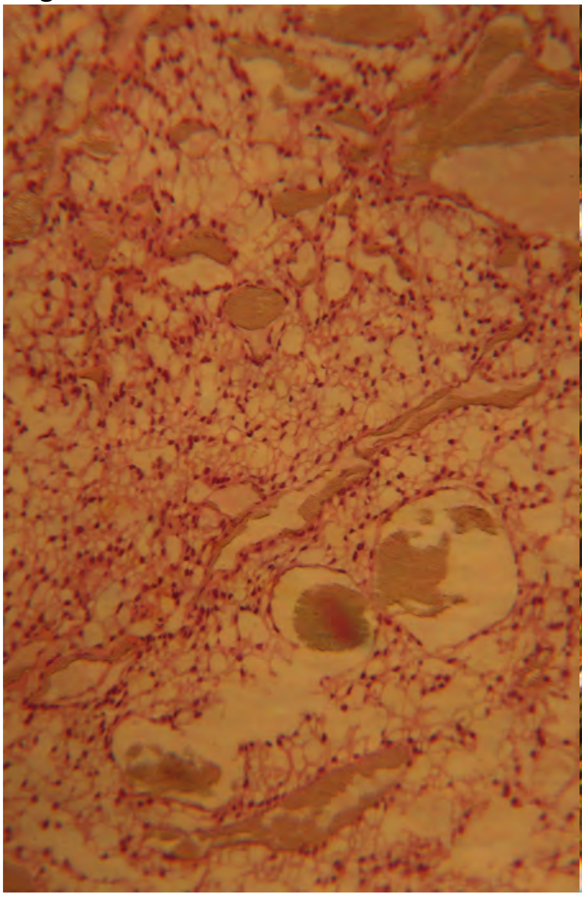
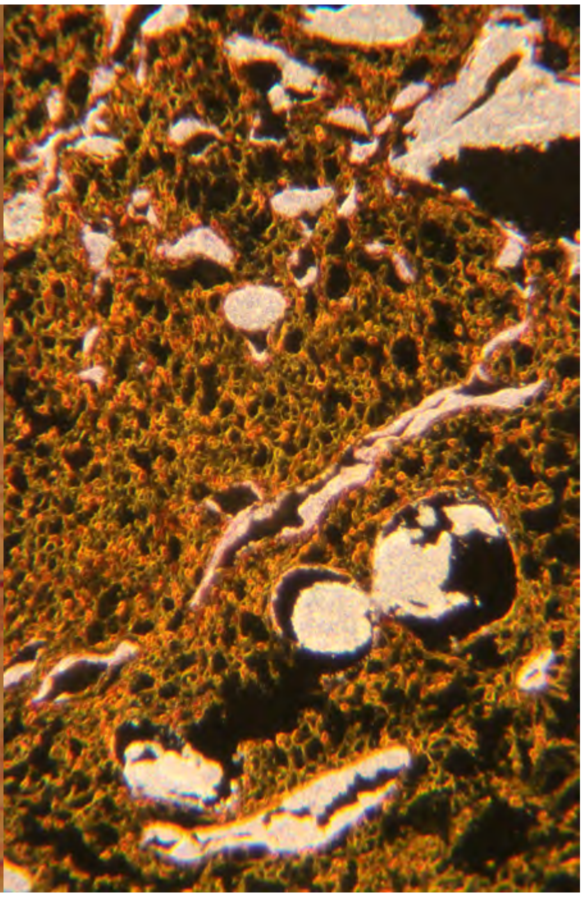

24:27 PRU 1.48

HE 20x

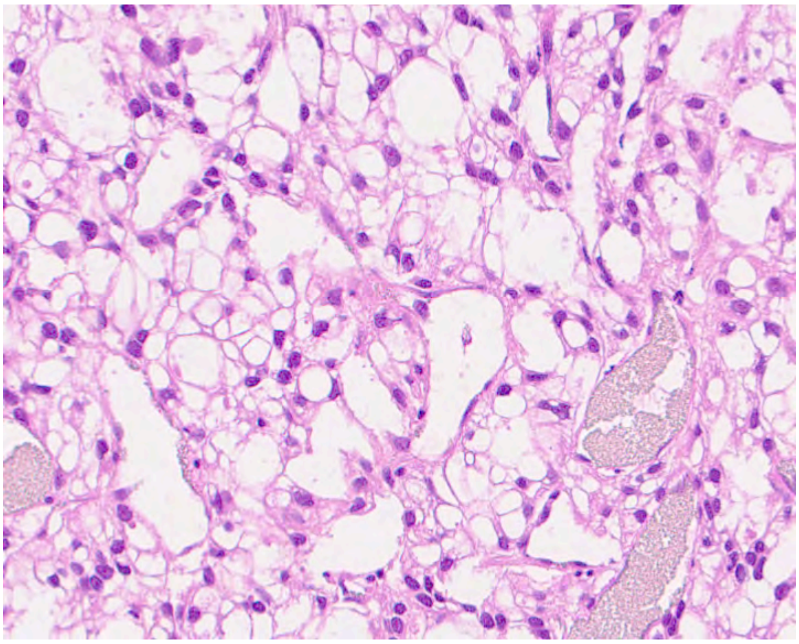

24:27 20x PRU 1.48

H25

Sex: Male
Age at surgery: 70 years
Survival from surgery:18 years
Cause of death: other cause
Initial stage: 2.5x2.5 cm pT1a NX M0
Tumour type: Oncocytoma ISUP grade NE
Tumour volume: 8 cm^3

Specimen weight: 432 g
Perfusion pressure: 40 mmHg Perfusate flow:70 mL/min
Specimen PRU: 0.57
Cortical tissue PRU: NE n=9
Tumour tissue PRU span: NE n=5

Reference withdrawal failed

Angiography

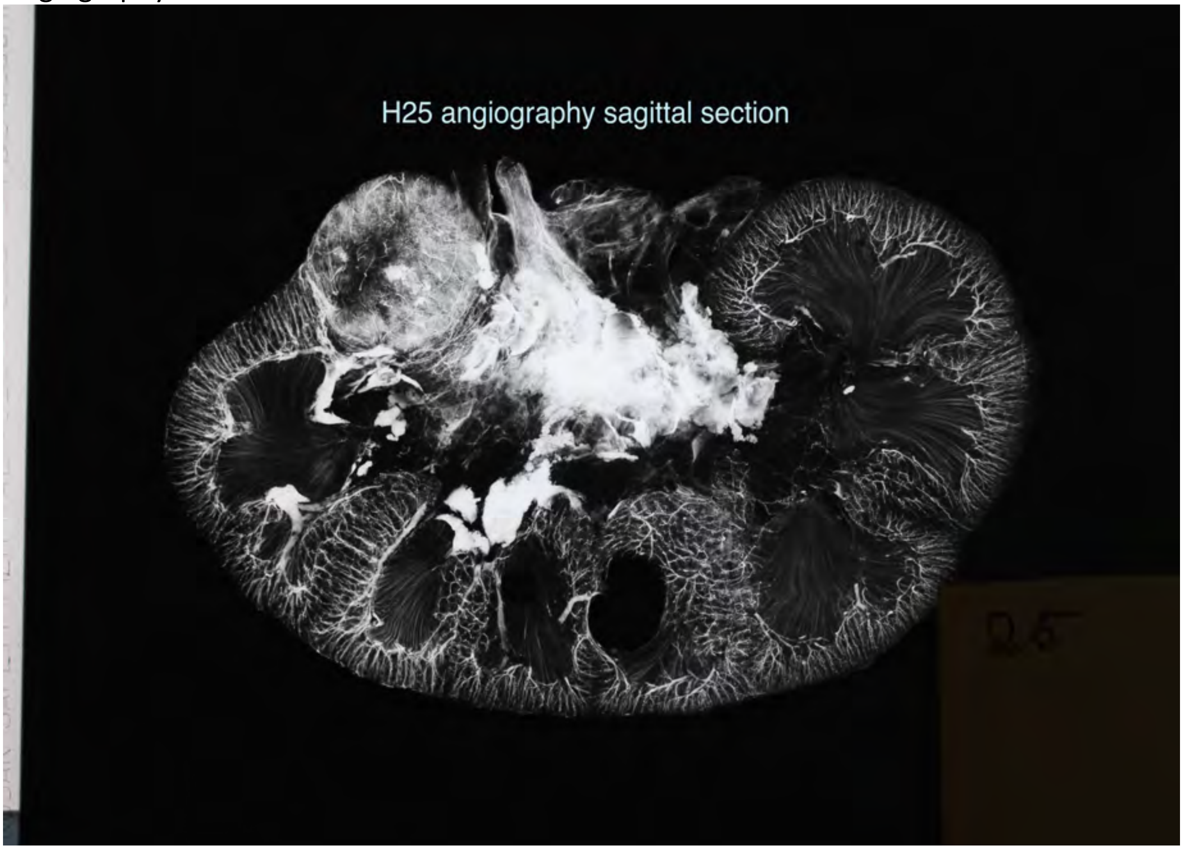

Periphery richly vascularized, but poor trapping of 15 um spheres.

Autoradiography

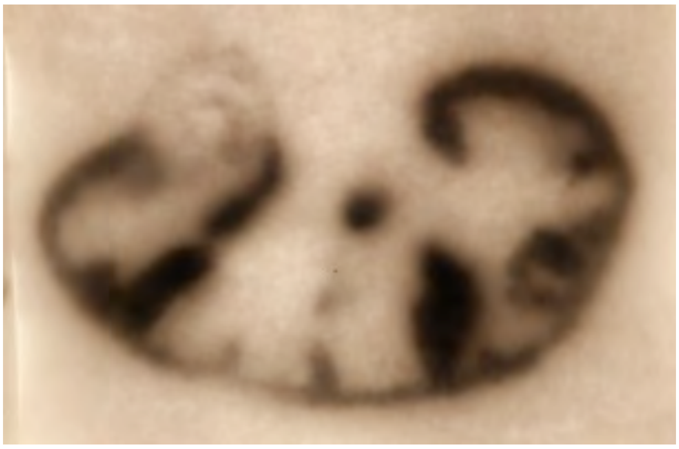

Darkfield CD31

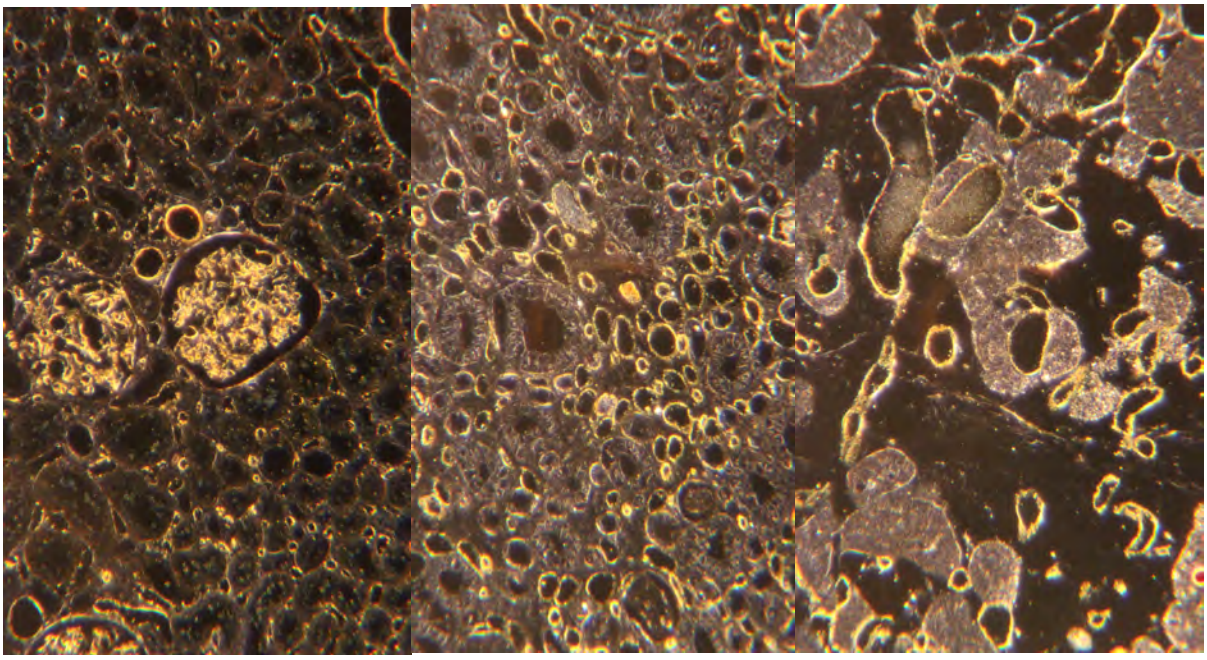

Cortex 25:1 PRU_{rel} 1 Medulla 25:10 Tumour 25:11 PRU_{rel} 3.87

Tumour HE 16x

Bright field

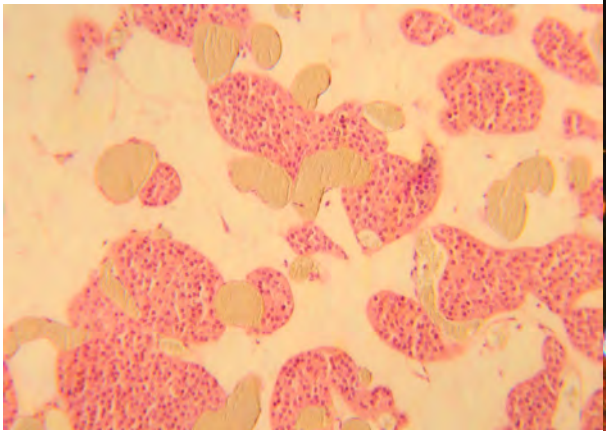

Darkfield

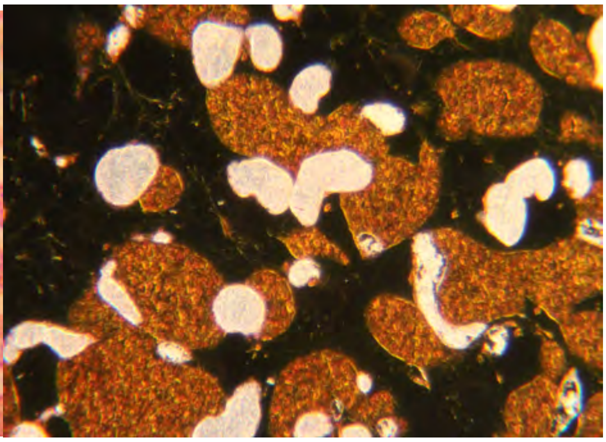

25:11 Oncocytoma PRU_{rel} 3.87

HE 20x

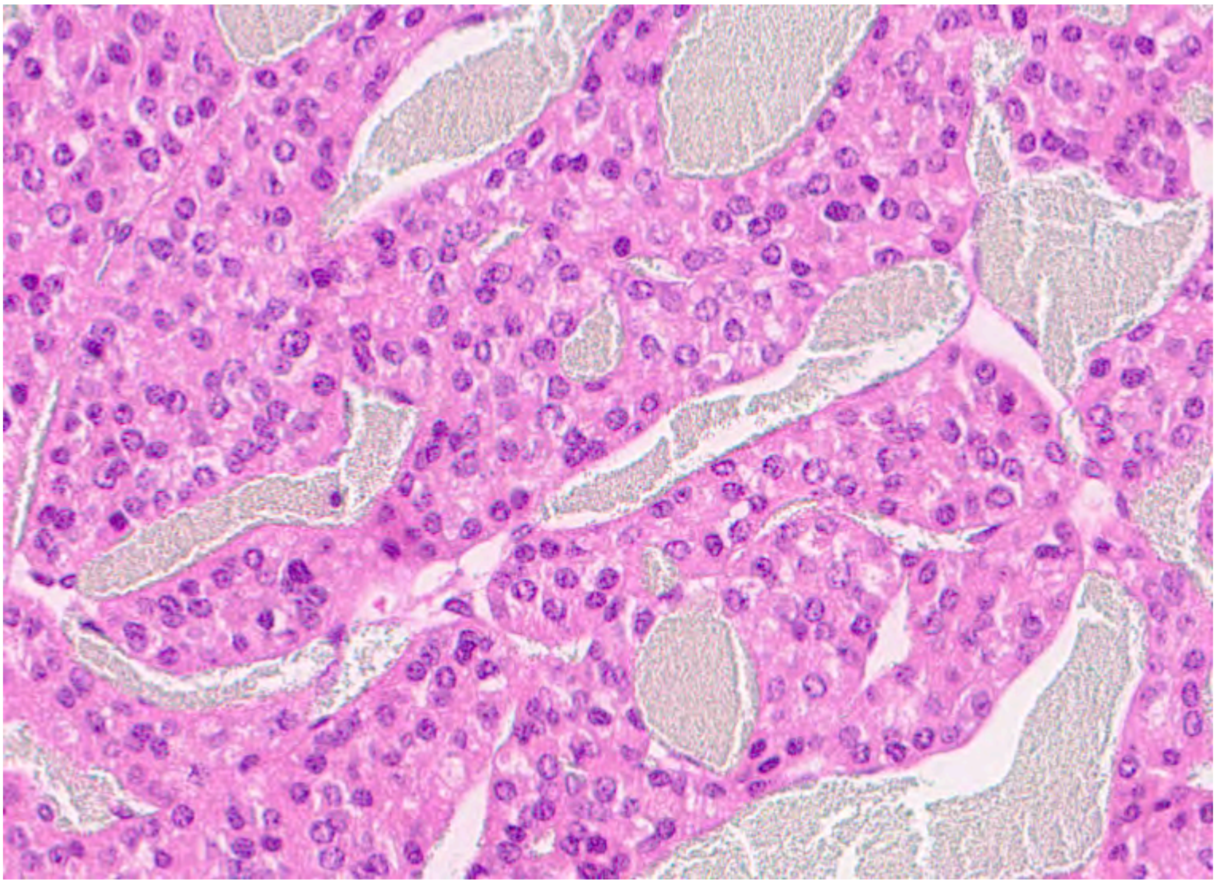

25:11 20x PRUrel 3.87

H26

Sex: Female
Age at surgery: 64 years
Survival from surgery: 9 years
Cause of death: Renal cancer
Initial stage: 5 cm pT1b N0
Tumour type: CCRCC. ISUP grade 2
Tumour volume: 17 cm^3

Specimen weight: 390 g
Perfusion pressure: 37 mmHg Perfusate flow: 39 mL/min
Specimen PRU: 0.95
Cortical tissue PRU:0.15 +/-0.02 n=12
Tumour tissue PRU span: 0.63-8.41 n=14

Angiography

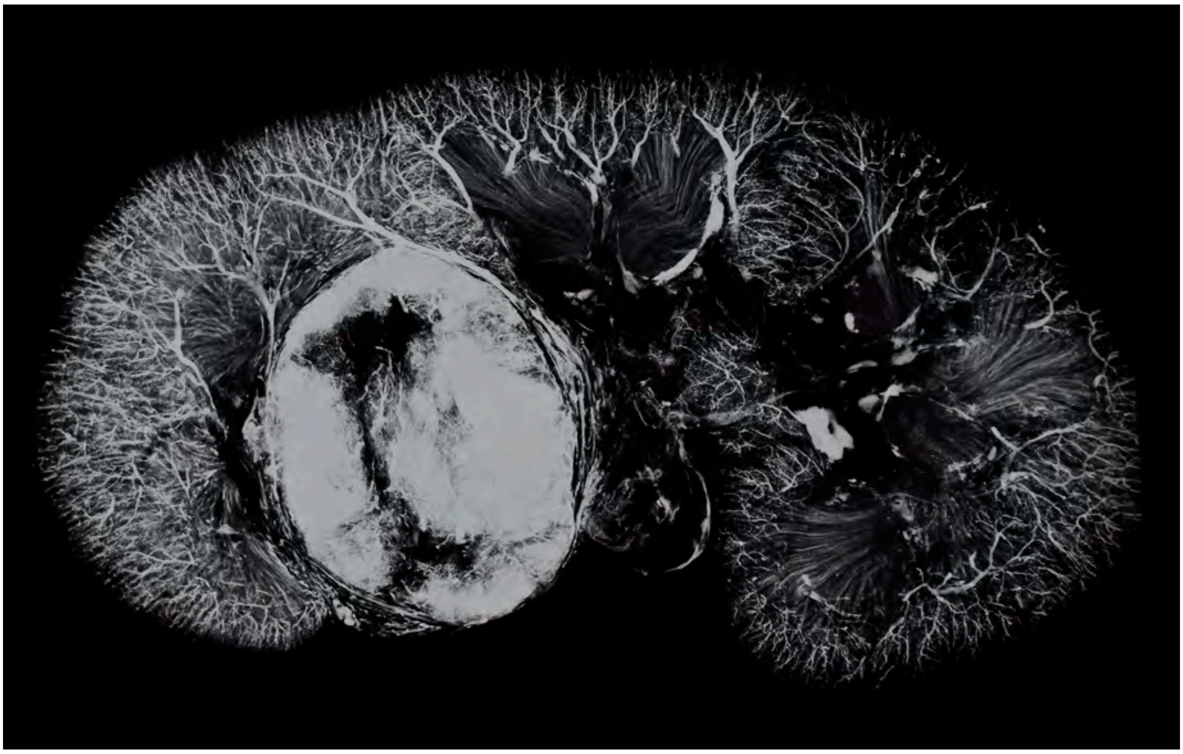

Periphery richly vascularized with poor 15 um sphere trapping

Autoradiography

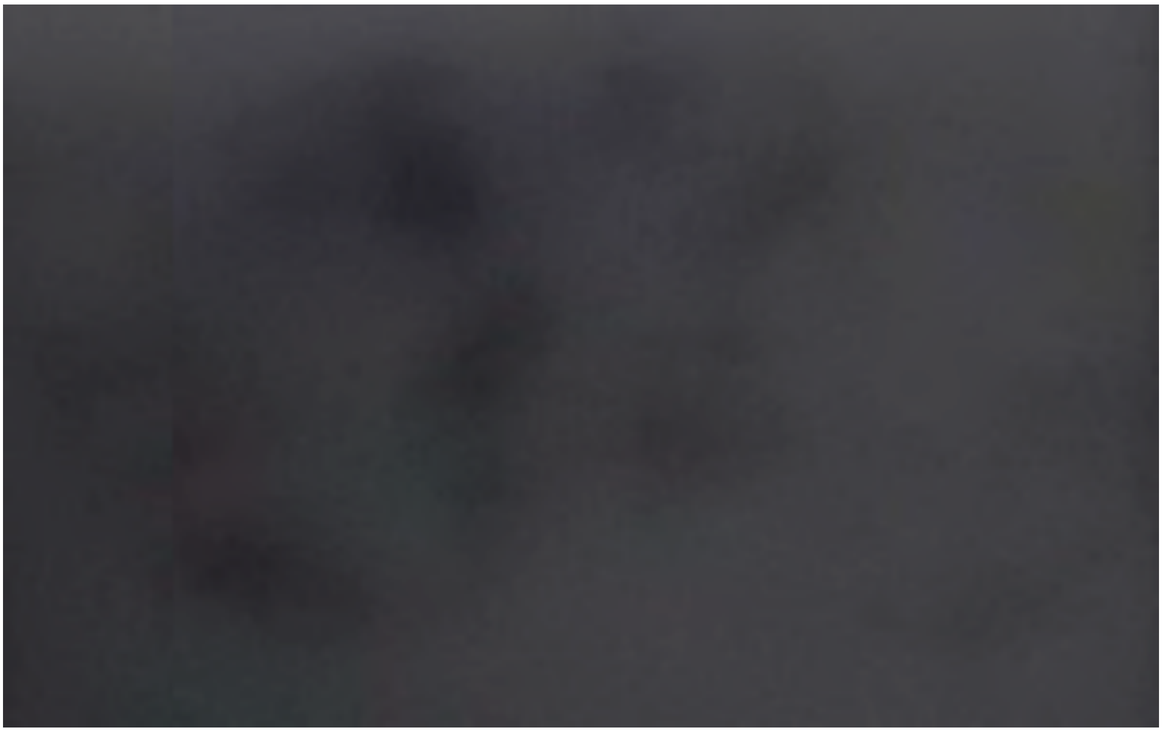

Dark-field macrophotography

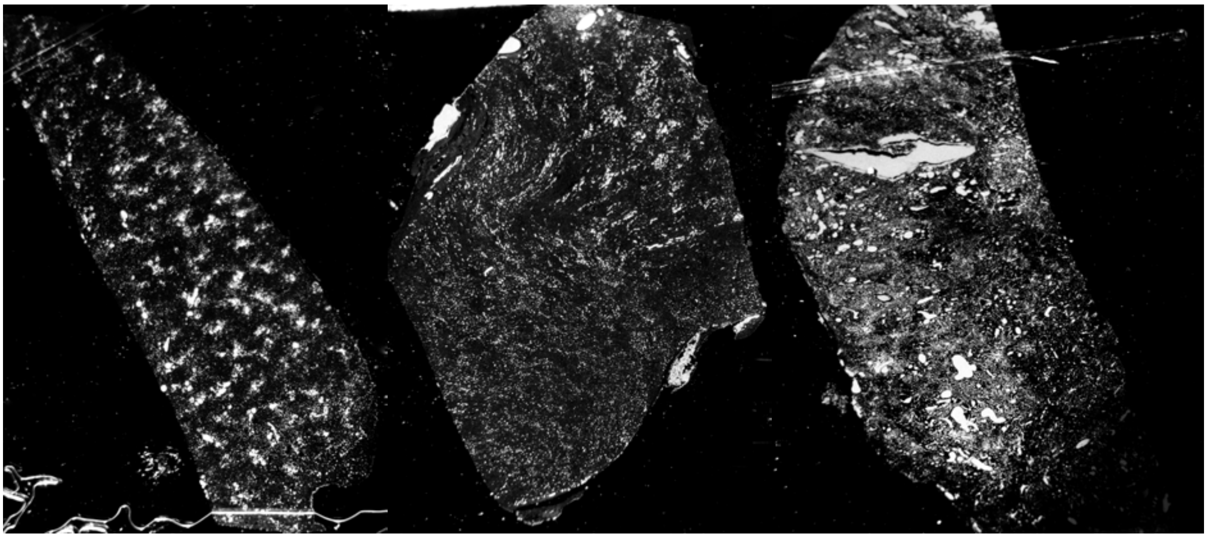

Cortex 26:1 P 0.18   Medulla 26:13 P0.51   Tumour 26:24 P 0.85

Darkfield

Contrast CD31

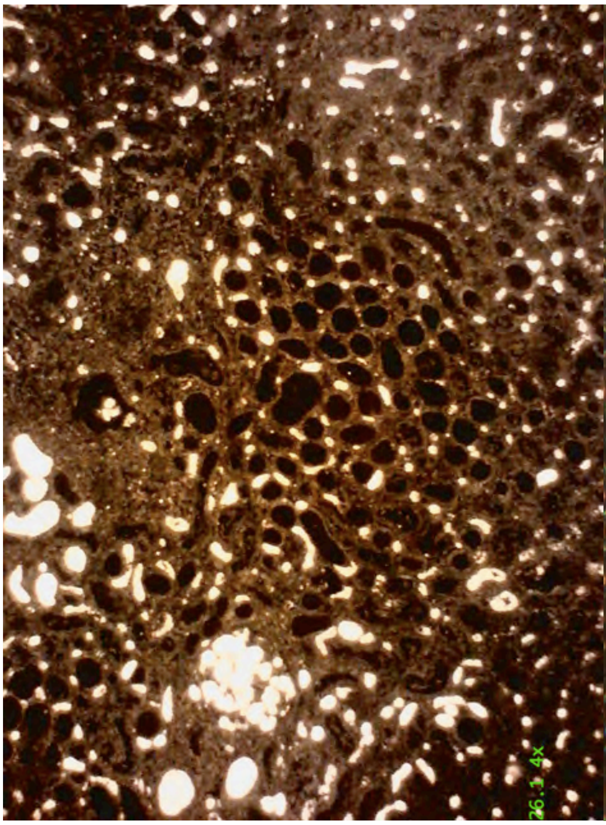

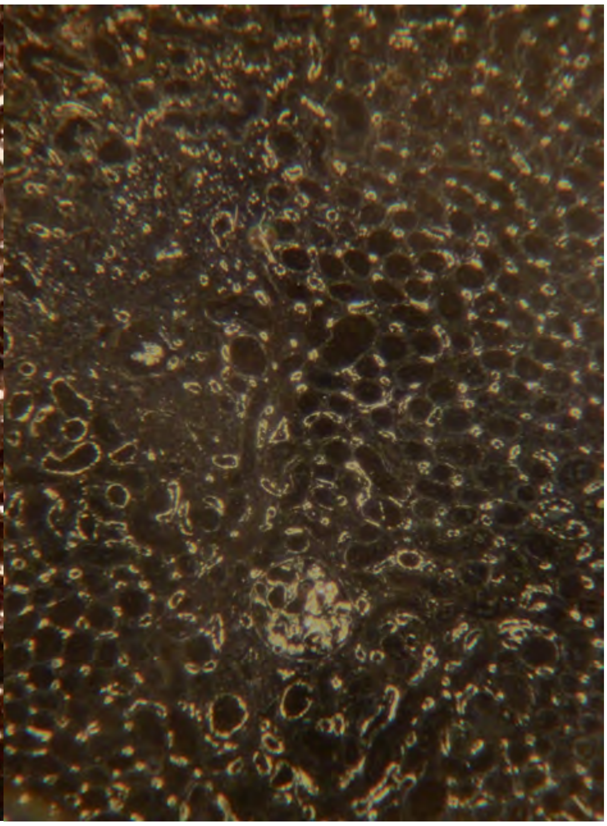

Cortex 26:1 PRU 0.18

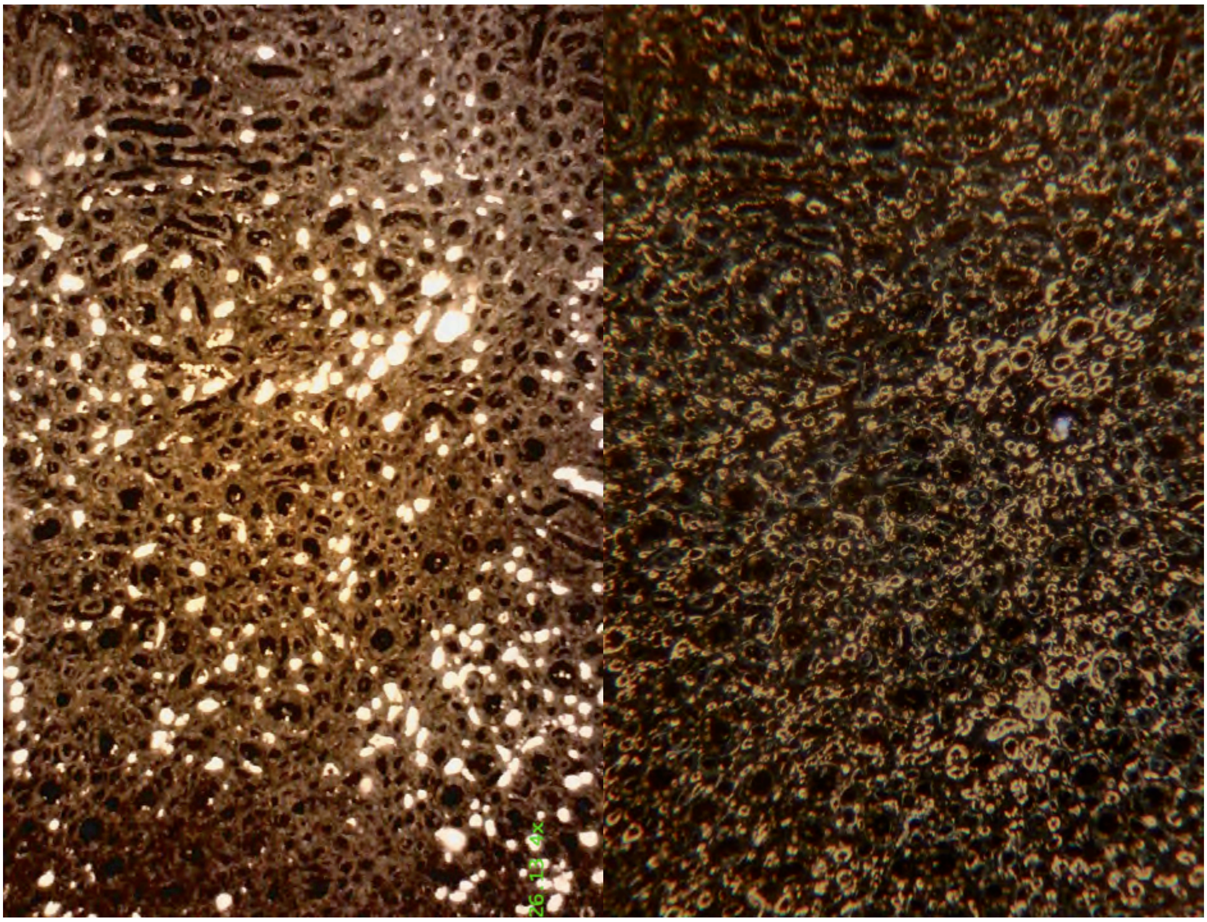

Medulla 26:13 PRU 0.51

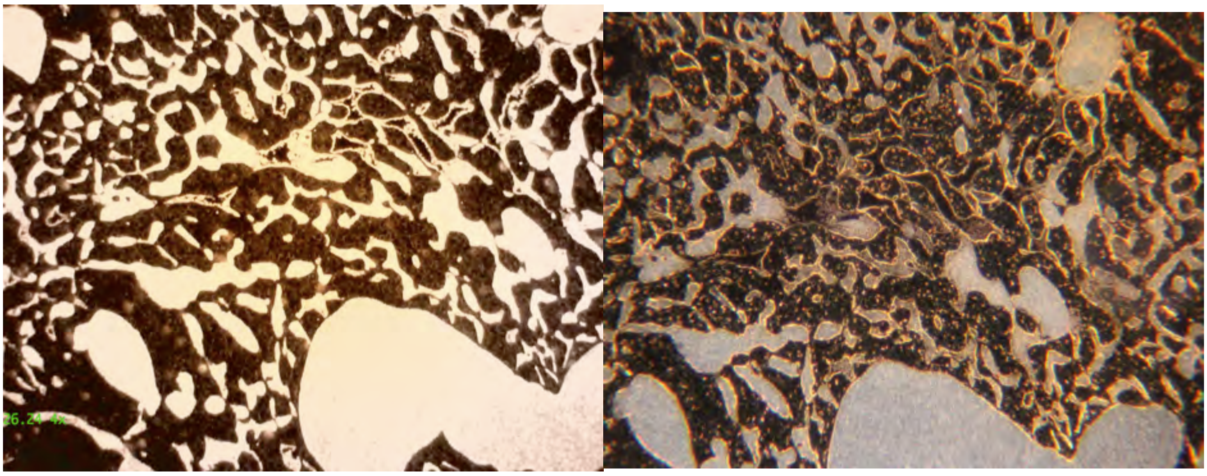

Tumour 26:24 PRU 0.85

Tumour samples HE stained 16x

Bright field Darkfield

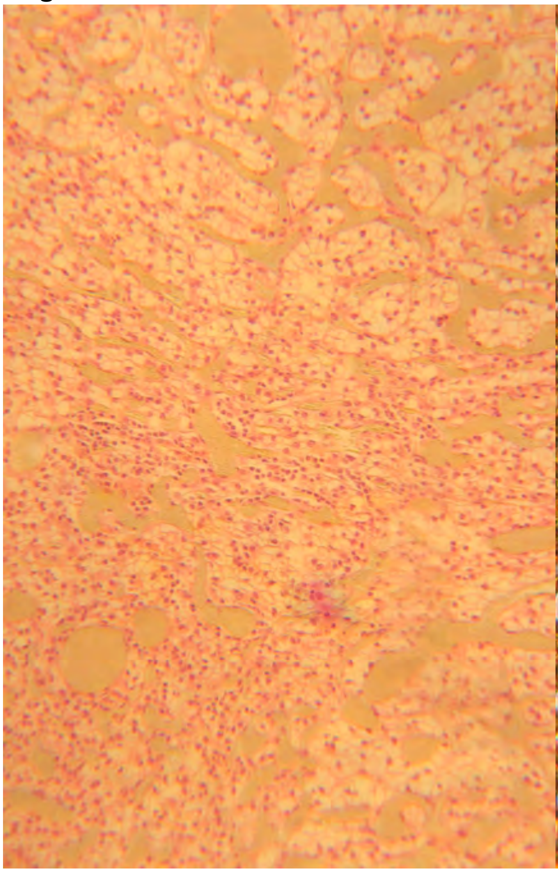 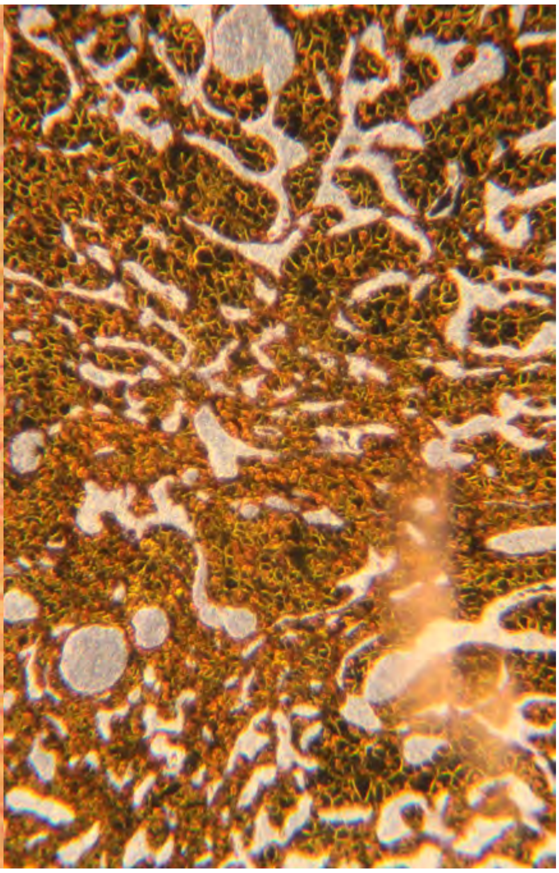

26:24 PRU 0.85

HE 20x

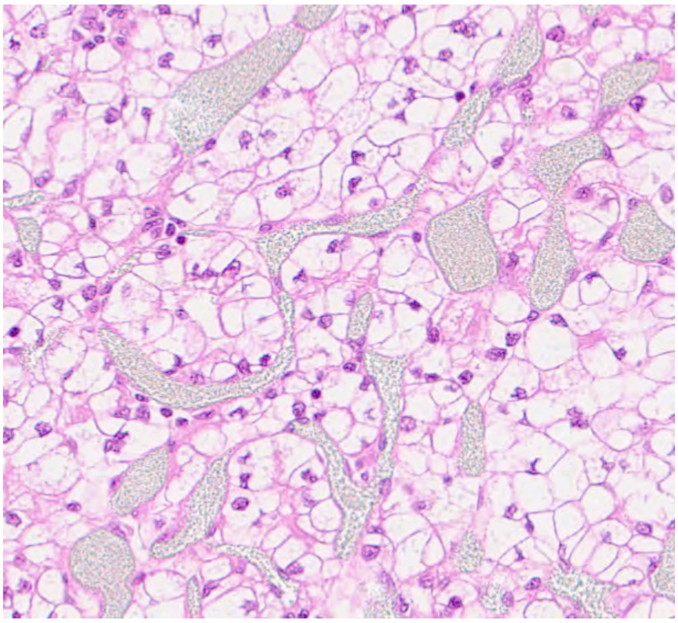

26:24 PRU 0.85

H27

Sex: Male
Age at surgery: 65 years
Survival from surgery:19 years
Cause of death: other cause
Initial stage: 10 cm pT2 NX M0
Tumour type CCRCC with rhabdoid differentiation. ISUP grade 4.

Specimen weight: NE
Perfusion pressure: 38 mmHg. Perfusate flow: 55 mL/min
Specimen PRU: 0.69
Cortical tissue PRU: 0.08 +/-0.02 n=9
Tumour tissue PRU span: 0.48-3.3 n=7

Angiography

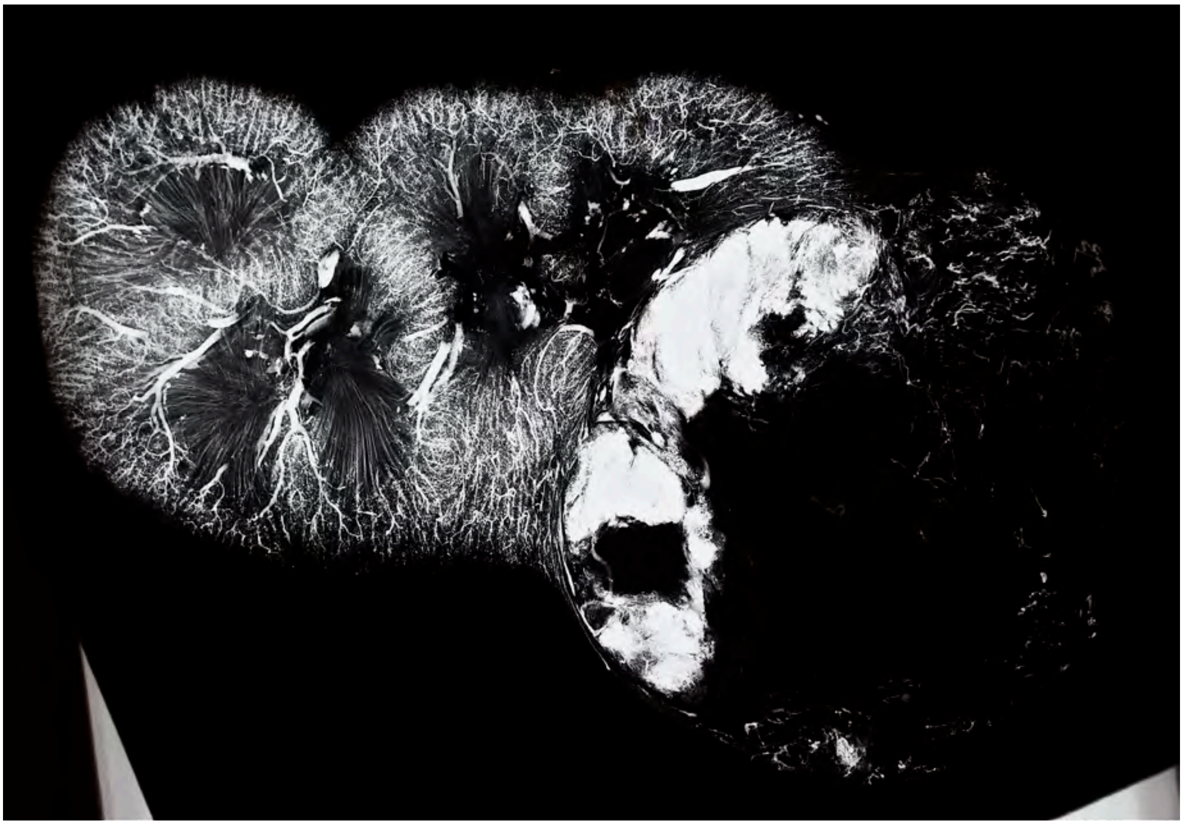

Partly well vascularized, mostly avascular. Heterogenous trapping of 15 um spheres

Autoradiography

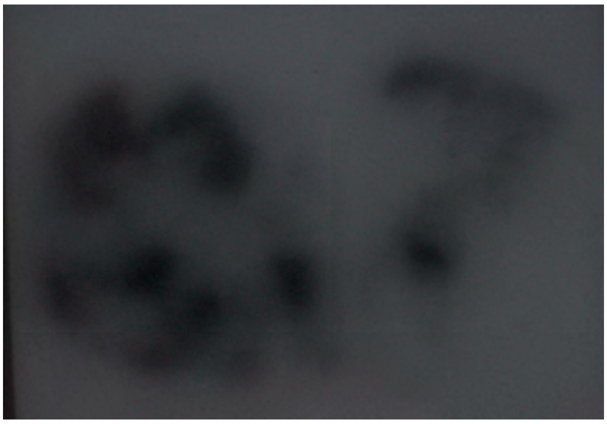

Dark-field macrophotography

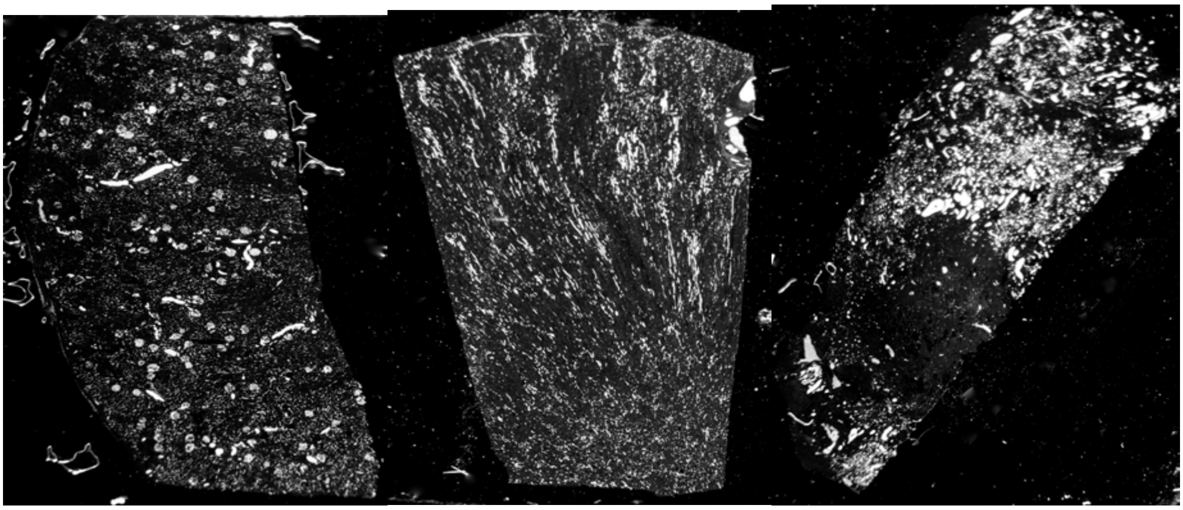

Cortex 27:3 PRU 0.08   Medulla 27:10 P 0.37   Tumour 27:18 P 2.9

Darkfield
Contrast CD31

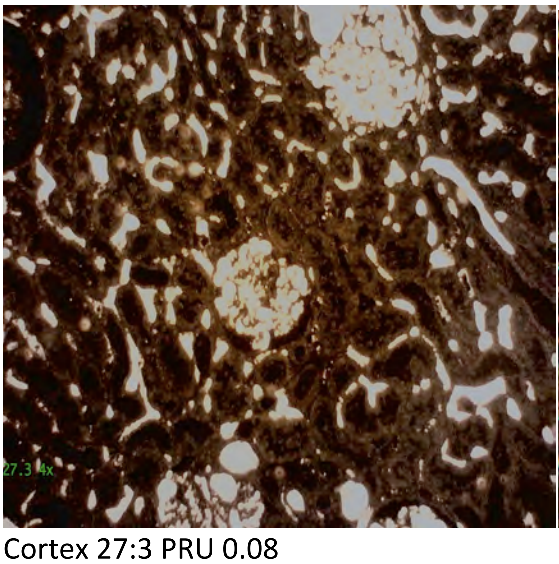

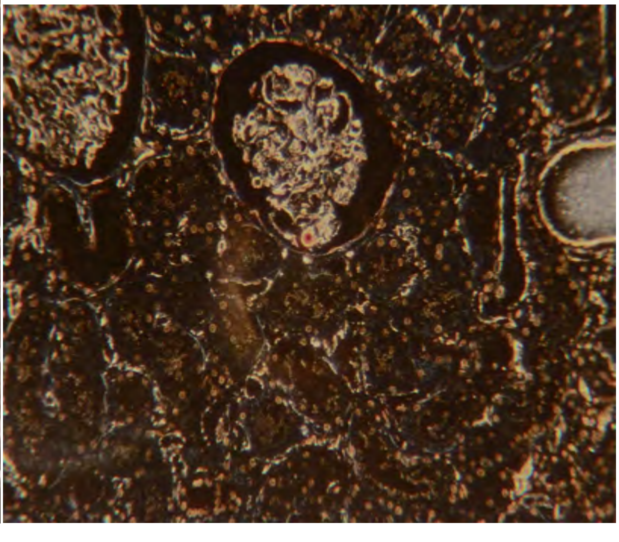

Cortex 27:3 PRU 0.08

Contrast CD31

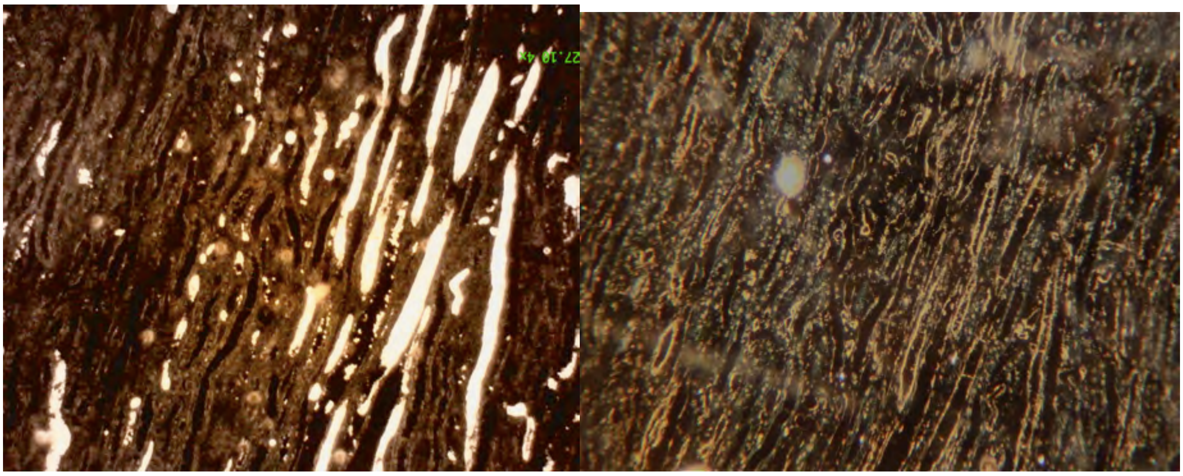

Medulla 27:10 PRU 0.37

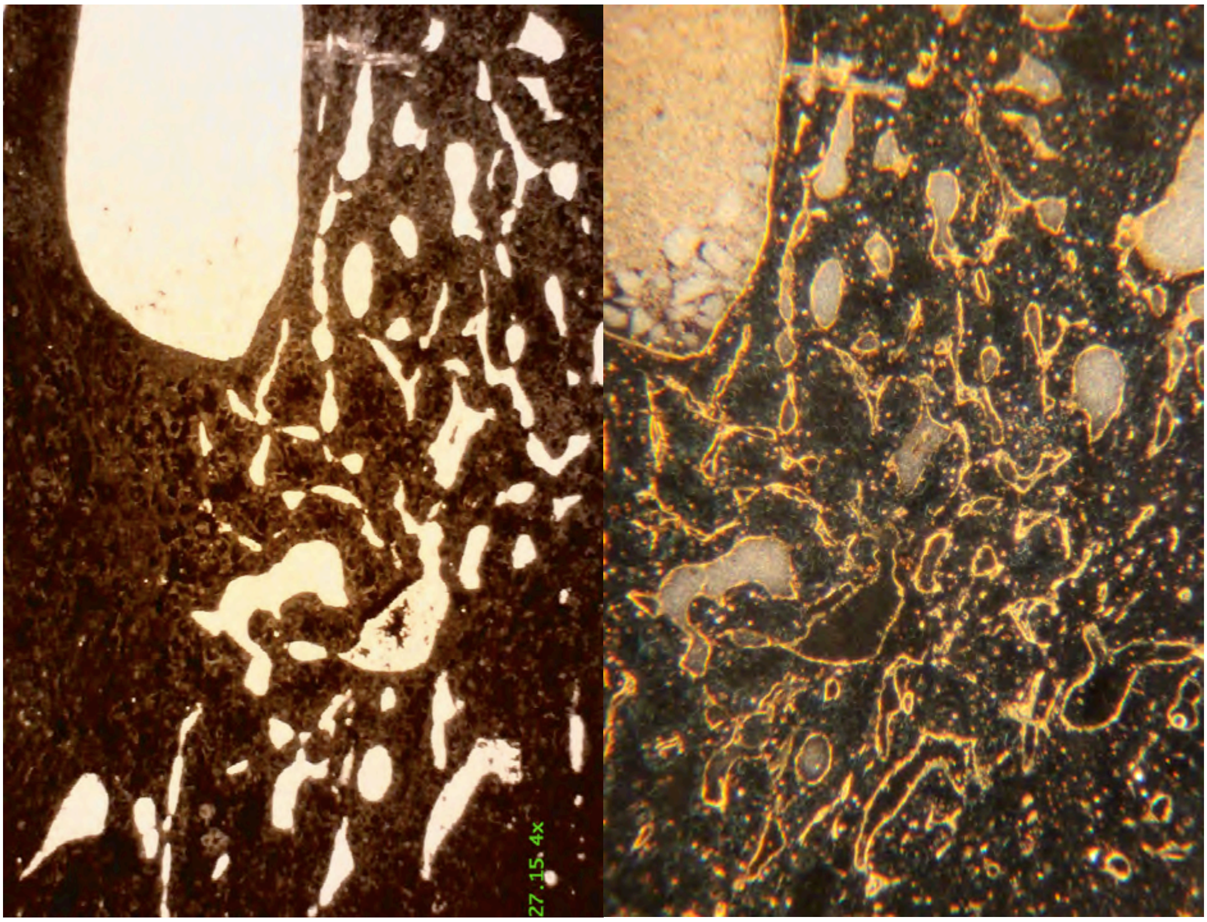

Tumour 27:15 PRU 1.11

Tumour samples HE stained 16x

Bright field

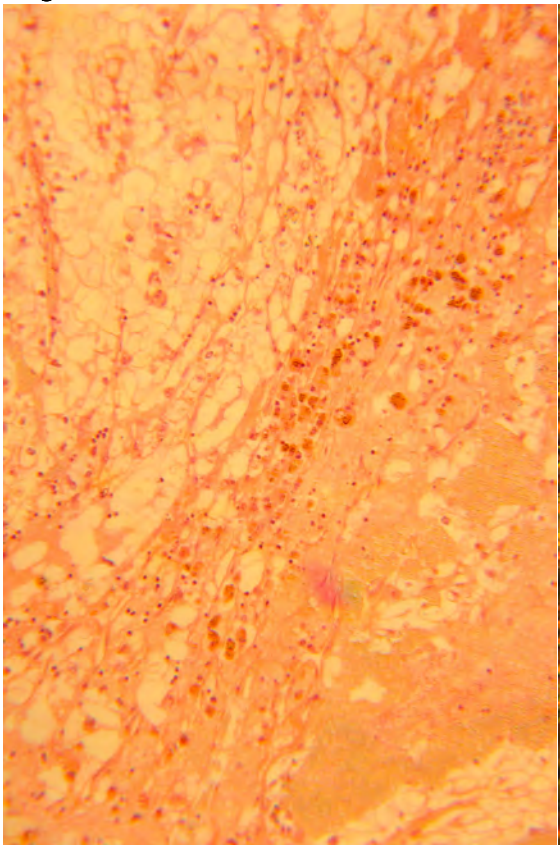

Darkfield

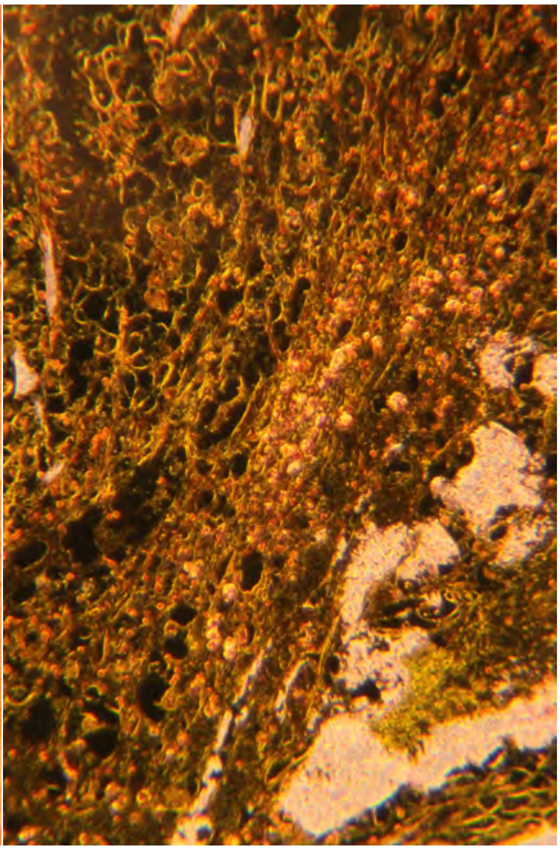

27:15 PRU 1.11

HE 20x

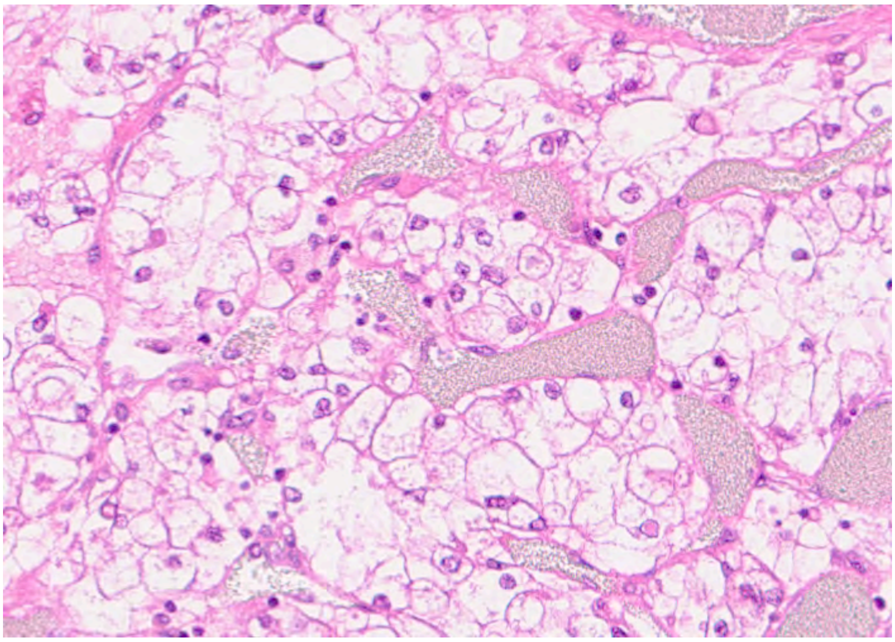

27:15 20x PRU 1.11

H28

Sex: Male
Age at surgery: 47 years
Survival from surgery: 35 years
Cause of death: other cause
Initial stage : pT1b N0 M0
Tumour type: CCRCC   ISUP grade 2

Specimen weight: NE
Perfusion pressure: 31 mmHg Perfusate flow: 39 mL/min
Specimen PRU: 0.79
Cortical tissue PRU: 0.22 +/- 0.04   n=9
Tumour tissue PRU span: 0.45-12.9 n=17

Angiography

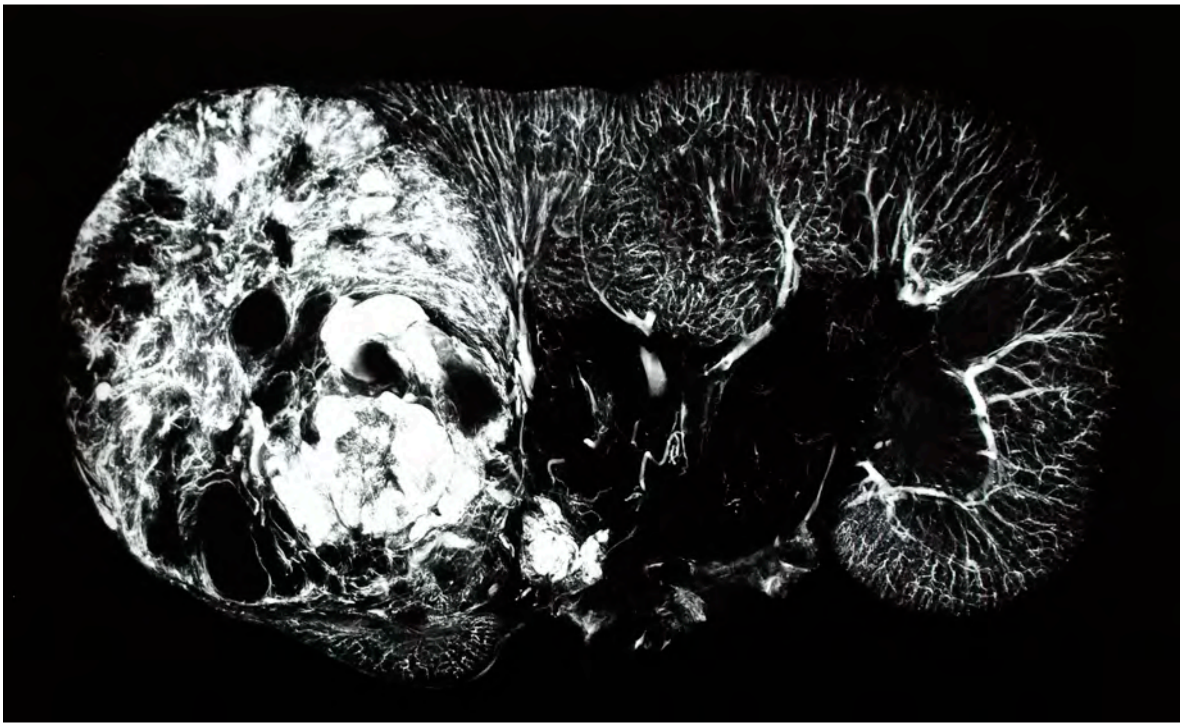

Heterogenously vascularized with poor 15 um sphere trapping

Autoradiography

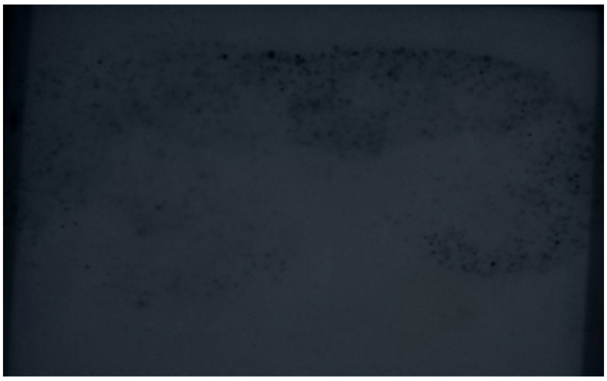

Dark-field macrophotography

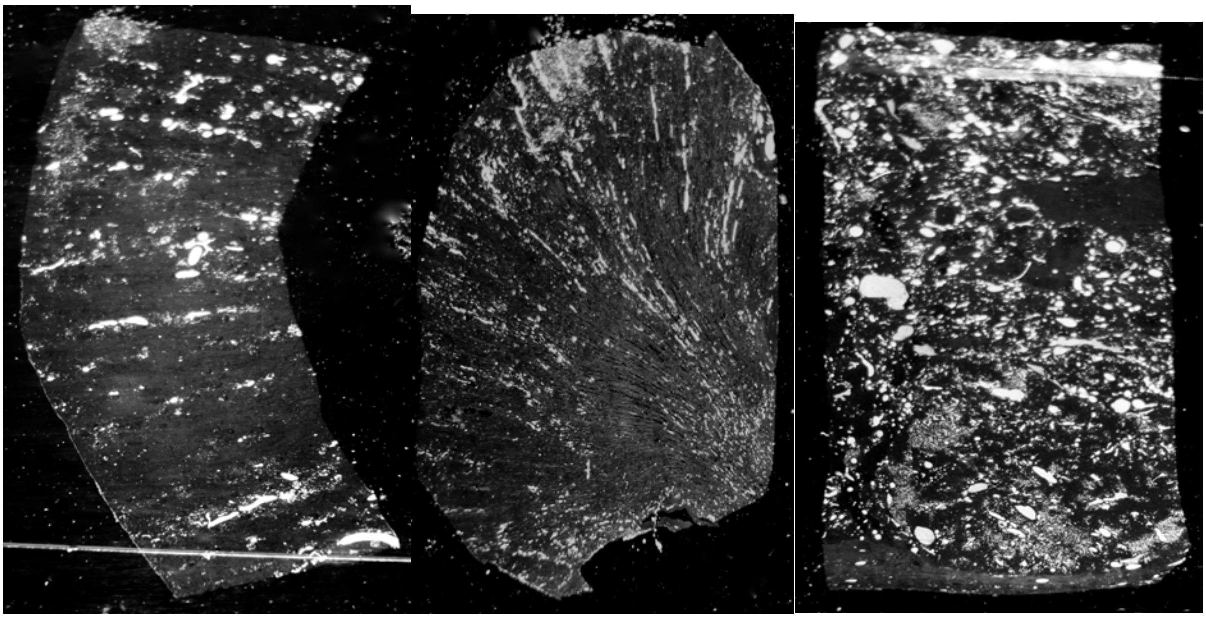

Cortex 28:4 P 0.2 Medulla 28:9 P 3.8 Tumour 28:13 P 0.63

Darkfield

Contrast CD31

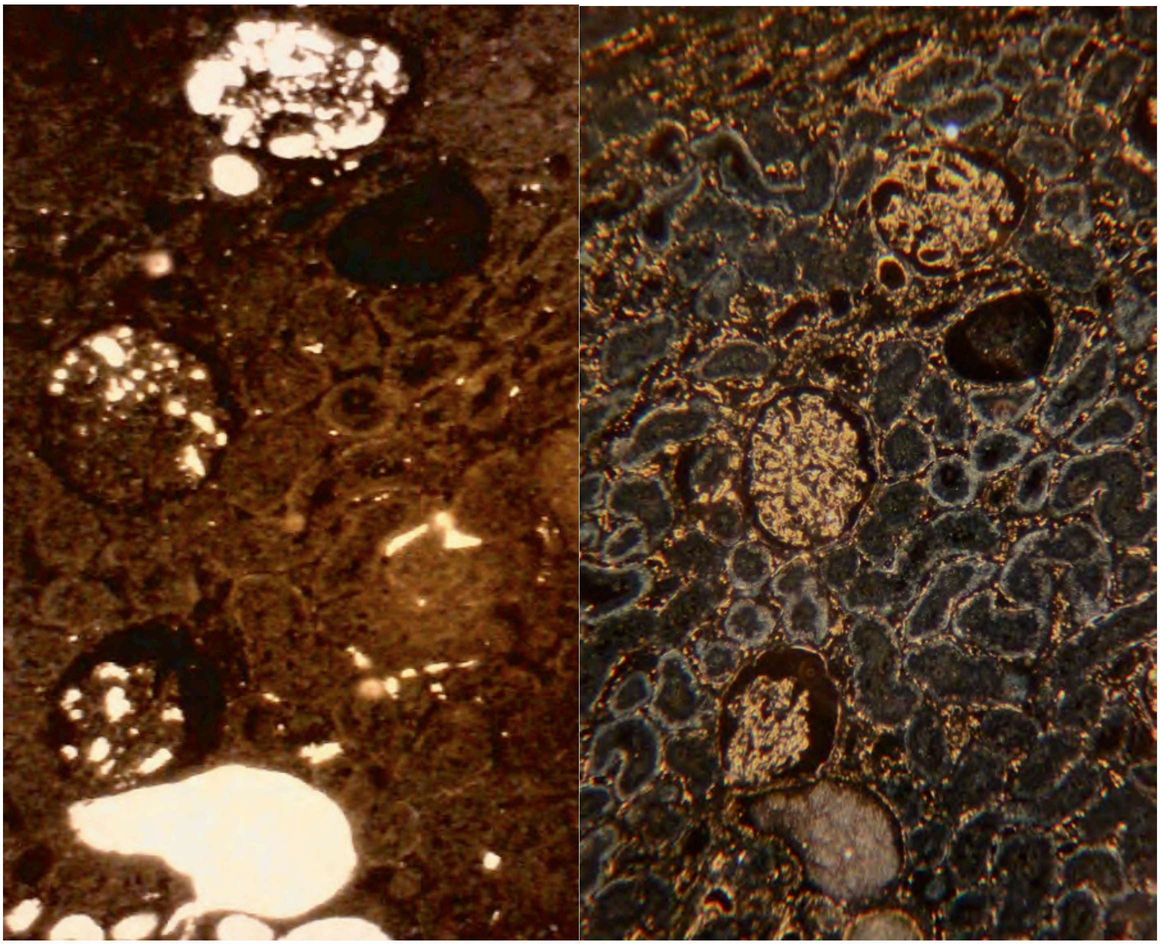

Cortex 28:4 PRU 0.2

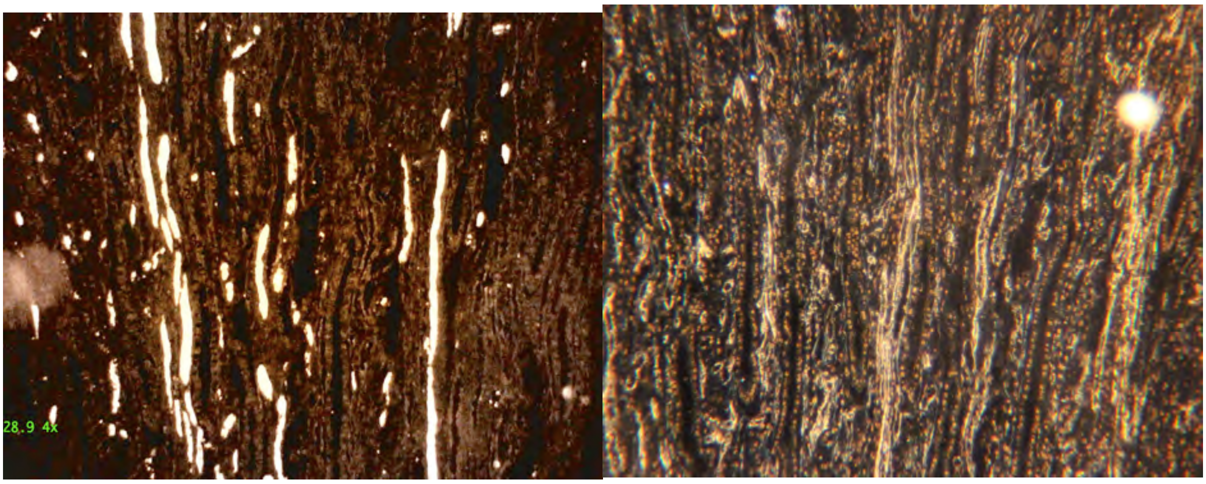

Medulla 28:9 PRU 3.8

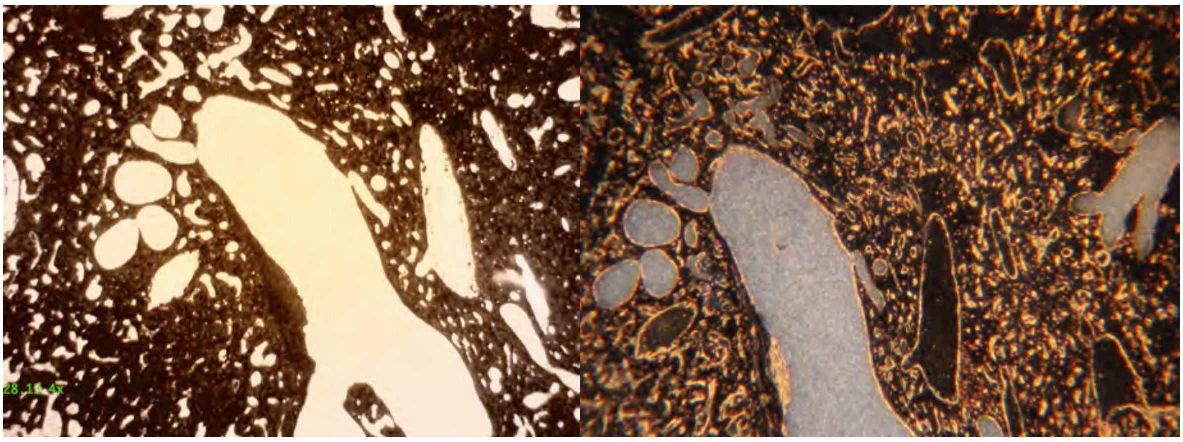

Tumour 28:13 PRU 0.63

Tumour samples HE stained 16x

Bright field Darkfield

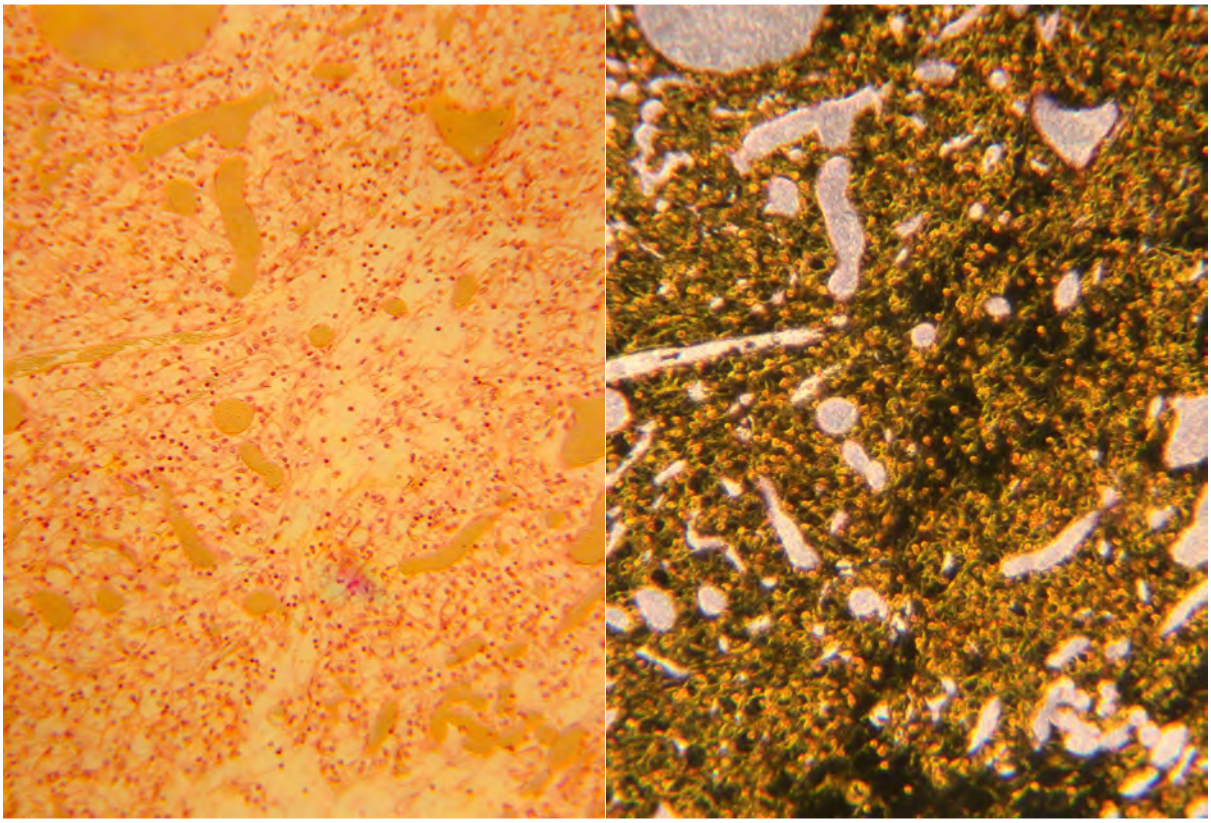

28:13 PRU 0.63

HE 20x

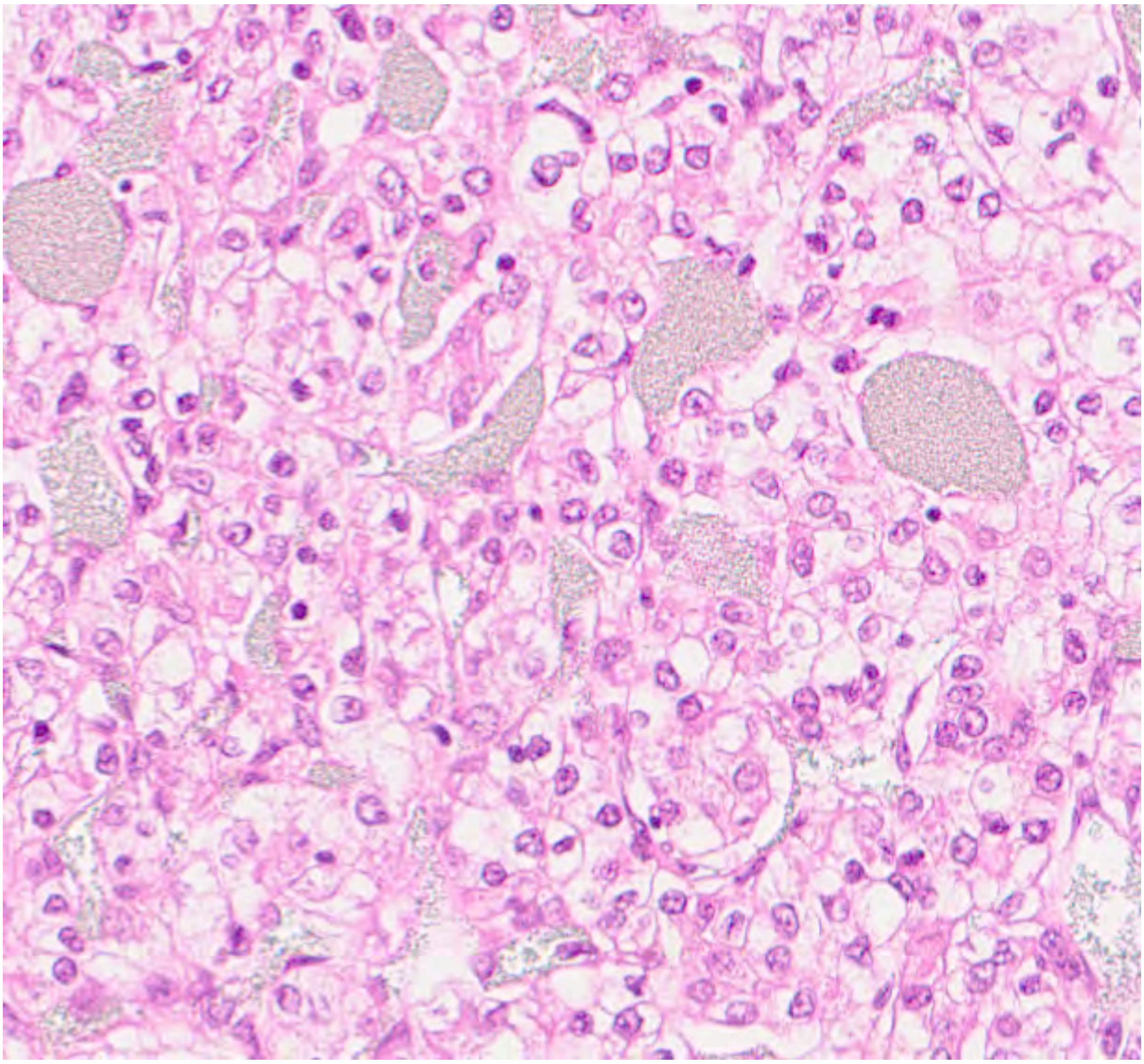

28:13 20x PRU 0.63

H29

Sex: Female
Age at surgery: 65 years
Survival from surgery: 24 years
Cause of death: other cause
Initial stage: NE
Tumour type: CCRCC, ISUP grade 1
Tumour volume: 62 cm^3

Specimen weight: NE
Perfusion pressure NE Perfusate flow: NE
Specimen PRU: NE
Cortical tissue PRU: NE
Tumour tissue PRU span: NE

Angiography

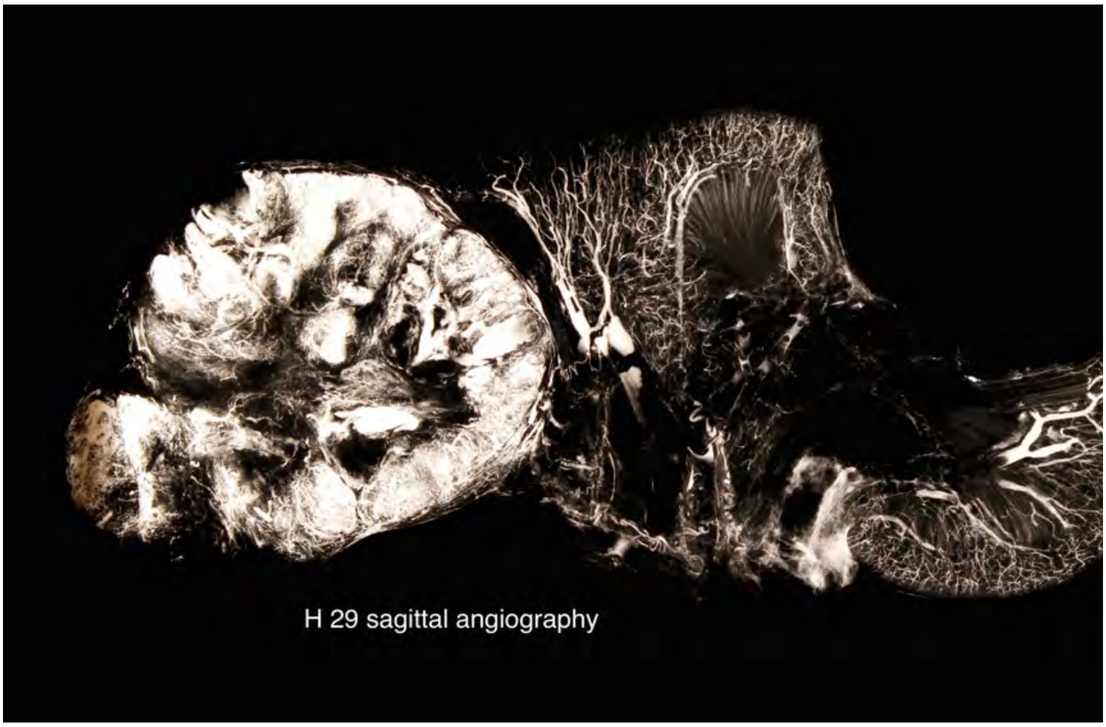

Periphery well vascularized with heterogenous 15 sphere trapping

Autoradiography

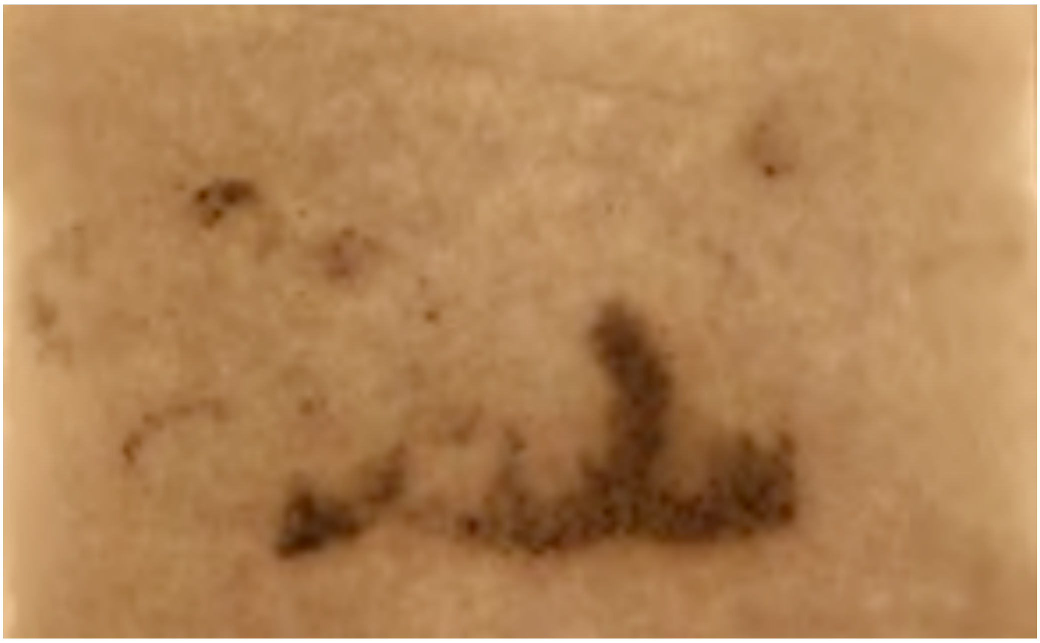

Darkfieldmacro

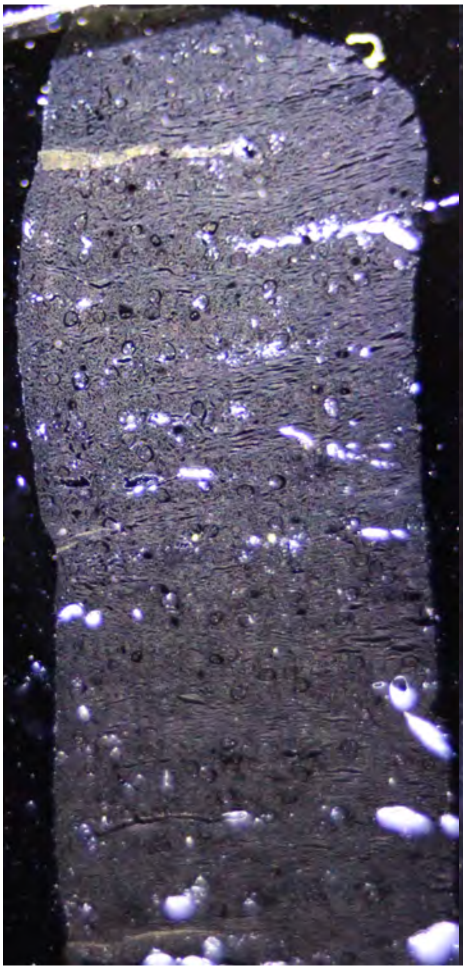

29:14 cortex

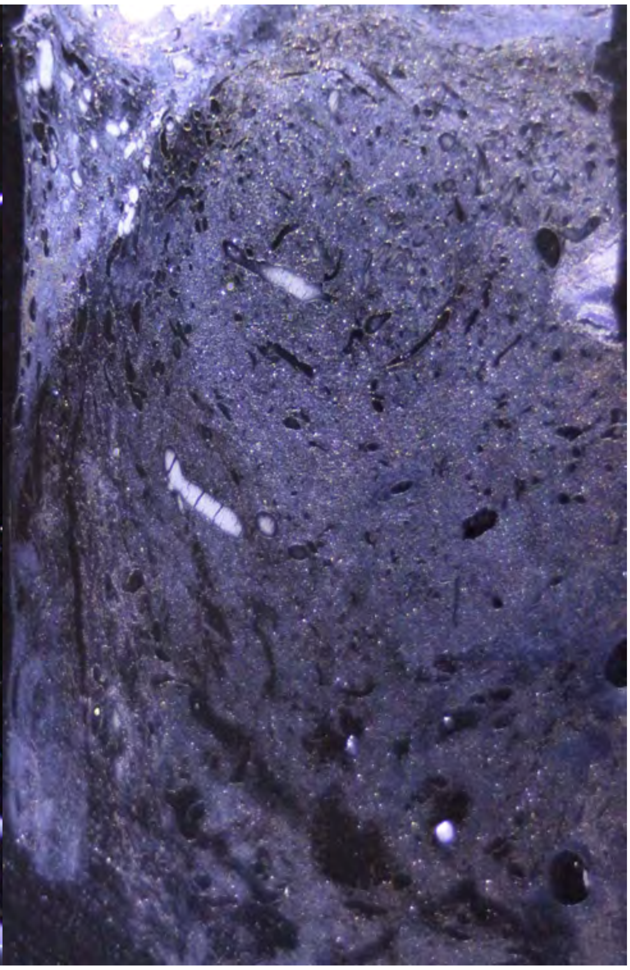

29:6 tumour

Darkfield CD31

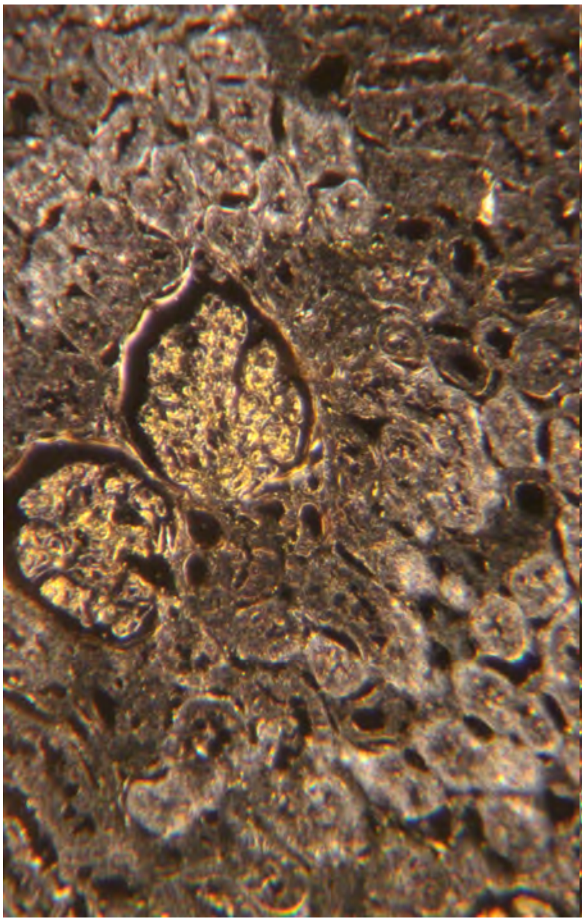

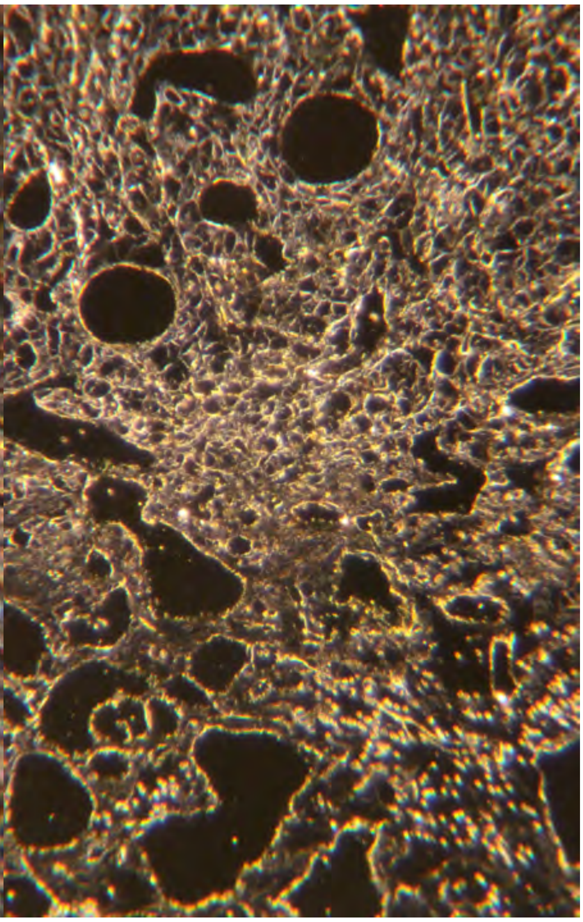

Cortex 29:18 PRU NE

Tumour 29:6 PRU NE

Tumour HE 16x

Brightfield

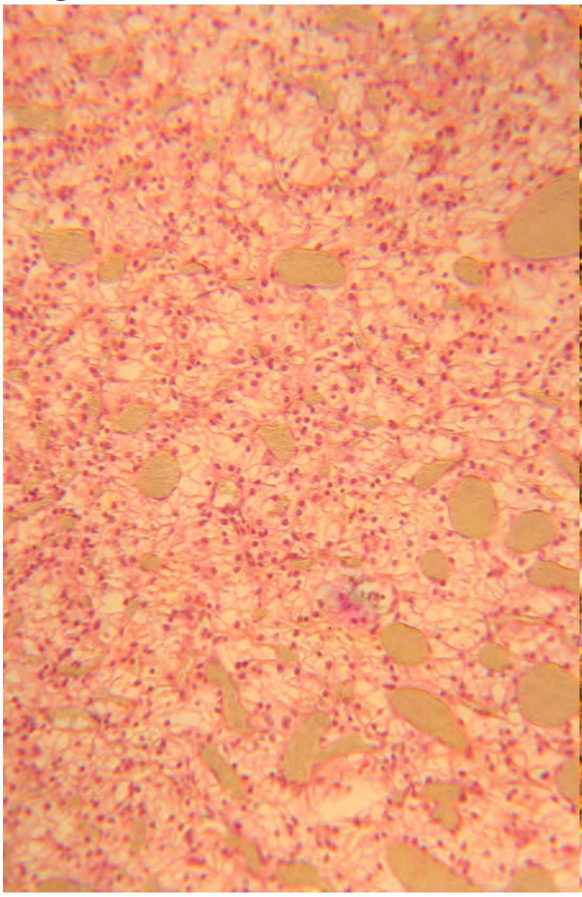

Darkfield

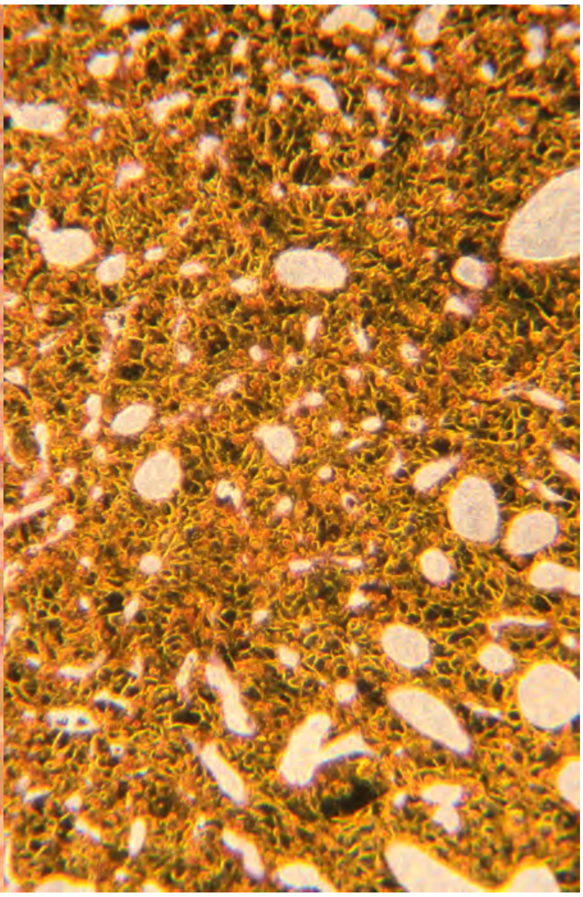

29:6 PRU NE

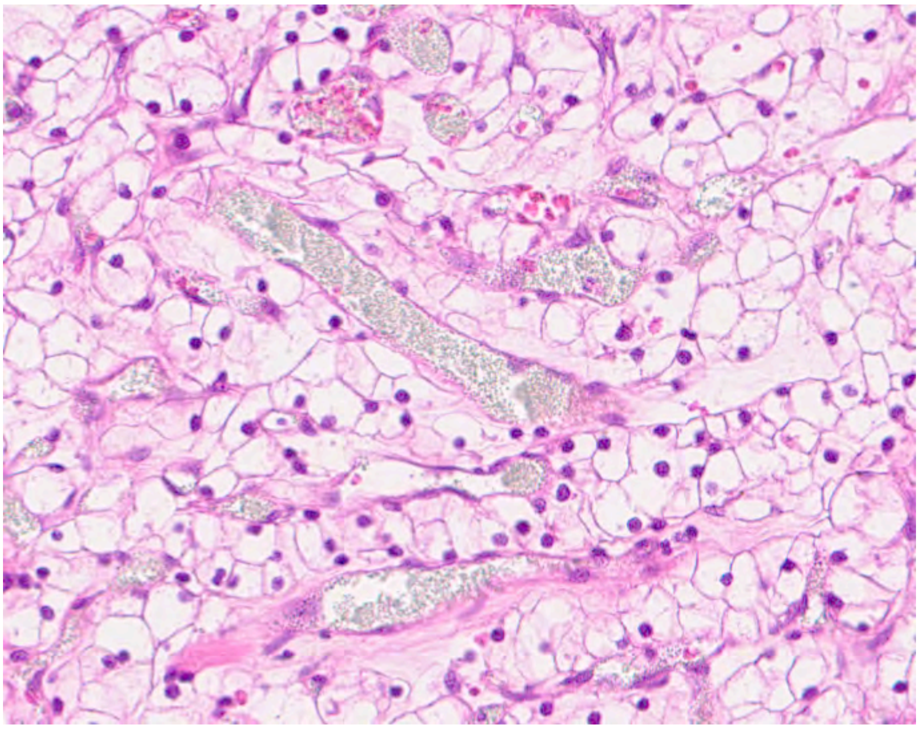

29:6 20x PRU NE
